# Implementing health promotion programmes in schools: a realist systematic review of research and experience in the United Kingdom

**DOI:** 10.1186/s13012-015-0338-6

**Published:** 2015-10-28

**Authors:** M. Pearson, R. Chilton, K. Wyatt, C. Abraham, T. Ford, HB Woods, R. Anderson

**Affiliations:** 1 Institute of Health Research, University of Exeter Medical School, St Luke’s Campus, Exeter, EX1 2LU UK; 2 School of Health and Related Research, University of Sheffield, Regent Court, 30 Regent Street, Sheffield, S1 4DA UK

**Keywords:** Health promotion in schools, Implementation, Evaluation, Realist review, Public health

## Abstract

**Background:**

Schools have long been viewed as a good setting in which to encourage healthy lifestyles amongst children, and schools in many countries aspire to more comprehensive, integrated approaches to health promotion. Recent reviews have identified evidence of the effects of school health promotion on children’s and young people’s health. However, understanding of how such programmes can be *implemented* in schools is more limited.

**Methods:**

We conducted a realist review to identify the conditions and actions which lead to the successful implementation of health promotion programmes in schools. We used the international literature to develop programme theories which were then tested using evaluations of school health promotion programmes conducted in the United Kingdom (UK). Iterative searching and screening was conducted to identify sources and clear criteria applied for appraisal of included sources. A review advisory group comprising educational and public health practitioners, commissioners, and academics was established at the outset.

**Results:**

In consultation with the review advisory group, we developed four programme theories (*preparing for implementation*, *initial implementation*, *embedding into routine practice*, *adaptation and evolution*); these were then refined using the UK evaluations in the review. This enabled us to identify transferable mechanisms and enabling and constraining contexts and investigate how the operation of mechanisms differed in different contexts. We also identified steps that should be taken at a senior level in relation to *preparing for implementation* (which revolved around negotiation about programme delivery) and *initial implementation* (which centred on facilitation, support, and reciprocity—the latter for both programme deliverers and pupils). However, the depth and rigour of evidence concerning *embedding into routine practice* and *adaptation and evolution* was limited.

**Conclusions:**

Our findings provide guidance for the design, implementation, and evaluation of health promotion in schools and identify the areas where further research is needed.

**Electronic supplementary material:**

The online version of this article (doi:10.1186/s13012-015-0338-6) contains supplementary material, which is available to authorized users.

## Background

Schools have long been viewed as a good setting in which to encourage healthy lifestyles and choices amongst children. The practice of health promotion in schools has been reinforced by the World Health Organization’s (WHO) *Ottawa Charter for Health Promotion* [1], the lifetime health and well-being benefits for children and communities that are expected to follow [2], and latterly by research evidence of synergy between health and education [3]. Schools in many countries aspire to more comprehensive, integrated approaches to health promotion which address both individuals’ attitudes and behaviours and the school environment [4, 5]. The WHO concept of health-promoting schools [2, 6, 7], known also as a ‘settings’ approach [8] and in North America as coordinated school health programmes, provides a framework for those approaches which incorporate a formal health curriculum; promotion of a healthy school environment and ethos that can benefit pupils, teachers, and non-teaching staff alike; and engagement with families and communities [9].

Recent reviews have identified evidence about the contribution that comprehensive, integrated approaches to health promotion in schools can make to improving children’s and young people’s health in a number of areas [9, 10]. Understanding of *how* these effects are attained is increasing [11]. However, understanding of how the constituents of such programmes can be best *implemented* in schools remains a neglected area [12].

The reality of implementing health promotion programmes in schools involves the active engagement of a range of actors [13, 14] and the adaptation of programmes to local contexts [15, 16] within a wider educational and public health system. They can thus be considered as complex interventions (multi-component, context-sensitive, and highly dependent on the behaviours of participants and providers) within a complex system [17].

Our aim was to identify the conditions and actions which lead to the successful implementation of health promotion programmes in schools (see Table 1 for definitions) taking full account of these complexities. Our research questions were as follows:

**Table 1:** Definition of terms used in the review

**Implementation**
The varied aspects and means by which an intervention (or programme) is integrated into one or a number of organisations. Implementation is the critical gateway between an organisational decision to adopt an intervention and the routine use of that intervention; the transition period during which individuals become increasingly skilful, consistent, and committed in their use of an intervention.
Adapted from Damschroder et al. [41]
**Health promotion programme**
To distinguish our focus from broad, often national, policies and programmes, we defined school health promotion programmes as a designated combination of activities, learning materials, and messages which:
• are intended to achieve specific health promotion, health education, or healthy behaviour goals in pupils
• can be adopted and adapted within schools (e.g. whole years or classes)
• involve the dedicated time of pupils within school in order to participate in or learn from the programme
Such programmes may have been developed within a school or as part of a wider (e.g. research-based) initiative. They could be delivered in particular lessons or times within the school day (e.g. Personal, Social and Health Education lessons), before or after school (e.g. after-school gardening club), or have their messages and learning materials delivered within the lessons of other subjects.
**Realist review terms**
Adjudicate—To make a judgement about *methodological quality* or *applicability in this instance* and account for this judgement based on findings from the use of the critical appraisal tool or an explicit argument about why a piece of evidence was not applicable
Consolidate—To bring together. In a realist synthesis, ‘to bring together *into a more coherent whole*’
Context—The wider configuration of factors, not necessarily connected to a programme, which may enable or constrain the operation of specific mechanisms
Juxtapose—To place two or more things (evidence fragments) together, especially in order to suggest a link between them or emphasise the contrast between them
Mechanism—The way in which a programme’s resources or opportunities interact with the reasoning of individuals and lead to changes in behaviour
Programme theory—A model linking outcomes to programme activities and the underlying theoretical assumptions of a programme or intervention [20]. These models contain, even if they do not explicitly state, ideas about how a problem can be best addressed and how factors that may undermine the actions of a programme can themselves be addressed [24]
Reconcile—To make two or more apparently conflicting things (evidence fragments) consistent or compatible
Situate—To place something (a piece or pieces of evidence) in a context or set of circumstances and show the connections (between it/them and other evidence fragments)

1. What are the main factors or mechanisms that are thought to explain the success or failure of the implementation of health promotion programmes in schools?
2. Is there an association between these factors and mechanisms and the successful implementation of health promotion programmes in schools?
3. For what public health problems and in what circumstances do schools provide a feasible and sustainable setting for health promotion in the United Kingdom?

## Methods

We chose to conduct a realist review in order to attain a contextualised understanding of *how* and *why* complex interventions achieve particular effects—in realist terminology (see Table 1), how mechanisms lead to outcomes in particular contexts [18, 19]. The realist approach involves testing ‘programme theories’—often expressed as a model linking outcomes to programme activities and the underlying theoretical assumptions [20]. This approach has commonalities with other approaches such as Intervention Mapping [14] and Medical Research Council guidance on process evaluation of complex interventions [21], but differs by being explicitly situated in a realist philosophy of science [22, 23]. A realist philosophy of science posits that the identification and testing of contextualised, generative mechanisms provide the greatest explanatory potential for phenomena and the strongest basis for inferring how mechanisms will operate in other contexts [23]. It differs from an ‘idealist’ philosophy that endeavours to establish causation by ruling out alternative atheoretical patterns.

Contained within programme theories, even if not explicitly stated, are ideas about how a problem can best be addressed and how factors that may undermine the actions of the programme can themselves be addressed [24]. Realist review methods have been specifically advocated for evaluating evidence about complex interventions and their implementation [19, 25]. For this topic, three factors led us to choose realist review over a mediator and moderator analysis. First, the measures (or ‘markers’) of implementation are not well-developed or standardised. Second, we envisaged that the diversity of trial methodology and complexity of the relationships between phenomena would be likely to preclude use of meta-regression of multivariate studies. Third, the diversity of qualitative and quantitative research evidence required a coherent approach for synthesis.

The review was conducted in two phases. First, ideas about what enables or inhibits the implementation of health promotion programmes in schools (programme theories) were identified from a range of published and other sources. Second, these programme theories were tested (challenged, endorsed, and/or refined) using evidence from evaluations of United Kingdom (UK) school health promotion programmes. We endeavoured to identify mechanisms (how a programme’s resources or opportunities interact with the reasoning of individuals and lead to changes in behaviour) and contexts (the wider configuration of factors, not necessarily connected to a programme, that may enable or constrain the operation of specific mechanisms) so that context-mechanism-outcome configurations could be specified. The identified evidence meant that this was possible to a much greater extent in relation to earlier (preparation and initial implementation) rather than later (embedding and evolution) stages of implementation. Our focus on implementation therefore includes intervention delivery characteristics that are often evaluated in conventional effectiveness studies [26] but extends this focus to include levels of complexity about those delivering a programme and the system in which they practise.

The full protocol for the review has previously been published [27]. The review is reported in accordance with the RAMESES publication standards [28].

### Search strategy

Our approach to searching was iterative, consisting of sensitising, wide-ranging, and supplementary searches. This enabled us to map and explore a wide range of conceptual sources relating to the implementation of health programmes (both in schools and other settings), whilst also locating empirical studies conducted in the UK for programme theory testing.

### Screening (theory-development stage)

The first stage of the review was designed to both identify and develop programme theories and to ‘map the terrain’ of the implementation of health promotion programmes in schools in Organisation for Economic Co-operation and Development (OECD) countries. Sources that provided rich descriptions of the delivery of school-based health promotion for children aged 5–16 years in any OECD country were included. These included editorials, opinion pieces, commentaries, comparative effectiveness studies, process evaluations, qualitative research, and systematic reviews.

Titles and abstracts were read by the reviewers (MP, RC) to identify key ‘implementation’ terms and synonyms that could inform the development with the information specialist (HW) of the ‘sensitising’ search strategy. Key documents relating to the implementation of health programmes in schools were also identified and used to search for other documents which had cited them. We deliberately used a wide definition of ‘key’—for example, sources could be considered ‘key’ because they were a candid reflection on the implementation of a health promotion programme or because they were strongly conceptualised (i.e. a strongly theoretically informed inquiry). This stage was also used to ‘sensitise’ us as researchers to the emerging field of implementation science (as it related to health promotion in schools) and to potential programme theories.

However, we did not intend for the sensitising stage to be exhaustive—the aim was to locate a reasonable range of terms and sources that could inform further searches and deepen our understanding of the field (see Additional file 1 for record of (and reasons for) the sources obtained).

To help focus our identification and development of programme theories, we kept in mind examples of theories that struck a balance between being broad enough to identify a potentially significant relationship and specific enough to be testable (i.e. middle-range theories, the most useful theories on which we would focus in our development of bespoke programme theories). These middle-range theories can be thought of as lying between localised and non-theoretical individual examples or instances and broad, generic theories, both of which would be harder to test using information about particular programmes.

### Screening (theory-testing stage)

For inclusion in the second stage of the review, studies had to be linked to an empirical evaluation of a primary- or secondary-school-based health promotion programme in the UK (i.e. schools for children aged 5–16 years). For example, a process evaluation that documented implementation processes alongside a trial was considered to be ‘linked’. We included evaluations that used a range of comparative study designs—RCTs, controlled before and after studies, and before and after studies. Detailed inclusion criteria are described in the protocol [27].

Screening was conducted by two reviewers (MP, RC) using EPPI-Reviewer 4 (EPPI-Centre, Social Science Research Unit, Institute of Education) to manage references and record coding decisions.

**Fig. 1:**
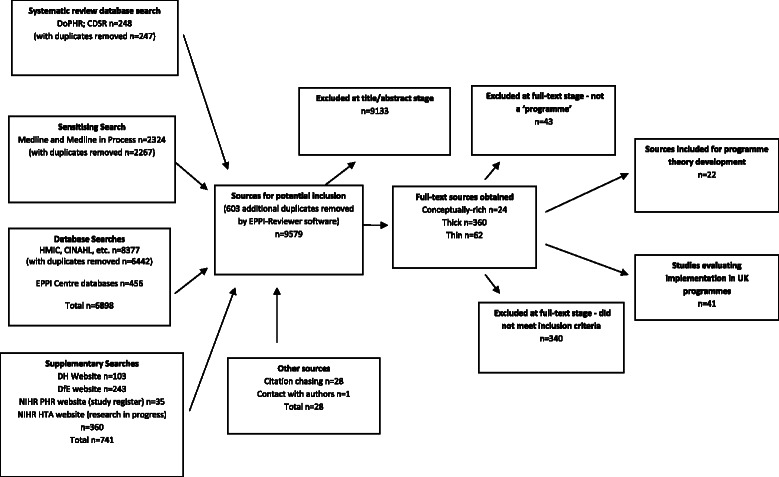
Flowchart of sources through the review

We ‘mapped’ sources for both theory-development and theory-testing stages in two main ways. We first used the abstract to assess the likely clarity, richness, and extent of conceptualisation of programme theories that a source could potentially provide. This assessment used criteria proposed by Ritzer [29] and Roen et al. [30] (Table 2). We then categorised sources by ‘type’—policy document, editorial, opinion piece or letter, commentary, reflection on practice, comparative effectiveness study, evaluation and/or process evaluation, qualitative research, survey, systematic review, narrative review, or conceptual review. This enabled us to use a sampling strategy which focused on those sources that would potentially contribute the most to the development of a conceptual framework (i.e. those that were ‘conceptually rich’). It also enabled us to purposively sample ‘less conceptually rich’ sources such as policy documents or editorials that could nevertheless contain important contributions for the development of a conceptual framework. This strategy was informed by the idea of ‘theoretical saturation’, where data collections stops at the point at which collection of further data is considered unlikely to yield further insights [31]. The flow of studies through the review is shown in Fig. 1.

**Table 2:** Criteria used for assessing the conceptual richness of sources

‘Conceptually rich’ [29]	‘Thicker description’ [30] but not ‘conceptually rich’	‘Thinner description’ [30]
Theoretical concepts are unambiguous and described in sufficient depth to be useful	Description of the programme theory or sufficient information to enable it to be ‘surfaced’	Insufficient information to enable the programme theory to be ‘surfaced’
Relationships between and amongst concepts are clearly articulated	Consideration of the context in which the programme took place	Limited or no consideration of the context in which the programme took place
Concepts sufficiently developed and defined to enable understanding *without* the reader needing to have first-hand experience of an area of practice	Discussion of the differences between programme theory (the design and orientation of a programme—what was intended) and implementation (what ‘happened in real life’)	Limited or no discussion of the differences between programme theory (the design and orientation of a programme—what was intended) and implementation (what ‘happened in real life’)
Concepts grounded strongly in a cited body of literature	Recognition and discussion of the strengths and weaknesses of the programme as implemented	Limited or no discussion of the strengths and weaknesses of the programme as implemented
Concepts are parsimonious (i.e. provide the simplest, but not over-simplified, explanation)	Some attempt to explain anomalous results and findings with reference to context and data	No attempt to explain anomalous results and findings with reference to context and data
-	Description of the factors affecting implementation	Limited or no description of the factors affecting implementation
-	Typified by	Typified by
	Terms—‘model’, ‘process’, or ‘function’	Mentioning only an ‘association’ between variables
	Verbs—‘investigate’, ‘describes’, or ‘explains’	
	Topics—‘experiences’

### Development of programme theories

We recognised from the outset that building a conceptual framework for the implementation of health promotion programmes in schools would be a process that initially focused on discussion and debate within the research team. Both reviewers (MP and RC) read, annotated, and took notes from all of the sources categorised as conceptually rich (*n* = 19) with a view to producing a coherent framework that encompassed all of the implementation aspects identified in the sources. We pursued citations from these sources where we judged that they held potential to contribute substantively to the conceptual framework and, as a result, included two further sources. One was directly related to health promotion in schools [32] whilst one was not specific to health promotion but related to change at the level of the school [33]. One further source categorised as ‘thick’ was included [15] as it was closely linked with another conceptually rich source [34]. A total of 22 sources informed the development of the conceptual framework and theory-development review phase (Additional file 2).

Amongst these, a narrative review by Samdal and Rowling [5] presented a list of eight ‘rationales for implementation components’ that were presented in a form similar to programme theories. We took the decision to use these theories as our starting point from which to explore how the programme theories in the other 21 conceptually rich sources could expand or refine these theories. This enabled us to develop a ‘long list’ of 12 programme theories (Additional file 3) that encompassed all of the concepts around implementation identified in the 22 conceptually rich sources (including the Samdal and Rowling narrative review [5]) and to identify the unique contributions of each source. These programme theories were developed and prioritised further on the basis of discussions in our first review advisory group meeting with educational and health professionals and health researchers (see Additional file 4 for further details).

The final expression of four programme theories encompassed the processes of preparing for, introducing, embedding, and adapting health promotion programmes in schools. These were discussed and agreed via email correspondence with the advisory group’s members. The three stages of programme theory development, showing the areas in which different sources contributed, are documented in Additional file 5.

**Table 3:** Characteristics of included empirical UK studies and the programme theories (PT) for which they provided evidence

Programme details	Study type	How implementation assessed
Active primary school (pilot)		
*Co-ordinators worked with schools to provide opportunities for physical activity within and outside of the curriculum.*		
Physical activity		
Delivered to: pupils (5–11 years) (primary)		
Delivered by: co-ordinators		
Lowden et al. [42]	BA	Available resources
	Process evaluation	Adaptation
APPLES—the Active Programme Promoting Lifestyle Education in Schools		
*Multi-disciplinary health promotion team worked with schools to develop school action plans tailored to each school’s perception of needs.*		
Obesity		
Delivered to: pupils (8–10 years) (primary)		
Delivered by: health promotion team		
Sahota et al. [43]	CRCT	Engagement
	Process evaluation	
ASSIST—A Stop Smoking in Schools Trial		
*Pupils nominated ‘influential peers’ who received training to become ‘peer supporters’ who aimed to reduce/stop smoking amongst their peers.*		
Substance use (tobacco)		
Delivered to: pupils (12–13 years) (secondary)		
Delivered by: peers (12–13 years)		
Audrey et al. [44]	NA	Researchers’ reflections on programme implementation
Audrey et al. [45]	NA	NA
Audrey et al. [46]	Process evaluation	Adolescents’ perspectives
Audrey et al. [43]	Process evaluation	Teachers perceptions
Holliday et al. [47]	Process evaluation	Fidelity of delivery
Blueprint		
*15 lessons (‘normative’ focus) delivered over 2 years; parenting skill workshops; local media coverage, managed by media relations agency; education and training for local retailers; involvement of wider community of drug professionals and organisations.*		
Substance use (legal and illegal substances)		
Delivered to: pupils (11–13 years) (secondary)		
Delivered by: teachers		
Stead et al. [48]	CBA	Teacher training
	Process evaluation	Curriculum
		School drug advisor support for delivery in schools
Stead et al. [49]	Process evaluation	Fidelity
		Adaptation of content
		Teaching confidence
		Training
Blueprint Evaluation Team [50]	Evaluation	Materials
		Student participation
Citizenship Safety Project		
*Secondary school pupils trained to teach aspects of accident prevention and risk awareness*		
Injury prevention		
Delivered to: pupils (6–7 years) (primary)		
Delivered by: peers (14–15 years)		
Frederick, Barlow [51]	CBA	Perceptions of project (teacher/year 10 students)
	(pilot study)	
Eat Smart Play Smart		
*Behaviourally focused ‘lunchtime clubs’ of 25 min/week (for 20 weeks) to raise value of desired behaviour, tasting opportunities, incentives, and practical skills.*		
Obesity		
Delivered to: pupils (5–7 years) (primary)		
Delivered by: researchers		
Warren et al. [52]	RCT	Practical tasks/participation
		Incorporation of materials into curriculum
Family Smoking Education (Health Education Authority)		
*Teachers’ guide, parents’ leaflet, and pupils’ booklet were provided and delivered within the curriculum in the way that teachers judged to be most suitable.*		
Substance use (tobacco)		
Delivered to: pupils (11–13 years) (secondary)		
Delivered by: teachers		
Newman, Nutbeam [53]	CBA	Teachers’ feedback on programme delivery
GGHB Sexual Health Education		
*One lesson per week (5-week duration, as part of PSE curriculum), emphasising pupils’ ownership of the issues and challenging misconceptions with factual information. Mainly single sex groups. Included whole group, small group, and individual work and video, reading material, and quizzes.*		
SRE		
Delivered to: pupils (11–16 years) (secondary)		
Delivered by: teachers		
Lowden, Powney [54]	Process evaluation	Student engagement
		Teachers’ experiences
HeLP—Healthy Lifestyle Programme		
*Over three terms (18 months), a range of activities intended to change behaviour at individual, family, and institutional levels—competitions, workshops, parents’ evening, PSHE lessons, drama activities, 1:1 goal-setting and assessment, newsletters.*		
Obesity		
Delivered to: pupils (8–11 years) (primary)		
Delivered by: teachers, drama group, researchers		
Wyatt et al. [55]	CRCT (pilot)	Delivery
	Process evaluation	Resources
KAT—Kids, Adults Together		
*Classroom activities, ‘fun evening’ for families, DVD for pupils to watch with parents.*		
Substance use (alcohol)		
Delivered to: pupils (9–11 years) (primary)		
Delivered by: teachers		
Rothwell, Segrott [56]	Process evaluation	Engagement
NE Choices		
*Drama workshop, ‘youth work projects’, outdoor activity programme for ‘high-risk’ youth, and drugs awareness sessions for parents, training for teachers, youth workers, and school governors.*		
Substance use (legal and illegal drugs)		
Delivered to: pupils (13–16 years) (secondary)		
Delivered by: various (theatre company, teachers, youth workers)		
Stead et al. [57]	CBA	Project aims versus youth work aims
	Process evaluation	Concordance/adaptation to participants needs
PhunkyFoods Programme		
*Lesson plans (1-h duration) to support delivery through art, drama, music, and play activities. Schools can adapt lesson plans. DVDs, books, and games provided.*		
Healthy eating and physical activity		
Delivered to: pupils (5–11 years) (primary)		
Delivered by: school staff		
Teeman et al. [58]	BA	Teachers’ experiences
	Process evaluation	
Project Tomato		
*Manual and 12 curriculum-related lesson plans formed ‘core elements’; other elements were ‘customisable’—cooking lessons, growing club information, team set-up information. Support materials (kit bags, newsletters, parent handouts) provided.*		
Obesity		
Delivered to: pupils (8–9 years) (primary)		
Delivered by: teachers		
Christian [59]	CRCT	Fidelity
	Process evaluation	‘Appreciation’ of programme
RIPPLE—Randomised Intervention of Pupil Peer Led Sex Education		
*Peer educators trained by an external team to prepare classroom sessions (3 × 1 h), which were delivered without teacher supervision. Sessions used participatory learning methods and activities, covering relationships, STIs, and contraception.*		
SRE		
Delivered to: pupils (13–14 years) (secondary)		
Delivered by: peers (16–17 years)		
Forrest et al. [60]	Process evaluation	Participation in programme
		Student engagement
		Sex educator characteristics
Strange et al. [61]	Process evaluation	Engagement with the programme
		Perceived benefits beyond health promotion
Strange et al. [62]	Process evaluation	Engagement with the programme
Oakley et al. [63]	Process evaluation	Engagement with the programme
Stephenson et al. [64]	CRCT	Programme participation
	Process evaluation	Student satisfaction with programme
		Fidelity
Strange et al. [65]	CRCT	Student engagement
Schools on the Move		
*Teacher training covering embedding physical activity into the curriculum; distribution of pedometers to pupils, with instructions on how to use and record their activity levels on a website.*		
Physical activity		
Delivered to: pupils (4–11 years) (primary and secondary)		
Delivered by: teachers		
Stathi et al. [66]	BA	Programme participation
	Process evaluation	Schools ethos
		Resources
		Programme support
		Rewards
SHARE—Sexual Health and Relationships: Safe, Happy and Responsible		
*5-day teacher training, to deliver 20 classroom sessions (piloted and developed over 2 years). Sessions included small group work and games, information leaflets, and skill development through interactive video and role play.*		
SRE		
Delivered to: pupils (13–15 years) (secondary)		
Delivered by: teachers		
Wight et al. [67]	CRCT	School attitudes (PSE, context)
	Process evaluation	Teachers’ attitudes/teaching styles
	(preliminary baseline)	Teachers’ relations to pupils
		Teaching elements
Wight, Abraham [68]	Programme development	NA
Buston, Hart [69]	Process evaluation	Student attitudes
		Teacher confidence
Buston et al. [70]	Process evaluation	‘Fit’ with school organisation
		Teachers’ explanations and reflections
Buston et al. [71]	CRCT	Fidelity
	Process evaluation	
Buston et al. [72]	Process evaluation	Student engagement (discomfort, gender, teacher, trust, fun)
Buston, Wight [73]	Process evaluation	Young women
		Group discussions
		Timing, skill-based lessons
Wight et al. [74]	CRCT	Organisational factors (i.e. timetabling)
	Process evaluation	Skill-based delivery
Wight, Buston [75]	Process evaluation	Teacher training,
		Developing confidence
		Familiarisation of programme
		Collegiality
Buston, Wight [76]	Process evaluation	Student engagement (understanding variation between classes)
		Teachers’ explanations and reflections
Smoking and Me		
*1-day teacher training and teachers’ guide—five lesson outlines and guidance for choosing group leaders and managing groups. Lessons predominantly small group work—discussion, role play, and decision-making activities.*		
Substance use (tobacco)		
Delivered to: pupils (12–13 years) (secondary)		
Delivered by: teachers, peers		
Newman et al. [77]	CBA	Pupil engagement
		Fidelity
SPICED—Schools Partnership in Children’s Education on Drugs		
*2-day training for professionals delivering programme. Seven classroom sessions, using participative learning, role play, and group discussion—covering facts about legal and illegal drugs, develop understanding of risk, and promote confidence and decision-making skills.*		
Substance use (legal and illegal drugs)		
Delivered to: pupils (9–11 years) (primary)		
Delivered by: teacher, police officer, school nurse		
Crosswaite et al. [78]	Process evaluation	Classroom setting
		Training provided
		Issues around involvement of professional groups
UK Resilience Programme		
*Professionals delivering the programme attended 10-day training course in USA. Manualised intervention (18 × 1 h workshops) teaching cognitive behavioural and social problem-solving skills.*		
Well-being		
Delivered to: pupils (11–12 years) (secondary)		
Delivered by: trained facilitators		
Challen et al. [79]	CBA	Pupils’ and teachers’ experiences
Challen et al. [80]	Process evaluation	
Y-Active		
*Various physical activities—breakfast club, physical education, lunchtime fitness classes, after-school play club, after-school sports. A ‘small fee’ was charged for breakfast and after-school activities (opt-in only).*		
Physical activity and well-being		
Delivered to: pupils (9–11 years) (primary)		
Delivered by: YMCA sports coaches/play workers		
Stathi, Sebire [81]	Process evaluation	Project staff’s experiences

*BA* before and after study, *CBA* controlled before and after study, *CRCT* cluster randomised controlled trial, *NRCT* non-randomised controlled trial, *SRE* sex and relationship education, *SES* socio-economic status, *NC* non-comparative, *NR* not reported, *NA* not applicable

### Testing of programme theories

**Fig. 2:**
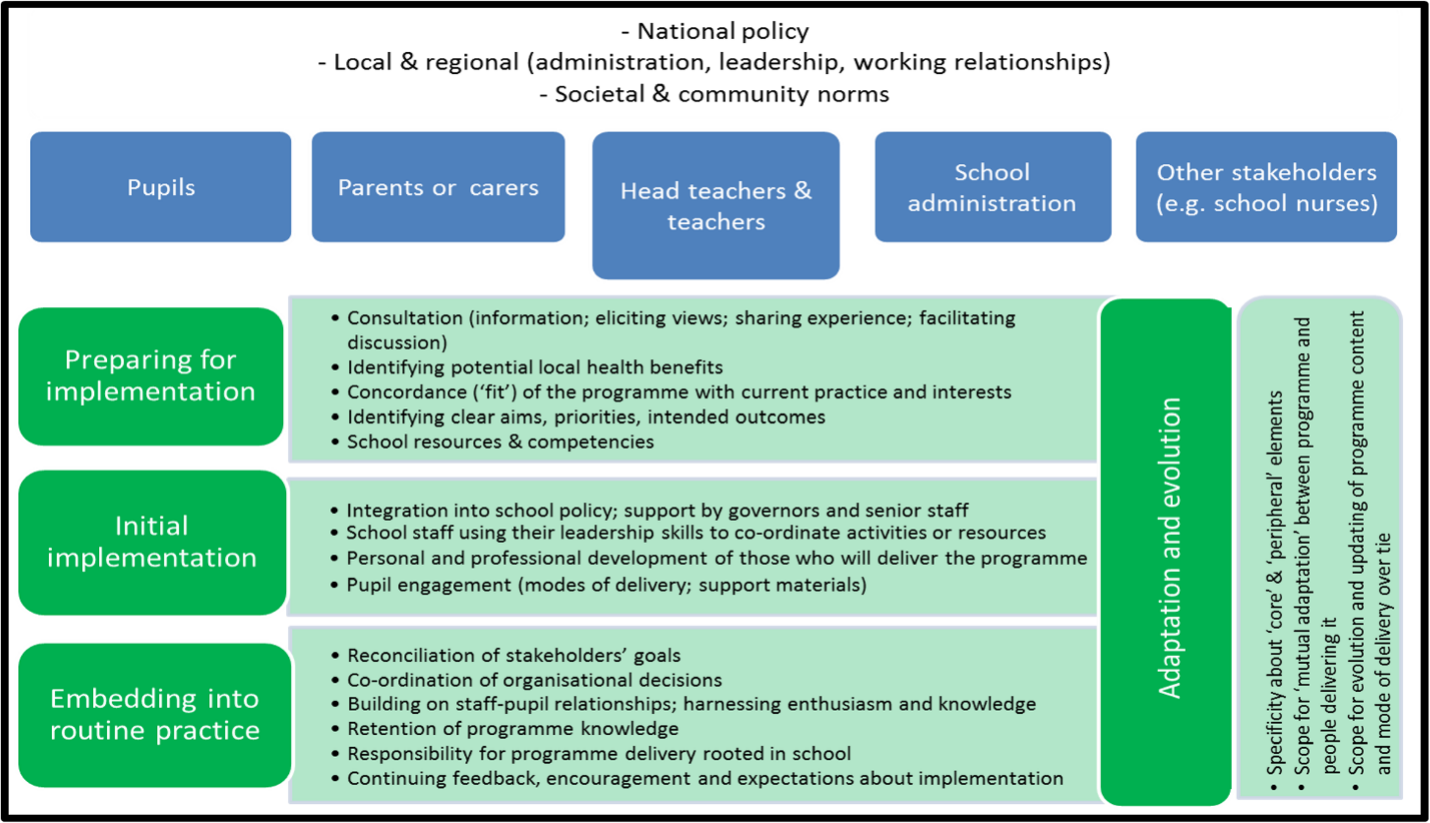
Conceptual framework for designing and implementing health promotion programmes in schools

To help guide our efforts in the extraction and synthesis of relevant evidence from included studies in the second (theory-testing) stage of the review, we summarised the programme theories in a conceptual framework (Fig. 2). Phase 2 of the review included evaluations of health promotion programmes delivered in UK primary or secondary schools that reported findings that enabled aspects of the four programme theories to be tested. Table 3 lists the details of the 41 included papers, reporting evaluations of 20 different health promotion programmes in schools—11 of which were delivered in primary schools and 9 in secondary schools. A brief summary of each programme is provided in the table, with full details reported in Additional file 6.

**Table 4:** Programme theories tested in the review

Theme	Context-mechanism-outcome	Area and sources	Full synth.^a^
**Programme theory 1—preparing for implementation**			
Preparation for the introduction of a health promotion programme to a school is more likely to be successful when systematically planned in conjunction with other school responsibilities. This involves			
Pre-delivery consultation	Well-established, uncontentious programmes are perceived as credible and ‘workable’ by teachers and require only basic pre-delivery consultation to be viewed as acceptable; *However* If a programme contains novel topic or delivery components, then staff’s unfamiliarity with these elements risks them being viewed as unacceptable. In these cases, more extensive pre-delivery consultation is required. *Also* If a programme’s topic or approach is contentious for some stakeholders, then consultation needs to be inclusive of these stakeholders.	Drug [50]; SRE [45] [54] [63] [70]; Tob [44] [45]	p.1–4; 8–11
Pupil engagement	Whatever the topic of a programme, pupils are engaged if the relevance and multiple benefits (personal, social, academic) of a programme are made clear. *Also* Novelty can be a strong way of initially engaging pupils’ attention, but novelty alone is insufficient for maintaining engagement.	Ob [58] [55]; SRE [54] [69] [72]; Tob [53] [77] [44] [45]; WB [79]	p.5–6
Reciprocity	For all programmes, successful preparation for delivery hinges on teachers’ and pupils’ judgement that they will receive the support they deem to be necessary to achieve multiple goals.	Ob [58]; PA [42] [66]; SRE [54] [70] [71] [75]; Tob [77] [44] [45] [47]; WB [79]	p.5–10
Current practice and interests	Programmes can ‘work with’ current practice and interests in a number of ways (e.g. by meeting an unmet need, by complementing, or by driving change), but the contexts in which these processes occur remains unclear.	Ob [58] [55]; PA [42]; SRE [68]; Tob [45]	p.9–10
Identifying clear aims and priorities, including intended outcomes		None identified	Not applicable
**Programme theory 2—introducing a programme within a school**			
The introduction of a health promotion programme to a school is more likely to be successful when it is incorporated into school activities through			
Integrating a programme into the life of a school (senior support)	Across a wide range of programmes, it is necessary for the actions of seniors to be *tangible* so that teachers feel confident in playing their role in programme delivery. *However* The extent of senior support is less important where pupil groups are more stable (e.g. in primary schools) and less contentious health issues are addressed.	Ob [43]; PA [42]; SRE [69] [63]; Tob [47]; WB [79]	p.12–13
Integrating a programme into the life of a school (leadership skills)	If a named co-ordinator is willing, able, and has the support and capacity to take responsibility, then programme delivery is improved. The success of this role depends on a person’s credibility and influence rather than his/her level of seniority.	Ob [58]; PA [42] [81]; SRE [62]; Tob [47]	p.13–14
Engaging those who deliver and participate in health promotion programmes (training and professional development)	If teachers perceive that training for a programme addresses relevant skill or knowledge deficits, then they are more likely to be motivated to engage with that training. *However* If a programme’s approach is discordant with teachers’ personal values, then engagement can be problematic.	Drug [57] [48]; Ob [58]; PA [42]; SRE [69] [61] [75]; Tob [46]; WB [79]	p.15–17
Engaging those who deliver and participate in health promotion programmes (pupils)	At both primary and secondary school levels, programme flexibility to accommodate pupils’ different rates of physical, psychological, and social development facilitates engagement. At primary school, pupils experiencing a programme as fun is the main way to engage pupils. At secondary school, pupils’ engagement pivots on a perception that a programme is both fun to take part in *and* addresses a perceived knowledge or skill deficit. *Also* Where a programme addresses controversial behaviours, teacher-pupil confidentiality is key to pupil engagement.	Alc [78] [48]; Drug [66]; Ob [58] [52] [55]; PA [62] [63]; SRE [54] [60] [72] [73]; Tob [56] [82]	p.17–22
**Programme theory 3—embedding a programme into routine practice**			
The routine delivery (‘embedding’) of a programme takes time and motivation. It is likely to involve changes in the school environment and the development of new relationships between stakeholders that require pro-active management so that			
Different stakeholders’ goals are reconciled		None identified	Not applicable
Organisational decisions in other areas of school life are made taking into account how they impact onprogramme delivery	No additional insight to that in programme theory 2 (‘integrating a programme’)	Tob [47]; WB [80]	p.22
School staff’s existing relationships with pupils are built upon		None identified	Not applicable
Stakeholders’ enthusiasm, knowledge, and experience are harnessed		None identified	Not applicable
Knowledge of ‘core’ and ‘peripheral’ elements and minimum resources, skills, and informational content is retained		None identified	Not applicable
Responsibility for programme delivery becomes rooted in the school	Insufficient evidence to express context-mechanism-outcome	PA [55]; WB [74]	p.23
Expectations about implementation are fed back	No additional insight to that in programme theory 2 (‘integrating a programme’)	Drug [78]; Ob [58] [52]; Tob [46]	p.23–24
**Programme theory 4—fidelity of implementation and programme adaptation**			
The preparation for, introduction, initial delivery, and ongoing sustainability of a health promotion programme in a school is more likely to be successful when there is			
Specificity about essential, optional, and adaptable programme elements	Insufficient evidence to express context-mechanism-outcome	Drug [57] [48] [49]; Ob [59]; [58]; SRE [54]; Tob [44] [47]	p.24–26
Scope for ‘mutual adaptation’ between the programme and the people delivering it	Insufficient evidence to express context-mechanism-outcome	Drug [48]; Ob [58]; PA/WB [81]; SRE [71]; Tob [77]; WB [80]	p.24–26

Key (type of health promotion programme): *Alc* alcohol, *Drug* legal and illegal drugs, *Ob* obesity, *PA* physical activity, *SRE* sex and relationship education, *Tob* tobacco, *WB* well-being

^a^For full synthesis, see Additional file 11

#### Critical appraisal

All included studies were critically appraised using the Wallace et al. [35] tool for assessing quantitative, qualitative, and mixed-methods studies. This enabled the strengths and weaknesses of different aspects of each study to be identified, rather than a summary verdict on the quality of the whole study. A summary of the key points of the critical appraisal was included in each data extraction table and collated in a summary critical appraisal table (see Additional file 7).

#### Data extraction

Information on study type, the programme being evaluated, the content and delivery of the programme, and research methods (sample, participants, data collection and analysis) and evidence to enable testing of each of the four programme theories were extracted to data extraction tables (Additional file 8). To facilitate synthesis, where evaluation of a health promotion programme was reported across multiple publications, all data was extracted to a single table.

As evidence to test the programme theories was rarely reported in a consistent format or section within papers, we used contents pages, the executive summary, sub-headings, and/or the conclusions (as appropriate to the publication type) in order to ‘gain a foothold’ and start the process of reading and data extraction. This was an iterative process which involved our critical consideration of the extent to which studies’ findings enabled the programme theories to be tested. Our decision-making was guided by looking for ‘markers of implementation’ (for example, stakeholders’ experiences, perceptions, and competencies—see Additional file 9), although we did not limit extracted data to only these ‘markers’ if we judged other evidence to be relevant. For example, relevant evidence could be indirect, such as the effect of homophobic attitudes (on the part of both teachers and pupils) on levels of engagement in sex and relationship education (SRE).

The volume of reported information in some studies and our desire to not lose the potential contribution of authors’ analyses to the synthesis meant that we judged when it was necessary to either summarise data or extract authors’ analyses. Here we used the distinction made in meta-ethnography between first- and second-order interpretations [36]. In recording data, we used double quotes where study participants’ views, experiences, or understandings were reported in their own words (first-order interpretation) and single quotes where study authors’ analyses were extracted (second-order interpretations). As our aim was to identify and extract key pieces of information, we recorded additional contextual information or critical appraisal findings immediately adjacent to the relevant extracted data. This guarded against our synthesis being conducted without knowledge of these factors, which might relate to a particular piece of evidence but not the study as a whole. To attain consistency, each critical appraisal and data extraction was checked by the lead reviewer (MP), with feedback or revisions provided as appropriate.

#### Synthesis

Consistent with a realist approach to the explanation of complex phenomena, where relationships between phenomena may be multi-factorial, inter-dependent, and emergent, we treated the ‘ways of synthesising’ as principles to critically apply rather than strict instructions to use on each piece of evidence. The iterative and explanatory nature of synthesis in a realist review meant that the processes of juxtaposition, reconciliation, consolidation, situation, and adjudication of different sources and evidence [18] (see Table 1) were used in combination rather than separately. Whilst we had conducted critical appraisal before the synthesis (rather than concurrently, as advocated by Pawson [37, 38], in adjudicating between different sources, we were careful to use the findings of the critical appraisal in relation to the relevant aspects of or insights from a study rather than judging the validity of each study as a whole. If our initial critical appraisal was unable to support a judgement about a particular piece of evidence, we returned to the original source so that a bespoke appraisal incorporating rigour and relevance could be made. We believe that this is a more transparent process for incorporating rigour and relevance in the conduct of a realist synthesis than solely appraising studies during synthesis.

Throughout the synthesis, we bore in mind the implications of emerging explanations for testing each of the programme theories. Details of the practical stages of the synthesis can be found in Additional file 10.

## Findings

We present our findings as a summary of contextualised mechanisms relating to each of the issues encountered in each programme theory, noting the depth, breadth, and overall rigour of the underlying evidence. The summaries are intended to facilitate decision-makers’ and practitioners’ *sensemaking* of local contexts, thereby facilitating self-organisation at the local level [39].

To enhance the readability of the summaries within a limited space, citations to the evidence underpinning each programme theory are contained in Table 4 (rather than in the text) together with a summary of context-mechanism-outcome configurations. Additional file 11 contains a longer version of the findings (see Table 4 for page numbers relating to each context-mechanism-outcome configuration), with examples and greater detail about the rigour of individual pieces of evidence used in the synthesis.

### Programme theory 1: preparing for implementation

#### Pre-delivery consultation

Whilst the rigour of the underlying evidence is highly variable, both the type of health promotion programme *and* the recent school history of delivering programmes on the topic are likely to impact on the extent and depth of pre-delivery consultation needed. A more ‘mature’ and uncontentious area of health promotion such as physical activity, where there is a history of delivering similar programmes and existing staff and organisational networks provide a foundation to support programme delivery, is likely to require substantive but brief ‘pre-delivery’ consultation with school staff and parents. Where aspects of health promotion are less well-established, such as social and emotional issues in SRE, and where the topic may be a highly charged personal issue for teachers (for example, in terms of morality and sexual identity), more extensive ‘pre-delivery’ consultation with school staff and parents is likely to be necessary. Areas of health promotion such as healthy eating and smoking prevention, whilst relatively uncontentious, may still require significant pre-delivery consultation, especially where a programme contains novel components of delivery or content with which school staff are unfamiliar.

#### Pupil engagement

Making a health promotion programme appealing to pupils is not necessarily straightforward. Programmes need to be developmentally appropriate and address issues perceived as relevant by pupils, whilst at the same time stretching pupils’ understanding of health issues that may lie well outside of their experience or understanding. ‘Sweeteners’ can play an important role—pupils are strategic thinkers themselves and may well respond to the ‘multiple pay-offs’ that a programme can offer such as the development of transferable educational or life skills. None of these more complex considerations should pressurise programme designers and school staff into overlooking the potential of a simple ‘hook’, such as the novelty of an external provider, for engaging pupils’ attention.

#### Reciprocity

Preparing for the delivery of a health promotion programme in a school revolves around reciprocity. On the whole, teachers will devote their time and energy if they believe that they will get the practical and educational support to enable them to play their role. Even if this reciprocity is more symbolic than practical at the initial stages, it can start a process of engagement that fosters co-operation towards achieving a common goal, such as improved health outcomes for pupils through delivery of a health promotion programme.

Reciprocity is also important for pupils. Long-term health gains are mostly an abstract concept for pupils of both primary and secondary school age, so they need to perceive other, more short-term (and non-health) gains from participating in a health promotion programme. Amongst other things, this can be related simply to enjoyment (having some fun), identity development (e.g. status amongst peers), or mid-term goals (e.g. developing transferable skills).

Both teachers and secondary-school-age pupils try to ensure that actions contribute to more than one beneficial outcome. This does not override the contribution of intrinsic motivation, such as teachers’ desire to play a pastoral role in child development or pupils’ appreciation of knowledge about a healthy lifestyle. However, it does highlight that the delivery of a health promotion programme in a school has to take place within current frameworks that demand outcomes on many levels. Teachers will want to balance pupils’ educational goals and psycho-social development with the demands of the local and school political environment, their personal work/life balance, and career development. Pupils will want to achieve their educational goals and develop psycho-socially (albeit this may simply be perceived as ‘growing up’). The extent to which the preparation for delivery of a programme considers how these diverse goals can be accomplished is central to successful implementation.

#### Negotiation (about SRE programme delivery)

There is evidence from a well-conducted process evaluation of a SRE programme (the SHARE programme—Sexual Health and Relationships: Safe, Happy and Responsible) that negotiation about, and adaptation of, health promotion programmes in schools takes place in a wider context than ‘health’. At the school level, decisions about programme content and delivery are political in the sense that they aim to balance the views and demands of a broad range of stakeholders. The extent to which this applies in areas of health promotion other than SRE is unclear.

#### Concordance of the programme with current practice and interests

There is weaker, observational evidence from a range of health promotion programmes that concordance between a programme and current school activities and priorities works in a number of different ways:

- Meeting an unmet need in a school in a way that is consistent with other school activities (i.e. ‘meshing’)
- ‘Working with’ and therefore contributing to the development of a particular school ethos (i.e. ‘complementing’)
- Co-ordinating other school activities to fit with programme components (i.e. ‘driving’)

Sometimes, even where there appears to be a lack of concordance between a programme and some school activities, this can act as a stimulus for change and mutual accommodation. However, this may require early recognition and careful negotiation.

### Programme theory 2: introducing a programme within a school

#### Integrating a programme into the life of a school

Active support by senior figures within a school is necessary but has to extend deeper than written policies—the ways in which policies will be put into action ‘on the ground’ need to be specific and clear. This is because the organisation and delivery of health promotion programmes can be experienced by teachers as an additional responsibility, and one which they are unlikely to want to ‘go the extra mile for’ if doing so is perceived as risky for their professional life, personal well-being, or work-life balance. The pathway of programme introduction and delivery needs to be both paved (practical assistance—specific training, resources, and co-ordination with other aspects of school life) and sheltered (from local or national outside parties who disagree with a programme’s focus or approach).

The importance of this ‘on the ground’ support broadly follows a continuum, with support being less pivotal at the primary school level where a teacher’s class usually consists of the same group of pupils and less contentious health promotion topics are addressed. At the secondary school level, where pupils’ subject options can lead to more change in class composition, there may be pronounced differences in levels of maturity, and as more contentious health promotion topics are addressed, this support becomes more important. The need for and specific type of support and training will also critically depend on whether the people delivering the programme are teachers or other professionals working within a school. For example, teachers may need skills and confidence in specific behaviour change techniques that are part of the programme, whereas outside professionals delivering the programme may need skills and confidence in classroom management.

Whether programmes are delivered by teachers, external professionals, peer educators, or a mixture of these, in both primary and secondary schools, it was consistently reported that a named co-ordinator was important for initiating and sustaining programme delivery. The profession or status of this person, and whether or not he/she was a school employee, was far less important than his/her willingness to co-ordinate, his/her skills and capacity to do so, and his/her ability to exert influence within the school.

#### Engaging those who deliver and participate in health promotion programmes

Across both primary and secondary school levels and a range of health promotion topics, the motivation of those delivering programmes to engage in training depended on whether or not the training addressed knowledge or skill deficits that were relevant from their point of view. This links with reciprocity (see programme theory 1 summary)—both teachers and pupils are more likely to engage when they can see the likely personal, social, and/or developmental gains from participating. Engagement can be problematic where there is discordance between health promotion topics and personal values, although this is only reported in relation to SRE.

The engagement of pupils as participants broadly follows a continuum in line with psycho-social development. At the primary school age, the key issue is whether or not a programme is fun. As pupils progress through the secondary school years and health promotion addresses more contentious issues such as sexual relationships and substance use, fun remains necessary but is not sufficient. Addressing a perceived skill or knowledge deficit and the quality of the relationship between participants and those delivering the programme assume a greater importance. Participants’ confidence in the maintenance of confidentiality can be highly important for engagement in topics such as SRE and substance use.

In both primary and secondary schools and for a range of health promotion topics and programme types, engagement was facilitated by programmes being sufficiently flexible to allow tailoring to different levels of pupils’ physical, psychological, and social development and different levels of skill and experience (both of pupils and those who are delivering a programme).

### Programme theory 3: embedding a programme into routine practice

The research timeframes of included studies were mostly too short (2 years or less) to produce evidence about the embedding of health promotion programmes. There is limited evidence in the short term about the impact of co-ordination of programmes with other school activities, but this does not add substantively to that identified in the programme theory 2 summary. Other evidence about embedding is limited to the views of teachers and managers about aspects they *think* would help, such as senior support and networking. However, the fact that teachers and managers had to venture ideas about how to embed programmes strongly suggests that considerations of sustainability were simply not part of any of the design of programmes.

### Programme theory 4: fidelity of implementation and programme adaptation

There was substantial variation across all programmes in how they were delivered in different schools, but in the included studies, it was not possible to distinguish ‘warranted variation’ (for example, based on informed professional judgement) from ‘unwarranted variation’ driven by other factors. The usefulness and acceptability of programmes where core and customisable elements were specified was not evaluated, although there was considerable ambivalence expressed by teachers about the usefulness of more prescriptive programmes. An evaluation of a SRE programme suggests that fidelity of implementation is enabled when teachers work within a collegial atmosphere where issues about programme delivery can be openly discussed with colleagues and support obtained from senior staff in the school and the programme’s developers.

## Discussion

This is the first review of the implementation of health promotion programmes in schools to have been conducted using a recognised review method. The use of realist review was novel in this field, and through its application, we have been able to improve understanding of transferable mechanisms rather than simply identifying de-contextualised implementation processes. Our review has consolidated and refined existing conceptual frameworks and used evaluations in UK schools of a range of health promotion topic areas to specify key context-mechanism-outcome configurations. These configurations are presented in a narrative designed to facilitate decision-makers’ and practitioners’ use of the findings in conjunction with knowledge of their local contexts. In this way, we have extended the work of Greenberg et al. [32] and Samdal and Rowling [5] by moving beyond statements about the principles of good implementation practice and towards a more refined understanding of the complexity of implementation within educational, public health, and social systems that are constrained in multiple, setting-specific ways.

Our findings have identified key transferable mechanisms (e.g. reciprocity) that impact on implementation and which apply to both teachers and pupils. We have also been able to specify how an accepted principle of implementation, such as congruence between existing school activities and proposed health promotion activities, can operate differently (but beneficially) according to context—for example, by meeting unmet needs, complementing existing activities, or paradoxically by stimulating change so that congruence is achieved. Our findings have also identified where some of the mechanisms that underpin implementation differ in how they operate between primary and secondary schools and between health promotion topics. By exploring context-mechanism-outcome configurations, we have also been able to go beyond generic ‘one recommendation fits all’ statements. For example, we have been able to specify the actions that senior school figures should take in order to provide support for the implementation of a health promotion programme.

Whilst our synthesis provides greater specificity in relation to *preparing for implementation* and *initial implementation* of health promotion programmes in schools, the amount, depth, and rigour of evidence about the later stage of *embedding into routine practice* and the cross-cutting theme of *adaptation and evolution* (Fig. 2) were limited. This meant that we were unable to explore important areas identified in our programme theory relating to embedding into routine practice. For example, we were unable to locate evidence about how different stakeholders’ goals are reconciled; how stakeholders’ enthusiasm, knowledge, and experience are harnessed; and how knowledge about core and customisable elements of programmes are retained in the longer term. An explanation for this is that the timeframe over which evaluations are funded simply do not extend sufficiently far to investigate these factors. Nevertheless, some of the key mechanisms and contexts identified as relevant to the initial implementation of programmes may also be important for longer term embedding or ‘scaling up’ from school to school.

Regarding adaptation and evolution, the planned content and delivery of a number of the programmes evaluated were under-specified, meaning that assessment of fidelity and judging the extent to which (or justification for) adaptation of programmes took place was highly problematic. Tailoring of programmes to meet the needs of participants, both those who deliver a programme (for example, addressing specific skill deficits) and the pupils who are its intended beneficiaries (for example, tailoring to different levels of physical, psychological, and social development), whilst preserving the essential functional components of the programme, is a central challenge which we have not been able to address using the evidence located for this review.

Our findings provide a platform for future evaluations of implementation processes in schools. For example, whilst we have identified the impact of a wide range of stakeholders about the content and delivery of a SRE programme, we were unable to locate evidence about this for other types of health promotion programmes. SRE programmes may represent a distinct, and in some communities particularly contentious, area of health promotion. However, in the absence of research about how stakeholders impact on the implementation of other types of health promotion programmes, we do not know if the mechanisms in operation are the same or different.

Whilst we could, as the review’s authors, speculate about more specific recommendations for public health programme designers, promoters, deliverers, and evaluators, we believe we are not best placed to do this—especially as what may be a salient and useful insight for one group of potential research users (such as head teachers or school governors) may be seen as irrelevant or obvious to another group (such as programme developers and promoters). For this reason, we have undertaken another consultation exercise with representatives of these different groups, which will lead to tailored evidence summaries and recommendations for these different key groups, including commissioners and funders of public health programmes.

### Strengths and limitations

This realist review has drawn on a range of types of evidence about implementing health promotion programmes in schools to produce new insights that are relevant to a range of decision-makers. Using a realist approach has provided a consistent logic of inquiry for synthesising evidence across different types of programmes. Through our provision of extensive review process documentation, we hope to enable other reviewers to judiciously apply a realist approach, as well as provide substantive material for critiques that will foster methodological development.

Whilst we have been able to identify context-mechanism-outcome configurations (such as reciprocity) in depth for certain aspects of implementation, this was not the case for all aspects. These differences reflect the extent and depth of the underlying evidence but also, quite simply, the difficulty of identifying ‘hidden’ mechanisms. For those embarking on realist reviews now, it is worth contemplating that we could have widened our searches to other fields, included evaluations from outside of the UK, and/or drawn more closely on our review advisory group’s knowledge to help identify context-mechanism-outcome configurations in these more ‘difficult’ areas.

The notable lack of evidence about what determines the longer term sustainability of programmes (i.e. their embedding in schools year on year), or their ‘spread’ from school to school, may require comparative primary research, for example, comparing effective programmes which have become widespread within some countries (like the ASSIST programme in Wales and England; see University of Bristol’s REF 2014 impact statement [40] for a description of the impressive uptake of this programme) with others which, whilst found to be effective, never became widely adopted. This may reveal further how different schools or different school systems either complement or conflict with the practicalities of delivering particular programmes. Such research may also reveal whether longer term implementation may rely on stable organisational and budgetary boundaries, together with more compelling evidence of cost-effectiveness—or other factors which would probably not be revealed by initial evaluations in relatively few schools, when subsidised by evaluation funding and often also energised by the original developers of the programme.

## Conclusions

Through applying a realist approach, we have been able to identify mechanisms in action that affect the successful implementation of health promotion programmes in schools. At the preparatory stage, implementation hinges on negotiation about programme delivery and the acceptability (or otherwise) of the programme to those who will deliver it. Addressing fears about programme novelty, contentious subject matter, and the extent of support for delivery are likely to be important. At the initial implementation stage, programme delivery needs to be both facilitated within a school and protected from external forces. This becomes more important where the composition of groups of pupils is more complex and where more contentious health issues are addressed. The available evidence was insufficient for us to confidently identify mechanisms about the process of embedding programmes into practice or the circumstances where the adaptation and evolution of programmes is necessary for them to be feasible and sustainable.

Our inclusion of a diversity of sources of information and integration of a review advisory group’s input throughout the review have enabled us to produce findings that are both academically rigorous and applicable to decision-making at a range of local and strategic levels. Further research should focus more on investigating and refining the identified mechanisms (both in trials of interventions and evaluations of local practice), the dynamic nature of programme adaptation during implementation, and programme sustainability.

## Availability of supporting data

The data sets supporting the results of this article are included within the article and its additional files.

## Additional files

Additional file 1: **Search strategy.** The file is a record of (and reasons for) the sources obtained. (DOCX 20 kb)
Additional file 2: **Contribution of conceptually rich studies.** A total of 22 sources informed the development of the conceptual framework and theory-development review phase. (DOCX 30 kb)
Additional file 3: **Long list of programme theories.** The file contains a list of 12 programme theories that encompassed all of the concepts around implementation identified in the 22 conceptually rich sources and to identify the unique contributions of each source. (DOCX 17 kb)
Additional file 4: **review advisory group.** The RAG was formed to contribute to the identification, selection, and refinement of programme theories to be tested in the review. (DOCX 24 kb)
Additional file 5: **Contribution of sources to development of programme theories.** The file is a documentation of the three stages of programme theory development, showing the areas in which different sources contributed. (DOCX 23 kb)
Additional file 6: **Programme descriptions.** The file reports the full details of each programme. (DOCX 26 kb)
Additional file 7: **Critical appraisal summary.** A summary of the key points of the critical appraisal collated in a summary critical appraisal table. (DOCX 21 kb)
Additional file 8: **Data extraction tables.** The file lists information on study type, the programme being evaluated, the content and delivery of the programme, and research methods (sample, participants, data collection and analysis) and evidence to enable testing of each of the four programme theories. (DOCX 24 kb)
Additional file 9: **Markers of implementation.** Our decision-making was guided by looking for ‘markers of implementation’, although we did not limit extracted data to only these ‘markers’ if we judged other evidence to be relevant. (DOCX 19 kb)
Additional file 10: **Practical stages of the synthesis.** The file lists the details of the practical stages of the synthesis. (DOCX 17 kb)
Additional file 11: **Long version of synthesis.** The file contains a long version of the findings, with examples and greater detail about the rigour of individual pieces of evidence used in the synthesis. (DOCX 84 kb)
