# Supplementary material for: Implementing health promotion programmes in schools: a realist systematic review of research and experience in the United Kingdom
Source: Implement Sci. 2015 Oct 28;10:149. doi: 10.1186/s13012-015-0338-6 (PMC4625879; doi:10.1186/s13012-015-0338-6)
Supplement: Additional file 1: — Search strategy. The file is a record of (and reasons for) the sources obtained. (DOCX 20 kb) [file 13012_2015_338_MOESM1_ESM.docx]

**Sensitising search**

A search was undertaken in Medline and Medline in Process. Keyword and MeSH terms were combined in the following format: ((school terms AND child terms) AND (generic intervention terms e.g. health promotion OR problem terms e.g. obesity) AND (implementation terms e.g. feasibility, sustainability, acceptability)). The search was limited to English language as the review aimed to generate an understanding of the implementation of health promotion interventions in schools in contemporary UK settings. In order to retrieve a diverse range of evidence to inform both parts of the review no study filters were applied. The full search strategy was:

Database: Ovid MEDLINE(R) In-Process & Other Non-Indexed Citations and Ovid MEDLINE(R) <1946 to Present>

Search Strategy:

--------------------------------------------------------------------------------

1 (child* or young adult or young people or pupil* or student* or adolescen* or teenage* or girl* or boy* or youth*).ti,ab.

2 Child/

3 Adolescent/

4 1 or 2 or 3

5 (school* or junior high or teacher* or teaching assistant or educational setting*).ti,ab.

6 exp Schools/

7 5 or 6

8 4 and 7

9 (health promotion or illness prevention or health inequalit* or intervention or multi-component or multi-modal or life skills or social skills or social competence or social influence or social-cognitive or refusal skills or resistance skills or resistance training or shame reduction or harm* minimi?ation or harm* reduction or anger management or anger control or empathy training or cognitive training or affective imagery training or role rehearsal or psycho-educational or behavio?r change or peer-led or teacher-led or partnership-based or internet based or psycho-social or multi-component or self-control).ti,ab.

10 Health Promotion/

11 (mental health or wellbeing or self-esteem or self-confidence or assertiveness or risk taking or victimi?ation or obesity or weight loss or overweight or waist circumference or BMI or healthy eating or nutrition or diet or school meal* or school dinner* or physical* activ* or exercise or fitness or physical education or sedentary or smok* or tobacco or cigarette* or substance misuse or substance abuse or street drug or illegal drug* or illicit drug* or cannabis or marijuana or inhalant* or alcohol or binge drink* or bully* or bullied or bullies or violen* or aggress* or delinquen* or classroom behavio?r or accident prevention or injury prevention or dog bite* or contracepti* or pregnan* or sexual activity or sexually transmitted disease* or sexually transmitted infection or dating violence or risk taking or sexual abuse).ti,ab.

12 *Mental health/ or *Obesity/pc or *weight loss/ or *Overweight/pc or *body mass index/ or *exp exercise/ or *exp Sports/ or *physical fitness/ or exp *Smoking/pc or *Substance-Related Disorders/pc or *exp street drugs/ or *Alcohol Drinking/pc or juvenile delinquency/ or *Violence/pc or *Accident Prevention/ or *Contraception/ or *Pregnancy in Adolescence/pc or *Sexual Behavior/ or *Sexually Transmitted Diseases/pc or *child abuse/pc

13 9 or 10 or 11 or 12

14 (implementation or feasibility or sustainability or acceptability).ti,ab.

15 8 and 13 and 14

16 limit 15 to english language

**Database searches**

After the results (2324 hits) from the above search were examined for additional implementation terms and synonyms and key sources, the search strategy was translated to a number of other data sources. A wide range of databases were searched (see table below). A broad range of sources was searched to reflect the multidisciplinary nature of the review, covering health, social science and educational evidence.

Table 1 Databases searched

| Medline and Medline in Process via Ovid |
| --- |
| CINAHL via EBSCO |
| British Nursing Index via Ovid |
| British Education Index via ProQuest |
| Cochrane Library, including DARE, CENTRAL, HTA and CDSR via Wiley |
| ERIC via ProQuest |
| Australian education index via ProQuest |
| ASSIA via Proquest |
| Sociological Abstracts via Proquest |
| Social Policy and Practice via Ovid |
| PsycINFO via Ovid |
| HMIC via Ovid |
| Science Citation Index via Web of Knowledge (Thomson ISI) |
| Social Science Citation Index via Web of Knowledge (Thomson ISI) |
| EPPI Centre Databases – Bibliomap, DoPHER, TRoPHI, The database on Obesity and Sedentary behaviour studies, Schemes to promote healthy weight among obese and overweight children in England, Database of Education Research, Current Educational Research in the United Kingdom (CERUK) |

**Supplementary searches**

In addition to bibliographic database searches web searching was undertaken to identify evidence not indexed in bibliographic databases, such as ongoing programmes from the UK or unpublished evaluations of health promotion in UK schools. The following sources were searched:

- Department of Health ([www.dh.gov.uk/](http://www.dh.gov.uk/)) - using the keywords “health” AND “schools” and a search within these results for "policy" (July 2012).
- Department of Education ([www.dfe.gov.uk/](http://www.dfe.gov.uk/)) publication section - scanned for publications related to health promotion in UK schools (July 2012).
- National Institute for Health Research Public Health Research Programme website completed and ongoing evaluations section [(www.phr.nihr.ac.uk/funded_projects/index.asp](http://(www.phr.nihr.ac.uk/funded_projects/index.asp)) (October 2012)
- National Institute for Health Research Health Technology Assessment Programme website ‘research in progress’ section ([www.hta.ac.uk/project](http://www.hta.ac.uk/project)) (October 2012)
