# Supplementary material for: Implementing health promotion programmes in schools: a realist systematic review of research and experience in the United Kingdom
Source: Implement Sci. 2015 Oct 28;10:149. doi: 10.1186/s13012-015-0338-6 (PMC4625879; doi:10.1186/s13012-015-0338-6)
Supplement: Additional file 2: — Contribution of conceptually rich studies. A total of 22 sources informed the development of the conceptual framework and theory-development review phase. (DOCX 30 kb) [file 13012_2015_338_MOESM2_ESM.docx]

| **Author** | **Conceptual framework** | **Area(s) of health prom.** | **Implementation aspects already present in Samdal & Rowling’s (**[**2011**](#_ENREF_23)**) PTs** | **Implementation aspects that add to/refine Samdal & Rowling’s (**[**2011**](#_ENREF_23)**) PTs** |
| --- | --- | --- | --- | --- |
| [Adelman and Taylor (1997](#_ENREF_1))* | ‘Scale-up model for replicating new approaches to schooling’ | Generic | Local policy commitment to a programme.  Allocation of adequate resources (including staff time) to enable implementation.  Phases included pre-implementation, implementation, and institutionalisation (‘sustainability’).  Capacity building of stakeholders. | The ‘match’ between an organisation (and the people within it) and the implementation process of a programme. This ‘match’ (or ‘fit’ or ‘readiness’) may need to be actively cultivated.  “A critical mass of stakeholders must consistently move in the direction of desired outcomes” (p.200) - barriers to working relationships therefore need to be addressed on an ongoing basis. This also highlights the need to harness the energy and enthusiasm of stakeholders.  Identifying the core and adaptable aspects of a programme. |
| [Arbeit et al. (1991](#_ENREF_2)) | Social learning theory (Bandura) | Cardio-vascular | Formation of a bespoke committee for implementation, with representatives from all stakeholder groups, echoes Samdal & Rowling’s ‘planning and preparation’ phase. | None |
| [Basch (1984](#_ENREF_3)) | Various (review) | Generic | Engagement with the wider community (e.g. parents).  Availability of, and ability to co-ordinate, finances to resource the programme. | Local adaptation of programmes may be important for successful implementation.  Consistency of the programme with school’s strategy. |
| [Bisset et al. (2009](#_ENREF_4)) | Actor Network Theory | Diet | Engagement with students and teachers and encouragement of their active participation.  Alignment of programme with school strategies and/or broader (regional/national) policies. | Achieving programme goals is recognised by non-education professionals (nutritionists) to inherently require active responses that adapt the programme so as to accommodate the goals of education stakeholders and student and teacher participants.  ‘Consultation’ is about more than seeking stakeholders’ views – it’s an active process of management of the ‘socio-technical networks’ that arise from the introduction of a programme, and in particular the reconciliation of education and health actors’ goals. |
| [Choi (2003](#_ENREF_5)) | Multiple – with focus on “multilevel processes involving human behaviour in social settings” | Generic | The context in which a programme is implemented is experienced both individually and refracted through group processes. | None |
| [Corboy and McDonald (2007](#_ENREF_6)) | Applies the conceptual model of implementation in schools (Greenberg et al.([Greenberg et al., 2005](#_ENREF_11))) | Mental health | Participant responsiveness – both for pupils (their interest in the programme) and Principals (consistency of the programme with other school aims and activities).  Programme support and training.  Engagement and consultation with teachers from an early stage. | Enablers (all directly drawn from [Greenberg et al. (2005](#_ENREF_11)):  - specificity in, and communication of, the programme model.  - user friendly, visually appealing, age appropriate and culturally sensitive support materials  - readiness of teachers and facilitators to deliver the programme (perceptions, skills, knowledge, beliefs).  - local or district-level context, e.g. stability of administration and leadership, and quality of working relationship with schools. |
| [Dusenbury et al. (2003](#_ENREF_7)) | Fidelity:  - adherence  - dose  - quality of programme delivery  - participant responsiveness  - programme differentiation | Mental health  Pro-social behaviour  Substance misuse | Teacher understanding of programme (through specific training) enabled implementation. | Barriers to implementation:  - programme complexity (inc. co-ordination between many people)  - need for ‘special skills’ in delivery  - re: adapting programmes (i.e. ‘process of accommodation’ between programme and organisation) - lack of knowledge about which programme elements are ‘core’ (not adaptable) and ‘less core’ (adaptable) |
| [Dusenbury et al. (2005](#_ENREF_8)) | See Dusenbury et al.([Dusenbury et al., 2003](#_ENREF_7)) | Substance misuse | See Dusenbury et al.([Dusenbury et al., 2003](#_ENREF_7)) | Less-experienced teachers likely to require greater instruction in programme delivery; more-experienced teachers were observed (despite the ‘minimal’ training given) to implement the programme “more completely and with better quality”, even though they were also critical of the programme. |
| [Gabrielsen (1993](#_ENREF_9)) | Review and critique of concepts of action in health campaigns – no specific framework proposed | Generic | Critique of conceptualisations in health campaigns as a whole, not in relation to implementation in schools. | None |
| [Graczyk (2000](#_ENREF_10)) | Unclear with regard to implementation | Social and emotional learning | Supervision and coaching of school personnel in programme delivery. | Potential for adaptation of the programme to the context of the school. |
| [Greenberg et al. (2005](#_ENREF_11))* | Conceptual model of school-based implementation | Generic | Capacity of school to deliver the programme.  Engagement of teachers and students.  Provision of appropriate training in programme delivery. | ‘Fit’ of programme with current practice within a school.  Stakeholders’ previous experiences of health promotion programmes - may be perceived as a ‘passing fashion’ rather than a worthwhile exercise.  Other aspects are identified, such as materials and training, but are intended as a measurement system rather than being expressed conceptually. |
| [Harachi et al. (1999](#_ENREF_12)) | None stated | Substance misuse | Staff development to enable teachers to deliver the programme in the way intended. | None |
| [Hoagwood and Johnson (2003](#_ENREF_13)) | Various implicit frameworks in the reviewed sources | Mental health | Organisational climate (as perceived by individuals) and culture (group processes within a ‘unit’, although this is only partially accounted for in Samdal & Rowling). | Organisational culture includes:  - normative expectations about behaviour and values  - power structure, both within schools and more widely (e.g. within a district) – for example, flexibility, discretion, hierarchy of authority, division of labour)  ‘Fit’ between the programme’s characteristics and objectives and those of teachers, students and the organisation |
| [Jensen and Simovska (2005](#_ENREF_15)) | None stated | Generic | Student participation | None |
| [Jowers (2007](#_ENREF_16)) | Applies Adelman & Taylor’s ([1997](#_ENREF_1)) dissemination model | Substance misuse | ‘Initial implementation’ – providing staff with support and guidance.  ‘Institutionalisation’ – maintaining change by incorporating a programme into the school’s structure. | ‘Creating readiness’:  - gaining stakeholder support (e.g. through provision of information)  - changing the school environment – may be fundamental issues in a school’s culture, e.g. allocation of time, staff, resources, materials  ‘Ongoing evolution and renewal’ – continuous programme development and integration of new knowledge into its delivery |
| [Kalafat et al. (2007](#_ENREF_17)) | Unclear | Pro-social behaviour | Programme co-ordinator’s characteristics and competencies.  School Principal support for the programme.  Nature of the relationship between the centre implementing the programme and the school, community and families. | None |
| [Kealey et al. (2000](#_ENREF_18)) | Behaviour change, as applied to teacher training | Smoking | Senior strategic support (e.g. at district-level) facilitates implementation considerably.  Readiness of senior staff and teachers.  Ways to support the sustainability of programmes. | Teacher engagement is attained not only by communicating information about the programme, and providing training and teaching materials, but by motivating teachers and harnessing their enthusiasm, knowledge and experience.  Scope for mutual adaptation between the programme and people delivering it. |
| [Moore (2011](#_ENREF_19)) | Socio-ecological framework | Diet | Pupil’s engagement with the goals of the programme.  Understanding of programme goals and effect on how the programme is delivered (especially of non-educational staff). | None |
| [Osganian et al. (2003](#_ENREF_20)) | Rogers’ diffusion theory | Diet  Physical activity | Successful implementation has to address issues across the organisation (i.e. all staff and non-staff groups) and at multiple levels (individual, group and strategic). This is particularly the case if changes are to be institutionalised, i.e. the sustainability of changes. | None |
| [Ozer (2006](#_ENREF_21)) | Proposes framework encompassing individual, school and community level factors | Violence | Readiness to change/ ‘buy-in’ from staff at all levels and students.  Quality of staff-student relations. | ‘Fit’ between settings and programmes and the extent to which the school context enables teachers to deliver the programme as intended.  ‘External context’ (e.g. prevailing societal or community norms around violence) may dominate and act as a significant barrier to implementation.  History of programme – e.g. where teachers are cynical because of prior, unsuccessful attempts at change. |
| [Robertson-Wilson et al. (2009](#_ENREF_22)) | Hogwood & Gunn’s ([1984](#_ENREF_14)) ‘ideal’ preconditions for implementation | Physical activity | Adequate time and resources.  Objectives are understood and agreed upon. | Ideal conditions for implementation [Hogwood and Gunn (1984](#_ENREF_14)):  - circumstances outside of the implementing agency do not “impose crippling constraints”  - the relationship between programme and outcomes is direct, “with few intervening variables”  - “tasks are fully specified in the correct sequence” |

References

ADELMAN, H. S. & TAYLOR, L. 1997. Toward a Scale-Up Model for Replicating New Approaches to Schooling. *Journal of Educational and Psychological Consultation,* 8**,** 197-230.

ARBEIT, M. L., SERPAS, D. C., JOHNSON, C. C., FORCIER, J. E. & BERENSON, G. S. 1991. The implementation of a cardiovascular school health promotion program: utilization and impact of a school health advisory committee: the Heart Smart program. *Health Education Research,* 6**,** 423-430.

BASCH, C. E. 1984. Research on disseminating and implementing health education programs in schools. *Journal of School Health,* 54**,** 57-66.

BISSET, S., DANIEL, M. & POTVIN, L. 2009. Exploring the intervention-context interface: A case from a school-based nutrition intervention. *American Journal of Evaluation,* 30**,** 554-571.

CHOI, J. N. 2003. How Does Context Influence Individual Behavior? Multilevel Assessment of the Implementation of Social Innovations. *Prevention & Treatment,* 6**,** 23.

CORBOY, D. & MCDONALD, J. 2007. An evaluation of the CAST program using a conceptual model of school-based implementation. *Australian e-Journal for the Advancement of Mental Health,* 6**,** 1-15.

DUSENBURY, L., BRANNIGAN, R., FALCO, M. & HANSEN, W. B. 2003. A review of research on fidelity of implementation: Implications for drug abuse prevention in school settings. *Health Education Research,* 18**,** 237-256.

DUSENBURY, L., BRANNIGAN, R., HANSEN, W. B., WALSH, J. & FALCO, M. 2005. Quality of implementation: developing measures crucial to understanding the diffusion of preventive interventions. *Health Education Journal,* 20**,** 308-313.

GABRIELSEN, T. S. 1993. Action Oriented Health Education: A Critical Review of Health Campaigns in Denmark. *Health Promotion International,* 8**,** 13-19.

GRACZYK, P. A. 2000. Criteria for evaluating the quality of school-based social and emotional learning programs. *In:* BAR-ON, R. & PARKE, J. D. (eds.) *The handbook of emotional intelligence: Theory, development, assessment, and application at home, school and in the workplace.* San Francisco, CA: Jossey-Bass.

GREENBERG, M. T., DOMITROVICH, C. E., GRACYK, P. A. & ZINS, J. E. 2005. The study of implementation in school-based preventive interventions: Theory, research and practice. Washington, DC: U.S. Department of Health and Human Services.

HARACHI, T. W., ABBOTT, R. D., CATALANO, R. F., HAGGERTY, K. P. & FLEMING, C. B. 1999. Opening the black box: using process evaluation measures to assess implementation and theory building. *American Journal of Community Psychology,* 27**,** 711-731.

HOAGWOOD, K. & JOHNSON, J. 2003. School psychology: A public health framework I. From evidence-based practices to evidence-based policies. [References]. *Journal of School Psychology,* .41.

HOGWOOD, B. W. & GUNN, L. A. 1984. *Policy analysis for the real world,* Oxford, Oxford University Press.

JENSEN, B. B. & SIMOVSKA, V. 2005. Involving students in learning and health promotion processes--clarifying why? what? and how? *Promotion et Education,* 12**,** 150-156.

JOWERS, K., L. 2007. Taking school-based substance abuse prevention to scale: District-wide implementation of Keep A Clear Mind. [References]. *Journal of Alcohol and Drug Education,* .51.

KALAFAT, J., ILLBACK, R. J., SANDERS, D., JR., KALAFAT, J., ILLBACK ROBERT, J. & SANDERS, D., JR. 2007. The relationship between implementation fidelity and educational outcomes in a school-based family support program: development of a model for evaluating multidimensional full-service programs. *Evaluation & Program Planning,* 30**,** 136-148.

KEALEY, K. A., PETERSON, A. V., JR., GAUL, M. A., DINH, K. T. & PETERSON, A. V. J. 2000. Teacher training as a behavior change process: principles and results from a longitudinal study. *Health Education & Behavior,* 27**,** 64-81.

MOORE, S., N. 2011. Health improvement, nutrition-related behaviour and the role of school meals: The usefulness of a socio-ecological perspective to inform policy design, implementation and evaluation. *Critical Public Health,* .21.

OSGANIAN, S. K., PARCEL, G. S., STONE, E. J., OSGANIAN STAVROULA, K., PARCEL GUY, S. & STONE ELAINE, J. 2003. Institutionalization of a school health promotion program: background and rationale of the CATCH-ON study. *Health Education & Behavior,* 30**,** 410-417.

OZER, E. J. 2006. Contextual effects in school-based violence prevention programs: A conceptual framework and empirical review. *The Journal of Primary Prevention,* 27**,** 315-340.

ROBERTSON-WILSON, J. E., LEVESQUE, L., ROBERTSON-WILSON JENNIFER, E. & LEVESQUE, L. 2009. Ontario's daily physical activity policy for elementary schools: is everything in place for success? *Canadian Journal of Public Health,* Revue Canadienne de Sante Publique. 100**,** 125-129.

SAMDAL, O. & ROWLING, L. 2011. Theoretical and empirical base for implementation components of health-promoting schools. *Health Education,* 111**,** 367-390.
