# Supplementary material for: Implementing health promotion programmes in schools: a realist systematic review of research and experience in the United Kingdom
Source: Implement Sci. 2015 Oct 28;10:149. doi: 10.1186/s13012-015-0338-6 (PMC4625879; doi:10.1186/s13012-015-0338-6)
Supplement: Additional file 3: — Long list of programme theories. The file contains a list of 12 programme theories that encompassed all of the concepts around implementation identified in the 22 conceptually rich sources and to identify the unique contributions of each source. (DOCX 17 kb) [file 13012_2015_338_MOESM3_ESM.docx]

| **No.** | **Programme theory** |
| --- | --- |
| 1 | Preparing and planning for school development – Systematic planning identifying clear aims and priorities, taking into account the current situation (including stakeholders’ ‘readiness’) and competence at school level, and consulting stakeholders, is vital to achieve sustainable change. The ‘readiness’ of stakeholders for programme implementation depends on adequate provision of information, people’s prior experiences of programmes, and the concordance (‘fit’) of the programme with stakeholders’ current practice, students’ interests and schools’ existing policies, strategies and power structures. |
| 2 | Policy and institutional anchoring – Inclusion of an intervention in policy documents and use of process to ensure institutional anchoring is likely to stimulate commitment in staff and help prioritise resources to the intervention. The anchoring processes using strategies of alignment are core to building readiness and organisation capacity. |
| 3 | Professional development and learning – Training in leadership and identified priority areas helps build motivation and competence for executive staff, the core team and other staff, essential for quality implementation of change. |
| 4 | Leadership and management practices – Leadership actions in building ownership in the school community and anchoring the activities to school visions through feedback, encouragement and expectations to implement actions are fundamental to maintain focus and motivation among stakeholders for agreed change. |
| 5 | Relational and organisation context – The social relations and the structural conditions of a school, including expectations about behaviour and values and the focus of teachers’ relationships with pupils (nurturing or educational attainment) can impact on the achievement of agreed actions by providing a stimulating climate and opportunities. |
| 6 | Student participation – Student participation is a means and a goal to maximise motivation for health and learning. It values and provides conditions for them to be empowered. |
| 7 | Partnerships and networking – Active parental involvement in a variety of ways can facilitate parental support for values and actions of the school. Involving relevant collaborators may stimulate action and commitment through complementary expertise and expectations. |
| 8 | Sustainability – Long-term maintenance of the initiative is dependent on a continued focus on conditions that facilitate and ensure implementation of agreed actions for change, including ongoing development of the programme and integration of new knowledge into its delivery. |
| 9 | Management of emerging network(s) – The introduction of a programme takes time. It is likely to involve changes in the school environment and the development of new networks of relationships that require pro-active management so that different actors’ goals are reconciled and organisational decisions are co-ordinated. Co-operation between school staff and external stakeholders is important for harnessing stakeholders’ existing relationships with pupils, and their enthusiasm, knowledge and experience. |
| 10 | Programme adaptation – Specificity about ‘core’ and ‘peripheral’ programme elements is required to inform teachers’/others’ decision-making about how to deliver a programme in the unique context of their school. Scope for ‘mutual adaptation’ between the programme and the people delivering the programme is essential for successful implementation. |
| 11 | External contexts – Societal or community norms or the current state of local or regional administration, leadership or working relationships may overwhelm efforts to implement a programme. |
| 12 | Materials – Teaching materials that are appealing and appropriate to students’ age, interests and culture. |
