# Supplementary material for: Implementing health promotion programmes in schools: a realist systematic review of research and experience in the United Kingdom
Source: Implement Sci. 2015 Oct 28;10:149. doi: 10.1186/s13012-015-0338-6 (PMC4625879; doi:10.1186/s13012-015-0338-6)
Supplement: Additional file 4: — review advisory group. The RAG was formed to contribute to the identification, selection, and refinement of programme theories to be tested in the review. (DOCX 24 kb) [file 13012_2015_338_MOESM4_ESM.docx]

The Review Advisory Group (RAG) was formed to contribute to the identification, selection and refinement of programme theories to be tested in the review. The group comprised primary and secondary school-level educational professionals and senior academics linked to the review (with relevant experience of developing, delivering and evaluating health promotion programmes in schools). Educational professionals in the RAG included a PSHE Lead from a state secondary school, a primary school headteacher, and a retired primary school headteacher. Public Health professionals in the RAG included a Professional Lead for School Nursing, Senior Health Promotion Practitioner, Healthy Schools programme co-ordinator, and NHS Directorate Head of Health Policy. Table 1 lists the members of the RAG and their meeting attendance. Educational professionals were recruited through the Devon Association of Primary Headteachers and school contact lists within Devon established through ongoing Institute of Health Services Research (University of Exeter Medical School). To maintain goodwill with schools that were already actively involved with research projects, we approached only those that were not currently involved with research.

Table 1 Membership of Review Advisory Group

| **Name** | **Role** | **RAG#1** | **RAG#2** |
| --- | --- | --- | --- |
| Charles Abraham | Professor, University of Exeter | ● | ● |
| Betsy Allen | Professional Lead for School Nursing (Devon) |  | ● |
| Tamsin Ford | Professor, University of Exeter Medical School |  | ● |
| David Glenny | Primary school headteacher (retired) | ● | ● |
| Greg Ireland | PHSE advisor, ISCA College (Exeter) | ● | ● |
| Molly Marlow | Headteacher, (Willow Brook Primary School (Exeter) | ● |  |
| Rhiannon Phillips | Senior Health Promotion Practitioner, Public Health Wales | ● | ●^*^ |
| Kate Pordage | Cornwall Healthy Schools Programme Co-ordinator | ● |  |
| Ian Tearle | Head of Health Policy, Directorate of Public Health, NHS Devon | ● | ● |
| Katrina Wyatt | Associate Professor, University of Exeter Medical School | ● |  |

**^*^** joined meeting by phone

All potential participants were provided with background knowledge about the project and information about what their role would involve if they joined the group. The overarching aims of the RAG were to:

1. Sharpen the focus of the review so that it would be relevant to those directly involved in the implementation of health promotion programmes in schools
2. Provide a ‘reality check’ for the review team through the discussion of emerging findings from the review and their relevance to the current UK context
3. Contribute to shaping the presentation of the review’s findings so that it would be relevant to and usable by the intended audiences

Once members were recruited, the first meeting was held in September 2012 with the aims of providing participants with more details and progress relating to the project; develop relations between the core review team and participants; and to focus the review based on feedback gathered from members of the RAG. See Table 1 for detailed content and the evolving different aims of each of the two meetings.

Table 2 Content and aims of the RAG meetings

| **RAG meeting** | **Content and aims of meeting** |
| --- | --- |
| Meeting 1  18th September 2012 | Introductions (research team, RAG members)  Aims and approach of the review  Aims of the Review Advisory Group meetings  Whole group discussion - What helps or hinders the implementation of health promotion programmes in UK schools?  Whole group discussion - Good and bad experiences of implementing health promotion programmes in schools |
| Meeting 2  4th March 2013 | Project overview and progress  Whole group discussion and feedback on draft review components:   - conceptual framework - programme overview table - draft synthesis examples   Whole group discussion - Audiences for the review and how best to communicate review findings |

Discussions within the RAG covered a wide variety of implementation material. The following represents a snap shot of some areas discussed within the initial group meeting. It was acknowledged that schools have long been the setting for certain types of health education and activities to promote healthy lifestyles (e.g. sex education, Physical Education lessons). Schools or teachers are also seen as a key source of referral for certain “health services” (e.g. speech & language therapy, educational psychology). In addition, it was stated that while PSHE health education is now part of the National Curriculum, a lack of content specification and the extent and quality of provision of PSHE is so variable that it is effectively non-mandatory. A number of factors affecting initial access to, acceptability or feasibility within a school were noted with the head and senior teachers, lead teachers for PSHE and other teaching staff being the main gatekeepers. Schools were described as idiosyncratic and autonomous in terms of how they function, having a good understanding of their own needs and priorities. It was suggested that external providers of health promotion initiatives should be clear about how a programme is supposed to work and that discussion should be had with the school/headteachers regarding the programme’s suitability within their school.

Key factors mentioned for trying out a new programme/activity (if externally offered) were cost, time commitment, physical space and whether it tackles unmet problem/subject need. Broader contextual considerations for competing activities and goals within schools, included educational/exam achievements, including pressure on some headteachers not to commit any staff resources or school time for activities or programmes that do not also contribute towards those educational outcomes (e.g. exam results). It was also noted that particular teachers may have what was termed ‘initiative fatigue’ and that health promotion programmes may be perceived as just another ‘great idea’ that needs to be handled (and ultimately ignored) rather than genuinely engaged with.

A number of factors affecting initial implementation were discussed, these related to the ‘co-creation’ and ‘co-delivery’ of programmes between teachers and external (e.g. topic experts); using external experts who were particularly compelling and engaging with the young people, and; that health promotion or education may be delivered by non-teachers such as Child Welfare Officers or school nurses. It was mostly agreed that the longer term/repeated provision of health promotion programmes or provision by more schools was especially dependent on the people delivering them having sufficient time, organisational support, competence and relevant knowledge, and confidence to deliver them well. In addition, differing types of school and school contexts may also impact on implementation; for example, having timetable slots for PSHE; the need to tailor programmes (e.g. to be able to define core (essential) elements from optional (supportive/complementary) features of a programme), finally that certain schools are not ‘written off’ as being inappropriate for delivery of programmes.

Important aspects of a programme or its activity related to issues of universal versus targeted programmes and whether there was parental engagement. Inherent limitations or unavoidable challenges of working in schools related to the level of attitude/behaviour change possible solely by providing activities or programmes in school and the strong influence of parental/community/societal norms was acknowledged.

It was also recognised that external policies and national initiatives have a role (e.g. Healthy Schools accreditation programme) but that there was a danger they could become “tick-box” exercises, encouraging minimal or piecemeal changes rather than fundamental or whole school change. The profile of health education/promotion in schools itself (as opposed to specific programmes) could be raised by a national policy and/or local (city/regional) endorsement i.e. “we should be doing this as well” type incentives or expectations. Finally, a number of points were raised regarding programme/activity effectiveness, such as the measurement and monitoring of outcomes.

Following this meeting, comments were incorporated into four key programme theories and circulated to the RAG members. In selecting these programme theories consideration was also given to them being able to:

1. Offer the greatest potential explanatory power (i.e. ability to explain differences in successfulness within and between programmes).
2. Be ‘testable’ (i.e. provide enough detail to support the presence (and/or its strength) of a programme theory or mechanism).
3. Not be too generic (i.e. too vague to be of value to individual schools or those involved in implementation).

The second RAG meeting was held in March 2013 with the aims of updating the group on progress, discussing the development of the conceptual framework, how best to present materials and to share a draft excerpt of the synthesis. These findings have been tabulated in terms of conceptual framework, programme overview table and the draft synthesis. This meeting contributed to the revision of the conceptual framework figure and valuable observations about what could be added to the programme overview table (i.e. years a programme is delivered, extent of evidence located from each programme) as well as observations regarding synthesis presentation (i.e. re-capping on relevant programme theories, providing an executive summary).
