# Supplementary material for: Implementing health promotion programmes in schools: a realist systematic review of research and experience in the United Kingdom
Source: Implement Sci. 2015 Oct 28;10:149. doi: 10.1186/s13012-015-0338-6 (PMC4625879; doi:10.1186/s13012-015-0338-6)
Supplement: Additional file 5: — Contribution of sources to development of programme theories. The file is a documentation of the three stages of programme theory development, showing the areas in which different sources contributed. (DOCX 23 kb) [file 13012_2015_338_MOESM5_ESM.docx]

**STAGE 1**

Using a systematic narrative review approach (Mays et al. 2005), Samdal & Rowling (2011) identified research-based knowledge about the components of the process of implementing health-promoting schools or “an educational change process that focused on health” (p.371) and developed a theoretically-based rationale for each component. Amongst other criteria, studies were excluded if they solely investigated the implementation of a pre-packaged programme. The Samdal & Rowling review therefore focused more on ‘health promoting schools’ (i.e. changing these organisations as whole units in order to promote or enable healthy behaviour) than health promotion *in* schools (the focus of our review). Each of the eight components was expressed in a form amenable to testing (i.e. similar to a programme theory).

**Samdal & Rowling’s (2011) ‘rationales for implementation components’:**

1) Preparing and planning for school development – Systematic planning identifying clear aims and priorities, taking into account the current situation and competence at school level, and consulting stakeholders, is vital to achieve sustainable change.

2) Policy and institutional anchoring – Inclusion of an intervention in policy documents and use of process to ensure institutional anchoring is likely to stimulate commitment in staff and help prioritise resources to the intervention. The anchoring processes using strategies of alignment are core to building readiness and organisation capacity.

3) Professional development and learning – Training in leadership and identified priority areas helps build motivation and competence for executive staff, the core team and other staff, essential for quality implementation of change.

4) Leadership and management practices – Leadership actions in building ownership in the school community and anchoring the activities to school visions through feedback, encouragement and expectations to implement actions are fundamental to maintain focus and motivation among stakeholders for agreed change.

5) Relational and organisation context – The social relations and the structural conditions of a school, can impact on the achievement of agreed actions by providing a stimulating climate and opportunities.

6) Student participation – Student participation is a means and a goal to maximise motivation for health and learning. It values and provides conditions for them to be empowered.

7) Partnerships and networking – Active parental involvement in a variety of ways can facilitate parental support for values and actions of the school. Involving relevant collaborators may stimulate action and commitment through complementary expertise and expectations.

8) Sustainability – Long-term maintenance of the initiative is dependent on a continued focus on conditions that facilitate and ensure implementation of agreed actions for change.

**STAGE 2**

**Development of Samdal & Rowling’s theories using conceptually-rich papers and discussion at first Review Advisory Group meeting:**

Black text – Samdal & Rowling (2011) theory, based on their review

Red text – additions sourced from the 21 conceptually-rich papers identified in our review

Blue text – additions sourced from first Review Advisory Group meeting

Components of the theories that were identified in more than one of the three sources (Samdal & Rowling 2011; the 21 conceptually-rich papers; Review Advisory Group) are identified only as the source in which they were first identified.

1) Preparing and planning for school development – Systematic planning identifying clear aims and priorities, taking into account the current situation (including stakeholders’ ‘readiness’^[[1]](#footnote-1)^) and competence at school level, and consulting stakeholders, is vital to achieve sustainable change. The ‘readiness’ of stakeholders for programme implementation depends on adequate provision of information^[[2]](#footnote-2)^, people’s prior experiences of programmes^[[3]](#footnote-3)^, and the concordance (‘fit’) of the programme with stakeholders’ current practice^[[4]](#footnote-4)^, students’ interests^[[5]](#footnote-5)^ and schools’ existing policies, strategies and power structures^[[6]](#footnote-6)^.

2) Policy and institutional anchoring – Inclusion of an intervention in policy documents and use of process to ensure institutional anchoring is likely to stimulate commitment in staff and help prioritise resources to the intervention. The anchoring processes using strategies of alignment are core to building readiness and organisation capacity.

3) Professional development and learning – Training in leadership and identified priority areas helps build motivation and competence for executive staff, the core team and other staff, essential for quality implementation of change.

4) Leadership and management practices – Leadership actions in building ownership in the school community and anchoring the activities to school visions through feedback, encouragement and expectations to implement actions are fundamental to maintain focus and motivation among stakeholders for agreed change.

5) Relational and organisation context – The social relations and the structural conditions of a school, including expectations about behaviour and values^[[7]](#footnote-7)^ and the focus of teachers’ relationships with children (nurturing or educational attainment) can impact on the achievement of agreed actions by providing a stimulating climate and opportunities.

6) Student participation – Student participation is a means and a goal to maximise motivation for health and learning. It values and provides conditions for them to be empowered.

7) Partnerships and networking – Active parental involvement in a variety of ways can facilitate parental support for values and actions of the school. Involving relevant collaborators may stimulate action and commitment through complementary expertise and expectations.

8) Sustainability – Long-term maintenance of the initiative is dependent on a continued focus on conditions that facilitate and ensure implementation of agreed actions for change, including ongoing development of the programme and integration of new knowledge into its delivery^[[8]](#footnote-8)^.

9) Management of emerging network(s) – The introduction of a programme takes time. It is likely to involve changes in the school environment^[[9]](#footnote-9)^ and the development of new networks of relationships^[[10]](#footnote-10)^ that require pro-active management so that different actors’ goals are reconciled^[[11]](#footnote-11),^^[[12]](#footnote-12)^ and organisational decisions are co-ordinated. Co-operation between school staff and external stakeholders is important for harnessing stakeholders’ existing relationships with children, and their enthusiasm, knowledge and experience^[[13]](#footnote-13)^.

10) Programme adaptation – Specificity about ‘core’ and ‘peripheral’ programme elements is required to inform teachers’/others’ decision-making about how to deliver a programme in the unique context of their school^[[14]](#footnote-14)^. Scope for ‘mutual adaptation’ between the programme and the people delivering the programme is essential for successful implementation.^[[15]](#footnote-15),^^[[16]](#footnote-16),^^[[17]](#footnote-17)^

11) External contexts – Societal or community norms^[[18]](#footnote-18)^ or the current state of local or regional administration, leadership or working relationships^[[19]](#footnote-19)^ may overwhelm efforts to implement a programme.

12) Materials – Teaching materials that are appealing and appropriate to students’ age, interests and culture^[[20]](#footnote-20)^.

**STAGE 3**

**Refinement of programme theories through email discussion with Review Advisory Group**

Blue text – additions and revisions sourced from email discussion with Review Advisory Group

**Programme theory 1 - Preparing for implementation**

Preparation for the introduction of a health promotion programme to a school is more likely to be successful when systematically planned in conjunction with other school responsibilities. This involves:

- identification of a potential health benefit for pupils at a local level
- consultation with stakeholders (head-teacher, school staff, pupils, parents, governors)
- consideration of the concordance of the programme with current practice and interests
- identifying clear aims and priorities, including intended outcomes
- taking into account the current situation and competencies in a school and the implications of these for programme refinement

*Consultation with stakeholders involves*:

- providing information on the programme (e.g. who and what is involved, evidence of effectiveness)
- eliciting views on ‘readiness for change’ as a starting point for engagement
- encouraging the sharing of previous experiences and knowledge of delivering health promotion programmes
- facilitating discussion about the concordance (‘fit’) of the programme with:

- current practice

- pupils’ interests

- current school policies, resources and organisation

**Programme theory 2 - Introducing a programme within a school**

The introduction of a health promotion programme to a school is more likely to be successful when it is incorporated into school activities through:

- being integrated into school policy (e.g. a School Improvement Plan) and supported by governors and senior staff to whom monitoring and progress reports are made
- school staff using their leadership skills to co-ordinate activities or resources for programme delivery
- providing adequate opportunity and/or training for the personal and professional development of those who will deliver the programme and engaging with their interests
- modes of delivery that appeal to and engage pupils and provide cognitive and/or emotional rewards
- providing support materials that are appealing and appropriate to pupils’ age, interests and culture

**Programme theory 3 - Embedding a programme into routine practice**

The routine delivery (‘embedding’) of a programme takes time and motivation. It is likely to involve changes in the school environment and the development of new relationships between stakeholders that require pro-active management so that:

- different stakeholders’ goals are reconciled
- organisational decisions in other areas of school life are made taking into account how they impact on programme delivery
- school staff’s existing relationships with children are built upon
- stakeholders’ enthusiasm, knowledge and experience are harnessed
- knowledge of ‘core’ and ‘peripheral’ elements and minimum resources, skills and informational content is retained
- responsibility for programme delivery becomes rooted in the school

The longer-term sustainability of programmes and the extent to which health promoting messages and activities permeate other aspects of school life, is dependent on continuing feedback, encouragement, and expectations about implementation. Over time, this may originate less from outside the school and more from inside.

**Programme theory 4 - Programme adaptation**

The preparation for, introduction, initial delivery, and ongoing sustainability of a health promotion programme in a school is more likely to be successful when there is:

- specificity about ‘core’ (essential) and ‘peripheral’ (optional/adaptable) programme elements, including the minimum levels of resources and/or skills necessary to support these elements (to inform decision-making about how to deliver a programme in the context of a particular school)
- scope for ‘mutual adaptation’ between the programme and the people delivering it, including the evolution and updating of programme content and mode of delivery over time

1. Adelman & Taylor (1997) [↑](#footnote-ref-1)
2. Jowers et al. (2007) [↑](#footnote-ref-2)
3. Greenberg et al. (2005) [↑](#footnote-ref-3)
4. Greenberg et al. (2005); Hoagwood & Johnson (2003) [↑](#footnote-ref-4)
5. Hoagwood & Johnson (2003) [↑](#footnote-ref-5)
6. Hoagwood & Johnson (2003); Ozer 2006; Basch (1984) [↑](#footnote-ref-6)
7. Hoagwood & Johnson (2003) [↑](#footnote-ref-7)
8. Jowers et al. (2007) [↑](#footnote-ref-8)
9. Jowers et al. (2007) [↑](#footnote-ref-9)
10. Dusenbury et al. (2003) [↑](#footnote-ref-10)
11. Bisset et al. (2009) [↑](#footnote-ref-11)
12. Adelman & Taylor (1997) [↑](#footnote-ref-12)
13. Kealey et al. (2000) [↑](#footnote-ref-13)
14. Dusenbury et al. (2003); Greenberg et al. (2005) [↑](#footnote-ref-14)
15. Kealey et al. (2000); Basch (1984); Bisset et al. (2009) [↑](#footnote-ref-15)
16. Adelman & Taylor (1997) [↑](#footnote-ref-16)
17. Graczyk et al. (2000) [↑](#footnote-ref-17)
18. Ozer (2006) [↑](#footnote-ref-18)
19. Greenberg et al. (2005) [↑](#footnote-ref-19)
20. Greenberg et al. (2005) [↑](#footnote-ref-20)
