# Supplementary material for: Implementing health promotion programmes in schools: a realist systematic review of research and experience in the United Kingdom
Source: Implement Sci. 2015 Oct 28;10:149. doi: 10.1186/s13012-015-0338-6 (PMC4625879; doi:10.1186/s13012-015-0338-6)
Supplement: Additional file 6: — Programme descriptions. The file reports the full details of each programme. (DOCX 26 kb) [file 13012_2015_338_MOESM6_ESM.docx]

| **HP prog** | **Programme description** |
| --- | --- |
| Active Primary School | ‘The Active Primary School Pilot Programme (APSPP) provides Co-ordinators to work with primary schools in order to increase the range and quality of opportunities for primary-aged children to become more physically active. It approaches these aims by providing relevant opportunities (play, sport and recreation, physical education, and active travel) both within and outwith the formal school curriculum.’ Developed in response to the *Youth Sport Strategy for Scotland* (1996). Aimed to involve teachers, parents, the school board and members of the Parent Teacher Association, and ‘where appropriate, partnerships with other agencies and health initiatives are also pursued’. Co-ordinators’ work was aimed at facilitating teachers to incorporate a range of physical activities (sports, games, dance, skill development) into the curriculum and ‘encouraging physical activity to feature in all schools’ development planning’. |
| APPLES | ‘A multidisciplinary, multiagency programme using a population approach. The programme team includes a dietitian, community paediatrician, health promotion specialist, psychologist, obesity physician and nutritional epidemiologist. The programme targeted the whole school community including parents, teachers, catering staff and the school environment. It was designed to take place over one academic year and was based on the concept of school action plans, which were to be developed by the individual schools on the basis of their perceived needs. The programme was intended to influence dietary and physical activity behaviour and not simply knowledge in the school children. The team provided training for teachers and some resources, and the project manager also provided input and contracted the schools regularly throughout the year to give support. The underlying approach was to be non-prescriptive and to ensure ownership of the programme by the schools’ (p.1-2). Programme implementation was supported by a project manager who provided ‘direct input’ to programme activities in schools. |
| ASSIST | The ASSIST intervention model was based on: recruitment of influential students who were nominated by their peers, and implementation by external trainers rather than school teaching staff. Based on diffusion of innovation theory (how ideas and practices spread within communities through personal interaction), ‘influential’ students were trained to become ‘peer supporters’ with regard to reducing/stopping smoking amongst their peers. The role of peer supporters was to provide health information and advice, support peers and act as role models.  Influential students were identified via a survey of Year 8 students, and invited to take on the role. Training (2 days, off school premises) was a ‘student-centred experiential process’ which had been developed by a team of health promotion specialists, teachers and youth trainers. It was designed to increase peer supporters’ knowledge about smoking health risks, improve their communication skills and contribute to their personal development. Post-training programme visits were made to peer supporters and one, four and seven weeks so as to offer support, resolve problems, and provide an opportunity to consolidate their skills and learning. |
| Blueprint | Developed over 3 years (led by Home Office ‘in partnership with’ Dept.of Health and Dept. for Education and Skills). Rather than adapting existing programmes to the English context, the programme was ‘based on the distillation of key principles of effective drug education rather than relying on any one programme model, although it was particularly informed by Project STAR and Life Skills Training.’ (and Dusenbury & Falco’s (1995) principles of effective drug education). Lesson materials and plans were piloted. Programme consisted of five connected drug prevention components delivered in 23 schools:  1) Schools – curriculum (15 lessons, approx. 50 minutes per lesson) delivered over 2y with a normative focus (‘demonstrating to young people that both actual rates of drug use, and approval of drug use, are lower than they think, and examines social influence factors within peer networks and through the media’. Supporting resources were provided for pupils, parents, teachers and school governors. Teachers received a total of 6 days of training and funding was provided to extend the role of School Drug Advisers.  2) Parents – series of 6 parenting skill workshops (bullying, relationships and talking about drugs)  3) Media – ‘A media relations agency was contracted to generate news coverage about Blueprint throughout the study and to co-ordinate stakeholder communications.’  4) Health policy – Local Authorities in the four Blueprint areas received additional funding to increase work in retailer education and training, youth education, proof-of-age schemes, test purchasing, prosecutions, and media communications.  5) Community – activities were ‘developed to involve the wider community of drug professionals and organisations’ |
| Citizenship Safety Project | ‘In response to concerns to widen the age range covered by injury prevention education in schools, the Injury Minimization Programme for Schools (IMPS) team developed the Citizenship Safety Project (CSP). This innovative project supports citizenship education and can be integrated into the National Curriculum in PSHE lessons, meeting requirements of Key Stage 4 attainment targets in citizenship. The CSP is a cross-age tutoring intervention in which Year 10 (14–15 years) students teach Year 2 pupils (6–7 years) aspects of accident prevention and risk awareness. |
| Eat Smart Play Smart | ‘The Be Smart programme lasted for four school terms (~14 months) and was held over 8 weeks per term, weekly in term 1 and fortnightly in terms 2-4, giving an intervention of 20 weeks in total. Intervention groups (nutrition and/or physical activity curriculum) lasted 25 minutes, were delivered at lunchtime clubs, had an interactive approach and were behaviourally focussed (based on Social Learning Theory - Bandura, 1986) and incorporated:  - raising the value of the desired behaviour, including the short-term benefits, which are most likely to appeal to children  - providing the opportunity to taste healthy foods and undertake non-competitive physical activity  - providing incentives to reinforce messages, for example verbal praise and small prizes  - developing practical skills and thus self-confidence in the desired behaviour  - working with parents (as far as possible) to overcome barriers to the desired health behaviour.  All materials developed were reviewed by experienced primary school teachers and amended on the basis of their recommendations.’ |
| Family Smoking Education | Adapted from a Norwegian programme. Consisted of a teachers’ guide (programme background and suggestions for pupil activities), parents’ leaflet (to encourage active parental participation consistent with the programme aims), and pupils’ booklet (education about effects of smoking on the body and wider pollution). Although a minimum time period for delivery (NR) and teachers were asked to cover every theme of the project, schools were free to deliver the project materials in a way that they felt best suited (e.g. within pastoral or tutorial time (12 schools); social education lessons (2); health education (1); English lessons (1); Religious Education lessons (1); Science lessons (3). Mean time spent delivering = 3h. Wide range in the number of lessons used (from 2 lessons of 70m to 11 lessons of 35m). Around two-thirds of schools used class discussion and teacher-led activities, one-third used groupwork, and <20% used a practical demonstration. |
| GGHB Sexual health programme | The sexual health education model developed by Greater Glasgow Health Board (GGHB) was part of an on-going effort by the health board to design a school-based approach for use in areas of multiple deprivation. The main aim of the programme was to ‘raise awareness of the meaning of full-sexual well-being, encourage informed choice, and discourage unplanned pregnancy’. The programme was developed following two years of pilot work, emphasised a non-judgmental ethos and was delivered by teachers (over 5 weeks, 1 lesson per week) to mainly single sex groups as part of the PSE curriculum. Teachers had access to GGHB programme staff for advice. Programme involved whole class, small group and individual work, with supporting materials such as video, reading material and quizzes. Topics included biology and physiology, relationships, safer sex, pregnancy, and communication and power in relationships. Project work aimed to ‘enable students to examine in detail sexual health issues that affect them’.  Programme approach summarised as: ‘grounded in a belief that pupils should explore their own lives in regard to sexuality and sexual health. This experiential approach emphasises pupils’ ownership of the issues and challenges misconceptions and myths with factual information. Pupils are encouraged to process their own decisions concerning sexual activity. Programme aims to encourage peer-support within classes to counter sexual pressure and embarrassment over body changes.’ |
| HeLP | ‘The Healthy Lifestyle Programme (HeLP) is a school-based intervention which seeks to deliver healthy lifestyle messages and provide simple individually tailored strategies to assist change relating to healthy activity and eating. HeLP takes a population approach seeking to change behaviour at a family as well as at an individual and institutional level. HeLP aims to deliver a general healthy lifestyle message relating to the energy balance and within this context, three evidence-based key messages are emphasised: a decrease in the consumption of sweetened fizzy drinks, an increase in the proportion of healthy snacks consumed and a reduction in TV viewing and other screen-based activities.’ (p.2) The following activities took place:  Term 1 – Assembly, competition, rugby and dance workshops, parents’ evening  Term 2 – PSHE lessons, drama activities (‘children choose which character they most resemble and then work with that character to help them change their behaviour’ (p.7), parent information sheets, 1:1 goal-setting, newsletters, parents’ evenings  Term 3 – Assembly, PSHE lesson, drama workshop, class assembly, 1:1 goal reassessment, newsletter  The 18-month programme aimed to use processes of behaviour change, namely:  - establishing motivation and creating a receptive environment  - developing children’s confidence and skills and helping them make decisions  - helping children create an action plan and implement goals  - keeping children motivated by helping them to monitor, assess and adapt goals |
| KAT | Kids, Adults Together (KAT) comprises of a classroom component, engagement with parents through a fun evening for families with children aged 9-11 years, and a specially made DVD for children to take home, for families to watch together. |
| NE Choices | ‘NE Choices is a multi-component drugs prevention programme for young people in the North-East of England. Based on a ‘social influences approach’ to drugs prevention, developed using social marketing principles / techniques. Integrated intervention components were an all day out of school drama workshop [supported by video and software] (Year 10), classroom support and follow-up (for workshop), six month youth work projects (Year 11), intensive outdoor activity programme for ‘high-risk’ young people (Year 11), drama and drugs awareness sessions for parents, also training for teachers, youth workers and school governors.’. Findings from formative evaluation in the first year of delivery was used to refine programme components. Teachers received 1-day training and a manual to support delivery. Year 10 pupils could volunteer to ‘devise and deliver a piece of drugs education work of their own choosing to peers’. Two youth workers were recruited to provide 30 sessions (each of 3h), with some additional funding provided by LAs. Parents were involved in order to ‘identify and explore their drugs information and other needs… [findings were used to] inform the development of a drama session for parents focusing on the theme of parent-child communication.’ |
| PhunkyFoods | ‘The PhunkyFoods Programme (PFP) is an initiative launched in 2005 by Purely Nutrition, teaches primary children key messages related to healthy eating and physical exercise in a light hearted and fun manner through art, drama, music, play and practical experience with food. It aims to enhance pupil performance, increase concentration, and improve behaviour, motivation and self-esteem. Lesson plans are available to teach a one-hour lesson of healthy eating and a one-hour lesson of physical activity every week to every primary year group, although schools can deliver the PFP in a variety of ways (in different curriculum contexts and/or during out of hours school clubs) and for varying lengths of time. In addition to training for teachers and classroom assistants (supplemented by extensive online resources), support is provided through various resources such as DVDs, books and games, which schools receive when they join the PFP.’ Each school had a named PFP co-ordinator. |
| Project Tomato | Using the Theory of Planned Behaviour as a basis, the programme consisted of core and customisable elements designed to address the factors that initiate and maintain change in eating behaviours (familiarisation, repetition, activities, modelling and the environment).  Core elements: manual and 12 curriculum-related lesson plans  Customisable elements (headteachers could decide which to adopt): cooking lessons, growing club information, funding support, information to setup a Project Tomato team.  Support materials: Project Tomato kit bags, newsletters and parent handouts. |
| RIPPLE | ‘Peer-led sex education. Peer-educators were trained by an external team to prepare classroom sessions aimed at improving the younger pupils’ skills in sexual communication and condom use, and their knowledge about pregnancy, STIs (including HIV), contraception, and local sexual health services. The peer-educators were also given support to prepare lesson plans and identify resources. Ongoing help was provided by teachers who organised suitable times for teams of peer educators to deliver three sessions of SRE to year 9 pupils. Teachers were not present in the classroom. Every session lasted around 1 h and used participatory learning methods and activities focusing on relationships, STIs, and contraception. These sessions replaced the usual teacher-led SRE delivered during personal, social, and health education in intervention schools. The control schools continued with their usual teacher-led SRE and they received £1800 to spend on anything except SRE.’ |
| Schools on the Move | Website – ‘through which pupils can enter daily pedometer readings and track their personal step-count progress. Teachers also have access to the site, enabling them to track progress of individuals, classes and year groups and to target intervention accordingly. Lead teachers within each school (who are not always PE teachers) receive training before pedometers are distributed to pupils. The training covers familiarisation with website resources, ideas for embedding physical activity into the curriculum and whole school ethos. Lead teachers are expected to cascade the training to teachers of participating classes within their respective schools, and to encourage colleagues to register as pedometer-wearing participants themselves.’ <http://www.pathsforall.org.uk/component/option.../task,doc_download/> (Inchley, Cuthbert (2007) ‘An investigation of the use of pedometers…’)) |
| SHARE | The SHARE intervention (Sexual Health and Relationships: Safe, Happy and Responsible) is a five day teacher training programme plus a 20 session pack: 10 sessions in the third year of secondary school (at 13­14 years) and 10 in the fourth year (at 14­15 years). It is intended to reduce unsafe sexual behaviours, reduce unwanted pregnancies, and improve the quality of sexual relationships. The programme was developed and piloted in Scotland over two years in consultation with teachers, sex education specialists, and education and health promotion departments. The programme combines active learning (for example, work in small groups and games), information leaflets on sexual health, and development of skills, primarily through the use of interactive video but also through role playing. In the 12 control schools sex education for third and fourth years varied from seven to 12 lessons in total and was primarily devoted to provision of information and discussion.  The process of programme development – two pilots; the first in 4 Scottish schools, with 9 teachers (17 classes) – evaluated through observation of lessons, participant surveys and semi-structured interviews with teachers and pupils. Programme development commented on by 5 sex education experts and lead researchers of related evaluations. This ‘resulted in substantial changes to pilot materials... [allowing] practical constraints [to be] addressed in the design and piloting stage.’. Second pilot in a further 4 schools (15 teachers, 23 classes) used same evaluation methods – this time, lead to smaller revisions to teachers’ pack/course. |
| Smoking and Me | Adapted from a US programme – teachers’ guide provides 5 lesson outlines (emphasising ‘recognising and practicing skills for managing social situations in which smoking occurs’) and ‘background information on young people and smoking, guidance for teachers on choosing group leaders… and managing groups’. No visual aids or pupil project materials are provided. A large part of the ‘work’ of the programme is done by pupils themselves in small groups – ‘group leaders [peer leaders – same age] lead discussion, role-play and decision-making activities’, with support from teachers.  Prior to programme delivery, all staff from the schools who would be involved with its teaching were asked to attend a 1-day training course – which aimed to provide information about the issue (smoking and young people) and ‘familiarise teachers with the project guide and the group leader approach to teaching’. Contributors to the training day were: researchers, health education officers, and teachers who had previously taught the programme. |
| SPICED | The SPICED programme consists of seven classroom-based lessons facilitated by the classroom teacher, a police officer and a school nurse. The three professionals worked in partnership for the duration of the programme using a SPICED pupils’ booklet and a range of supporting resources (e.g. substance use picture cards). Role play, participative learning and group discussion featured prominently in the programme. All professionals delivering SPICED participated in a two day training course and seven classroom-based sessions including an initial session with parents/carers. The other six sessions aimed to provide children with information about legal and illegal drugs, develop their understanding of risk, support them in discussing and exploring substance use issues, as well as promoting their personal confidence, self-esteem and decision making skills.’ |
| UK Resilience Programme | The UK Resilience Programme is the first larger-scale use of the Penn Resilience Program (PRP) curriculum. Implemented as 18 hours of workshops for Year 7 children in 22 UK secondary schools.  ‘The Penn Resiliency Program is intended to build resilience and promote optimistic thinking, adaptive coping skills and social problem-solving in children, with the aim of improving psychological well-being, but potentially also behaviour, attendance and academic outcomes... It is a manualised intervention comprising 18 hours of workshops. The curriculum teaches cognitive-behavioural and social problem-solving skills. Participants are encouraged to identify and challenge negative beliefs, to employ evidence to make more accurate appraisals of situations and others’ behaviour, and to use effective coping mechanisms when faced with adversity. Participants also learn techniques for positive social behaviour, assertiveness, negotiation, decision-making, and relaxation.’. The programme can be delivered by a range of professionals (teachers, learning mentors, teaching assistants, psychologists, health professionals). Preparation for delivery consisted of 8-10 days training *in the USA* (week 1: adult-level Cognitive Behavioural Therapy (CBT) skills; week 2: familiarisation with programme curriculum and ‘practising how to communicate it to pupils’) |
| Y-Active | ‘Y-Active is a multicomponent children’s program delivered by the Central YMCA in London, UK. A component of Y-Active is the outreach PA and well-being program which is delivered in 7 schools in the London area. The case-study school used in the present research represents the most developed partnership, the outcome of approximately 5 years of collaboration between the YMCA and the school.  *Intervention Content.* The Y-Active program consists of (a) breakfast club, (b) physical education, (c) Fit Kids lunchtime fitness classes, (d) after-school play club, and (e) after-school sports. Physical education and Fit Kids (both once per week per year group) are integrated in the school’s provision for all pupils. Breakfast club, after-school play club and after-school sports (once per week per year group) are opt-in activities for which parents pay a small fee.  *Location/Environment.* Breakfast club, Fit Kids classes, and after-school sports are delivered at school in a variety of places, including a sports hall, a dining hall, and the playground. Due to restricted space within the school, physical education is delivered at the nearby YMCA sports center. Pupils walk approximately 10 minutes under supervision to and from this facility.  *Personnel.* All intervention components are organized, planned, and delivered by a team of trained YMCA sports coaches and play workers. A school administrator liaises between the school and the YMCA on logistic issues such as timetabling and collecting fees. The Y-Active manager at the YMCA overseas the broader Y-Active program.’ |
