# Supplementary material for: Implementing health promotion programmes in schools: a realist systematic review of research and experience in the United Kingdom
Source: Implement Sci. 2015 Oct 28;10:149. doi: 10.1186/s13012-015-0338-6 (PMC4625879; doi:10.1186/s13012-015-0338-6)
Supplement: Additional file 7: — Critical appraisal summary. A summary of the key points of the critical appraisal collated in a summary critical appraisal table. (DOCX 21 kb) [file 13012_2015_338_MOESM7_ESM.docx]

| **HP prog** | **Author** | **Critical appraisal summary** |
| --- | --- | --- |
| Active Primary School | Lowden et al. (2004) | Unclear how pilot schools were selected, limited information regarding data collection (i.e. procedure/administration) and analysis. Very limited qualitative evidence provided to judge credibility of statements. Ethics and confidentiality not reported. |
| APPLES | Sahota et al. (2011) | No detail regarding the context of how the programme was implemented and a lack of description regarding focus group attendance, as well as how information and data were analysed. No primary data about the implementation process reported (only authors’ summary). |
| ASSIST | ^1^Audrey et al. 2004  ^2^Audrey et al. 2006 (HER 21)  ^3^Audrey et al. 2006 (SSM 63)  ^4^Audrey et al. 2008  ^5^Holliday et al. 2009 | ^1^Research methods reported in linked papers  ^2^Description of programme development, critical appraisal tool not applicable  ^3^Data collection and analysis thoroughly described and conducted; in-depth interviews appropriate to explore implementation issues. Reasonable level of contextual detail provided.  ^4^Data collection and analysis thoroughly described and conducted; in-depth interviews appropriate to explore implementation issues. Reasonable level of contextual detail provided.  ^5^Limitations in sampling strategy re: how schools were selected for exploration of implementation issues (and how the schools’ characteristics may have impacted on implementation). Data collection not adequately described, but data analysis thoroughly reported and conducted. Candid reflection on the potential impact of the fact that peer trainers and researchers shared office space may have had on implementation processes. |
| Blueprint | ^1^Stead et al. (2007) (D&AR)  ^2^Stead et al. (2007)  ^3^Blueprint Evaluation Team (2007) | ^1^Limited information regarding school settings, lack of detail regarding data collection (i.e. administration, type, procedure) and analyses (i.e. how conducted, supplementary forms of evidence used)  ^2^Although report is in-depth and contains extensive details about implementation issues, there are significant weaknesses in the reporting of research methods, e.g. sampling strategy for survey, interviews and lesson observation not reported; data analysis processes not reported. Link between data and reported findings often unclear. No reflection on m’logical limitations and potential impact on findings.  ^3^Mostly survey-based material so depth is limited in relation to implementation. |
| Citizenship Safety Project | Frederick & Barlow (2006) | Limited information on school context. Opportunistic sample, therefore limited representativeness of population. No triangulation or validity checks reported for qualitative data analysis. |
| Eat Smart Play Smart | Warren et al. (2003) | No detail regarding process and impact evaluation findings i.e. context, primary evidence (authors comments only), data collection (measures only), data analysis, reflexivity or generalisability. |
| Family Smoking Education | Newman & Nutbeam (1989) | Significant weaknesses in reporting (scant or no information on study design, data collection and analysis) suggest that the overall study design and conduct was weak and that findings should be viewed with caution. |
| GGHB Sexual health programme | Lowden & Powney (1996) | Even though the study design (single school) was intended to inform local decision-making by feeding into the development of the programme, there are still considerable limitations – in particular, the sampling and data analysis strategies are not described and the potentially significant impact (social desirability bias) of only conducting (separate) *group* interviews with pupils and teachers is not considered. However, the authors are careful to situate the analysis within key contextual information, e.g. differences between boys’ and girls’ experiences, the local social networks of the pupils. |
| HeLP | Wyatt et al. (2011) | Substantial detail on how Intervention Mapping process was used. The iterative development of the intervention is closely described. However, limited information on how data analysis and interpretation was conducted and no primary supporting data on implementation process provided. |
| KAT | Rothwell & Segrott (2011) | Although a pilot study, convenience sampling strategy and low survey response rate are weaknesses). However, it is clear that the process evaluation endeavoured to include all stakeholders (children, parents, teachers) and to make use of a wide range of data collection methods (survey, focus groups (at a level appropriate for children, including ‘draw & write’), interviews and observation). |
| NE Choices | Stead et al. (2001) | Detailed descriptions of implementation processes, but link between data and findings often not made clear. Sampling strategy for interviews, focus groups, and workshop/classroom observations not reported. Data collection and analysis not adequately reported. |
| PhunkyFoods | Teeman et al. (2008) | Data analysis not reported – lack of assurance regarding validity of data interpretation |
| Project Tomato | Christian et al. (2012) | Study focuses on measuring fidelity of implementation and ‘appreciation’ of deliverers and participants of the programme components – descriptive statistical analysis is appropriate and clearly conducted, but not theoretically-informed (despite the programme itself being theoretically-driven). Contextual information limited to demographics and locations rather than details directly relating to implementation. Questions remain over the reporting of the high survey response rates, where it is unclear whether these were equally distributed amongst the 24 schools or whether these rates are compounded (where more than one survey was conducted). |
| RIPPLE | ^1^Oakley et al. (2004)  ^2^Strange et al. (2006)  ^3^Forrest et al. (2002)  ^4^Strange et al. (2002; HER 327-337)  ^5^Strange et al. (2002; HER 339-349)  ^6^Stephenson et al. (2004) | ^1^Focus of the paper is on how evaluation process can impact on programme delivery and reflections on the issues arising in conducting a process evaluation. Research methods detailed in linked papers (appraised separately).  ^2^Analysis conducted using combined process and outcome data (described a controversial, though guidelines followed)  ^3^Little information about implementation context, but data collection and analysis appropriately conducted. Large numbers of pupils included in focus groups.  ^4^Whilst statistical analysis of survey data is carefully and appropriately conducted, the low response rate of the personally-administered surveys and the lack of data on non-responders is not adequately addressed. Details of FG data collection and analysis are minimal or non-existent.  ^5^Conduct of FGs and data collection/analysis clearly documented and justified. The issue of non-participants in the FGs is acknowledged, but it remains unclear what impact this had on the findings.  ^6^Paper primarily reports trial findings; refers to linked papers for details of process evaluation methods. |
| Schools on the Move | Stathi et al. (2006) | Data collection procedures and use of recognised inventories in survey promise much, but sampling strategies and data analysis lack rigour and authors’ critical reflections on the study design and conduct are similarly weak. Quantitative analysis of survey data is limited to descriptive statistics and analysis of qualitative interview data is also descriptive and not theoretically-integrated. |
| SHARE | ^1^Wight et al. 2002  ^2^Wight et al. 1998  ^3^Wight & Buston 2003  ^4^Buston & Wight 2002  ^5^Buston et al. 2002 (CHS 4)  ^6^Buston & Wight 2004  ^7^Wight & Abraham 2000  ^8^Buston et al. 2001  ^9^Buston et al. 2002 (HER 17)  ^10^Buston & Hart 2001 | ^1^ Authors acknowledge that paper reflects the early stages of the investigation Limited information regarding implementation context, lack of detail regarding how process evaluation was conducted and how material was analysed. No primary data provided regarding the process evaluation, therefore unable to assess credibility of statements regarding delivery of the intervention  ^2^No detail provided regarding data collection or analysis, only limited primary data available to assess the credibility of claims made  ^3^Criteria for purposive sample (interviews and lesson observations) clearly described, but sampling strategy for survey not reported. Substantial detail provided about data collection and analysis. Findings clearly contextualised.  ^4^Small sample size acknowledged. However, clear criteria for purposive sample, in-depth and reflective description of how data was collected in an enquiring and sensitive manner, and full and reflective description of analysis process. Authors acknowledge that ‘the sample discussed in this paper substantially over-represents those who have had sex at an early age’  ^5^Purposive (maximum variety) sample drawn from large ‘population’ of schools in the trial, using interim findings (from survey) as a basis. Data collection and analysis fully described. Study designed and conducted to address issues identified in previous research (e.g. impact of classroom setting and teenagers’ gender roles)  ^6^Data collection and analysis described in greater detail in linked papers.  ^7^NA  ^8^Thorough data collection and analytic process; context in which programme was implemented richly described; candid reflection on limitations of findings.  ^9^Inclusive sampling strategy, with no teachers declining to be interviewed. Analysis of qualitative interview data somewhat limited by using a largely pre-defined framework, but good discussion of potential impact of teachers ‘over-selling’ fidelity of programme delivery to satisfy closely-involved researchers. Rigour of lesson forms questionable, as these were not validated and inconsistencies in teachers’ self-assessments were not addressed.  ^10^Strongly grounded in authors’ arguments that an assumption of heterosexuality is incorporated in many schools’ SRE, resulting in a neglect of gay and lesbian pupils’ needs. This provides the focus for the data analysed in this study, but was not a focus for the trial and qualitative research from which the data is drawn. Data collection was extensive, both in terms of spread across the 25 schools and data collection methods used (surveys, interviews, observations of practice). |
| Smoking and Me | Newman et al. (1991) | Significant weaknesses in reporting (scant or no information on study design, data collection and analysis) suggest that the overall study design and conduct was weak and that findings should be viewed with caution. |
| SPICED | Crosswaite et al. (2004) | Data collection and analysis processes not reported. Sampling strategy of schools and respondents not reported. Presentation of qualitative data is weakly-contextualised and largely descriptive. Connection between data and analysis frequently unclear. |
| UK Resilience Programme | Challen et al. (2009; 2011) | Whilst sampling strategy and data collection procedures are well-described, data analysis of process evaluation data is reported in very little detail even though there was extensive qualitative data from interviews. Findings are closely linked to examples from the data, but lack the richness that would be expected in a process evaluation of this scale. |
| Y-Active | Stathi & Sebire (2011) | While this study was well reported in terms of context, sampling, data collection, analysis, reflexivity and generalisability, it does reflect a single school: “The case study school used in the present research represents the most developed partnership, the outcome of approximately 5 years of collaboration between the YMCA and the school” (S240). On this basis its generalisability relating to the other Y-Active programmes is limited. |
