# Supplementary material for: Implementing health promotion programmes in schools: a realist systematic review of research and experience in the United Kingdom
Source: Implement Sci. 2015 Oct 28;10:149. doi: 10.1186/s13012-015-0338-6 (PMC4625879; doi:10.1186/s13012-015-0338-6)
Supplement: Additional file 8: — Data extraction tables. The file lists information on study type, the programme being evaluated, the content and delivery of the programme, and research methods (sample, participants, data collection and analysis) and evidence to enable testing of each of the four programme theories. (DOCX 24 kb) [file 13012_2015_338_MOESM8_ESM.docx]

| **HP Programme** |  |
| --- | --- |
| Name of programme | Active Primary School Pilot Programme |
| Area of HP | Physical activity (PA) |
| Authors (year/ study type) | Lowden et al. (2004/ BA process evaluation) |
| Quality appraisal | Unclear how pilot schools were selected, limited information regarding data collection (i.e. procedure/administration) and analysis. Very limited qualitative evidence provided to judge credibility of statements. Ethics and confidentiality not reported. |
| Programme description | ‘The Active Primary School Pilot Programme (APSPP) provides Co-ordinators to work with primary schools in order to increase the range and quality of opportunities for primary-aged children to become more physically active. It approaches these aims by providing relevant opportunities (play, sport and recreation, physical education, and active travel) both within and outwith the formal school curriculum.’ (p.1) Developed in response to the *Youth Sport Strategy for Scotland* (1996). Aimed to involve teachers, parents, the school board and members of the Parent Teacher Association, and ‘where appropriate, partnerships with other agencies and health initiatives are also pursued’ (p.1). Co-ordinators’ work was aimed at facilitating teachers to incorporate a range of physical activities (sports, games, dance, skill development) into the curriculum and ‘encouraging physical activity to feature in all schools’ development planning’. (p.1) |
| Year(s) delivered | Since 2000 |
| Description of UK location(s) where delivered | 21 local authorities (pilot study conducted in 11)  East Lothian (n=3)  Inverclyde (n=2)  Perth and Kinross (n=2)  Stirling (n=2)  West Lothian (n=2)  ‘In each year of the evaluation the age and sex distribution of pupils remained consistent across schools’ (p.6)  Free School Meal Entitlement (FSME) P5-P7 pupils - Table 4 |
| **Research methods** |  |
| Theoretical approach | NR |
| Sample | All co-ordinators, headteachers, and Local Authority representatives from the evaluated schools |
| Participants | Co-ordinators, headteachers, and Local Authority representatives |
| Data collection | 21 local authorities (pilot study conducted in 11)  - Monitoring forms (APSPP co-ordinators (n=5 per year), classroom teachers (n=11 per year), headteachers (n=36 per year (across all the co-ordinators schools)) - information on policies, practices, developments and features pertinent to the promotion of physical activity across the Programme schools  - Interviews – telephone, face-to-face, focus groups (co-ordinators (n=5 per year), headteachers (n=11 per year), local authority representatives (n=5 per year)) - perceptions of how the Programme had been working, along with any contextual insights |
| Analysis | Statistical methods NR, frequency / descriptive findings noted throughout |
| Time of follow-up | NR |
| **Evidence about programme theory#** |  |
| **1 - Preparing for implementation** | **Preparation for the introduction of a health promotion programme to a school is more likely to be successful when systematically planned in conjunction with other school responsibilities.** This involves: |
| 1a | Consultation with stakeholders, which involves:   - providing information on the programme (e.g. who and what is involved, evidence of effectiveness) - eliciting views on ‘readiness for change’ as a starting point for engagement - encouraging the sharing of previous experiences and knowledge of delivering health promotion programmes   ‘Our qualitative information highlights important factors and measures in promoting pupils’ physical activity in the face of such challenges [local levels of social deprivation, parental attitudes, access to PA opportunities, geographical remoteness]…Key factors include:   - Schools having had a history of local authority and school management interest and involvement in developing physical activity measures and related policies…those schools showing notable increases in pupils’ levels of physical activity have headteachers and local authority management that are particularly active in their support for the Programme. They are often involved in wider health developments such as the Health Promoting School and the Integrated Community School initiatives - Related to the above, such schools are characterised by networks and partnerships that support and facilitate efforts to promote pupils’ physical activity and health.’ (p.9) - facilitating discussion about the concordance (‘fit’) of the programme with:   - current practice  - pupils’ interests  - current school policies, resources and organisation  ‘Since 2001 there has been a reduction in the proportion of schools which deploy teachers to provide extended curricular activities (70% in 2001, 60% in 2002 and 48% in 2003). Headteachers and local authority managers stressed that other demands on teachers’ time limited their involvement in supporting extended curricular provision. The reduction in teachers’ involvement appears to bear this out but also reflects the Co-ordinators’ efforts to encourage the use of volunteers and local coaches’ (p.16)  Inhibiting factors:  ‘Other demands and priorities, such as curricular pressures and national testing, could limit the time available for planning and input for physical activity. Teachers and local authority managers, in particular, noted that even though the programme was increasing teachers’ awareness about pupils’ physical activity, pressures from the curriculum, planning timetables, and teachers’ other responsibilities could affect whether physical activity featured in a school’s development plan. Such pressures could also affect the extent to which teachers could actively support the Programme. Some pointed out that development planning usually occurs one year in advance and this could limit the speed at which developments could be implemented.  The extent and quality of resources and facilities for physical activity eg small and shared sports hall facilities; lack of equipment; poor quality playing fields and lack of transport. |
| 1b | Identification of a potential health benefit for pupils at a local level  NR |
| 1c | Consultation with stakeholders (head-teacher, school staff, pupils, parents, governors)  NR |
| 1d | Consideration of the concordance of the programme with current practice and interests  ‘One local authority manager stressed that it was important that Co-ordinators and authorities developed opportunities for physical activity that were sustainable. To this end, in this authority only those activities were developed which could utilise the existing and projected local resources, facilities, personnel/skills and venues.’ (p.34)  Headteachers identified the way in which co-ordinators ‘filled a skills gap’ by providing or co-ordinating a range of activities that schools did not traditionally provide, but which appealed to pupils. Headteachers perceived teachers as responding positively to the coaching and training provided to them by co-ordinators, although it was also suggested by some headteachers that this required ‘challenging stereotypes and promoting positive attitudes concerning access to physical activity’ (p.34). |
| 1e | Identifying clear aims and priorities, including intended outcomes  NR |
| 1f | Taking into account the current situation and competencies in a school and the implications of these for programme refinement  Inhibiting factors:  Fear of litigation in some cases has meant some schools were seen as ‘overly cautious’ about promoting pupils’ physical activity. For example, some were reluctant to let children play outside on surfaces that might not be ideal but were not dangerous.‘ (p.39-40) |
| **2 - Introducing a programme** | **The introduction of a health promotion programme to a school is more likely to be successful when it is incorporated into school activities through:** |
| 2a | Being integrated into school policy (e.g. a School Improvement Plan) and supported by governors and senior staff to whom monitoring and progress reports are made  At the start the evaluation (2001), the findings from the headteachers’ monitoring forms revealed that physical activity had yet to figure strongly in schools’ development planning. Only 23 per cent of the schools’ development plans featured the promotion of physical activity. By 2003 the number of schools that included promoting pupils’ physical activity in their development plans had risen to 60 per cent. While headteachers reported that the APSPP had raised awareness of the importance of physical activity in school planning, other factors such as national and local policies were also important. A similar increase occurred concerning the proportion of schools that included promoting physical activity outside of the timetabled curriculum (from 18% in 2001 to 53% in 2003).  There has also been an increase in the extent to which schools possess an inclusive policy to encourage all pupils to participate in physical activity (from 53% in 2001 to 93% in 2003)…The additional qualitative information gathered each year provided insights that further demonstrated that the Active Primary School Programme was largely responsible for such developments rather than other factors’ (p.21-22)  ‘By 2003 the local authority managers reported that the Programme had largely been integrated into the strategic planning for health and physical activity.’ (p.34)  ‘There is also greater cohesion and structure in schools’ extended curriculum physical activity developments’ (p.36) |
| 2b | School staff using their leadership skills to co-ordinate activities or resources for programme delivery  ‘There has been an increase in the proportion of schools that liaise with local authorities’ planning and transport departments to develop safe walking and cycling routes (from 18% in 2001 to 33% in 2003). There has also been an increase in the number of schools providing information to children and their parents about active travel (from 29% in 2001 to 40% in 2003).’ (p.261)  ‘…headteachers emphasised that often it was the role and input of the APSPP Co-ordinator that stimulated action in schools at policy and other levels…the findings strongly suggest that APSPP Co-ordinators are having an impact on schools’ capacity to promote pupils’ physical activity. The qualitative information highlights that this is especially the case when they have the support of local authority managers, headteachers and other key personnel.’ (p.31)  ‘Local authority managers frequently stressed that one of the main benefits of the Programme was that it had put in place full-time Co-ordinators who were supporting schools at cluster-level. It was felt that without such facilitators most schools, even those most committed to promoting physical activity, would not be able to fulfil that potential’ (p.34) |
| 2c | Providing adequate opportunity and/or training for the personal and professional development of those who will deliver the programme and engaging with their interests  ‘The vast majority of Co-ordinators’ schools in the pilot clusters (around 90% of schools) allocated funding to support staff to undertake relevant training courses…Overall, training for parent-volunteers and teachers has increased during the pilot. In 2002 and 2003 all five Co-ordinators reported facilitating training opportunities for teachers and volunteers across their schools, as well as providing broader support’ (p.22)  ‘Increased availability of coaches to support extended curriculum and curricular physical activity. Headteachers valued the efforts of the Co-ordinators in arranging such coaching and facilitating training for teachers and others to help sustain those physical activities that were in demand from pupils following ‘taster sessions’.’ (p.33)  ‘Co-ordinators and some managers believed that it was easier to enlist support for the Programme in schools if it entailed little or no additional work for headteachers and teachers, and if children were not removed from regular lessons.’ (p.39) |
| 2d | Modes of delivery that appeal to and engage pupils and provide cognitive and/or emotional rewards  ‘…it appears that the reason for the increase in pupils’ break/playtime activity is that the APSPP Co-ordinators and school management have worked closely to promote playtime activities. For example, training classroom assistants to promote active play, development of the playground environment, and providing resources to facilitate greater pupil activity.’ (p.12)  ‘Headteachers believed that this [ability to promote pupils’ PA] helped to promote pupils’ interest in physical activity by highlighting the variety of ways in which they could enjoy being active ... headteachers saw the Co-ordinators as helping to provide ‘taster sessions’ of different types of activities that stimulated pupils’ interest in physical activity. This approach meant that pupils who would not normally have participated in physical activity had been able to access activities ... Some headteachers believed that the ethos of the Programme meant that physical activity and sport were promoted in a non-competitive way, thus further enhancing the inclusive nature of the activities. Others commented on how the Programme had helped their schools to facilitate greater access to physical activity by disabled pupils’’ (p.32-33) |
| 2e | Providing support materials that are appealing and appropriate to pupils’ age, interests and culture  ‘Over the past three years, around 90 per cent of Co-ordinators’ schools have consistently provided joint physical activity events, festivals, and shared coaching. The proportion of schools that reported having links in place with sports clubs and other organisations that promoted physical activity increased from 59 per cent in 2001 to 65 per cent in 2002, but then decreased in 2003 to 53 per cent.’ (p.27)  ‘Increased access to physical activity and sports equipment. Headteachers generally believed that this helped schools promote a greater range of physical activity and was especially important in those areas where access to external leisure and sports venues was limited. However, one headteacher stressed that this equipment also had to be suited to the facilities the school already possessed. For example, if a school had limited outdoor playing areas or grass areas were regularly waterlogged then equipment such as cricket sets could be of limited use’ (p.33)  ‘Other factors include…arranging transport…challenging stereotypes…increasing pupil ownership (p.34) |
| **3 – Embedding a programme into routine practice** | **The introduction and routine delivery (‘embedding’) of a programme takes time and motivation. It is likely to involve changes in the school environment and the development of new relationships between stakeholders that require pro-active management so that:** |
| 3a | - different stakeholders’ goals are reconciled - organisational decisions in other areas of school life are made taking into account how they impact on programme delivery - school staffs’ existing relationships with children are built upon   LA managers perceived the ‘successes’ of co-ordinators’ training to have laid foundations for longer-term PA promotion by ‘increasing the range of personnel with skills available to schools to support promoting pupils’ physical activity (e.g. coaches, playground supervisors, classroom assistants, nursery nurses, parents, volunteers)’ (p.35)   - stakeholders’ enthusiasm, knowledge and experience are harnessed - knowledge of ‘core’ and ‘peripheral’ elements and minimum resources, skills and informational content is retained   ‘There was consensus that the sustainability of the Programme would require continued financial support in order to provide and maintain the necessary level of Co-ordinators’ (p.40)   - responsibility for programme delivery becomes rooted in the school   ‘…the Co-ordinators had worked with schools and authorities to develop infrastructures to help sustain the impact of the Programme’ (p.38)  LA managers perceived the longer-term strength of the PA programme to be less about being rooted in a school and more about developing schools’ networking with other bodies that could support the promotion of PA, e.g. LA departments (health, culture, sport, leisure), NHS health promotion staff, governing bodies, local clubs. (summary only, no supporting data) |
| 3b | The longer-term sustainability of programmes and the extent to which health promoting messages and activities permeate other aspects of school life, is dependent on continuing feedback, encouragement, and expectations about implementation. Over time, this may originate less from outside the school and more from inside.  ‘The APSPP Co-ordinators had ensured that playground games were sustainable by implementing training for teachers and classroom assistants. The Programme had also supported this development by facilitating the provision of playground markings and suitable equipment.’ (p.33) |
| **4 - Programme adaptation** | **The preparation for, introduction, initial delivery, and ongoing sustainability of a health promotion programme in a school is more likely to be successful when there is:** |
| 4a | Specificity about ‘core’ (essential) and ‘peripheral’ (optional/adaptable) programme elements, including the minimum levels of resources and/or skills necessary to support these elements (to inform decision-making about how to deliver a programme in the context of a particular school)  NR |
| 4b | Scope for ‘mutual adaptation’ between the programme and the people delivering it, including the evolution and updating of programme content and mode of delivery over time  ‘These Co-ordinators recognised the need for the Programme model to be flexible to meet local needs…’ (p.41) |

| **HP Programme** |  |
| --- | --- |
| Name of programme | APPLES |
| Area of HP | Obesity |
| Authors (year/ study type) | Sahota et al. (2001/ CRCT with process evaluation) |
| Quality appraisal | No detail regarding the context of how the programme was implemented and a lack of description regarding focus group attendance, as well as how information and data were analysed. No primary data about the implementation process reported (only authors’ summary). |
| Programme description | ‘A multidisciplinary, multiagency programme using a population approach. The programme team includes a dietitian, community paediatrician, health promotion specialist, psychologist, obesity physician and nutritional epidemiologist. The programme targeted the whole school community including parents, teachers, catering staff and the school environment. It was designed to take place over one academic year and was based on the concept of school action plans, which were to be developed by the individual schools on the basis of their perceived needs. The programme was intended to influence dietary and physical activity behaviour and not simply knowledge in the school children. The team provided training for teachers and some resources, and the project manager also provided input and contracted the schools regularly throughout the year to give support. The underlying approach was to be non-prescriptive and to ensure ownership of the programme by the schools’ (p.1-2). Programme implementation was supported by a project manager who provided ‘direct input’ to programme activities in schools. |
| Year(s) delivered | September 1996 – July 1997 |
| Description of UK location(s) where delivered | ‘10 state primary schools in Leeds. Sociodemographic measures suggested that they were somewhat advantaged schools with 1-42% of children from ethnic minorities and 7-29% entitled to free school meals (compared with 11% and 25% for Leeds children as a whole)’ (p.2) |
| **Research methods** |  |
| Theoretical approach | NR |
| Sample | All teachers (19 of the 20 who were eligible) who attended programme training |
| Participants | Teachers |
| Data collection | Survey (n=19; teachers) |
| Analysis | NR |
| Time of follow-up | At the end of school year in which delivered |
| **Evidence about programme theory#** |  |
| **1 - Preparing for implementation** | **Preparation for the introduction of a health promotion programme to a school is more likely to be successful when systematically planned in conjunction with other school responsibilities.** This involves: |
| 1a | Consultation with stakeholders, which involves:   - providing information on the programme (e.g. who and what is involved, evidence of effectiveness) - eliciting views on ‘readiness for change’ as a starting point for engagement   Programme design and development was informed by the results of a survey of ‘all school staff, including secretarial and catering staff, and parents of year 4 and 5 pupils’ (p.2) (school staff n=124; parents n=410 (64% response rate) showing high levels of agreement with the following statements (i.e. indicating acknowledgement of the importance of healthy eating) (%=agree with statement):  - It is important for schools to take a major role in promoting the health of children (school staff 88%; parents 92%)  - There should be an emphasis on teaching about balanced eating and physical activity in school (school staff 94%; parents 94%)  - Schools should have a food policy (school staff 67%; parents 70%)  - The family alone should not be responsible for their child’s food habits and physical activity (school staff 97%; parents 80%)  - Schools should be responsible for encouraging physical activity (school staff 87%; parents 95%)  - A child’s diet affects its health (school staff 91%; parents 91%)  - A child’s diet affects its health in adulthood (school staff 88%; parents 85%)  The survey was also used to find out from parents ‘what changes they would like to see in school and information they would like to receive. The responses were used by schools to develop the school action plans… [and] progress towards these targets was monitored by regular staff meetings and surveys of packed lunches, breaktime snacks, and playground activities.’ (p.2). Also, ‘feedback to the survey and changes within the school were circulated to parents during the year.’ (p.2)   - encouraging the sharing of previous experiences and knowledge of delivering health promotion programmes - facilitating discussion about the concordance (‘fit’) of the programme with:   - current practice  - pupils’ interests  - current school policies, resources and organisation |
| 1b | Identification of a potential health benefit for pupils at a local level  NR |
| 1c | Consultation with stakeholders (head-teacher, school staff, pupils, parents, governors)  NR |
| 1d | Consideration of the concordance of the programme with current practice and interests  NR |
| 1e | Identifying clear aims and priorities, including intended outcomes  NR |
| 1f | Taking into account the current situation and competencies in a school and the implications of these for programme refinement  NR |
| **2 - Introducing a programme** | **The introduction of a health promotion programme to a school is more likely to be successful when it is incorporated into school activities through:** |
| 2a | Being integrated into school policy (e.g. a School Improvement Plan) and supported by governors and senior staff to whom monitoring and progress reports are made  ‘All schools incorporated nutrition education in the curriculum [and] included a “fit is fun” programme in physical education lessons and undertook to improve their health resources.’ (p.3). Across all ten schools, 76 of the 85 (89%) actions points in school action plans were achieved. (but no further details provided about the extent or depth of these action points) |
| 2b | School staff using their leadership skills to co-ordinate activities or resources for programme delivery  ‘Reasons for not achieving action points [within school action plan] included shortage of time, staff sickness, and impending inspection by government teaching standards officers.’ (p.3) |
| 2c | Providing adequate opportunity and/or training for the personal and professional development of those who will deliver the programme and engaging with their interests  ‘[Teachers] found the training useful, that the resources were useful and they would continue to use them’ (p.2) |
| 2d | Modes of delivery that appeal to and engage pupils and provide cognitive and/or emotional rewards  NR |
| 2e | Providing support materials that are appealing and appropriate to pupils’ age, interests and culture  ‘The project manager offered a variety of ways that she could support the schools in implementing their action plans.’ (p.3) E.g. class sessions on healthy eating, competitions, and involvement in food awareness week were taken up by all ten schools. (Less popular support was attendance at parents’ evenings and open days and ‘health weeks’, taken up by only four schools). |
| **3 – Embedding a programme into routine practice** | **The introduction and routine delivery (‘embedding’) of a programme takes time. It is likely to involve changes in the school environment and the development of new relationships between stakeholders that require pro-active management so that:** |
| 3a | - different stakeholders’ goals are reconciled - organisational decisions in other areas of school life are made taking into account how they impact on programme delivery - school staffs’ existing relationships with children are built upon - stakeholders’ enthusiasm, knowledge and experience are harnessed - knowledge of ‘core’ and ‘peripheral’ elements and minimum resources, skills and informational content is retained - responsibility for programme delivery becomes rooted in the school   NR |
| 3b | The longer-term sustainability of programmes and the extent to which health promoting messages and activities permeate other aspects of school life, is dependent on continuing feedback, encouragement, and expectations about implementation. Over time, this may originate less from outside the school and more from inside.  NR |
| **4 - Programme adaptation** | **The preparation for, introduction, initial delivery, and ongoing sustainability of a health promotion programme in a school is more likely to be successful when there is:** |
| 4a | Specificity about ‘core’ (essential) and ‘peripheral’ (optional/adaptable) programme elements, including the minimum levels of resources and/or skills necessary to support these elements (to inform decision-making about how to deliver a programme in the context of a particular school)  NR |
| 4b | Scope for ‘mutual adaptation’ between the programme and the people delivering it, including the evolution and updating of programme content and mode of delivery over time  NR |

| **HP Programme** |  |
| --- | --- |
| Name of programme | A Stop Smoking in Schools Trial (ASSIST) |
| Area of HP | Substance use (smoking) |
| Authors (year/ study type) | ^1^Audrey et al. 2004/programme development  ^2^Audrey et al. 2006 (HER 21)/programme description  ^3^Audrey et al. 2006 (SSM 63)/process evaluation  ^4^Audrey et al. 2008/process evaluation  ^5^Holliday et al. 2009/process evaluation |
| Quality appraisal | ^1^Research methods reported in linked papers  ^2^Description of programme development, critical appraisal tool not applicable  ^3^Data collection and analysis thoroughly described and conducted; in-depth interviews appropriate to explore implementation issues. Reasonable level of contextual detail provided.  ^4^Data collection and analysis thoroughly described and conducted; in-depth interviews appropriate to explore implementation issues. Reasonable level of contextual detail provided.  ^5^Limitations in sampling strategy re: how schools were selected for exploration of implementation issues (and how the schools’ characteristics may have impacted on implementation). Data collection not adequately described, but data analysis thoroughly reported and conducted. Candid reflection on the potential impact of the fact that peer trainers and researchers shared office space may have had on implementation processes. |
| Programme description | The ASSIST intervention model was based on: recruitment of influential students who were nominated by their peers, and implementation by external trainers rather than school teaching staff. Based on diffusion of innovation theory (how ideas and practices spread within communities through personal interaction), ‘influential’ students were trained to become ‘peer supporters’ with regard to reducing/stopping smoking amongst their peers. The role of peer supporters was to provide health information and advice, support peers and act as role models.  Influential students were identified via a survey of Year 8 students, and invited to take on the role. Training (2 days, off school premises) was a ‘student-centred experiential process’ which had been developed by a team of health promotion specialists, teachers and youth trainers. It was designed to increase peer supporters’ knowledge about smoking health risks, improve their communication skills and contribute to their personal development. Post-training programme visits were made to peer supporters and one, four and seven weeks so as to offer support, resolve problems, and provide an opportunity to consolidate their skills and learning.  (Audrey et al. 2004; Audrey et al. 2006 HER 21; Audrey et al. 2008) |
| Year(s) delivered | 2002 (10 weeks) |
| Description of UK location(s) where delivered | West England, south-east Wales  Audrey et al. (2006b)(Table 3) – 4 sampled interventions schools (n=year 8 students/% of free school meal entitlement)  South-east Wales n=269/4.8; n=130/25.9  West of England n=232/21.8; 159n=6  Audrey et al. (2008) (Table 3 number of schools within ASSIST trial (intervention & control) - state funded, fee-paying, comprehensive, independent, sex, denominational, welsh) |
| **Research methods** |  |
| Theoretical approach | NR |
| Sample | Various e.g. randomised sample of students for interview; ‘population’ sample of Year 8 students (survey); purposive sample of schools for process evaluation |
| Participants | Children aged 13-14y; Teachers and key staff, including ‘key programme contact’ teachers; ASSIST team members; health promotion trainers, youth workers |
| Data collection | Interviews (n=33) and focus groups (n=10) with peer supporters  Interviews (n=32) with Year 8 pupils in intervention schools who indicated they had conversations about smoking with peer supporters  Surveys of supervising teachers in intervention schools (re: peer-supporter recruitment n=27; re: peer-supporter training n=31)  Interviews with key programme contact teachers (n=19)  Interviews with health promotion trainers (n=11)  Observation of delivery of peer-support training and follow-up (varied between 2 and 6 sessions each at recruitment, training, and peer-support FU sessions #1-4)  Note; ‘Non-participant observation, interviews and focus groups were conducted in four intervention schools purposefully selected on the basis of location, size and level of deprivation.’ (Audrey et al. 2006 SSM 63; p.323) |
| Analysis | Preliminary coding framework developed to identify common themes (Audrey et al. (2006b) |
| Time of follow-up | Varied from immediately post-intervention to 10wks, as appropriate  Outcome data at 1-year follow-up (Audrey et al., 2006b), 2 year (Audrey et al. 2008) |
| **Evidence about programme theory#** |  |
| **1 - Preparing for implementation** | **Preparation for the introduction of a health promotion programme to a school is more likely to be successful when systematically planned in conjunction with other school responsibilities.** This involves: |
| 1a | Consultation with stakeholders, which involves:   - providing information on the programme (e.g. who and what is involved, evidence of effectiveness)   Schools were approached via a letter to the headteacher – when an interest to take part in the study was expressed, ASSIST staff visited ‘the senior member of the teaching staff responsible for co-ordination of the intervention’ (, p.272) *(no details on how schools identified this staff member or protected their project time)*. The aim of the visit was to ‘ensure that the school was fully aware of the level of commitment and organisation required’ and to negotiate and agree this commitment (p.272), i.e. researcher access to Year 8 students, communication with nominated students, time and support for peer-supporters to attend the off-site training and on-site follow-up visits from researchers. These school co-oridnators were ‘also responsible for liaison with teachers whose lessons would be disrupted by the withdrawal of the selected students’ (p.272). ASSIST staff’s roles (e.g. organisation of training events) were made clear and student safety issues clarified (e.g. all ASSIST trainers were police-vetted; risk assessments for training events). (Audrey et al. 2004). >50% of the schools approached to take part in the trial wanted to participate; no schools withdrew from the intervention (Audrey et al. 2008)  Some teachers were concerned about including “challenging pupils” as peer-supporters, e.g suggesting that some pupils were ‘not suitable to represent the school or that they did not deserve the privilege of participating in the intervention because of a history of truancy or disruptive behaviour… [but] despite some misgivings, the majority of school contacts recognised the importance of the peer nomination process and gave active support. This resulted in the recruitment of peer supporters who were balanced in terms of gender and broadly representative of their peer group.’ (Audrey et al. 2008, p.83)   - eliciting views on ‘readiness for change’ as a starting point for engagement - encouraging the sharing of previous experiences and knowledge of delivering health promotion programmes - facilitating discussion about the concordance (‘fit’) of the programme with:   - current practice  ‘Overall, teachers welcomed the fact that the training was delivered by external trainers, suggesting that it created additional interest amongst the students and acknowledging that students might experience difficulties in discussing smoking behaviour with teachers. Teachers also welcomed the use of outside trainers to relive the burden on teaching staff’ (Audrey et al. 2008, p. 84)  A number of teachers talked about how the programme was deliberately implemented in way designed to minimise conflict with other timetabled teaching and assessment:  “I would say that disruption has been minimal because that is how we tried to do it, that we weren’t hitting every maths group, for example, each time they came in for their training.” (Head of PHSE (Audrey et al. 2008, p.86))  “For this, as a year, it’s not so onerous for them. As staff, Year 8, there’s no pressure on it, whereas Year 9 we’ve got SATs to aim for, we’ve got mock SATs and so on, and obviously into Year 10 and 11 you’ve got GCSEs. So, it’s the better year for them to go for.” (Pastoral deputy head (Audrey et al. 2008, p.85))  However, others perceived that there was simply too much pressure on the school timetable:  “To rearrange, perhaps if there was a test on and they needed to be seen, and so on and so forth, does pose a problem for some pupils… It is making a comment about life in school at the moment. There seems to be one thing after another after another. Now that has an effect on staff and it certainly has an effect on pupils too.” (Acting head of religious studies) (Audrey et al. 2008, p.85)  - pupils’ interests  At the peer-supporters recruitment meeting, ‘the trainers explained that the skills of a peer-supporter could be transferred to other aspects of their school career and when seeking employment’ (Audrey et al. 2004, p.274)  96% of Year 8 students completed peer questionnaire  (Holliday et al. 2009)  90% (848/942) of students invited to the recruitment meeting completed the two-day training.  82% (687/835) of students who consented to act as peer-supporters were ‘judged to have undertaken the role of peer-supporter [10 weeks] and handed in their [reflective] diaries’  (Audrey et al. 2004, p.278)  89% of peer-supporters attended 3 or 4 follow-up sessions; 71% of peer-supporters handed in their reflective diary  (Holliday et al. 2009)  Other aspects of the school year (e.g. holidays) sometimes extended the time over which the programme was delivered (i.e. the timescale over which peer-supporters engaged with their peers and were in turn supported by the ASSIST team), which could impact on students’ ability to maintain their interest.  Peer-supporter attendance at number of follow-up sessions (p.54):  1 session – 1.4%  2 sessions – 6.9%  3 sessions – 22.4%  4 sessions – 66.9%  (Holliday et al. 2009, p.55).   - current school policies, resources and organisation   NR |
| 1b | Identification of a potential health benefit for pupils at a local level  ‘Student smoking was perceived to be a difficult issue and, because they were unclear how to address the problem effectively, staff appeared to welcome the opportunity to test a new initiative’ (Audrey et al. 2008, p.84):  “We discuss and we sort of run out of ideas really, when it comes to smokers on the field and to what degree punishing them, writing letters home, informing parents. All that stuff is a bit, it’s negative and not very effective. So to have an approach which is more thought out, and more involving kids, involving pupils being more active and spreading the message, yeah it’s good.” (Head of Year 8) (Audrey et al. 2008, p.85) |
| 1c | Consultation with stakeholders (head-teacher, school staff, pupils, parents, governors)  NR |
| 1d | Consideration of the concordance of the programme with current practice and interests  Data not reported about the number of schools where this applied, but the peer-supporter approach was noted by some teachers to fit very well with school ethos:  “It’s definitely the ethos of the school to let pupils take responsibility for themselves.” (Deputy Head of Year (Audrey et al. 2008, p.85))  “Health education might be the particular message but I think it fits into a much broader spectrum about education in general, about giving kids confidence with information to actually go and talk to other people and put forward a point of view.” (Guidance co-ordinator) (Audrey et al. 2008, p.85) |
| 1e | Identifying clear aims and priorities, including intended outcomes  NR |
| 1f | Taking into account the current situation and competencies in a school and the implications of these for programme refinement  NR |
| **2 - Introducing a programme** | **The introduction of a health promotion programme to a school is more likely to be successful when it is incorporated into school activities through:** |
| 2a | Being integrated into school policy (e.g. a School Improvement Plan) and supported by governors and senior staff to whom monitoring and progress reports are made  Venues for recruitment and follow-up sessions varied markedly in their suitability for large and small group discussion (pressure to adhere to the curriculum could also be a factor here):  “You suddenly get landed with a science lab, which meant that everybody was really spread out and they’re an unpleasant sort of environment to be in. It was sort of some schools thought it through as to ‘This room will be nice for you’, you know, ‘it will be a good atmosphere’. Others, it was obviously just whatever room happened to be free.” (Trainer)  (Holliday et al. 2009, p.52) |
| 2b | School staff using their leadership skills to co-ordinate activities or resources for programme delivery  The school co-ordinator/contact was identified as key for delivering the school-based sessions ‘to the intended schedule and in appropriate venues’:  “... at [school], she’s got so many other responsibilities in the school I know it has been hard for her to get messages round and although she has done it, often it has been quite late in the day and often we have had to ring up and remind her... and then you get the other extreme, you know, [teacher] at [school] everything is just so organised you know.” (Trainer)  (Holliday et al. 2009, p.56)  However, it was also recognised that these organisational/leadership skills could be significantly affected by wider factors (e.g. school ethos and organisation):  “Each follow-up visit has resulted in trailing around with the pupils and year head trying to find a vacant classroom. They are apparently not allowed to pre-book free classrooms!” (Trainer)  (Holliday et al. 2009, p.56) |
| 2c | Providing adequate opportunity and/or training for the personal and professional development of those who will deliver the programme and engaging with their interests  The varied backgrounds of the trainers (teachers, health promotion, youth workers) meant that there were ‘variations in expertise and differences in style, which on occasion caused conflict’, e.g.:  “He [trainer] could focus their attention very quickly and use personal anecdotes and things to actually get their attention and give them a measure of what was being wanted. But he lacked the basic understanding about the health risks of smoking which I would have said would be useful to trainers.” (Trainer)  “There were possibly two occasions when I felt the mix of trainers didn’t complement each other as well as they could have done and the styles clashed a little bit and trainers were uncomfortable about working with people.” (Trainer)  (Holliday et al. 2009, p.56)  Factors from process evaluation which are ‘suggested… to have contributed to the recruitment and retention of such a broad spectrum of young people [as peer-deliverers of the programme]’:  - pride in being nominated by their peers  - novelty of being taken out of school to be trained  - commitment to learning more about smoking and reducing smoking among their peers  - use of external trainers, rather than school teachers  - style and content of the programme  - being able to take ‘time off’ usual lessons to attend follow-up sessions  - possibility of receiving a gift voucher  (Audrey et al. 2006 SSM 63; p.325) |
| 2d | Modes of delivery that appeal to and engage pupils and provide cognitive and/or emotional rewards  Some (if not all) peer-supporters demonstrated subtlety in how they spoke with peers about smoking, e.g.:  “They didn’t ask you directly, you know, ‘do you smoke?’, ‘what do you think about smoking?’. They just kind of brought it into the conversation, which made people feel sort of more comfortable about talking about it. So I think that was good how they did it.” (female non-peer-supporter) (Audrey et al. 2006 SSM 63; p.325) |
| 2e | Providing support materials that are appealing and appropriate to pupils’ age, interests and culture  NR |
| **3 – Embedding a programme into routine practice** | **The introduction and routine delivery (‘embedding’) of a programme takes time and motivation. It is likely to involve changes in the school environment and the development of new relationships between stakeholders that require pro-active management so that:** |
| 3a | - different stakeholders’ goals are reconciled - organisational decisions in other areas of school life are made taking into account how they impact on programme delivery - school staffs’ existing relationships with children are built upon - stakeholders’ enthusiasm, knowledge and experience are harnessed - knowledge of ‘core’ and ‘peripheral’ elements and minimum resources, skills and informational content is retained - responsibility for programme delivery becomes rooted in the school   Peer-supporters’ ability to take part in follow-up sessions (a key part of the programme for addressing issues that arose and providing feedback) could be impacted by a lack of co-ordination with other aspects of the school day/curriculum, e.g.:  “I think school organisation and the lessons that were missed for the follow-up visits were very important. You know, some schools chose a variety of lessons [in which peer-supporters could take part in the follow-up], other ones only chose PHSE or PE and CDT and the kids really enjoy PE and CDT and they hated missing those... [School] was another one where twice at least they missed a PE lesson and CDT lesson and there was an absolute mutiny about whether they were going to stay or not for some of them.” (Trainer)  (Holliday et al. 2009, p.55) |
| 3b | The longer-term sustainability of programmes and the extent to which health promoting messages and activities permeate other aspects of school life, is dependent on continuing feedback, encouragement, and expectations about implementation. Over time, this may originate less from outside the school and more from inside.  Authors urge caution in interpreting short-term sustainability of the programme (towards the end of the 10-week intervention period) as pupils would not necessarily have known who the peer-supporters were – but results below show <25% had spoken about smoking in the ‘last few weeks’. (Audrey et al. 2006 SSM 63; p.329)    Peer-supporters found it progressively harder to initiate conversations about smoking (1^st^ FU- week 2; 4^th^ FU – week 10) (Audrey et al. 2006 SSM 63; p.329):    Similar findings from interviews with peer-supporters:  “Like we had two days off school [for the peer-supporter training] and then in the first week there was loads of people saying ‘oh, what did they teach you?’ and you had loads more conversations then’ (male peer-supporter) (Audrey et al. 2006 SSM 63; p.328)  “I ended up talking to the same people again. Not many of my friends smoked or anything so it was just like telling them the facts and they got bored in the end.” (female peer-supporter) (Audrey et al. 2006 SSM 63; p.328) |
| **4 - Programme adaptation** | **The preparation for, introduction, initial delivery, and ongoing sustainability of a health promotion programme in a school is more likely to be successful when there is:** |
| 4a | Specificity about ‘core’ (essential) and ‘peripheral’ (optional/adaptable) programme elements, including the minimum levels of resources and/or skills necessary to support these elements (to inform decision-making about how to deliver a programme in the context of a particular school)  Activities within the peer-supporter training were classified as red, amber or green. Red activities were essential, amber activities were ‘intended to consolidate skills or information already learned and which could be omitted’, and green activities were those which would be omitted first (these were activities aimed at keeping the training ‘fresh’ through mixing groups and running ‘games with a purpose’ when peer-supporters’ concentration waned) (Audrey et al. 2004, p.276) |
| 4b | Scope for ‘mutual adaptation’ between the programme and the people delivering it, including the evolution and updating of programme content and mode of delivery over time  Some schools had wider concerns than programme delivery, e.g. they did not want to ‘reward’ certain students (identified as ‘influential’ and therefore potential peer-supporters) for poor behaviour by allowing them to be trained for the programme:  “The four pupils the school wouldn’t let come were all influential, especially [pupil]. Apparently not allowed to come because it would be seen as a privilege. School didn’t want to be seen giving privileges to pupils who have behaved badly.” (Trainer)  .. or because they were concerned about the school’s image:  “I got the impression that some of the better schools were worried about reputation... so what they classed as bad kids were probably not at all, relative to other schools that were prepared to give them a chance.” (Trainer)  (Holliday et al. 2009, p.50)  Despite the red, amber, green classification of programme components, some trainers still experience a tension between the programme as designed and how they thought it should be delivered to a particular group:  “But because it was to measure the scientific outputs, it had to be done in the same way each time, you know, you just had to get on with it and do it, there were certain things that you couldn’t let drop. That inflexibility I found sort of hard, because then you were just delivering the programme regardless of the needs of the group.” (Trainer) (p.57)  Nevertheless, some of the flexibility of the programme was welcomed:  “You get stale if you have to deliver everything literally to the letter and it was nice to have a certain amount of flexibility to be able to do things differently... I wouldn’t say that anything was done hugely differently. It just comes down to personality and the style of the trainers ultimately as to how it’s delivered.” (Trainer) (p.57)  (Holliday et al. 2009) |

| **HP Programme** |  |
| --- | --- |
| Name of programme | Blueprint |
| Area of HP | Substance use (legal & illegal drugs) |
| Authors (year/ study type) | ^1^Stead et al. D&AR (2007/ process evaluation) - fidelity of component delivery  ^2^Stead et al. (2007/ CBA process evaluation) – delivery  ^3^Blueprint Evaluation Team (2007/ evaluation) – pupils and parents response to Blueprint |
| Quality appraisal | ^1^Limited information regarding school settings, lack of detail regarding data collection (i.e. administration, type, procedure) and analyses (i.e. how conducted, supplementary forms of evidence used)  ^2^Although report is in-depth and contains extensive details about implementation issues, there are significant weaknesses in the reporting of research methods, e.g. sampling strategy for survey, interviews and lesson observation not reported; data analysis processes not reported. Link between data and reported findings often unclear. No reflection on methodological limitations and their potential impact on findings.  ^3^Mostly survey-based material so depth is limited in relation to implementation. |
| Programme description | Developed over 3 years (led by Home Office ‘in partnership with’ Dept.of Health and Dept. for Education and Skills). Rather than adapting existing programmes to the English context, the programme was ‘based on the distillation of key principles of effective drug education rather than relying on any one programme model, although it was particularly informed by Project STAR and Life Skills Training.’ (p.654) (and Dusenbury & Falco’s (1995) principles of effective drug education) (Stead D&AR 2007). Lesson materials and plans were piloted. Programme consisted of five connected drug prevention components delivered in 23 schools:  1) Schools – curriculum (15 lessons, approx. 50 minutes per lesson) delivered over 2y with a normative focus (‘demonstrating to young people that both actual rates of drug use, and approval of drug use, are lower than they think, and examines social influence factors within peer networks and through the media’ (p.654) (Stead D&AR 2007). Supporting resources were provided for pupils, parents, teachers and school governors. Teachers received a total of 6 days of training and funding was provided to extend the role of School Drug Advisers.  2) Parents – series of 6 parenting skill workshops (bullying, relationships and talking about drugs)  3) Media – ‘A media relations agency was contracted to generate news coverage about Blueprint throughout the study and to co-ordinate stakeholder communications.’ (p.655-6) (Stead D&AR 2007)  4) Health policy – Local Authorities in the four Blueprint areas received additional funding to increase work in retailer education and training, youth education, proof-of-age schemes, test purchasing, prosecutions, and media communications.  5) Community – activities were ‘developed to involve the wider community of drug professionals and organisations’ (p.656) (Stead D&AR 2007) |
| Year(s) delivered | 2003-2005 |
| Description of UK location(s) where delivered | ‘Mixed sex comprehensive schools within England with a named health or drug education co-ordinator and an established drug policy, engaged in the National Healthy School Standard programme and having long-term involvement with promoting a whole-school approach to health and wellbeing’ (Stead D&AR p. 656)  Location north-west/midlands area. Each area contained two comparison group schools - one of relatively low and one of relatively high score for Index of Multiple Deprivation (Stead, 2007) |
| **Research methods** |  |
| Theoretical approach | NR |
| Sample | ^1^‘The intention was to observe eight teachers delivering all 15 Blueprint lessons (longitudinal observations) and then to sample lessons delivered by a minimum of three other teachers in each of the remaining intervention schools (cross-sectional observations). In all 74 teachers were observed delivering 320 Blueprint lessons’ (p.659)  ^2^Various (p.32-35) i.e. Lifestyle survey: ‘retrospective sample at each survey wave’ (p.32); qualitative research with young people: ‘the sample was drawn from classes not participating in the Lifestyle and Impact surveys’ (p.33)  ^3^Various (p.15-16) i.e. Prevalence survey: ‘retrospective sample at each survey wave’ (p.15); parents survey: ‘A one-off postal survey was conducted in May-July 2005 of all parents/carers in Blueprint’ (p.15) |
| Participants | Pupils aged 11-13yrs/ parents, teachers, drug prevention professionals |
| Data collection | ^1^Observation of Blueprint lessons (adherence to content, activities, use of support materials) (n=74 teachers observed over 320 lessons)  Interviews with teachers, pupils, School Drug Advisors supporting Blueprint delivery (n=NR)  Impact survey – pupils’ reactions to Blueprint (n=NR)  ^2,3^Survey:  - young peoples reactions to each year of the programme, communication with parents about drugs/drug use mediating variables. In comparison schools – young people’s response to drug education/prevention activities (n=2779/ yr2003; n=2751/ yr2005; n=2745/ yr2006)  - parents (n=NR) – parents/carers reactions to and involvement in Blueprint, also communication with children about drugs  - Interviews:  Pupils (n=48) – exposure to and experiences with drugs, perceptions and experiences of Blueprint, and how they discussed drugs and Blueprint with significant others, including parents and friends  Parents (n=25) - involvement in, and reaction to the Blueprint activities and the quality and nature of communication between parents and their child(ren) about the Blueprint programme and about drug issues in general  - Classroom delivery evaluation:  Classroom (n=266 Blueprint lessons) - fidelity of delivery to the approaches specified in the teacher manuals and teacher training programme. Comparisons (n=54 drug education/PSHE lessons). Complemented by teacher interviews and sample teacher logs  - Teacher training evaluation:  Extent to which the training equipped teachers to deliver the Blueprint programme in schools. A range of methods was used - observation of teacher and School Drug Advisors (SDA) training; self-completion training questionnaires completed by training participants. teacher survey (n=175 Blueprint-trained teachers). Qualitative interviews (teacher/headteachers, n=41)- to explore teachers’ experiences in more depth  - Community case studies:  Focused on the support provided by SDAs involved with Blueprint delivery. Interviews conducted with SDAs, who also kept a log of Blueprint support activities |
| Analysis | ^1,2^NR  ^3^Surveys analysed at cross-sectional level (analysis of variance – ANOVA) |
| Time of follow-up | During delivery of programme |
| **Evidence about programme theory#** |  |
| **1 - Preparing for implementation** | **Preparation for the introduction of a health promotion programme to a school is more likely to be successful when systematically planned in conjunction with other school responsibilities.** This involves: |
| 1a | Consultation with stakeholders, which involves:   - providing information on the programme (e.g. who and what is involved, evidence of effectiveness)   ‘There was a period of substantial groundwork prior to the delivery of the Blueprint lessons in schools. The contractor team spent considerable time communicating with schools to ensure that they understood what would be required of them in participating in Blueprint. In addition, a member of the School Component Contractor team presented the project to a whole-staff meeting at each Blueprint school. In an effort to facilitate and streamline Blueprint-related communications, each school was asked to identify a ‘Blueprint Coordinator’ who would act as the main liaison between the school and the School Component Contractor (and other Blueprint contractors). This was often a PSHE specialist or Head of Year, although sometimes the Head or Deputy Head took on the role.’ (Stead et al. 2007, p.66-67)   - eliciting views on ‘readiness for change’ as a starting point for engagement - encouraging the sharing of previous experiences and knowledge of delivering health promotion programmes - facilitating discussion about the concordance (‘fit’) of the programme with:   - current practice  - pupils’ interests  - current school policies, resources and organisation  Although details of consultation are not provided, reports average level of attendance at Blueprint lessons of 95% and that ‘all [74] of the observed teachers had received the pre-programme training.’ (Stead et al. 2007 D&AR, p.660). |
| 1b | Identification of a potential health benefit for pupils at a local level  NR |
| 1c | Consultation with stakeholders (head-teacher, school staff, pupils, parents, governors)  Although details of consultation are not provided, parents largely rated the programme positively:  ‘Pleased my child had these drug lessons’ 92%  ‘The lessons are suitable for my child’s age’ 85%  ‘The lessons taught my child something new’ 86%  ‘The lessons at my child’s school are good’ 76% (Blueprint Evaluation Team 2007) |
| 1d | Consideration of the concordance of the programme with current practice and interests  NR |
| 1e | Identifying clear aims and priorities, including intended outcomes  NR |
| 1f | Taking into account the current situation and competencies in a school and the implications of these for programme refinement  NR |
| **2 - Introducing a programme** | **The introduction of a health promotion programme to a school is more likely to be successful when it is incorporated into school activities through:** |
| 2a | Being integrated into school policy (e.g. a School Improvement Plan) and supported by governors and senior staff to whom monitoring and progress reports are made  NR |
| 2b | School staff using their leadership skills to co-ordinate activities or resources for programme delivery  NR |
| 2c | Providing adequate opportunity and/or training for the personal and professional development of those who will deliver the programme and engaging with their interests  ‘it appeared some teachers did not understand clearly the principles behind some of these approaches, particularly normative education and minimisation of harm. For example, the year 8 lessons used the scenario of a party to highlight potential risks and develop strategies for their minimisation. While the majority of teachers and pupils seemed to understand this approach, a sizeable minority tended to adopt a more simplistic and unrealistic prohibitive position of ‘Don’t drink alcohol, don’t smoke and don’t take drugs!’’ (Stead D&AR 2007, p.660)  Concerns raised by teachers about programme delivery:  - classroom management and control when delivering the interactive activities (especially among new teachers)  - being uncomfortable with the employment of interactive learning approaches because of the potential for disruption and unpredictable outcomes (more experienced teachers).  - not being able to answer pupils’ questions about drugs.’ (Stead D&AR 2007, p.661)  ‘Several teachers felt they would not have been able to deliver the programme without the training’ (materials alone would have been insufficient preparation). At the end of the second day of training, 81% (yr 7) & 93% (yr 8) felt it had been a good opportunity for practising the lessons, 97% felt that practising the lessons had been useful (p.48). (Stead et al. 2007)  Some teachers found the movement into personal issues and affective or pastoral care most challenging. It was also acknowledged that the lessons required a high level of preparation. Training also provided an opportunity for interaction, networking, reassurance and validation from sharing experiences, 99% of those trained in Year 8 rated the chance for informal conversation with other teachers as very or fairly useful (p.56) (Stead et al. 2007)  Benefits of Review Days were to reflect and interact with colleagues:   - 89% (Year 7) agreed “it was a good opportunity to see how their experiences compared with others (85% Year 8) - 67% (Year 7) “it was a good opportunity to reflect on how I delivered the lessons” (68% Year 8)   “...views tended to be less positive about the review days than about the training overall” (p. 59). 61% (Year 7) 59% (Year 8) said the review days had been a good use of time, 1 in 10 teachers in both years said it had been a “waste of time”. Only ‘(33%) of Year 7 participants felt that the Year 7 review day helped them plan for Blueprint in Year 8, and the same proportion of Year 8 participants felt that the Year 8 review day helped them plan for future drug education in school’ (p. 60). (Stead et al. 2007)  Some co-ordinators felt that non-PHSE teachers who were ‘unfamiliar with interactive learning approaches’ (which they were taught in the Blueprint training programme) could not be expected to use these techniques in programme delivery – however, observation of Blueprint delivery found ‘good quality teaching by form tutors with little previous experience of active learning methods, as well as by experienced PHSE specialist teams’ (Stead et al. 2007, p.92) |
| 2d | Modes of delivery that appeal to and engage pupils and provide cognitive and/or emotional rewards  ‘The proportion of observed teachers who consistently met or exceeded 75% fidelity [for lesson delivery] was highest for those activities that involved teacher – pupil interactions in whole-class settings and lowest for those activities which predominantly involved pupil – pupil interaction. Some of the teachers, when interviewed, emphasised that this was because the pupil – pupil interactions could be rather unpredictable and difficult to control and, as a result, they sometimes intervened more than the lesson plan specified’ (Stead D&AR, p.659)  ‘In year 7, three-quarters or more of the pupils were rated, in the observations, as actively participating in 79% of the learning activities, rising to 82% in year 8. Pupils reported in the Impact Survey that their highest levels of participation were in those learning activities which were highly interactive. Levels of participation tended to be lower for whole class discussions led by the teacher (see Table 5)’ (Stead D&AR 2007, p.661)  All of the active learning methods (role play, pupil presentations, group work, party scenario, quiz and class discussions) were rated as ‘enjoyable’ by >=50% of pupils, with the more active methods (role play and pupil presentations) being rated as ‘enjoyable by >80% of pupils. The most positively rated aspects were:  • having believed what the lessons told them (84% in Y7, 78% in Y8);  • having learnt something new (80% in Y7, 74% in Y8);  • having paid attention to the lessons (70% in Y7, 60% in Y8);  • having found them easy to understand (69% in Y7, 70% in Y8);  • having found them useful (66% in Y8);  • having found them a good preparation for real life (67% in Y8);  • having covered the things people their age need to know (71% in Y8).  (Blueprint Evaluation Team 2007)  ‘Pupil participation in most Blueprint lessons was high. It was particularly high in those aspects of the lessons where the emphasis was on active and interactive learning. Participation was least high in the teacher-led activities’ (Stead et al. 2007, p.119)  ‘In the qualitative interviews, teachers working with mixed-ability classes described the challenge of coping with an often very diverse range of pupils. Pupils with low literacy levels or short attention spans tended to struggle with some of the learning activities, while the more able in the class finished earlier and became bored...Some teachers described having to repeatedly *“stop and simplify”* tasks and to clarify the meaning of written instructions’ (Stead et al. 2007, p.114)  ‘Some teachers were concerned that some of the Blueprint content was inappropriate for the age and experiences of their pupils. Some teachers felt that the more naïve pupils were bewildered by some of the content, particularly on illegal drugs, and could not see the relevance to their own lives. As one of these teachers put it:’  *“Call me naïve, and I probably am, but I don’t think too many of our 11- and 12-year-olds have come in contact with crack*  *cocaine….Yes they do need to know about it but when I was delivering it, there was a bemused sort of silence….We had all*  *the details and things like poppers being used as a sex drug in the research, and I felt uncomfortable with that I must say.”* (Teacher) (Stead et al. 2007, p.115)  ‘Generally, pupils were perceived by teachers to engage better with content which resonated  with their own experiences, such as alcohol and tobacco.’  *“I personally would have thought that maybe, for Year 7, it might have been more appropriate to just focus on smoking and perhaps cannabis as well and maybe alcohol.”* (Blueprint Co-ordinator) (Stead et al. 2007, p.115)  ‘In the impact surveys, recall of Blueprint resources was high. Most young people expressed positive opinions about them and thought that they contained a lot of useful information. They were particularly positive about the pupil booklets and the board game  88% of the pilot school pupils recalled having played this game and 76% said that they had enjoyed it. The qualitative interviews were also very positive about the Blueprint materials. As with some of the classroom activities, some enjoyed doing the crosswords and other *Your Street Your Story* workbook activities in Year 7, while others found them difficult.’ (p.78) (Stead et al. 2007)  ‘This antipathy was also reported in the qualitative research with young people. Where they had experience of working in groups with other pupils whom they did not know well, young people found it harder to talk and to express their opinions. They were more concerned about how others would react to what they said and tended not to contribute as much as when they worked with their friends. Some felt particularly shy in this position and felt alienated during group work or class discussions, leaving them bored and disengaged. They also reported that they felt more confident about discussing sensitive topics, particularly about the use of cigarettes, alcohol and illicit drugs, in friendship groups where they could predict how their friends would react and did not worry  that they would be laughed at or that they would embarrass them by telling other pupils or get them into trouble with their teacher:’  *“If you know a person, you know what they’re thinking and everything. You’re more relaxed… if you’re with people you don’t like get on with, then you wouldn’t talk to anyone and don’t do as much in the lesson”*. (pupil, age NR) (Stead et al. 2007, p.82)  Characteristics of ‘good Blueprint teaching’ identified by pupils:  - being able to translate topics into real life situations  - being able to control the class, especially ‘where lessons were loud and there was a lot of interaction between pupils’  - being able to trust the teacher to maintain confidentiality  - the teacher being approachable  - teachers who ‘recognised young people’s right to choose whether or not to use drugs’ (Stead et al. 2007, p.95) |
| 2e | Providing support materials that are appealing and appropriate to pupils’ age, interests and culture  ‘Two of the 15 Blueprint lessons focused mainly on the normative education approach. Its impact and effectiveness depends on young people being convinced of the validity and reliability of any drug prevalence data that are used. In some pilot schools the majority of pupils clearly did not believe the survey evidence that was produced, particularly in relation to smoking, alcohol consumption and the use of volatile substances, even when the evidence was derived from questionnaires which they themselves had completed. This approach, if it is to be effective, probably needs to be integrated more into PSHE as a whole and pupils need a better understanding of how surveys are conducted and the steps which researchers take to ensure validity and reliability’ (Stead et al. 2007, p.118)  ‘The pupils often challenged whether they would react to real-life situations in the reflective way proposed in their Blueprint lessons’ (Stead et al. 2007, p.119)  ‘The learning activities introduced in Lesson 8, especially the activity called *Fags in the Loo*, were criticised by many of the pupils in observed lessons and by some of the teachers because they did not perceive them to be realistic. *Fags in the Loo* was seen as a bullying scenario and in their experience the offer to experiment with a particular substance was usually made by someone they knew and involved persuasion rather than bullying. It was also widely felt amongst the pupils that the suggested strategy, *The Five Refusal Skills*, would not work very effectively in ‘the real world’’ (Stead et al. 2007, p.119)  ‘Pupils also appreciated the quality of the support materials, particularly the board games, pupil booklets, drug information resources and card sort games. They felt that the topics and themes were very relevant to their lives and everyday experiences’ (Stead et al. 2007, p.119/120)  Pupils’ perceptions of drug information sources:  Pupils were given a list of 11 information sources on drugs, and asked whether they had gained a lot, a little, or no information at all from each source. 81% of pupils reported that they gained ‘a lot’ of information from lessons at secondary school, in both Y7 and Y8. Approximately a third reported that parents (33% in Y7, 32% in Y8) and leaflets/booklets (29% in Y7, 34% in Y8) were the next most common source, and a fifth (18% in Y7, 20% in Y8) cited friends. (Blueprint Evaluation Team 2007, p.18) |
| **3 – Embedding a programme into routine practice** | **The introduction and routine delivery (‘embedding’) of a programme takes time and motivation. It is likely to involve changes in the school environment and the development of new relationships between stakeholders that require pro-active management so that:** |
| 3a | - different stakeholders’ goals are reconciled - organisational decisions in other areas of school life are made taking into account how they impact on programme delivery - school staffs’ existing relationships with children are built upon - stakeholders’ enthusiasm, knowledge and experience are harnessed - knowledge of ‘core’ and ‘peripheral’ elements and minimum resources, skills and informational content is retained - responsibility for programme delivery becomes rooted in the school   NR |
| 3b | The longer-term sustainability of programmes and the extent to which health promoting messages and activities permeate other aspects of school life, is dependent on continuing feedback, encouragement, and expectations about implementation. Over time, this may originate less from outside the school and more from inside.  NR |
| **4 - Programme adaptation** | **The preparation for, introduction, initial delivery, and ongoing sustainability of a health promotion programme in a school is more likely to be successful when there is:** |
| 4a | Specificity about ‘core’ (essential) and ‘peripheral’ (optional/adaptable) programme elements, including the minimum levels of resources and/or skills necessary to support these elements (to inform decision-making about how to deliver a programme in the context of a particular school)  ‘the observations indicated that use of some resources, such as the pupil booklets, was variable. In some cases this was because the materials had not been distributed in time for specific lessons, although sometimes it was because teachers regarded use of any materials not explicitly specified in the lesson plan as optional’ (Stead D&AR 2007, p.660)  ‘Most teachers experienced difficulties in completing all of the content and tasks in the specified time, and adherence to the timings specified for each individual activity was low (only 36% in year 7 and 39% in year 8)’ (Stead D&AR 2007, p.660)  ‘For the most part, teachers tended to over-run significantly on core drug education activities and under-run on generic activities such as lesson introductions and reviews of what had been learned, even though the importance of reviewing what had been learned was emphasised in the Blueprint guidance. This was due partly to poor preparation by the teachers but mainly to unrealistic timings, particularly for activities that either involved pupils moving between groups or required considerable ‘settling down’ time afterwards. In the interviews, some teachers felt that the depth and quality of the learning experience for pupils were undermined by the pressure of the clock, and several experienced a tension between the need to adhere to content and methods and the need to adhere to specified timings. Having to curtail animated discussions or question and answer sessions in which pupils seemed genuinely interested also conflicted with notions of good teaching practice’ (Stead D&AR 2007, p.661)  The requirements of the programme in terms of fidelity were perceived by the teachers as very different to their normal practice, posing problems. 87% (yr7) and 79% (yr8) agreed it was ‘crucial that Blueprint was delivered exactly as instructed’, despite this 42% (yr7) felt that ‘teachers should be allowed to adapt to suit the need of their pupils’ this increased in yr8 to 59%. The prescriptive nature of the programme added pressure to delivery, feeling frequently frustrated that they could not alter or abort content they disliked or could not expand activities they felt were working well. ‘Other teachers welcomed the fidelity as it would force others to improve their practice or opt out of activities they may usually avoid, similarly it provided a ‘comfort blanket’ particularly for those new to PSHE, unfamiliar with topics or the methods’ (p.58).  *“I didn’t feel I had ownership of the lesson and I think that mattered. It was like somebody else had created this and I*  *wasn’t altogether at ease or as comfortable as I would normally be.”* (Blueprint Co-ordinator) (Stead et al. 2007, p.108)  Some teachers (Year 7) acknowledged they modified some lesson plans and omitted some activities due to unrealistic demands in the Blueprint manual, particularly in relation to the timings of some activities or what they perceived to be a lack of differentiation  for pupils with learning and behavioural difficulties. Some teachers reduced the scope for interactive learning because they were concerned about the loss of classroom control’ (p.70) (Stead et al. 2007)  ‘Several teachers reported ignoring the manual’s recommended way of allocating groups for specific activities, preferring instead to let the pupils stay in friendship groups. In their view, this saved time, ensured that they concentrated on the task in hand and reduced the scope for disruptive behaviour.’ (p.71) (Stead et al. 2007)  ‘Some teachers also reported curtailing particular activities that the pupils found dull or overelaborate… Year 8 lessons teachers were introducing modifications and cutting aspects of the lesson plans because they were feeling more relaxed in their approach to lesson preparation. They felt that they were still being faithful to the Blueprint approach but that they now had more flexibility in delivering it.’ (p.71) (Stead et al. 2007)  Based on adjusted scores – ‘the fact that a significant proportion of teachers failed to follow faithfully the requirements to explain the objectives of some of the lessons at the beginning and find five minutes for review and reflection at the end significantly deflated their overall fidelity scores. The main reason for this was that most teachers tended to over-run on the main learning activities and made up time by cutting these other generic elements.’ (p.73) (Stead et al. 2007)  [See Table 4.1] ‘Mean fidelity scores for each mode of delivery (pupil-pupil; teacher-class; teacher-led) were relatively high. The proportion of observed teachers consistently meeting or exceeding 75% was highest for those learning activities that involved teacher–pupil interactions in whole class settings and lowest for those activities which predominantly involved pupil–pupil  interaction. The relatively low mean fidelity score for teacher-led inputs may reflect the fact that teachers felt most in control of these activities and sometimes cut or modified them to save time for other learning activities.’ (p.74) (Stead et al. 2007)    ‘Some other teachers reported reducing the interactivity in certain activities because they felt that their pupils would not be able to cope.’ (p.75)  *“The role play – these children didn’t have the ability to do that……I still did the task as a group but what I did was I had*  *another teacher come into the class and I did the role play in front of everyone and then we took the children with us so we*  *did the role play together.”* (Blueprint Co-ordinator) (Stead et al. 2007, p.75)  ‘A small number of teachers reported that they made some of the learning activities more interactive than specified in the teacher manual, either because they involved too much input from the teacher or because they feared that the pupils would find them too boring.’ (p.75)  *“Rather than me reading all the scenario out [in Lesson 14] I actually got different people to read the different characters*  *out to make it a little bit more interesting, rather than hearing my voice all the way through”.* (Teacher) (Stead et al. 2007, p.75)  The group agreement (how Blueprint lessons would proceed) – this was on visibly displayed in 50% of observed Year 7 lessons and 65% of observed Year 8 lessons. More likely to be consistently displayed if all of the lessons were held in a designated PSHE room or in the teacher’s own form room. It was least likely to be consistently displayed if the teacher had to carry the group agreement poster around from lesson to lesson. Although the group agreement was more visible in Year 8 it was only referred to infrequently – references were observed in just 27% of lessons, compared with references to it in 51% of the observed Year 7 lessons.’ (p.81) (Stead et al. 2007)  Allocating pupils to groups – ‘Whilst the majority of teachers attempted to use the procedures proposed for allocating pupils  to groups, particularly in the earlier lessons, adherence declined for the later lessons and for Year 8. In the interviews a number of teachers commented that, during the training, they had found these allocation methods creative and enjoyable but felt that this was because professionals and adults were happy to work in whatever groups emerged from the process. They were emphatic that this was not usually the case with their pupils. Such was the difficulty of persuading pupils to work in any group other than friendship groups that some teachers reported that they abandoned the recommended allocation methods.’ (Stead et al. 2007)  Active and interactive learning – ‘Where teachers found themselves running out of time and found it necessary to cut  elements of the game or quiz, the pupils became frustrated and the learning value of the activity was diminished’ (Stead et al. 2007, p.97)  Factors influencing delivery (programme factors) - ‘the importance of fidelity, particularly in a research trial – stressed in the  teacher manuals and in the teacher training for Blueprint – also presented some teachers with particular challenges. Despite recognising and accepting the reasons for the emphasis on adherence to the lesson plans, teachers noted that it made delivery more stressful, particularly when observed by evaluators. Even when not observed, they still felt uncomfortable about keeping to the script when they disagreed with some aspects of the content or methods.’  *“I mean, I enjoyed doing Blueprint as well but I didn’t like the way you had to stick to [it]… there were certain tasks that the*  *kids really enjoyed and I would have liked to elaborate a bit more on them and other tasks which were a bit boring for them, so I would have liked to skip them but, as I said, I went to the programme.”* (Teacher) (Stead et al. 2007, p.107) |
| 4b | Scope for ‘mutual adaptation’ between the programme and the people delivering it, including the evolution and updating of programme content and mode of delivery over time  ‘content fidelity scores are contained within a band between 65% and 82%. The scores are generally lower for Year 8 (Lessons 11–15). The overall mean content fidelity score for all of the lessons (combining both years) is 72%, while the mean score for Year 7 was 74% and the mean score for Year 8 was 68%.’ (p.70) (Stead et al. 2007)  Differentiation – ‘In response to feedback, the teachers had asked for more flexibility in the structure of the learning activities, more opportunities for the more able students to pursue a topic in greater depth and also some alternative ways of approaching a task or activity that would ensure that the less able or pupils with specific learning difficulties would also be able to fully engage in the learning process.’ (p.82) (Stead et al. 2007)  ‘13 optional activities were included in the five Year 8 lessons. Five of these, one for each lesson, were described as ‘alternatives’. Four of the five options proposed alternative ways of delivering a specific learning activity for those pupils who were struggling with their reading, writing or number work or were generally less able. The fifth option was for use with the more able pupils and enabled them to explore the topic in more depth if they had finished the main activity ahead of the rest of the class.’ (p.82) (Stead et al. 2007)  ‘there was a tendency (in both years) for teachers to over-run on the main learning activities, particularly the longest ones, and this was then countered by cuts in the time available for review and reflection at the end of the lesson. Only 47% of the observed review sessions in Year 7 were close to their specified time and 41% were significantly under the specified time. In Year 8 less than one-third of observed reviews were delivered in or close to the specified time (usually five minutes) while just under a half of the reviews were delivered in less than the specified time.’ (p.86)  *“They [Blueprint] were wanting that level of sensitive response but I just feel that the ‘pace’… .and rigour of the lessons, two*  *horrible words in education, I don’t think they naturally lent themselves to creating that atmosphere for that to happen. I*  *think if you’re being reflective, if you’re being sensitive in discussion, you can’t be looking at your watch whilst you do*  *that.”* (Teacher) (Stead et al. 2007, p.86)  ‘Based on feedback from year 7 that teachers felt they did not have sufficient time to cover everything adequately, programme developers reduced the average number of activities per lesson for year 8, but lessons continued to be content-rich, and time constraints continued to be a problem for some teachers’ (Stead D&AR 2007, p.659)  Factors influencing delivery (responsiveness to feedback from teachers) – ‘There was a widespread feeling amongst the teachers that the development team had listened to them and introduced changes in the Year 8 programme that reflected their concerns. The timings for activities were perceived to be more realistic. They liked the more flexible design of the lesson plans and the introduction of voluntary extension activities.’  *“I think with the Year 8 there was the feeling that the quality was really there rather than being spread over 10 and getting*  *some dodgy ones like the media lesson”.* (Blueprint Co-ordinator, Area C, School 11, Year 8) (Stead et al. 2007, p.108)  ‘The Blueprint curriculum component appeared to be quite robust and flexible. Although some teachers felt that it was too prescriptive, most of them changed their minds as they began to implement it. The majority of participating teachers welcomed the detailed lesson specifications for delivering an area of the curriculum with which they were not familiar or experienced. The component itself proved flexible enough to be delivered through different timetabling arrangements (i.e. in blocks of lessons or on a weekly basis) and mainly worked with pupils across a wide ability range’ (Stead et al. 2007, p.117)  ‘Some teachers reported that they felt that the fidelity requirement and the content-rich lessons made it difficult for them to employ their usual strategies for maintaining control in the classroom when pupils were being disruptive or noisy’ (Stead et al. 2007, p.117) |

| **HP Programme** |  |
| --- | --- |
| Name of programme | Citizenship Safety Project |
| Area of HP | Injury prevention |
| Authors (year/ study type) | Frederick & Barlow (2006/ CBA pilot study) |
| Quality appraisal | Limited information on school context. Opportunistic sample, therefore limited representativeness of population. No triangulation or validity checks reported for qualitative data analysis. |
| Programme description | ‘In response to concerns to widen the age range covered by injury prevention education in schools, the Injury Minimization Programme for Schools (IMPS) team developed the Citizenship Safety Project (CSP). This innovative project supports citizenship education and can be integrated into the National Curriculum in PSHE lessons, meeting requirements of Key Stage 4 attainment targets in citizenship. The CSP is a cross-age tutoring intervention in which Year 10 (14–15 years) students teach Year 2 pupils (6–7 years) aspects of accident prevention and risk awareness. (p.89) |
| Year(s) delivered | NR |
| Description of UK location(s) where delivered | UK location - NR  Demographic composition of schools:   \|  \| Secondary school \| Primary school \| \| --- \| --- \| --- \| \| School size \| 880 \| 401 \| \| Ethnic minorities (%) \| 1.25 (EFL) \| 1.7 \| \| Special educational needs (%) \| 12.4 \| 16.5 \| \| Free school meals (%) \| 6.6 \| 8.5 \| \| Statemented (%) \| 2 \| 0 \| \| Attainment on entry \| Above average \| Above average \| |
| **Research methods** |  |
| Theoretical approach | NR |
| Sample | Opportunistic |
| Participants | 22 (14-15 yrs), 55 (6-7 yrs), 1 teacher (yr 10), 1 teacher (yr 2), 4 male tutors |
| Data collection | Interviews (n=2, teachers) - to explore teacher’s perceptions of project, perceived advantages / disadvantages, barrier or facilitators  Diaries - (n=22 (only 13 completed)) – user perceptions concerning acceptability of project  Draw and write technique; “smiley faces” scale (n=NR) – knowledge about safety and risk awareness  Questionnaires (n=NR) – Self Esteem Scale (Rosenberg, 1965), Locus of Control Scale for Children (Nowicki & Strickland, 1973) |
| Analysis | Teacher interviews - reflections, categories, codes, themes (analysed by hand i.e. ‘cut up’ & ‘clumped together’)  Year 10 diaries - thematic analysis (analysed with NUD*IST)  Draw & Write – assessed through Southampton University then quantified and categorised using descriptive statistics and proportions  Questionnaires – Wilcoxon signed-rank test |
| Time of follow-up | Immediately post intervention |
| **Evidence about programme theory#** |  |
| **1 - Preparing for implementation** | **Preparation for the introduction of a health promotion programme to a school is more likely to be successful when systematically planned -** this involves**:** |
| 1a | Consultation with stakeholders, which involves:   - providing information on the programme - eliciting views on ‘readiness for change’ as a starting point for engagement - encouraging the sharing of previous experiences of delivering health promotion programmes - facilitating discussion about the concordance (‘fit’) of the programme with:   - current practice  - students’ interests  - current school policies, resources and organisation  NR |
| 1b | Consideration of the concordance of the programme with current practice and interests  ‘Structure, organisation and the CSP’s ability to reduce teacher input and increase student input were key elements discussed by both teachers:’ (p.92)  “The way the project is structured…teachers do not have to put much in as it’s student led…you don’t need loads of resources…” [Year 10 teacher] (p.92) |
| 1c | Identifying clear aims and priorities  NR |
| 1d | Taking into account the current situation and competencies in a school  NR |
| **2 - Establishing a programme** | **The introduction of a health promotion programme to a school is more likely to be successful when it is incorporated into school activities through:** |
| 2a | Being integrated into school policy  NR |
| 2b | School staff using their leadership skills to co-ordinate activities or resources for programme delivery  NR |
| 2c | Providing adequate opportunity and/or training for the personal and professional development of those who will deliver the programme  NR |
| 2d | Providing support materials that are appealing and appropriate to students’ age, interests and culture  NR |
| **3 – Embedding a programme into routine practice** | **The introduction and routine delivery (‘embedding’) of a programme takes time. It is likely to involve changes in the school environment and the development of new relationships between stakeholders that require pro-active management so that:** |
| 3a | - different stakeholders’ goals are reconciled - organisational decisions are co-ordinated - stakeholders’ existing relationships with children are built upon - stakeholders’ enthusiasm, knowledge and experience are harnessed   NR |
| 3b | The longer-term sustainability of programmes and the extent to which health promoting messages and activities permeate other aspects of school life, is dependent on continuing feedback, encouragement, and expectations about implementation.  ‘Areas thought to require modification included: evaluation, reflection and “closing the loop” or revisiting what they had learned’ (p.92)  ‘Disruption was not seen as a potential problem if the structure and planning of the project were maintained’ (p.92) |
| **4 - Programme adaptation** | **The introduction, initial delivery, and ongoing sustainability of a health promotion programme in a school is more likely to be successful when there is:** |
| 4a | Specificity about ‘core’ (essential) and ‘peripheral’ (optional/adaptable) programme elements (to inform decision-making about how to deliver a programme in the context of a particular school)  NR |
| 4b | Scope for ‘mutual adaptation’ between the programme and the people delivering it  ‘Resources were a key issue if teachers were to be trained’ (p.92)  ‘One of the main advantages that the teachers perceived was the transferability of the project to other year groups’ (p.91) |

| **HP Programme** |  |
| --- | --- |
| Name of programme | Eat Smart Play Smart |
| Area of HP | Obesity |
| Authors (year/ study type) | Warren et al. (2003/ RCT) |
| Quality appraisal | No detail regarding process and impact evaluation findings i.e. context, primary evidence (authors comments only), data collection (measures only), data analysis, reflexivity or generalisability. |
| Programme description | ‘The Be Smart programme lasted for four school terms (~14 months) and was held over 8 weeks per term, weekly in term 1 and fortnightly in terms 2-4, giving an intervention of 20 weeks in total. Intervention groups (nutrition and/or physical activity curriculum) lasted 25 minutes, were delivered at lunchtime clubs, had an interactive approach and were behaviourally focussed (based on Social Learning Theory - Bandura, 1986) and incorporated:  - raising the value of the desired behaviour, including the short-term benefits, which are most likely to appeal to children  - providing the opportunity to taste healthy foods and undertake non-competitive physical activity  - providing incentives to reinforce messages, for example verbal praise and small prizes  - developing practical skills and thus self-confidence in the desired behaviour  - working with parents (as far as possible) to overcome barriers to the desired health behaviour.  All materials developed were reviewed by experienced primary school teachers and amended on the basis of their recommendations.’ (p.289) |
| Year(s) delivered | 2000 |
| Description of UK location(s) where delivered | Three primary school in Oxford, selected on the basis of previous links to the Nutrition and Food Science Department at Oxford Brookes University and their close proximity to the university |
| **Research methods** |  |
| Theoretical approach | NA |
| Sample | ‘All children in years 1 and 2 (aged 5-7 years) from three primary schools in Oxford were targeted in January 2000. The primary schools were selected on the basis of previous links to the Nutrition and food Science Department at Oxford Brookes University and their close proximity to the University’ (p.288) |
| Participants | Children aged 5-7yrs |
| Data collection | Log of lesson evaluations, parental phone calls and letters  ‘Satisfaction survey’ regarding the intervention (parents and teachers) |
| Analysis | NR |
| Time of follow-up | 1 month after end of intervention |
| **Evidence about programme theory#** |  |
| **1 - Preparing for implementation** | **Preparation for the introduction of a health promotion programme to a school is more likely to be successful when systematically planned -** this involves**:** |
| 1a | Consultation with stakeholders, which involves:   - providing information on the programme - eliciting views on ‘readiness for change’ as a starting point for engagement - encouraging the sharing of previous experiences of delivering health promotion programmes - facilitating discussion about the concordance (‘fit’) of the programme with:   - current practice  - students’ interests  - current school policies, resources and organisation  NR |
| 1b | Consideration of the concordance of the programme with current practice and interests  NR |
| 1c | Identifying clear aims and priorities  NR |
| 1d | Taking into account the current situation and competencies in a school  NR |
| **2 - Establishing a programme** | **The introduction of a health promotion programme to a school is more likely to be successful when it is incorporated into school activities through:** |
| 2a | Being integrated into school policy  NR |
| 2b | School staff using their leadership skills to co-ordinate activities or resources for programme delivery  NR |
| 2c | Providing adequate opportunity and/or training for the personal and professional development of those who will deliver the programme  NR |
| 2d | Providing support materials that are appealing and appropriate to students’ age, interests and culture  ‘From the lesson evaluations in term 1 it was clear that children enjoyed practical tasks, quizzes and tasting sessions, and that these were incorporated as far as possible in future lessons.’ (p. 293)  Parental survey:  93% of parents rated programme content as average or above  88% rated their child’s enjoyment of the programme as above average |
| **3 - Programme adaptation** | **The introduction, initial delivery, and ongoing sustainability of a health promotion programme in a school is more likely to be successful when there is:** |
| 3a | Specificity about ‘core’ (essential) and ‘peripheral’ (optional/adaptable) programme elements (to inform decision-making about how to deliver a programme in the context of a particular school)  NR |
| 3b | Scope for ‘mutual adaptation’ between the programme and the people delivering it  ‘All the teachers felt it would be useful to incorporate some of the materials developed in the Be Smart programme into the curriculum, e.g. in Personal Social and Health Education (PSHE).’ (survey results) (p. 294) |
| **4 – Embedding a programme into routine practice** | **The introduction and routine delivery (‘embedding’) of a programme takes time. It is likely to involve changes in the school environment and the development of new relationships between stakeholders that require pro-active management so that:** |
| 4a | - different stakeholders’ goals are reconciled - organisational decisions are co-ordinated - stakeholders’ existing relationships with children are built upon - stakeholders’ enthusiasm, knowledge and experience are harnessed   NR |
| 4b | The longer-term sustainability of programmes and the extent to which health promoting messages and activities permeate other aspects of school life, is dependent on continuing feedback, encouragement, and expectations about implementation.  NR |

| **HP Programme** |  |
| --- | --- |
| Name of programme | Eat Smart Play Smart |
| Area of HP | Obesity |
| Authors (year/ study type) | Warren et al. (2003/ RCT) |
| Quality appraisal | No detail regarding process and impact evaluation findings i.e. context, primary evidence (authors comments only), data collection (measures only), data analysis, reflexivity or generalisability. |
| Programme description | ‘The Be Smart programme lasted for four school terms (~14 months) and was held over 8 weeks per term, weekly in term 1 and fortnightly in terms 2-4, giving an intervention of 20 weeks in total. Intervention groups (nutrition and/or physical activity curriculum) lasted 25 minutes, were delivered at lunchtime clubs, had an interactive approach and were behaviourally focussed (based on Social Learning Theory - Bandura, 1986) and incorporated:  - raising the value of the desired behaviour, including the short-term benefits, which are most likely to appeal to children  - providing the opportunity to taste healthy foods and undertake non-competitive physical activity  - providing incentives to reinforce messages, for example verbal praise and small prizes  - developing practical skills and thus self-confidence in the desired behaviour  - working with parents (as far as possible) to overcome barriers to the desired health behaviour.  All materials developed were reviewed by experienced primary school teachers and amended on the basis of their recommendations.’ (p.289) |
| Year(s) delivered | 2000 |
| Description of UK location(s) where delivered | Three primary school in Oxford, selected on the basis of previous links to the Nutrition and Food Science Department at Oxford Brookes University and their close proximity to the university |
| **Research methods** |  |
| Theoretical approach | NA |
| Sample | ‘All children in years 1 and 2 (aged 5-7 years) from three primary schools in Oxford were targeted in January 2000. The primary schools were selected on the basis of previous links to the Nutrition and food Science Department at Oxford Brookes University and their close proximity to the University’ (p.288) |
| Participants | Children aged 5-7yrs |
| Data collection | Log of lesson evaluations, parental phone calls and letters  ‘Satisfaction survey’ regarding the intervention (parents and teachers) |
| Analysis | NR |
| Time of follow-up | 1 month after end of intervention |
| **Evidence about programme theory#** |  |
| **1 - Preparing for implementation** | **Preparation for the introduction of a health promotion programme to a school is more likely to be successful when systematically planned -** this involves**:** |
| 1a | Consultation with stakeholders, which involves:   - providing information on the programme - eliciting views on ‘readiness for change’ as a starting point for engagement - encouraging the sharing of previous experiences of delivering health promotion programmes - facilitating discussion about the concordance (‘fit’) of the programme with:   - current practice  - students’ interests  - current school policies, resources and organisation  NR |
| 1b | Consideration of the concordance of the programme with current practice and interests  NR |
| 1c | Identifying clear aims and priorities  NR |
| 1d | Taking into account the current situation and competencies in a school  NR |
| **2 - Establishing a programme** | **The introduction of a health promotion programme to a school is more likely to be successful when it is incorporated into school activities through:** |
| 2a | Being integrated into school policy  NR |
| 2b | School staff using their leadership skills to co-ordinate activities or resources for programme delivery  NR |
| 2c | Providing adequate opportunity and/or training for the personal and professional development of those who will deliver the programme  NR |
| 2d | Providing support materials that are appealing and appropriate to students’ age, interests and culture  ‘From the lesson evaluations in term 1 it was clear that children enjoyed practical tasks, quizzes and tasting sessions, and that these were incorporated as far as possible in future lessons.’ (p. 293)  Parental survey:  93% of parents rated programme content as average or above  88% rated their child’s enjoyment of the programme as above average |
| **3 - Programme adaptation** | **The introduction, initial delivery, and ongoing sustainability of a health promotion programme in a school is more likely to be successful when there is:** |
| 3a | Specificity about ‘core’ (essential) and ‘peripheral’ (optional/adaptable) programme elements (to inform decision-making about how to deliver a programme in the context of a particular school)  NR |
| 3b | Scope for ‘mutual adaptation’ between the programme and the people delivering it  ‘All the teachers felt it would be useful to incorporate some of the materials developed in the Be Smart programme into the curriculum, e.g. in Personal Social and Health Education (PSHE).’ (survey results) (p. 294) |
| **4 – Embedding a programme into routine practice** | **The introduction and routine delivery (‘embedding’) of a programme takes time. It is likely to involve changes in the school environment and the development of new relationships between stakeholders that require pro-active management so that:** |
| 4a | - different stakeholders’ goals are reconciled - organisational decisions are co-ordinated - stakeholders’ existing relationships with children are built upon - stakeholders’ enthusiasm, knowledge and experience are harnessed   NR |
| 4b | The longer-term sustainability of programmes and the extent to which health promoting messages and activities permeate other aspects of school life, is dependent on continuing feedback, encouragement, and expectations about implementation.  NR |

| **HP Programme** |  |
| --- | --- |
| Name of programme | GGHB Sexual health programme |
| Area of HP | Sex and relationship education (SRE) |
| Authors (year/ study type) | Lowden & Powney (1996/ process evaluation) |
| Quality appraisal | Even though the study design (single school) was intended to inform local decision-making by feeding into the development of the programme, there are still considerable limitations – in particular, the sampling and data analysis strategies are not described and the potentially significant impact (social desirability bias) of only conducting (separate) *group* interviews with pupils and teachers is not considered. However, the authors are careful to situate the analysis within key contextual information, e.g. differences between boys’ and girls’ experiences, the local social networks of the pupils. |
| Programme description | The sexual health education model developed by Greater Glasgow Health Board (GGHB) was part of an on-going effort by the health board to design a school-based approach for use in areas of multiple deprivation. The main aim of the programme was to ‘raise awareness of the meaning of full-sexual well-being, encourage informed choice, and discourage unplanned pregnancy’ (p.52). The programme was developed following two years of pilot work, emphasised a non-judgmental ethos and was delivered by teachers (over 5 weeks, 1 lesson per week) to mainly single sex groups as part of the PSE curriculum. Teachers had access to GGHB programme staff for advice. Programme involved whole class, small group and individual work, with supporting materials such as video, reading material and quizzes. Topics included biology and physiology, relationships, safer sex, pregnancy, and communication and power in relationships. Project work aimed to ‘enable students to examine in detail sexual health issues that affect them’ (p.53).  Programme approach summarised as: ‘grounded in a belief that pupils should explore their own lives in regard to sexuality and sexual health. This experiential approach emphasises pupils’ ownership of the issues and challenges misconceptions and myths with factual information. Pupils are encouraged to process their own decisions concerning sexual activity. Programme aims to encourage peer-support within classes to counter sexual pressure and embarrassment over body changes.’ (p.52) |
| Year(s) delivered | 1995-1996 |
| Description of UK location(s) where delivered | One school (660 pupils; 60 staff) situated in a large urban housing estate on the outskirts of Glasgow where the number of women aged <22y having children is far higher than the average for the Greater Glasgow area. The area was ‘recognised as an area of multiple disadvantage and designated by the regional authority as an Area for Priority Treatment’. (p.4) |
| **Research methods** |  |
| Theoretical approach | NR |
| Sample | NR |
| Participants | Pupils 11-16y/ Teachers |
| Data collection | Questionnaire (pupils; mostly open-ended questions) (before (n=133) and after (n=99) programme delivery)  ‘Whole class’ interviews (approximately 20 pupils per class) – Before and after programme delivery - two classes aged 11-12y and two aged 15-16y  ‘Group’ interviews with teachers (before (n=10) and after (n=NR; ‘all teachers who worked on the programme’ p.18) programme delivery) |
| Analysis | NR |
| Time of follow-up | Immediately before and after programme delivery |
| **Evidence about programme theory#** |  |
| **1 - Preparing for implementation** | **Preparation for the introduction of a health promotion programme to a school is more likely to be successful when systematically planned in conjunction with other school responsibilities.** This involves: |
| 1a | Consultation with stakeholders, which involves:   - providing information on the programme (e.g. who and what is involved, evidence of effectiveness)   ‘Before the programme started, the teachers and health worker had four, one hour sessions where the materials were  scrutinised and discussed. This process also acted as a mechanism for fine-tuning the content to match the comments of the teachers’ (p.28)   - eliciting views on ‘readiness for change’ as a starting point for engagement - encouraging the sharing of previous experiences and knowledge of delivering health promotion programmes   ‘Being able to work in close collaboration with the Health Board in designing the programme seemed to be reassuring and promote teachers’ confidence in the programme.’ (p.29) (no data reported)  ‘The teachers said that familiarity with the material, experience of teaching on the school’s previous sexual health programme and their knowledge of the pupils had allowed them to deliver the programme effectively’ (p.46)   - facilitating discussion about the concordance (‘fit’) of the programme with:   - current practice  - pupils’ interests  - current school policies, resources and organisation  ‘The Health Board emphasised that the school should have ownership of the programme and its negotiations and work with the school have been guided by this principle’ (p.4)  Pupil survey during programme development identified the different interests of pupils, e.g. 11-13y – physical development, puberty, generic sexual health advice; 14-16y – HIV and other STDs; ‘information [which pupils saw] as relevant to their current lifestyle’ (p.21)  Discussion in group interviews identified differences in perceptions of boys and girls about the suitability of working in mixed-sex group. Boys preferred mixed-sex groups as ‘they could hear what the girls had to say on the topics and the course included learning about the opposite sex and relationships’. Girls were ‘notably less enthusiastic… they generally saw the boys as immature and likely to break confidentiality rules concerning any topics that were raised’ (p.25). No data presented to support these claims.  Teachers welcomed the ‘teacher-led’ programme, which they viewed as likely to ‘minimise disruption, emphasise continuity, and improve discipline’, although they also acknowledged that health workers could offer subject expertise and provide a ‘fresh face’ that could appeal to pupils (p.29) (no data reported) |
| 1b | Identification of a potential health benefit for pupils at a local level  NR |
| 1c | Consultation with stakeholders (head-teacher, school staff, pupils, parents, governors)  ‘Being able to work in close collaboration with the Health Board in designing the programme seemed to be reassuring and promote teachers’ confidence in the programme’ (p.29)  ‘The teachers who were involved in the programme actively shaped the curriculum and the Health Board designed the programme to take on board the teachers’ comments and earlier evaluation findings.’ (p.27)  ‘The teachers stressed the extremely important input and effort of the Health Board staff during their work with the school and said that their work contributed to the incorporation of the programme into the school curriculum.’ (p.31) (no data reported) |
| 1d | Consideration of the concordance of the programme with current practice and interests  NR |
| 1e | Identifying clear aims and priorities, including intended outcomes  NR |
| 1f | Taking into account the current situation and competencies in a school and the implications of these for programme refinement  NR |
| **2 - Introducing a programme** | **The introduction of a health promotion programme to a school is more likely to be successful when it is incorporated into school activities through:** |
| 2a | Being integrated into school policy (e.g. a School Improvement Plan) and supported by governors and senior staff to whom monitoring and progress reports are made  NR |
| 2b | School staff using their leadership skills to co-ordinate activities or resources for programme delivery  NR |
| 2c | Providing adequate opportunity and/or training for the personal and professional development of those who will deliver the programme and engaging with their interests  ‘Meetings with the teachers every month provided continuing support and addressed any emerging needs or queries’ (p.28) |
| 2d | Modes of delivery that appeal to and engage pupils and provide cognitive and/or emotional rewards  Although programme generally well-received by pupils, 15-16y old girls identified a number of areas where they felt that programme did not address their needs (HIV, contraception, pregnancy and options during pregnancy) and that teachers were largely unwilling to discuss these issues further. Authors note that within the catchment area of the school teenage pregnancy was not unusual, and that sexual relationships and pregnancy were ‘normalised’ within the girls’ social networks.  ‘Pupils can be seen to become more reluctant to talk to older adults (teachers and parents) about sexual health [at age 13y upwards]… as parents and teachers are often seen by young people as having a disciplinary role as well as a pastoral role.’ (p.40) (not data reported) |
| 2e | Providing support materials that are appealing and appropriate to pupils’ age, interests and culture  ‘A book used to inform pupils about teenage sexual health issues was criticised by the S5 girls in their group interviews as being for a younger age group and the experiences and scenarios covered had long since been encountered and dealt with by these girls. The S5 girls commented on other texts which had jargon or were ‘full of complicated words’ and were difficult to read’ (p.34) |
| **3 – Embedding a programme into routine practice** | **The introduction and routine delivery (‘embedding’) of a programme takes time and motivation. It is likely to involve changes in the school environment and the development of new relationships between stakeholders that require pro-active management so that:** |
| 3a | - different stakeholders’ goals are reconciled - organisational decisions in other areas of school life are made taking into account how they impact on programme delivery - school staffs’ existing relationships with children are built upon - stakeholders’ enthusiasm, knowledge and experience are harnessed - knowledge of ‘core’ and ‘peripheral’ elements and minimum resources, skills and informational content is retained - responsibility for programme delivery becomes rooted in the school   NR |
| 3b | The longer-term sustainability of programmes and the extent to which health promoting messages and activities permeate other aspects of school life, is dependent on continuing feedback, encouragement, and expectations about implementation. Over time, this may originate less from outside the school and more from inside.  NR |
| **4 - Programme adaptation** | **The preparation for, introduction, initial delivery, and ongoing sustainability of a health promotion programme in a school is more likely to be successful when there is:** |
| 4a | Specificity about ‘core’ (essential) and ‘peripheral’ (optional/adaptable) programme elements, including the minimum levels of resources and/or skills necessary to support these elements (to inform decision-making about how to deliver a programme in the context of a particular school)  ‘The lesson plans were very clearly set out which helped teachers make the transition to teaching the programme without the health workers being present’ (p.46) |
| 4b | Scope for ‘mutual adaptation’ between the programme and the people delivering it, including the evolution and updating of programme content and mode of delivery over time  NR |

| **HP Programme** |  |
| --- | --- |
| Name of programme | Healthy Lifestyle Programme (HeLP) |
| Area of HP | Obesity |
| Authors (year/ study type) | Wyatt et al. (2011/ CRCT (pilot) process evaluation) |
| Quality appraisal | Substantial detail on how Intervention Mapping process was used. The iterative development of the intervention is closely described. However, limited information on how data analysis and interpretation was conducted and no primary supporting data on implementation process provided. |
| Programme description | ‘The Healthy Lifestyle Programme (HeLP) is a school-based intervention which seeks to deliver healthy lifestyle messages and provide simple individually tailored strategies to assist change relating to healthy activity and eating. HeLP takes a population approach seeking to change behaviour at a family as well as at an individual and institutional level. HeLP aims to deliver a general healthy lifestyle message relating to the energy balance and within this context, three evidence-based key messages are emphasised: a decrease in the consumption of sweetened fizzy drinks, an increase in the proportion of healthy snacks consumed and a reduction in TV viewing and other screen-based activities.’ (p.2) The following activities took place:  Term 1 – Assembly, competition, rugby and dance workshops, parents’ evening  Term 2 – PSHE lessons, drama activities (‘children choose which character they most resemble and then work with that character to help them change their behaviour’ (p.7), parent information sheets, 1:1 goal-setting, newsletters, parents’ evenings  Term 3 – Assembly, PSHE lesson, drama workshop, class assembly, 1:1 goal reassessment, newsletter  The 18-month programme aimed to use processes of behaviour change, namely:  - establishing motivation and creating a receptive environment  - developing children’s confidence and skills and helping them make decisions  - helping children create an action plan and implement goals  - keeping children motivated by helping them to monitor, assess and adapt goals |
| Year(s) delivered | Phase 1 (2005-2006) Phase 2 (2006-2008) Phase 3 (2008-2010) |
| Description of UK location(s) where delivered | Six schools in the South West of England  Phase 1 – NR  Phase 2 – an area of high deprivation  Phase 3 - NR |
| **Research methods** |  |
| Theoretical approach | NR |
| Sample | Phase 1 & 2 NR, Phase 3 purposive / randomised |
| Participants | Phase 1 – children aged 8-11yrs  Phase 2 – children aged 9-10yrs  Phase 3 – children aged 9-10yrs |
| Data collection | Phase 1   - Focus groups (n=6/ 33 children, 2 per year group) – views on activities, messages and concepts the children remembered - Semi-structured interviews (n=6 teachers) – content NR - Parents invited to complete a questionnaire about the study (n=119)   Phase 2   - Focus groups (n=3/ 18 children) – two groups who ‘engaged fully’ (n=14) and one ‘indifferent’ group (n=4) - Interviews (n=3, staff; n=1, headteacher; n=5 parents) - Parents also invited to complete a questionnaire (n=77)   Phase 3   - Focus groups (n=6/ 38 children) – content NR - Semi-structured interviews (n=9, staff; n=17, parents) – content NR   Questionnaires |
| Analysis | NR |
| Time of follow-up | Phase 1 (NR); Phase 2 (6 week post intervention); Phase 3 (18 & 24 months after baseline) |
| **Evidence about programme theory#** |  |
| **1 - Preparing for implementation** | **Preparation for the introduction of a health promotion programme to a school is more likely to be successful when systematically planned in conjunction with other school responsibilities.** This involves: |
| 1a | Consultation with stakeholders, which involves:   - providing information on the programme (e.g. who and what is involved, evidence of effectiveness) - eliciting views on ‘readiness for change’ as a starting point for engagement - encouraging the sharing of previous experiences and knowledge of delivering health promotion programmes - facilitating discussion about the concordance (‘fit’) of the programme with:   - current practice  ‘Teachers thought that the education lessons should be taught consecutively over 1 week to maintain momentum and that the drama and goal setting had the potential to work synergistically by engaging the children through the drama and following this up with encouraging the children, with their parents support, to make changes through setting simple goals.’ (p. 4) Phase 1  ‘Teachers agreed Year 5 was the right target group as children are gaining independence while still amenable to the messages.’ (p. 7) Phase 3  - pupils’ interests  - current school policies, resources and organisation |
| 1b | Identification of a potential health benefit for pupils at a local level  NR |
| 1c | Consultation with stakeholders (head-teacher, school staff, pupils, parents, governors)  NR |
| 1d | Consideration of the concordance of the programme with current practice and interests  ‘Staff were enthusiastic about the programme, in part because it met the National Curriculum guidelines for Personal Social Health Education and Citizenship, and importantly because they felt it promoted families’ engagement with the school.’ (p. 4) Phase 2 |
| 1e | Identifying clear aims and priorities, including intended outcomes  NR |
| 1f | Taking into account the current situation and competencies in a school and the implications of these for programme refinement  NR |
| **2 - Introducing a programme** | **The introduction of a health promotion programme to a school is more likely to be successful when it is incorporated into school activities through:** |
| 2a | Being integrated into school policy (e.g. a School Improvement Plan) and supported by governors and senior staff to whom monitoring and progress reports are made  NR |
| 2b | School staff using their leadership skills to co-ordinate activities or resources for programme delivery  NR |
| 2c | Providing adequate opportunity and/or training for the personal and professional development of those who will deliver the programme and engaging with their interests  NR |
| 2d | Modes of delivery that appeal to and engage pupils and provide cognitive and/or emotional rewards  ‘All children (n=80) in the intervention schools participated in the healthy lifestyle week and 90% set goals with their families around lifestyle change. 75% of parents participated in one or more programme activity.” (p.7) Over the three phases <1% of children dropped-out.  ‘The children enjoyed the drama activities and felt that they could relate to the characters within the drama framework who made them more motivated to set their own goals.’ (p. 5) Phase 2  ‘Children were unanimous in their enjoyment of the drama activities and equally unequivocal that these activities should be carried out by people external to the school.’ (p. 7) Phase 3  ‘Qualitative data revealed that the dynamic nature of the drama and the use of the young actors did indeed engage the children to such an extent that they talked exuberantly about the programme with their parents and siblings and encouraged them to attend events, particularly if they were going to be performing with the actors’ (p. 8)  ‘Teachers felt using young actors to deliver the messages was key to achieving engagement with this age group... Some commented that the intervention had boosted the children’s self-esteem, had a positive effect on the class socially and created additional opportunities to link with parents’ (p. 7) Phase 3 |
| 2e | Providing support materials that are appealing and appropriate to pupils’ age, interests and culture  NR |
| **3 – Embedding a programme into routine practice** | **The introduction and routine delivery (‘embedding’) of a programme takes time and motivation. It is likely to involve changes in the school environment and the development of new relationships between stakeholders that require pro-active management so that:** |
| 3a | - different stakeholders’ goals are reconciled - organisational decisions in other areas of school life are made taking into account how they impact on programme delivery - school staffs’ existing relationships with children are built upon   ‘Some teachers suggested further activities for the subsequent term to reinforce the messages and refocus the children and their parents on their goals.’ (p. 4) Phase 2   - stakeholders’ enthusiasm, knowledge and experience are harnessed - knowledge of ‘core’ and ‘peripheral’ elements and minimum resources, skills and informational content is retained - responsibility for programme delivery becomes rooted in the school |
| 3b | The longer-term sustainability of programmes and the extent to which health promoting messages and activities permeate other aspects of school life, is dependent on continuing feedback, encouragement, and expectations about implementation. Over time, this may originate less from outside the school and more from inside.  NR |
| **4 - Programme adaptation** | **The preparation for, introduction, initial delivery, and ongoing sustainability of a health promotion programme in a school is more likely to be successful when there is:** |
| 4a | Specificity about ‘core’ (essential) and ‘peripheral’ (optional/adaptable) programme elements, including the minimum levels of resources and/or skills necessary to support these elements (to inform decision-making about how to deliver a programme in the context of a particular school)  NR |
| 4b | Scope for ‘mutual adaptation’ between the programme and the people delivering it, including the evolution and updating of programme content and mode of delivery over time  NR |

| **HP Programme** |  |
| --- | --- |
| Name of programme | Kids Adult Together (KAT) Programme |
| Area of HP | Substance use (alcohol) |
| Authors (year/ study type) | Rothwell & Segrott (2011/ process evaluation of pilot) |
| Quality appraisal | Although a pilot study, convenience sampling strategy and low survey response rate are weaknesses). However, it is clear that the process evaluation endeavoured to include all stakeholders (children, parents, teachers) and to make use of a wide range of data collection methods (survey, focus groups (at a level appropriate for children, including ‘draw & write’), interviews and observation). |
| Programme description | Kids, Adults Together (KAT) comprises of a classroom component, engagement with parents through a fun evening for families with children aged 9-11 years, and a specially made DVD for children to take home, for families to watch together |
| Year(s) delivered | Piloted during 2008 |
| Description of UK location(s) where delivered | Two schools in South East Wales. The areas served by the schools had:  - Substantially more lone-parent households with dependent children than the national average.  - Percentages of children entitled to free school meals were well above the national average  - Attendance figures were below the national target.  - Marked differences between the schools’ ethos, teaching and communication cultures and the head teachers’ leadership styles.  - Socioeconomic and geographical characteristics of the areas served by the schools were also very different.  - School 1 was in the South Wales Valleys where mining had been the chief occupation and unemployment had been high since closure of the mine.  - School 2 was in a suburb of a market town in a rural area where farming and tourism were the most important occupations and there were good transport links. |
| **Research methods** |  |
| Theoretical approach | NR |
| Sample | NR |
| Participants | Children aged 9-11yrs/ parents |
| Data collection | Phase 1 - how KAT originated and developed, relationship to existing evidence, theory and its aims  Audit trail (32 documents)/ interviews (n=10)  Phase 2  Classroom and Fun Evening observation (total 20h) - adherence to KAT manual; use of interactive methods; level of participation; which concepts, types of knowledge and skills were addressed  7 focus groups (n=41)/ Interviews (n=18)/ Follow-up interviews (n=8)  Interviews with teachers (programme delivery)  Interviews with parents (perceptions of programme)  Questionnaire (n=38) - parents views on KAT DVD, fun evening, reasons for non-attendance where applicable |
| Analysis | Thematic content analysis approach (NVivo 8)  Descriptive statistics from questionnaire using (SPSS 16) |
| Time of follow-up | Two rounds of focus groups and interviews:  1) 6 – 23 days after KAT event  2) 3 months later |
| **Evidence about programme theory#** |  |
| **1 - Preparing for implementation** | **Preparation for the introduction of a health promotion programme to a school is more likely to be successful when systematically planned in conjunction with other school responsibilities.** This involves: |
| 1a | Consultation with stakeholders, which involves:   - providing information on the programme - eliciting views on ‘readiness for change’ as a starting point for engagement - encouraging the sharing of previous experiences of delivering health promotion programmes - facilitating discussion about the concordance (‘fit’) of the programme with:   - current practice  - students’ interests  - current school policies, resources and organisation  NR |
| 1b | Identification of a potential health benefit for pupils at a local level  NR |
| 1c | Consultation with stakeholders (head-teacher, school staff, pupils, parents, governors)  NR |
| 1d | Consideration of the concordance of the programme with current practice and interests  NR |
| 1e | Identifying clear aims and priorities, including intended outcomes  ‘The organiser convened a working group to plan the family event and classroom preparation. Aims and learning objectives identified for the classroom component in the teachers’ pack are included at Table 1. The organiser identified KAT’s long-term aim as reducing the number of young people who drink too much and then become involved in antisocial behaviour and crime; and the short term objective as “for parents and children to openly recognise and discuss the issues.” The long-term aim was clear to all the working group members but KAT objectives were not shared among members of the working group and the organiser. Although minutes of a meeting held in June 2007 recorded objectives concerning attitudes, knowledge and skills relating to alcohol use, interviews with five members of the working group suggested that this did not capture how they expected the programme to achieve its aim. Interviewees mentioned a range of short-term objectives and only two mentioned encouraging family communication about alcohol. Other objectives included changes in parents’ drinking behaviour and providing help for schools in delivering the PSE curriculum.’ (p.3) |
| 1f | Taking into account the current situation and competencies in a school and the implications of these for programme refinement  NR |
| **2 - Introducing a programme** | **The introduction of a health promotion programme to a school is more likely to be successful when it is incorporated into school activities through:** |
| 2a | Being integrated into school policy (e.g. a School Improvement Plan) and supported by governors and senior staff to whom monitoring and progress reports are made  NR |
| 2b | School staff using their leadership skills to co-ordinate activities or resources for programme delivery  NR |
| 2c | Providing adequate opportunity and/or training for the personal and professional development of those who will deliver the programme and engaging with their interests  NR |
| 2d | Modes of delivery that appeal to and engage pupils and provide cognitive and/or emotional rewards  ‘Overall, KAT achieved high levels of acceptability among children, parents and school staff. Children had enjoyed KAT, particularly the classroom preparation which they described as ‘fun’ and as different from normal school work. The children also enjoyed learning about issues relating to alcohol. Parent interviewees liked the fun evening, saying it was interesting, nonjudgmental and informative. They enjoyed the informality of the evening:’ (p.7-8)    “[...] it was a fun event, you know? You can go along to things, can’t you, for smoking or whatever and it’s going to be really serious and you know, you mustn’t do this and you mustn’t do that. But it was all fun and everybody was involved and nobody sort of felt the finger pointing at them. (parent, first interview)” (p.8)    Survey responses from parents (n=16-18) suggested approval and enjoyment of the ‘alcohol awareness evening’, with at least 85% agreeing with the responses ‘I liked everything about it’, ‘I enjoyed seeing the displays of children’s work’, ‘It was good to talk openly about alcohol issues’, and ‘I would like to go to another KAT event’. (p.8)  ‘The DVD helped to extend the influence of the programme beyond the school-based components. Children in two focus groups (1 and 2) at S1 said they had been keen to watch it and for friends and family members to join them; and two parents (M5 and M6) reported that their children had watched the DVD more than once. One child had left the DVD ready for her parents’ friends to watch when they visited her home (M1). The children had talked at home about what happened in the DVD, . . . ‘(p. 10)  ‘The classroom preparation appeared to be effective in promoting communication about alcohol issues amongst members of the class, and five parents (M1, M6, M7, M8, and M9) said their children had talked about it at home. However, two parents (F1 and M10) reported that children had ‘mentioned’ the class work but no more, and five parents (M2, M3, M5, M7 and M11) said their children had said nothing to them. A child in one focus group (FG7) said they had deliberately ‘kept it quiet’ so that it would be a surprise at the fun evening. Most children were very keen to go to the fun evening, to show off their work, to see what it was like and to enjoy the refreshments and entertainment.’ (p. 10)  ‘Four parents (M1, M4, M7 and M10) said their children had put pressure on them to attend:’  “I went along because [child] was saying ‘We’re having this evening, you’ve got to come, Mam’. Otherwise I might not have gone because personally I wouldn’t have felt I needed to be aware of alcohol because I’m very aware of it. (parent, second interview)” (p.10)  ‘Children in four focus groups talked about having ‘made’ or ‘forced’ their parents to go, and two parents (M8 and M9) who did not seem to have been pushed into it said they went simply because their children were keen to go.’ (p. 10) |
| 2e | Providing support materials that are appealing and appropriate to students’ age, interests and culture  NR |
| **3 – Embedding a programme into routine practice** | **The introduction and routine delivery (‘embedding’) of a programme takes time and motivation. It is likely to involve changes in the school environment and the development of new relationships between stakeholders that require pro-active management so that:** |
| 3a | - different stakeholders’ goals are reconciled - organisational decisions in other areas of school life are made taking into account how they impact on programme delivery - school staffs’ existing relationships with children are built upon - stakeholders’ enthusiasm, knowledge and experience are harnessed - knowledge of ‘core’ and ‘peripheral’ elements and minimum resources, skills and informational content is retained - responsibility for programme delivery becomes rooted in the school   NR |
| 3b | The longer-term sustainability of programmes and the extent to which health promoting messages and activities permeate other aspects of school life, is dependent on continuing feedback, encouragement, and expectations about implementation. Over time, this may originate less from outside the school and more from inside.  NR |
| **4 - Programme adaptation** | **The introduction, initial delivery, and ongoing sustainability of a health promotion programme in a school is more likely to be successful when there is:** |
| 4a | Specificity about ‘core’ (essential) and ‘peripheral’ (optional/adaptable) programme elements, including the minimum levels of resources and/or skills necessary to support these elements (to inform decision-making about how to deliver a programme in the context of a particular school)  NR |
| 4b | Scope for ‘mutual adaptation’ between the programme and the people delivering it, including the evolution and updating of programme content and mode of delivery over time  NR |

| **HP Programme** |  |
| --- | --- |
| Name of programme | NE Choices |
| Area of HP | Substance use (drug, alcohol) |
| Authors (year/ study type) | Stead et al. (2001/ CBA/ process evaluation) |
| Quality appraisal | Detailed descriptions of implementation processes, but link between data and findings often not made clear. Sampling strategy for interviews, focus groups, and workshop/classroom observations not reported. Data collection and analysis processes not adequately reported. |
| Programme description | ‘NE Choices is a multi-component drugs prevention programme for young people in the North-East of England. Based on a ‘social influences approach’ to drugs prevention, developed using social marketing principles / techniques. Integrated intervention components were an all day out of school drama workshop [supported by video and software] (Year 10), classroom support and follow-up (for workshop), six month youth work projects (Year 11), intensive outdoor activity programme for ‘high-risk’ young people (Year 11), drama and drugs awareness sessions for parents, also training for teachers, youth workers and school governors.’ (p.1-2). Findings from formative evaluation in the first year of delivery was used to refine programme components. Teachers received 1-day training and a manual to support delivery. Year 10 pupils could volunteer to ‘devise and deliver a piece of drugs education work of their own choosing to peers’ (p.28). Two youth workers were recruited to provide 30 sessions (each of 3h), with some additional funding provided by LAs. Parents were involved in order to ‘identify and explore their drugs information and other needs… [findings were used to] inform the development of a drama session for parents focusing on the theme of parent-child communication.’ (p.28) |
| Year(s) delivered | 1997-1999 |
| Description of UK location(s) where delivered | Schools in Northumbria |
| **Research methods** |  |
| Theoretical approach | NR |
| Sample | NR |
| Participants | Pupils aged 13-16yrs/ parents, teachers, school governors, media, local community |
| Data collection | Observation of workshops (n=6) and classroom sessions (n=3)  Focus groups (pupils, age 14-15y) (n=8)  In-depth interviews (teachers) (n=10) |
| Analysis | NR |
| Time of follow-up | During programme delivery |
| **Evidence about programme theory#** |  |
| **1 - Preparing for implementation** | **Preparation for the introduction of a health promotion programme to a school is more likely to be successful when systematically planned in conjunction with other school responsibilities.** This involves: |
| 1a | Consultation with stakeholders, which involves:   - providing information on the programme (e.g. who and what is involved, evidence of effectiveness) - eliciting views on ‘readiness for change’ as a starting point for engagement - encouraging the sharing of previous experiences and knowledge of delivering health promotion programmes - facilitating discussion about the concordance (‘fit’) of the programme with:   - current practice  - pupils’ interests  - current school policies, resources and organisation  NR |
| 1b | Identification of a potential health benefit for pupils at a local level  NR |
| 1c | Consultation with stakeholders (head-teacher, school staff, pupils, parents, governors)  NR |
| 1d | Consideration of the concordance of the programme with current practice and interests  NR |
| 1e | Identifying clear aims and priorities, including intended outcomes  NR |
| 1f | Taking into account the current situation and competencies in a school and the implications of these for programme refinement  ‘In organisational terms, teachers were pleased with the support provided by Northumbria Drugs Prevention Team, particularly the provision of funding for supply cover to enable teachers to attend training. This was interpreted as a sign of genuine commitment to drugs education’ (p.48) |
| **2 - Introducing a programme** | **The introduction of a health promotion programme to a school is more likely to be successful when it is incorporated into school activities through:** |
| 2a | Being integrated into school policy (e.g. a School Improvement Plan) and supported by governors and senior staff to whom monitoring and progress reports are made  NR |
| 2b | School staff using their leadership skills to co-ordinate activities or resources for programme delivery  NR |
| 2c | Providing adequate opportunity and/or training for the personal and professional development of those who will deliver the programme and engaging with their interests  ‘The teacher training course and manual appeared to have increased teachers’ knowledge and confidence regarding their ability to address drugs in the classroom.  “It was good, the talk about the different types, hallucinogens and things I didn’t know. That prepared me ‘cos I did a lesson on that” (Teacher/Tutor)  “It’s really important that we’re bang up to date with what’s going on out there otherwise the kids will reject it. There was a good balance of input from some practitioners, giving us bang up to date information” (Co-ordinator)  “But having something that is well organised and constructive helps, like these packs with activities where it’s all written out for you, when you’ve got that you don’t have to do the actual preparation work, you can go at it a bit more enthusiastically” (Year 10 Tutor)  “I can see the benefit of that because it wasn’t preaching, it wasn’t where people were, ‘Drugs are wrong and you mustn’t take them’. It’s obvious that kids, especially in this area, are going to come across drugs in their everyday life, large majority of them. I think this is no-nonsense, bare-knuckle approach is probably the best way to do it, and I found that a little bit unusual but useful as well” (Year 10 Co-ordinator) (p.47)  ‘However, some teachers felt that some of the activities in the manual adopted a more explicit approach to drugs education than their school favoured; this was more the case in the denominated schools. One or two conducted no follow-up at all because they felt drugs education had been “rammed down pupils’ throats”’ (p.47)  ‘The necessity to meet deadlines, to complete a project on a pre-defined issue in a specified period of time and to deliver the project to peers, conflicted with young worker’s perceptions of peer education…’  “Peer education is about them doing if for themselves, not us saying ‘you’ve got to do this and you need to do that’…At the beginning I felt it was the process that was more important, but the further in I got, the more the emphasis started being on the outcome. And that’s not how I was sold it. I was sold it on the processes” (Youth worker) |
| 2d | Modes of delivery that appeal to and engage pupils and provide cognitive and/or emotional rewards  ‘Eighty-two percent of young people felt that drugs information was more interesting if created by young people for young people’ (p.35)  ‘The stand-alone format was perceived as less effective than the facilitated session as it failed to contextualise the Box [a walk-in interactive installation] and to encourage discussion and learning’ (p.35)  (Based on focus groups) Pupils perceived the actors in the workshop as treating them ‘with respect’ – ‘… meaning not only that the actors encouraged them to speak their own minds and treated their opinions as valid, but also that they could be trusted not to criticise or betray confidences – an important perceived difference compared with teachers.’ (p.42)  ‘The Factfile seems to have been reasonably well received with the majority considering it to be easy to read (80%), liking the appearance of it (61%) and considering it to have been written by someone who understands people of their age (58%). Approximately half (48%) thought that it told you everything you need to know about drugs. A minority expressed negative views of the Factfile, considering it to be boring (15%) and stating that they would never look at it again (14%). Males (p<0.05) and intending drug users (p<0.001) were the least positive in their opinion of the Factfile’ (p.46-47)  ‘A majority (91%) had received the CD-Rom D-Code. Nearly three-quarters (72%) had played the music tracks, but only around a third had played all or some of the drugs information game. Since the drama workshop, a tenth of respondents had visited the NE Choices website’ (p.47)  ‘Initial expectations that the project would continue many of the most enjoyable features of NE Choices - a focus on drama, engaging facilitators who treated the participants “like friends” or “like adults”, and access to good quality materials - were met’ (p.49)  ‘There was high recall of the two projects, both of which were perceived as novel, engaging and more credible than standard classroom approaches to drugs education’  “If the teachers had done it would have been really boring” (Year 11)  “They were using words that we use, if they’d used other words we wouldn’t have, like got it”  “Normally it’s like ‘don’t take drugs’…they were telling you the consequences of doing drugs”  “They were getting across that drugs are bad, but they were saying at the end of the day it’s your life”  “I would have thought they’d have actually said ‘don’t take drugs’ – I was quite surprised at that” (Year 9) |
| 2e | Providing support materials that are appealing and appropriate to pupils’ age, interests and culture  ‘In three of the projects, participants had initially been strongly anti-drug or held stereotypical views such as “drug users are bad” or “touch drugs and you end up dead”. Youth workers were concerned that these naϊve views would lead them to produce a peer education product that lacked credibility, and therefore spent several weeks exploring different viewpoints. Consequently, youth workers had spent more time than they had planned for on dispelling myths and building basic drugs information, before the group could begin to consider peer education ideas. As a result, several projects began with several weeks of drugs education, which slowed project development but was felt to be essential to the peer-education purpose.  “The biggest problem that we had was we perhaps assumed the kids’ knowledge was better than it actually was…We thought they were a long way off passing the information onto other younger people” (p.50) (Youth worker) |
| **3 – Embedding a programme into routine practice** | **The introduction and routine delivery (‘embedding’) of a programme takes time and motivation. It is likely to involve changes in the school environment and the development of new relationships between stakeholders that require pro-active management so that:** |
| 3a | - different stakeholders’ goals are reconciled - organisational decisions in other areas of school life are made taking into account how they impact on programme delivery - school staffs’ existing relationships with children are built upon - stakeholders’ enthusiasm, knowledge and experience are harnessed - knowledge of ‘core’ and ‘peripheral’ elements and minimum resources, skills and informational content is retained - responsibility for programme delivery becomes rooted in the school   NR |
| 3b | The longer-term sustainability of programmes and the extent to which health promoting messages and activities permeate other aspects of school life, is dependent on continuing feedback, encouragement, and expectations about implementation. Over time, this may originate less from outside the school and more from inside.  NR |
| **4 - Programme adaptation** | **The preparation for, introduction, initial delivery, and ongoing sustainability of a health promotion programme in a school is more likely to be successful when there is:** |
| 4a | Specificity about ‘core’ (essential) and ‘peripheral’ (optional/adaptable) programme elements, including the minimum levels of resources and/or skills necessary to support these elements (to inform decision-making about how to deliver a programme in the context of a particular school)  NR |
| 4b | Scope for ‘mutual adaptation’ between the programme and the people delivering it, including the evolution and updating of programme content and mode of delivery over time  ‘Teachers were encouraged to adapt these [lesson plans and worksheets] to suit their varying curricula and methods. In practice, schools delivered varying amounts of follow-up … brief discussion in one school to eight hours in another’ (p.30)  ‘Follow-up sessions were structured into the curriculum in different ways … weekly 40 minute tutorial programme…in the specialist PHSE programme’ (p.31)  ‘Overall, the evaluation of this element of the intervention suggests that there may be limits to the consistency that can be achieved in schools, even where a programme is supported by standardised materials and training and by a robust and highly consistent core component (the drama workshop)’ (p.31) |

| **HP Programme** |  |
| --- | --- |
| Name of programme | PhunkyFoods programme (PFP) |
| Area of HP | Healthy eating and physical exercise |
| Authors (year/ study type) | Teeman et al. (2008/ BA process evaluation) |
| Quality appraisal | Data analysis not reported – lack of assurance regarding validity of data interpretation |
| Programme description | ‘The PhunkyFoods Programme (PFP) is an initiative launched in 2005 by Purely Nutrition, teaches primary children key messages related to healthy eating and physical exercise in a light hearted and fun manner through art, drama, music, play and practical experience with food. It aims to enhance pupil performance, increase concentration, and improve behaviour, motivation and self-esteem. Lesson plans are available to teach a one-hour lesson of healthy eating and a one-hour lesson of physical activity every week to every primary year group, although schools can deliver the PFP in a variety of ways (in different curriculum contexts and/or during out of hours school clubs) and for varying lengths of time. In addition to training for teachers and classroom assistants (supplemented by extensive online resources), support is provided through various resources such as DVDs, books and games, which schools receive when they join the PFP.’ (p.1) Each school had a named PFP co-ordinator. |
| Year(s) delivered | NR |
| Description of UK location(s) where delivered | NR |
| **Research methods** |  |
| Theoretical approach | NA |
| Sample | NR |
| Participants | Children across all years n=NR |
| Data collection | Exploratory telephone survey n=108 – information about future visits for case study evaluation. Also, how schools were intending to implement the PFP  In-depth case study work n=20 primary schools:  First visit between November 2007 to March 2008 – to assess schools’ ‘starting-points’, their expectations of the PFP (in relation to process and impact) and their plans for implementation  Interviews (second visit) n=19 schools – semi-structured interviews (37 staff - 8 headteachers/deputy head , 18 PFP coordinators, 5 teachers, 6 teaching assistants & 18 parents/carers, 117 pupils) |
| Analysis | NR |
| Time of follow-up | NR |
| **Evidence about programme theory#** |  |
| **1 - Preparing for implementation** | **Preparation for the introduction of a health promotion programme to a school is more likely to be successful when systematically planned in conjunction with other school responsibilities.** This involves: |
| 1a | Consultation with stakeholders, which involves:   - providing information on the programme (e.g. who and what is involved, evidence of effectiveness) - eliciting views on ‘readiness for change’ as a starting point for engagement - encouraging the sharing of previous experiences and knowledge of delivering health promotion programmes - facilitating discussion about the concordance (‘fit’) of the programme with:   - current practice  - pupils’ interests  - current school policies, resources and organisation  The following issues were identified in the authors’ analysis, but their extent not always quantified:  - lack of co-ordination between PFP and other school health promotion activities (i.e. overlap with PE lessons, other ‘healthy eating’ initiatives, or PSHE) (p.7) (‘some schools’)  - timetabling pressures and (unspecified) ‘other pressures’ sometimes limited introduction and delivery of PFP (no. of schools where an issue NR) – interviews suggested there had been substantial variation in the extent to which the programme was delivered (p.19-20)  Lack of space and support staff perceived by school staff as ‘challenges’ to programme delivery (extent not quantified); schools did not always have kitchen facilities in which children could undertake the programme components which involved cooking. |
| 1b | Identification of a potential health benefit for pupils at a local level  NR |
| 1c | Consultation with stakeholders (head-teacher, school staff, pupils, parents, governors)  NR |
| 1d | Consideration of the concordance of the programme with current practice and interests  The following issues were identified in the authors’ analysis, but their extent not always quantified:  - lack of co-ordination in delivery of PFP across the school could mean needing to purchase additional PFP support materials, e.g. when PFP was delivered school-wide at the same time (‘some schools’)  - ‘Almost all’ school staff interviewed felt ‘that they had been introduced to the PFP too late in the school year to be able to incorporate it fully into their curriculum plans for the current year.’ (p. 7)  ‘Lack’ of concordance could also act to stimulate new thinking and activity related to health promotion within a school:  ‘Some schools said that they were developing a new topic-based or skills-based curriculum for the new school year, and that this was an opportunity to review how and where they could incorporate the PFP into it. Staff in one school said that the experience of using the PFP, and seeing how their pupils had responded to both PFP’s healthy eating and physical activity elements, had made them realise that they needed to re-examine and revamp their broader curriculum, which they now felt to be a bit dull and boring:  *“We realised we needed to make the curriculum a bit more interesting to children to make them more enthusiastic; teachers liked the practical element* [of the PFP] *and the fact that it was linked to the QCA schemes of work, so in future they would incorporate elements of it into their usual planning of lessons.”* PFP coordinator (p. 8)  The contribution PFP could make to attaining NHSS [National Healthy Schools Standard] was cited by school staff as a motivator for programme delivery, and for continuing the programme in the future:  *“We already had a focus on healthy eating, and we’re applying for healthy school status, so it’s complemented that very nicely.”* Teacher  *“We’re working towards NHSS and that’s one of the reasons (possibly the main reason) why we have subscribed to next year’s Phunky.”* Headteacher (p. 25)  ‘Overall, the case-study schools already had a range of different whole-school strategies in place to promote healthier lifestyles. Nevertheless, the programme was said to have raised the profile of health education and complemented existing health-related activities. According to interviewees, the presence of a dedicated health-related programme in the school supported existing drives to promote healthier choices and helped staff to deliver the key messages:’  *“It supports our Healthy Schools Award. We’re en route for that. It’s fitted in with our healthy school drive, SEAL assemblies - looking after ourselves and others, and national drives like 2 hours of PE.”* Headteacher  *“PhunkyFoods has brought more of a focus on health education; because it is a new resource, teachers have thought more about health education.”* PFP coordinator (p. 25) |
| 1e | Identifying clear aims and priorities, including intended outcomes  NR |
| 1f | Taking into account the current situation and competencies in a school and the implications of these for programme refinement  ‘The PFP was felt by staff to be particularly suitable for TAs to use in PPA time because of its detailed and structured lesson plans:  *“It’s that simple and that straightforward, you could pick it up just like that. A supply teacher could walk in and be able to do it how it should be done because it’s that simple to follow, because it’s broken down so well.”* Teaching assistant  *“If the plans weren’t so detailed, it would be a lot harder.”* Teaching assistant  *“The lesson plans are very very good. The resources and lesson plans are there for you. It would be fantastic as well if you really weren’t sure what you were doing. You could just teach the lesson plan that’s there. I think it’s a great resource.”* Teacher (p. 9-10)  ‘Two of the case-study schools had delivered the PFP solely through special health/fitness weeks, because they felt it was easier to fit the programme into a designated week rather than into an already crowded curriculum. Commenting  on the PFP, one teacher explained:’  *“... it’s one of those things, it’s lovely to have and to use, but unless you were doing a health week, you wouldn’t do an hour a day of it because you just don’t have the time usually to do that.”* Teacher  ‘Two other schools had also run healthy school weeks in addition to delivering the PFP throughout the year. Such weeks were seen by staff as an opportunity to invite parents/carers and governors into school, as part of the school’s effort to acquire or maintain NHSS. Where the PFP was delivered as a special event, it was also seen as an opportunity to involve everyone in the school, both staff and pupils, and to include both healthy eating and physical activities. Individual classes or year groups tended to focus on one particular topic of their choice.’  *“We wanted everybody to do it with how they felt comfortable, to take it to whichever level they wanted, and everybody just got into it with real gusto, they really went for it.”* PFP coordinator (p. 12) |
| **2 - Introducing a programme** | **The introduction of a health promotion programme to a school is more likely to be successful when it is incorporated into school activities through:** |
| 2a | Being integrated into school policy (e.g. a School Improvement Plan) and supported by governors and senior staff to whom monitoring and progress reports are made  NR |
| 2b | School staff using their leadership skills to co-ordinate activities or resources for programme delivery  - PFP co-ordinators were Teaching Assistants (TAs) in seven of the 18 schools – the TAs perceived that they did not have the power or influence to address issues (e.g. timetabling, other school activities) that limited PFP delivery (p.8)  - In 18/20 schools, before programme implementation it was perceived that there would not be time for delivery. Six months later, in 5 of these schools at least ‘some’ of the elements of the PFP curriculum were being delivered – in each of these 5 schools, ‘the PFP coordinator was a class teacher, and in general that person had disseminated information about the PFP at a staff meeting and had printed off information and lesson plans for other members of staff’ (p.8). Similarly - ‘Where coordinators also had a senior role in school, such as ‘healthy school coordinator’ or ‘enterprise coordinator’, it was more often the case that they had started to promote the adoption of the PFP throughout the whole school.’ (p.8) |
| 2c | Providing adequate opportunity and/or training for the personal and professional development of those who will deliver the programme and engaging with their interests  TAs were not confident in their abilities to supervise ‘large’ groups of children doing physical activity, so this programme component tended to be omitted. |
| 2d | Modes of delivery that appeal to and engage pupils and provide cognitive and/or emotional rewards  [After-school clubs] “*Phunky is something you can do with a mixed age group. The older ones help the younger ones. With a sports club I’d need to split it into two age groups. But Phunky is all-inclusive. All children can take part in it, whether or not they’re good at academic subjects or PE*” PFP coordinator (p. 10) [*RC – all inclusive nature of the programme*]  The PFP coordinator explained the club’s [After school club] popularity thus:  *“... it’s not just sitting down things, it’s a mixture of physical and practical activities, games and growing things, it’s ideal really.*” PFP coordinator (p. 11)  [Resources – lesson plans]  *“The planning really did get in there, went back to basics, started from the beginning, and helped children to understand why they should eat healthily.* (p. 13) Teaching assistant  [Delivering the PFP and what worked well]  ‘In part, because the majority of the case study-schools were delivering the PFP this year outside of the normal curriculum, whether it was through PPA time, golden time, after-school clubs or special events, the focus had been on looking to make lessons fun and exciting. According to interviewees, with few exceptions, the PFP lived up to their expectations:’  *“I know the children enjoy the activities. They love the practical things. Often they remember it more like that than if they’d done it in a D&T lesson. Some of the activities are the same as what they do in D&T but they remember it from Phunky rather than from the lesson.”* Headteacher (p.17)  ‘Growing vegetables, tasting new foods, and cooking were frequently mentioned as favourite activities:’  *“They love everything about* [the PFP], *especially the food. They don’t see they’re having a lesson on healthy eating and how they should be eating. They see it as fun. They’ve thoroughly enjoyed growing vegetables, looking after them, and looking at them to see how they’ve grown. Some of them don’t realise where food comes from, that carrots grow and don’t just come out of a bag in the supermarket. I think if parents see how much children get out of growing their own vegetables, they’ll want to be involved.”* PFP coordinator (p.18) |
| 2e | Providing support materials that are appealing and appropriate to pupils’ age, interests and culture  Range of support materials identified by some teachers as supporting childen who learnt in different ways, e.g.:  *“I think the best thing about Phunky is that there’s such a varied choice of things to use – like the plastic food, picture cards, discussion cards, things to do on the computer, there’s something for everyone; there isn’t any child that learns in a particular way that isn’t catered for. It’s not all the same kind of thing. Sometimes when you do a club there’s the worry that you’ll do the same thing all the time.”* Teacher (p. 17 - 18)  ‘Apart from one school which felt that there was a lack of exciting materials for older pupils, most interviewees said the resources were suitable for the age group they taught.  *“The children loved the flashcards because they were bright and colourful. Also because they had the words actually on the cards they were then able to copy some of them, so they were good for literacy as well as for healthy eating.”* Teaching assistant (p.16)  *“The children loved the interactive whiteboard – putting the body parts on – they loved the gory bits, the bits they could move, watch and follow.”* Teaching assistant (p.16)  Worksheets had a mixed response from staff. ‘Staff at one school thought they were a bit difficult for younger children and less able readers. Some felt that they were less exciting but necessary for pupils’ learning.’ (p.19)  Support could also be in a form to support those delivering PFP, e.g. “The additional support provided by the website and telephone helpline was also valued by schools who used it. For instance a PFP coordinator said that,”  “‘*A teacher asked me where to find something, and I didn’t have time to look, so I phoned the helpline and they told me exactly where*.’” (p. 19) |
| **3 – Embedding a programme into routine practice** | **The introduction and routine delivery (‘embedding’) of a programme takes time and motivation. It is likely to involve changes in the school environment and the development of new relationships between stakeholders that require pro-active management so that:** |
| 3a | - different stakeholders’ goals are reconciled - organisational decisions in other areas of school life are made taking into account how they impact on programme delivery - school staffs’ existing relationships with children are built upon - stakeholders’ enthusiasm, knowledge and experience are harnessed - knowledge of ‘core’ and ‘peripheral’ elements and minimum resources, skills and informational content is retained - responsibility for programme delivery becomes rooted in the school   NR |
| 3b | The longer-term sustainability of programmes and the extent to which health promoting messages and activities permeate other aspects of school life, is dependent on continuing feedback, encouragement, and expectations about implementation. Over time, this may originate less from outside the school and more from inside.  ‘Only two of the 18 case-study schools, delivering the PFP in some way this year, said that they were unlikely to do so next year. The PFP coordinator in one school said that this was because of other pressures to raise children’s attainment and to meet targets, which left no space in the curriculum for the PFP. In the other school, a TA had attempted to deliver the PFP to pupils throughout the school, and felt that preparing lessons for all year groups had involved too much time. A third school, which had delivered the PFP solely through a health week, was undecided about future plans.’ (p. 12) |
| **4 - Programme adaptation** | **The preparation for, introduction, initial delivery, and ongoing sustainability of a health promotion programme in a school is more likely to be successful when there is:** |
| 4a | Specificity about ‘core’ (essential) and ‘peripheral’ (optional/adaptable) programme elements, including the minimum levels of resources and/or skills necessary to support these elements (to inform decision-making about how to deliver a programme in the context of a particular school)  NR |
| 4b | Scope for ‘mutual adaptation’ between the programme and the people delivering it, including the evolution and updating of programme content and mode of delivery over time  *This data strongly suggests variability in delivery rather than any kind of planned approach to ‘mutual adaptation’. (MP)*  In 7 of the 18 schools, TAs delivered only the healthy eating component of PFP as:  • this was where they perceived the greatest need to be  • in part because school halls were generally in use for other activities  • in part because some TAs felt they would not be able on their own to control a whole class doing physical activities.’ (p. 9)  ‘Of the 18 case-study schools delivering the PFP, five were using elements of it within the curriculum, seven were delivering it through PPA time, and the remaining six schools were using it in after-school clubs or in one-off or occasional events such as health weeks or school assemblies. This was in line with the intentions expressed when the schools were first visited. However, there was considerable variation in the use made of the PFP, both within and between each of these broad categories of delivery.’ (p. 7) [*RC – varied use and delivery environments* ]  ‘At the time of the second visit, schools differed considerably in the extent to which they had actually incorporated the PFP into the curriculum. At one end of the spectrum, a school said that they were just dipping into parts of the programme. At the other end, a school had completely replaced its own health education programme with the PFP; each class was receiving a weekly health lesson, and individual teachers were using the physical activity lesson plans as they wished.  [Resources – lesson plans] ‘The 18 schools delivering the PFP differed considerably in how they used the lesson plans. Some schools were following the plans in sequence, and felt that this worked well because the planning and structure was in place and ready to use. TAs, in particular, said that they had valued the fact that they had needed to do ‘*little*’ additional planning or preparation.’  *“The lesson plans follow on. Each lesson goes over what you’ve learnt last week, so you can see if the children have remembered it. And they have remembered.”* Teaching assistant (p. 13)  ‘Where the PFP was being used with mixed age groups, most schools were using lesson plans for the youngest pupils and differentiating to meet older pupils’ needs.’  ‘Although one teacher commented that she would be happy with a few bullet points rather than whole lesson plans, the overwhelming majority of staff interviewed were very complimentary about the lesson plans:’  *“They give you step by step exactly what you should be doing and what the children should be learning from it. They’re very well laid out and planned for teachers to use – it’s not a huge hassle.”* Teacher (p.13)  **“***I think it’s a very good programme. It’s well structured. It’s built on each year and goes through the school. There is reinforcement without repetition. You can see the progression.”* PFP and healthy school coordinator  *“The activities we take from the website are really good, and they’re at the children’s level. So I’m not having to photocopy it and change it like you have to sometimes. They can get the activities done in the time, so they’re not having to rush it, or run out of time to get it finished.”* PFP coordinator (p.14)  ‘Some teachers, whilst following the lesson plans in general, said that they liked to incorporate some of their own ideas:’  “[The PFP] *is easy to use, but if I am honest I am using [the plans] like a guide, and I do adapt them a little bit and if I have got additional things I bring them in. But I do think they are easy to follow and I know what the outcome should be.”* PFP coordinator (p.14)  Some teachers valued the way that PFP could be ‘locally tailored’, e.g. ‘a teacher in a school in a fishing town not only brought in samples of fish but also used the topic to talk about the local industry, and took pupils on a visit to explore a fishing boat owned by the father of a child in her class.’ (p.14) |

| **HP Programme** |  |
| --- | --- |
| Name of programme | Project Tomato |
| Area of HP | Obesity |
| Authors (year/ study type) | Christian et al. (2012)/ process evaluation |
| Quality appraisal | Study focuses on measuring fidelity of implementation and ‘appreciation’ of deliverers and participants of the programme components – descriptive statistical analysis is appropriate and clearly conducted, but not theoretically-informed (despite the programme itself being theoretically-driven). Contextual information limited to demographics and locations rather than details directly relating to implementation. Questions remain over the reporting of the high survey response rates, where it is unclear whether these were equally distributed amongst the 24 schools or whether these rates are compounded (where more than one survey was conducted). |
| Programme description | Using the Theory of Planned Behaviour as a basis, the programme consisted of core and customisable elements designed to address the factors that initiate and maintain change in eating behaviours (familiarisation, repetition, activities, modelling and the environment).  Core elements: manual and 12 curriculum-related lesson plans  Customisable elements (headteachers could decide which to adopt): cooking lessons, growing club information, funding support, information to setup a Project Tomato team.  Support materials: Project Tomato kit bags, newsletters and parent handouts. |
| Year(s) delivered | 2007-2008 |
| Description of UK location(s) where delivered | NR |
| **Research methods** |  |
| Theoretical approach | NR |
| Sample | Randomised (cluster) |
| Participants | Children (8-9y), parents, teachers |
| Data collection | Survey – children (n=261, although this number includes responses to 3 different surveys); parents (n=120); teachers (n=19) |
| Analysis | Descriptive statistics |
| Time of follow-up | NR |
| **Evidence about programme theory#** |  |
| **1 - Preparing for implementation** | **Preparation for the introduction of a health promotion programme to a school is more likely to be successful when systematically planned in conjunction with other school responsibilities.** This involves: |
| 1a | Consultation with stakeholders, which involves:   - providing information on the programme (e.g. who and what is involved, evidence of effectiveness) - eliciting views on ‘readiness for change’ as a starting point for engagement - encouraging the sharing of previous experiences and knowledge of delivering health promotion programmes - facilitating discussion about the concordance (‘fit’) of the programme with:   - current practice  - pupils’ interests  - current school policies, resources and organisation  NR |
| 1b | Identification of a potential health benefit for pupils at a local level  NR |
| 1c | Consultation with stakeholders (head-teacher, school staff, pupils, parents, governors)  NR |
| 1d | Consideration of the concordance of the programme with current practice and interests  NR |
| 1e | Identifying clear aims and priorities, including intended outcomes  NR |
| 1f | Taking into account the current situation and competencies in a school and the implications of these for programme refinement  NR |
| **2 - Introducing a programme** | **The introduction of a health promotion programme to a school is more likely to be successful when it is incorporated into school activities through:** |
| 2a | Being integrated into school policy (e.g. a School Improvement Plan) and supported by governors and senior staff to whom monitoring and progress reports are made  NR |
| 2b | School staff using their leadership skills to co-ordinate activities or resources for programme delivery  NR |
| 2c | Providing adequate opportunity and/or training for the personal and professional development of those who will deliver the programme and engaging with their interests  NR |
| 2d | Modes of delivery that appeal to and engage pupils and provide cognitive and/or emotional rewards  NR |
| 2e | Providing support materials that are appealing and appropriate to pupils’ age, interests and culture  Support materials (including quizzes, snack boxes, games, etc.) were mostly ‘liked’ by parents and children. The ‘implementation scores’ of these materials were 35% (parents) and 56% (children), but it is unclear exactly what these percentages mean (although they are certainly ‘low’). This was despite the ‘appreciation’ score of these items being relatively high (children 73%; parents 76%). |
| **3 – Embedding a programme into routine practice** | **The introduction and routine delivery (‘embedding’) of a programme takes time and motivation. It is likely to involve changes in the school environment and the development of new relationships between stakeholders that require pro-active management so that:** |
| 3a | - different stakeholders’ goals are reconciled - organisational decisions in other areas of school life are made taking into account how they impact on programme delivery - school staffs’ existing relationships with children are built upon - stakeholders’ enthusiasm, knowledge and experience are harnessed - knowledge of ‘core’ and ‘peripheral’ elements and minimum resources, skills and informational content is retained - responsibility for programme delivery becomes rooted in the school   NR |
| 3b | The longer-term sustainability of programmes and the extent to which health promoting messages and activities permeate other aspects of school life, is dependent on continuing feedback, encouragement, and expectations about implementation. Over time, this may originate less from outside the school and more from inside.  NR |
| **4 - Programme adaptation** | **The preparation for, introduction, initial delivery, and ongoing sustainability of a health promotion programme in a school is more likely to be successful when there is:** |
| 4a | Specificity about ‘core’ (essential) and ‘peripheral’ (optional/adaptable) programme elements, including the minimum levels of resources and/or skills necessary to support these elements (to inform decision-making about how to deliver a programme in the context of a particular school)  ‘Core’ and ‘customisable’ programme elements were defined:  Core elements - Project Tomato manual & twelve curriculum-related lesson plans.  Customised elements - cooking; growing club information; funding support; information to set up a Project Tomato Team.  but implementation was low – 8 (of 24) schools did not implement *any* of the school-based (lesson) (core) components. Overall, 45% of curriculum-related lesson plans, 25% of the tasting sessions, 8% of the cooking club, and 8% of the gardening club were implemented. |
| 4b | Scope for ‘mutual adaptation’ between the programme and the people delivering it, including the evolution and updating of programme content and mode of delivery over time  ‘The customised elements were tailored to meet each school’s needs’ (p.3)  Headteachers could choose which ‘customisable’ elements to adopt. |

| **HP Programme** |  |
| --- | --- |
| Name of programme | RIPPLE |
| Area of HP | Sex and relationship education |
| Authors (year/ study type) | ^1^Oakley et al. (2004/ process evaluation)  ^2^Strange et al. (2006/ CRCT)  ^3^Forrest et al. (2002/ process evaluation)  ^4^Strange et al. (2002; HER 327-337/ process evaluation)  ^5^Strange et al. (2002; HER 339-349/ process evaluation)  ^6^Stephenson et al. (2004) |
| Quality appraisal | ^1^Focus of the paper is on how evaluation process can impact on programme delivery and reflections on the issues arising in conducting a process evaluation. Research methods detailed in linked papers (appraised separately).  ^2^Analysis conducted using combined process and outcome data (described a controversial, though guidelines followed)  ^3^Little information about implementation context, but data collection and analysis appropriately conducted. Large numbers of pupils included in focus groups.  ^4^Whilst statistical analysis of survey data is carefully and appropriately conducted, the low response rate of the personally-administered surveys and the lack of data on non-responders is not adequately addressed. Details of FG data collection and analysis are minimal or non-existent.  ^5^Conduct of FGs and data collection/analysis clearly documented and justified. The issue of non-participants in the FGs is acknowledged, but it remains unclear what impact this had on the findings.  ^6^Paper primarily reports trial findings; refers to linked papers for details of process evaluation methods. |
| Programme description | ‘Peer-led sex education. Peer-educators were trained by an external team to prepare classroom sessions aimed at improving the younger pupils’ skills in sexual communication and condom use, and their knowledge about pregnancy, STIs (including HIV), contraception, and local sexual health services. The peer-educators were also given support to prepare lesson plans and identify resources. Ongoing help was provided by teachers who organised suitable times for teams of peer educators to deliver three sessions of SRE to year 9 pupils. Teachers were not present in the classroom. Every session lasted around 1 h and used participatory learning methods and activities focusing on relationships, STIs, and contraception. These sessions replaced the usual teacher-led SRE delivered during personal, social, and health education in intervention schools. The control schools continued with their usual teacher-led SRE and they received £1800 to spend on anything except SRE.’ (Stephenson et al. 2004) |
| Year(s) delivered | 1998-1999 |
| Description of UK location(s) where delivered | Central and southern England, comprehensive and non-selective, from rural and urban areas  Intervention and control schools were divided into high, medium and low risk strata on the basis of, amongst other variables, eligibility for school meals, record of attainment of five or more GCSEs, and level of non-privately owned accommodation.  Eligibility for school meals - 11-50% (high strata), 4-10% (low strata)  Record of attainment of five or more GCSEs - 21-49% (high strata), 35-69% (low strata)  Non-privately owned accommodation – 23-60% (high strata), 20-38% (low strata) |
| **Research methods** |  |
| Theoretical approach | NR |
| Sample | Various |
| Participants | Pupils, peer educators, teachers and other school staff |
| Data collection | ^1, 6^Observations:  n=23 programmes of peer-educator training  n=57 sessions of peer-led SRE (13 intervention schools)  n=10 sessions of teacher-led SRE (4 control schools)  Focus groups - n=52 (year 9 pupils); n=18 (peer-educators)  Survey (pupils) n=7770 (1^st^ FU); n=6656 (2^nd^ FU)  Interviews (n=43/ key staff from all schools)  ^3^Focus groups (n=52 – no. of participants in each FG NR); Survey (n=7770)  ^4^Survey (peer-educators, before and after programme delivery) (n=268); Focus groups with peer-educators (n=NR)  ^5^ Focus groups with peer-educators (n=18 – i.e. 18 FGs; no. of participants in each NR) |
| Analysis | ^4^Wilcoxon signed-rank test (to test for mean changes measured on Likert scales)  ^6^Continuous outcomes - differences in means, derived from linear regression; secondary comparative outcomes adjusted to account for pupil-level baseline factors (e.g. dislike of school and housing tenure) |
| Time of follow-up | 6 and 18 months  ^3; 4^At the end of the school year in which the programme was delivered |
| **Evidence about programme theory#** |  |
| **1 - Preparing for implementation** | **Preparation for the introduction of a health promotion programme to a school is more likely to be successful when systematically planned in conjunction with other school responsibilities.** This involves: |
| 1a | Consultation with stakeholders, which involves:   - providing information on the programme (e.g. who and what is involved, evidence of effectiveness) - eliciting views on ‘readiness for change’ as a starting point for engagement - encouraging the sharing of previous experiences and knowledge of delivering health promotion programmes - facilitating discussion about the concordance (‘fit’) of the programme with:   - current practice  - pupils’ interests  - current school policies, resources and organisation  Of the 343 potentially eligible schools approached (by letter), 222 (65%) did not reply and 72 (21%) were ‘not interested’. 49 ‘interested’ schools were visited by a study team member – 12 (25%) schools withdrew (e.g. ‘staff changes, too much pressure on timetable, , already doing peer-led SRE, strong preference for one trial arm’), 8 (16%) were excluded because of location (i.e. distance from London-based study team) (Stephenson et al. 2004)  Fieldnotes documenting pre-allocation discussion with teachers (in 23 of the 29 schools; 13 intervention, 10 control) recorded the following teacher preferences for allocation:  Intervention schools  Peer-led SRE – 9  Teacher-led SRE – 2  No preference – 2  Control schools  Peer-led SRE – 6  Teacher-led SRE – 1  No preference – 3  Authors conclude that ‘the greater engagement of both students and teachers in the experimental as compared with the control schools with the RIPPLE study raises the possibility that the impact of ‘being studied’ may have been greater for participants in the experimental arm.’ (Oakley et al. 2004, p.456) |
| 1b | Identification of a potential health benefit for pupils at a local level  NR |
| 1c | Consultation with stakeholders (head-teacher, school staff, pupils, parents, governors)  NR |
| 1d | Consideration of the concordance of the programme with current practice and interests  NR |
| 1e | Identifying clear aims and priorities, including intended outcomes  NR |
| 1f | Taking into account the current situation and competencies in a school and the implications of these for programme refinement  ‘Other factors cited as determining the quality of teacher-led sex education were . . . the space given to sex education in the timetable’ (Oakley et al. 2004, p. 454)  Pupils identified factors that made it more difficult for peer-educators to deliver SRE, although the extent of these factors is not clear from the reported data:  - inappropriate classroom space (e.g. science laboratories)  - ‘lack of support for the programme by some teachers’  - ‘lengthy gap between the training of peer-educators and delivery of SRE’  - timetable clashes for peer-educators taking exams  (Oakley et al. 2004, p.453)  Reasons cited by authors (on basis of process data as a whole) for some classes in two schools not receiving any peer-led SRE at all:  - ‘peer-led sessions scheduled too near the end of term and some were then cancelled’  - ‘peer-educators were disorganised and lacked enthusiasm’ – ‘In one school this was influenced by low morale as a result of a sixth form closing down, which in turn resulted in few students available to volunteer as peer-educators. In the other school, weaknesses in the wider school management, the threat of school closure and staff sickness resulted in a lack of continuity with regard to the staff responsible for co-ordinating the peer-led training programme.’ (Oakley et al. 2004, p.450) |
| **2 - Introducing a programme** | **The introduction of a health promotion programme to a school is more likely to be successful when it is incorporated into school activities through:** |
| 2a | Being integrated into school policy (e.g. a School Improvement Plan) and supported by governors and senior staff to whom monitoring and progress reports are made  ‘Other factors cited as determining the quality of teacher-led sex education were support for sex education from the senior management team.’ (Oakley et al. 2004, p. 454) |
| 2b | School staff using their leadership skills to co-ordinate activities or resources for programme delivery  10% of peer-educators felt that they were not supported by teachers, e.g. in accessing resources for peer-led sessions; advising on classroom management; no recognition of the value of what peer-educators were doing. However, 34% *did* feel they were well supported. (Strange et al. 2002; HER p.339-349) |
| 2c | Providing adequate opportunity and/or training for the personal and professional development of those who will deliver the programme and engaging with their interests  Pupils pick-up on teachers who are not engaged with the topic, which diminished pupils enjoyment of SRE:  “... it’s not their subject is it, PSE? They’ve got to do it whether they’re like PE or geography... I mean, with their subject they’ve obviously got compassion in it, because they’re teaching it, but with PSE they’re just like they’ve got to do it.” (female pupil, age 13y) (Forrest et al. 2002)  Peer-educators reported ‘additional ways’ that they felt they had benefited by being involved in the programme – feeling more confident about teaching classes, speaking to large groups, presenting information, talking about sensitive issues and being more patients. ‘Some mentioned that they felt it had extended the range of career options open to them [61% agreed in survey], while others observed that taking part in the programme had helped them to make new friends and increase their respect for teachers.’ (p.333)  Positive aspects of being a peer-educator identified in survey:  ‘Agreed or strongly agreed useful to school studies’ 38% (females more likely than males to agree strongly (p=0.038))  ‘Agreed or strongly agreed useful to life outside of school’ 66% (females more likely than males to agree strongly (p=0.025))  Negative aspect of being a peer-educator identified in survey:  ‘Agreed or strongly agreed that being involved in the project interfered with studies’ 21%  (Strange et al. 2002, p.327-337) |
| 2d | Modes of delivery that appeal to and engage pupils and provide cognitive and/or emotional rewards  One intervention school did not implement RIPPLE ‘because it could not recruit enough peer-educators’ (no further information provided) (Stephenson et al. 2004)  ‘They [pupils] perceived peer-educators as having greater relevant expertise and respect for pupils, holding more similar values about sex, using familiar language, being less moralistic, and making the sessions fun’ (p. 343) (Strange et al. 2002; HER p.339-349)  ‘… focus group data showed that some girls had difficulty contributing or asking questions in mixed sex classes, and that participation was difficult when peer-educators were unable to engage boys or manage their behaviour.’ (p. 343) (Strange et al. 2002; HER p.339-349)  ‘Factors in the peer-led programme which inhibited students’ engagement included embarrassment on the part of peer educators, and difficulty in controlling classes. Although students frequently referred to difficulties with disruption in both peer-led and teacher-led classes, they were less critical of peer educators’ difficulties with managing classes than they were of teachers. This is likely to reflect their appreciation of peer educators’ lesser experience at managing classes. Some students felt that peer educators did not wish to ruin their relationship with students by imposing their authority or dispensing punishment.’ (Oakley et al. 2004, p. 453)  ‘Students who received sex education from peer educators were more likely than students in the control arm to report that the timing of their sex education was about right. However, more than half of students in both arms would have liked their sex education earlier.’ (Oakley et al. 2004, p. 454)  ‘Students identified the personal characteristics of sex educators and their ways of interacting with students as crucial. They perceived peer educators to have greater expertise and respect for students than teachers, described peer educators as more confident, empathetic, caring and trustworthy, less moralistic and patronizing, as holding similar sexual values and as using humour in the sessions, making these more fun. Students felt that peer educators provided more relevant, detailed information and that they were learning something new. Weaknesses of peer educators included some being ‘shy and embarrassed’ and some struggling to manage the disruptive behaviour of some students. Students also identified the use of skill based activities as important. These involved practising putting on a condom, and activities that involved moving around, working in small groups and discussion, all of which were elements in the peer-led programme (*Forrest et al., 2002*).’ (Strange et al., 2006, p. 338)  ‘…where participatory/active methods were used, the peer-led intervention increased the likelihood, compared to teacher-led sex education, of students using contraception at last sex (OR 1.49 (0.95,2.34)). Where participative/active methods were not used, the peer-led intervention reduced the likelihood of students using contraception at last sex (OR 0.54 (0.36, 0.80)) (p*<*0.001).’ (Strange et al. 2006, p. 341)  Pupils from ‘medium-risk’ intervention schools were more likely than those from ‘low-risk’ schools to be positive about their (peer) sex educators (OR 1.36 (95% CI 1.17, 1.59) and more likely to report receiving key information (OR 1.37 (95% CI 0.81, 2.31)) (Strange et al. 2006, p. 345) - in summary, ‘[Process and outcome data combined] students who were most socioeconomically disadvantaged, most dissatisfied with school, had lowest educational expectations and were most likely to report sex, were significantly less likely than others to report experience of many of these key dimensions of sex education or to report satisfaction with sex educators or key information. These data suggest that the peer-led programme may have failed sufficiently to increase engagement among those most at risk of poor sexual health.’ (Strange et al. 2006, p. 347)  Peer-led lesson more frequently provided novel and resonant SRE information (mentioned in 35/41 FGs) than the teacher-led lessons (mentioned in 4/10 FGs), e.g. information about STDs, condoms and contraception, how to use a condom effectively (p.201) (Forrest et al. 2002)  One school, with 75% British Asians in one intake year (the majority of whom were boys) were more positive about *teacher-led* lessons, in marked contrast to all other schools where pupils were ‘highly critical’ of teacher-led provision. Peer-led provision was valued by pupils for being able to ‘move around, talk, work in groups and touch contraceptives and condoms’ (p.202):  Pupil 1: “It’s better as an active subject, like the lessons where you put the condom on a cucumber... is... better than doing written work...”  Pupil 2: “and when you come to do it in a few years time you’ll... come back to the cucumber exercise and you’ll be laughing while you’re doing it sort of thing, going, ‘Oh I remember that’” (male pupils, aged 13y) (Forrest et al. 2002, p.202)  Other activities that pupils enjoyed included making posters, condom quizzes, ‘brainstorming’ in small groups, making presentations and run-around games – ‘some also highlighted the enthusiasm of peer educators... the imaginative use of resources, and opportunities for joking and laughter. Students thought teachers were suspicious of these kinds of activities, especially those which involved students working together because they might be an opportunity for digression and subversion’:  “I mean, some teachers are really against people working together and it’s like, ‘Work on your own’. They think that if you work together you might be talking too much and messing about.” (female pupil, 13y) (Forrest et al. 2002, p.202)  Statistically significant difference in ‘pupil enjoyment’ between peer-led and teacher-led provision (adjusted for gender) OR 2.15 (95% CI 1.58-2.90), i.e. peer-led provision was enjoyed more by pupils. ‘Fun was associated with a decrease in embarrassment and anxiety when talking about sex’ (p.203), but this could also occur in teacher-led provision – ‘Teachers who could introduce humour and tolerate or join in with joking were seen as much more effective as well as likeable.’ (p.203):  “I mean, the best kind of teachers are those who can mess about with you and be really open and still try to get the point across.” (male pupil, 13y) ((Forrest et al. 2002, p.203)  Teacher-led provision was largely perceived by pupils as moralistic and focusing on abstinence (until marriage) and STDs. (Forrest et al. 2002)  Peer-educators were perceived as respectful, in marked contrast to teachers – ‘they described being asked or invited to do things rather than being told, not being shouted at for talking or moving around and being talked to as equals’ (p.204):  “They [peer-educators] treated us as one of them really. Like, teachers say they’re always looking down on you – saying, you done that wrong, you ask this stupid question but they seemed to treat us better...” (female pupil, 13y) (Forrest et al. 2002, p.204)  Peer-educators viewed their sessions to be ‘engaging’ for participants (age 13-14y) when they were practical (e.g. condom demo), involved games, moving around, learning something new, and ‘fun’. Small rooms/ rooms with fixed furnishings (e.g. science labs) were perceived as inhibiting this ‘active’ approach. (Strange et al. 2002; HER p.339-349)  Engagement with 13-14y pupils was perceived by peer-educators as being threatened by a lack of ‘fit’ between SRE sessions and other aspects of school life, e.g. scheduling late in the day (pupils tired); lengthy gaps between sessions; timetabling tensions around exam times of the school year. (Strange et al. 2002; HER p.339-349)  Statistically significant difference between intervention and control arms with regard to pupil satisfaction with SRE, i.e. pupils more satisfied with peer-led SRE (range 1-5 (less to more satisfied)):  Girls - 3.47 [SD 0.52] (I) vs. 3.30 [0.50] (C), p=0.001  Boys - 3.51 [SD 0.53] (I) vs. 3.33 [0.52] (C), p=0.0001  (Stephenson et al. 2004) |
| 2e | Providing support materials that are appealing and appropriate to pupils’ age, interests and culture  NR |
| **3 – Embedding a programme into routine practice** | **The introduction and routine delivery (‘embedding’) of a programme takes time and motivation. It is likely to involve changes in the school environment and the development of new relationships between stakeholders that require pro-active management so that:** |
| 3a | - different stakeholders’ goals are reconciled - organisational decisions in other areas of school life are made taking into account how they impact on programme delivery - school staffs’ existing relationships with children are built upon - stakeholders’ enthusiasm, knowledge and experience are harnessed - knowledge of ‘core’ and ‘peripheral’ elements and minimum resources, skills and informational content is retained - responsibility for programme delivery becomes rooted in the school   NR |
| 3b | The longer-term sustainability of programmes and the extent to which health promoting messages and activities permeate other aspects of school life, is dependent on continuing feedback, encouragement, and expectations about implementation. Over time, this may originate less from outside the school and more from inside.  NR |
| **4 - Programme adaptation** | **The preparation for, introduction, initial delivery, and ongoing sustainability of a health promotion programme in a school is more likely to be successful when there is:** |
| 4a | Specificity about ‘core’ (essential) and ‘peripheral’ (optional/adaptable) programme elements, including the minimum levels of resources and/or skills necessary to support these elements (to inform decision-making about how to deliver a programme in the context of a particular school)  NR |
| 4b | Scope for ‘mutual adaptation’ between the programme and the people delivering it, including the evolution and updating of programme content and mode of delivery over time  ‘Considerable variation’ in the training of peer-educators – ‘… in one school the peer-educators received just one school-based training session, whereas in another the peer-led programme was incorporated into the sixth-form core-studies programme, and the peer-educators received more than six school-based sessions’ (Oakley et al. 2004, p.448) |

| **HP Programme** |  |
| --- | --- |
| Name of programme | Schools on the Move |
| Area of HP | Physical activity |
| Authors (year/ study type) | Stathi et al. (2006/ BA with process evaluation) |
| Quality appraisal | Data collection procedures and use of recognised inventories in survey promise much, but sampling strategies and data analysis lack rigour and authors’ critical reflections on the study design and conduct are similarly weak. Quantitative analysis of survey data is limited to descriptive statistics and analysis of qualitative interview data is also descriptive and not theoretically-integrated. |
| Programme description | Website – ‘through which pupils can enter daily pedometer readings and track their personal step-count progress. Teachers also have access to the site, enabling them to track progress of individuals, classes and year groups and to target intervention accordingly. Lead teachers within each school (who are not always PE teachers) receive training before pedometers are distributed to pupils. The training covers familiarisation with website resources, ideas for embedding physical activity into the curriculum and whole school ethos. Lead teachers are expected to cascade the training to teachers of participating classes within their respective schools, and to encourage colleagues to register as pedometer-wearing participants themselves.’ <http://www.pathsforall.org.uk/component/option.../task,doc_download/> (Inchley, Cuthbert (2007) ‘An investigation of the use of pedometers…’)) |
| Year(s) delivered | NR |
| Description of UK location(s) where delivered | Participating schools (n=56)  Case study schools (n=6):   - Worthing (participants registered at each school n=303) - Tynemouth (n=60) - Dagenham (n=94) - Mansfield (n=263) - Leicester (n=346) - Wolverhampton (n=31)   Chosen on the basis of gender (2 single-sex, and 4 co-educational schools), age (3 junior and 3 secondary schools) and region of the country (urban and more rural location, north and south, and variety in socio-economic status).  Pupils from case study schools (n=490 - 197/boys & 293/girls)  The age range was between 10 and 13 years (117 students/10 yrs, 163/11 yrs, 182/12, 24/13 (Mean age=11.28 yrs old; SD = 1.00). |
| **Research methods** |  |
| Theoretical approach | NR |
| Sample | “Six of the 54 participating schools were chosen as case study schools. Schools were chosen on the basis of gender (2 single-sex, and 4 co-educational schools), age (3 junior and 3 secondary schools) and region of the country (urban and more rural location, north and south, and variety in socio-economic status).” (p.9)  Focus group participants were recruited to ‘fully represent’ gender, age, ethnicity and activity levels (p.13) |
| Participants | NR (only evaluation participants stated) |
| Data collection | - Questionnaire (n=322) –general opinions about the Schools on the Move Project; more specific opinions about the Schools on the Move Project (Intrinsic Motivation Inventory (IMI) (Ryan, 1982)  - n=14 ‘individual, semi-structured interviews were conducted with teachers from the six participating schools, to explore their experiences and perceptions of the project and how it has impacted upon their knowledge, understanding and behaviour. Participants were asked to share their views on the project, the constraints of trying to become more physically active, their attitudes and strategies towards the maintenance of an active lifestyle and their suggestions and recommendations for further development of the project’ (p.13)  - n=10 focus groups (pupils 3-6 in each group) - exploring the students’ views and experiences of walking and physical activity, physical activity and active lifestyles (including where appropriate, the lifestyles and activity of other household members) |
| Analysis | Questionnaire – descriptive (means, standard deviations, frequency counts)  Interviews – ‘transcripts were coded and common themes identified… based on deductive and inductive analytical procedures. This included scanning the data for categories and relationships among the categories, developing working typologies based on an examination of initial cases, and modifying and refining these on the bases of subsequent cases. A detailed description of responses was shared between researchers to enable similarities and differences to be identified and agreed. Member checks were used to validate preliminary findings.’ (p.14) |
| Time of follow-up | NR |
| **Evidence about programme theory#** |  |
| **1 - Preparing for implementation** | **Preparation for the introduction of a health promotion programme to a school is more likely to be successful when systematically planned in conjunction with other school responsibilities.** This involves: |
| 1a | Consultation with stakeholders, which involves:   - providing information on the programme (e.g. who and what is involved, evidence of effectiveness) - eliciting views on ‘readiness for change’ as a starting point for engagement - encouraging the sharing of previous experiences and knowledge of delivering health promotion programmes - facilitating discussion about the concordance (‘fit’) of the programme with:   - current practice  - pupils’ interests  - current school policies, resources and organisation  Note that: ‘Most schools had a clear strategy on healthy living and healthy lifestyles which is evidenced by their participation in the project’ (p.24) – authors claim (although present no data to back-up) that such ‘whole school approaches’ ‘played a significant role in pupils’ participation’ because teachers were largely enthusiastic and activities in the programme ‘fitted’ with the schools’ approach (p.27)  ‘In some cases timetabling caused difficulties in the implementation of the project with teachers not having the time to use computers to enter data on the website. Other factors that hinder the implementation of the project were the time of the project as many classes had SAT’s exams and also the pedometers. Teachers mentioned that many students lost their pedometers and that made things more difficult’ (p.30)  ‘Teachers stressed that the programme implementation would not be an easy task due to timetabling and teaching responsibilities. They argued that support should be sustained if they were to maintain activity levels; especially when it comes to students’ engagement in physical activities’ (p.31) |
| 1b | Identification of a potential health benefit for pupils at a local level  NR |
| 1c | Consultation with stakeholders (head-teacher, school staff, pupils, parents, governors)  NR |
| 1d | Consideration of the concordance of the programme with current practice and interests  NR |
| 1e | Identifying clear aims and priorities, including intended outcomes  NR |
| 1f | Taking into account the current situation and competencies in a school and the implications of these for programme refinement  NR |
| **2 - Introducing a programme** | **The introduction of a health promotion programme to a school is more likely to be successful when it is incorporated into school activities through:** |
| 2a | Being integrated into school policy (e.g. a School Improvement Plan) and supported by governors and senior staff to whom monitoring and progress reports are made  NR |
| 2b | School staff using their leadership skills to co-ordinate activities or resources for programme delivery  NR |
| 2c | Providing adequate opportunity and/or training for the personal and professional development of those who will deliver the programme and engaging with their interests  NR |
| 2d | Modes of delivery that appeal to and engage pupils and provide cognitive and/or emotional rewards  Authors summarise pupil survey findings on motivations to take part in the project as:  ‘Most students reported that they participated in the project because they value the benefits of physical activity and they enjoy the exercise experience’. The authors claim that the more the following factors were present, the more that pupils were ‘intrinsically motivated, enjoyed the project and found it interesting’ – however, only partial and summary data that cannot be explicitly linked to this claim is provided:  - felt competent regarding the tasks in the project  - took part out of free will  - felt that the project was valuable and useful  - felt meaningfully related to other participants in the project  - perceived talk about physical activity to have increased (p.22)  ‘The findings from the students’ interviews suggest that the project motivated the students to become more active and increased awareness of the importance of a healthy lifestyle and the role of physical activity. The schools’ ethos played an important part in students’ participation and engagement with the project’ (p.24)  Pupils engaged with the programme because it was fun:  “… I liked it because we went on walks with our teacher. We went like round the school and the fun and games on the playground were fun” (pupil, age NR)  … and also because activities could involve their friends and peers:  “… every day me and my friend go round the playground once or twice running around for a bit… if I had to do it on my own I probably wouldn’t have done it.” (pupil, age NR)  ‘Students suggested that the website was a motivating factor to maintain activity. More specifically, recording steps on the web was a good way of monitoring performance and increasing activity levels... They talked about setting up step targets …‘*I thought that the website was good because you could find out your steps and your target, next target to reach…*’. In addition students commented on the structure of the website. They explained how they enjoyed the games, the colours and also the certificate given. (p.26) Also, teachers ‘…mentioned that recording steps and receiving new targets and certificates increased students’ engagement in the project’ (p.31) |
| 2e | Providing support materials that are appealing and appropriate to pupils’ age, interests and culture  NR |
| **3 – Embedding a programme into routine practice** | **The introduction and routine delivery (‘embedding’) of a programme takes time and motivation. It is likely to involve changes in the school environment and the development of new relationships between stakeholders that require pro-active management so that:** |
| 3a | - different stakeholders’ goals are reconciled - organisational decisions in other areas of school life are made taking into account how they impact on programme delivery - school staffs’ existing relationships with children are built upon - stakeholders’ enthusiasm, knowledge and experience are harnessed - knowledge of ‘core’ and ‘peripheral’ elements and minimum resources, skills and informational content is retained - responsibility for programme delivery becomes rooted in the school   NR |
| 3b | The longer-term sustainability of programmes and the extent to which health promoting messages and activities permeate other aspects of school life, is dependent on continuing feedback, encouragement, and expectations about implementation. Over time, this may originate less from outside the school and more from inside.  NR |
| **4 - Programme adaptation** | **The preparation for, introduction, initial delivery, and ongoing sustainability of a health promotion programme in a school is more likely to be successful when there is:** |
| 4a | Specificity about ‘core’ (essential) and ‘peripheral’ (optional/adaptable) programme elements, including the minimum levels of resources and/or skills necessary to support these elements (to inform decision-making about how to deliver a programme in the context of a particular school)  NR |
| 4b | Scope for ‘mutual adaptation’ between the programme and the people delivering it, including the evolution and updating of programme content and mode of delivery over time  NR |

| **HP Programme** |  |
| --- | --- |
| Name of programme | SHARE - Sexual Health and Relationships: Safe, Happy and Responsible |
| Area of HP | SRE |
| Authors (year/ study type) | ^1^Wight et al. 2002/ CRCT, process evaluation  ^2^Wight et al. 1998/ CRCT, process evaluation (preliminary baseline)  ^3^Wight & Buston 2003/ process evaluation (teacher training)  ^4^Buston & Wight 2002/ process evaluation (female pupils)  ^5^Buston et al. 2002 (CHS 4)/ process evaluation (engaging pupils)  ^6^Buston & Wight 2004/ process evaluation (class variation)  ^7^Wight & Abraham 2000/ description of programme development  ^8^Buston et al. 2001/ process evaluation (teachers)  ^9^Buston et al. 2002 (HER 17)/ process evaluation (teachers)  ^10^Buston & Hart 2001/ process evaluation |
| Quality appraisal | ^1^ Authors acknowledge that paper reflects the early stages of the investigation Limited information regarding implementation context, lack of detail regarding how process evaluation was conducted and how material was analysed. No primary data provided regarding the process evaluation, therefore unable to assess credibility of statements regarding delivery of the intervention  ^2^No detail provided regarding data collection or analysis, only limited primary data available to assess the credibility of claims made  ^3^Criteria for purposive sample (interviews and lesson observations) clearly described, but sampling strategy for survey not reported. Substantial detail provided about data collection and analysis. Findings clearly contextualised.  ^4^Small sample size acknowledged. However, clear criteria for purposive sample, in-depth and reflective description of how data was collected in an enquiring and sensitive manner, and full and reflective description of analysis process. Authors acknowledge that ‘the sample discussed in this paper substantially over-represents those who have had sex at an early age’  ^5^Purposive (maximum variety) sample drawn from large ‘population’ of schools in the trial, using interim findings (from survey) as a basis. Data collection and analysis fully described. Study designed and conducted to address issues identified in previous research (e.g. impact of classroom setting and teenagers’ gender roles)  ^6^Data collection and analysis described in greater detail in linked papers.  ^7^NA  ^8^Thorough data collection and analytic process; context in which programme was implemented richly described; candid reflection on limitations of findings.  ^9^Inclusive sampling strategy, with no teachers declining to be interviewed. Analysis of qualitative interview data somewhat limited by using a largely pre-defined framework, but good discussion of potential impact of teachers ‘over-selling’ fidelity of programme delivery to satisfy closely-involved researchers. Rigour of lesson forms questionable, as these were not validated and inconsistencies in teachers’ self-assessments were not addressed.  ^10^Strongly grounded in authors’ arguments that an assumption of heterosexuality is incorporated in many schools’ SRE, resulting in a neglect of gay and lesbian pupils’ needs. This provides the focus for the data analysed in this study, but was not a focus for the trial and qualitative research from which the data is drawn. Data collection was extensive, both in terms of spread across the 25 schools and data collection methods used (surveys, interviews, observations of practice). |
| Programme description | ^1^The SHARE intervention (Sexual Health and Relationships: Safe, Happy and Responsible) is a five day teacher training programme plus a 20 session pack: 10 sessions in the third year of secondary school (at 13­14 years) and 10 in the fourth year (at 14­15 years). It is intended to reduce unsafe sexual behaviours, reduce unwanted pregnancies, and improve the quality of sexual relationships. The programme was developed and piloted in Scotland over two years in consultation with teachers, sex education specialists, and education and health promotion departments. The programme combines active learning (for example, work in small groups and games), information leaflets on sexual health, and development of skills, primarily through the use of interactive video but also through role playing. In the 12 control schools sex education for third and fourth years varied from seven to 12 lessons in total and was primarily devoted to provision of information and discussion.  ^7^The process of programme development – two pilots; the first in 4 Scottish schools, with 9 teachers (17 classes) – evaluated through observation of lessons, participant surveys and semi-structured interviews with teachers and pupils. Programme development commented on by 5 sex education experts and lead researchers of related evaluations. This ‘resulted in substantial changes to pilot materials... [allowing] practical constraints [to be] addressed in the design and piloting stage.’ (p.27). Second pilot in a further 4 schools (15 teachers, 23 classes) used same evaluation methods – this time, lead to smaller revisions to teachers’ pack/course. |
| Year(s) delivered | 1993-1996 (programme developed) 1996-1999 (programme delivered and evaluated) |
| Description of UK location(s) where delivered | Non-denominational, co-educational state schools (East Scotland)  ‘Schools were classified as having deprived, mixed or affluent catchments. Ten schools were classified as affluent (<11% pupils receiving free school meals); seven schools as mixed (11±25% pupils receiving free school meals); and six schools as deprived (>25% pupils receiving free school meals).’ (Buston & Wight 2004) |
| **Research methods** |  |
| Theoretical approach | ^1,4,5,6^NR |
| Sample | ^1^NR  ^2^NR  ^3^Purposive (interviews and lesson observations); NR (survey)  ^4^Purposive – ‘a mix of young women who reported they had or had not experienced sexual intercourse… and, for the younger age group only, reported liking or not liking school’ p.237)  ^5^Purposive:  - pupils aged 14y sampled on basis of survey responses so that ‘for each sex, half reported experiencing heterosexual intercourse and half did not; and half reported being disaffected from school and half did not… also aimed to interview pupils from several classes within each school in order that the sample included pupils taught by different teachers and from different kinds of classes’ (p.321)  - pupils aged 15-16y – ‘two schools were selected for their extensive delivery of sex education (one in each arm of the trial) and within them pupils were selected who had received a substantial component of sex education’ (p.321)  ^6^Purposive  ^7^NA  ^8^All Assistant Head Teachers or PSE co-ordinators with responsibility for SRE in the programme schools, and at least one member of staff involved with teaching SRE  ^9^NR  ^10^Purposive (class observations; interviews); comprehensive (surveys) |
| Participants | ^1^Teachers  ^2^Pupils (13-15y)  ^3^Teachers delivering SRE (from both SHARE and control schools)  ^4^Female pupils (14-16y)  ^5^Pupils (14-16y)  ^6^Teachers  ^7^NA  ^8^All Assistant Head Teachers or PSE co-ordinators with responsibility for SRE in the programme schools, and at least one member of staff involved with teaching SRE  ^9^Teachers  ^10^Teachers, pupils (13-15y) |
| Data collection | Questionnaires (pupils n=7630; teachers n=173)  ^1^Baseline questionnaires n=7616; two year follow-up n=5854  ^2^Baseline (pupils) – friendships, future expectations; drug use; perceptions of sex education; sexual experiences and cognitions in respect to sexual encounters  ^3^Start n=81, 1^st^ year n=78, year after n=69  Interviews  ^3^Teachers (n=27)  ^4^Female pupils – Schools n=6; 14yrs (n=16) 16yrs (n=14) – introductory questions (i.e. families, friend, what they liked doing), location of most of their ideas about sex, evaluate their school’s sex education, attitudes to sex and sexual health issues, romantic, sexual relationships/behaviour  ^5^Pupils n=33 (14 female, 19 male), schools n=6 – all three age groups were asked about context and processual aspects of their classroom-based sex education  ^6^Teachers (n=59) (n=35 teachers agreed; in 24 schools either a member of senior management or member of the Guidance team was interviewed) – delivery of sex education i.e. `what is/are the class(es) like that you have been doing sex education with?',  ‘are prior relationships helpful or unhelpful?', `overall, what difficulties, if any, would you say you encountered?' and `how does teaching vary across classes?'  ^8^Interviews (n=58)  ^9^Interviews (n=11 Assistant Head Teachers; class teachers (n=15)  Lesson ‘pro-formas’ (completed by teachers – structured reflection of delivery of SRE lessons) (n=109)  ^6^All teachers delivering sex education to S3 in the first year of the study and to S4 in the second year of the study were sent a lesson pro-forma for each class they taught…Over half completed the form.  ^9^n=1030 (i.e. 1030 sessions, just under 50% of the total no. of sessions delivered)  ^1^Group discussions (n=NR); ^4^Schools (n=4) – as above interviews (except details regarding relationships or how they learnt about sex and how it had been applied in their personal lives); ^5^Group discussion (n=16; approx. 6 pupils in each discussion) schools (n=4) – all three age groups were asked about context and processual aspects of their classroom-based sex education |
| Analysis | ^1^Power and sample size calculation, restricted randomisation test, regression  ^2^NR  ^3^In depth interviews – ‘coded according to researchers’ prior conceptual categories and themes plus themes emerging from initial readings of the text’ (p.527)  Questionnaires – open-ended questions coded, analysed using SPSS  Observations of lessons – notes on teachers’ delivery (performance, interaction with pupils, pupils’ response) were independently rated (very good, OK, poor) by two researchers; discrepancies in rating resolved by discussion  ^4^Interviews and group discussions tape recorded, transcribed, sorted into sub-categories, constant comparative method  ^5^Interviews and group discussions tape recorded, transcribed, compared for similarities and differences until substantive categories emerged, hypotheses then generated/tested and developed  ^6^Interview transcripts coded and analysed, re-siting excerpts in the context of original interviews to appreciate the contexts in which the comments were made. Lesson observation forms were given ratings (high, medium, low - *definitions provided*) ‘the lessons in each category were then looked at together’ (p. 289). For both interview and observational data, initial hypotheses were developed from comparison of sub-sets of cases which were systematically tested against all cases and either rejected, modified or confirmed  ^7^  ^8; 9^Iterative development of coding themes (interviews) to ‘reflect how teachers discussed the issues and conceptualised them’; factors were analysed ‘to understand their inter-relationships and relative importance in different schools’ (Buston et al. 2001, p.355)  ^9^Data double-entered and cleaned in SPSS; descriptive statistics only reported  ^10^ |
| Time of follow-up | Various – during programme development, delivery and post-delivery (as appropriate) |
| **Evidence about programme theory#** |  |
| **1 - Preparing for implementation** | **Preparation for the introduction of a health promotion programme to a school is more likely to be successful when systematically planned in conjunction with other school responsibilities.** This involves: |
| 1a | Consultation with stakeholders, which involves:   - providing information on the programme (e.g. who and what is involved, evidence of effectiveness) - eliciting views on ‘readiness for change’ as a starting point for engagement - encouraging the sharing of previous experiences and knowledge of delivering health promotion programmes   ‘Teachers’ aggregate confidence scores were not related to their sex, age or subject specialism. However, they were strongly related to the length of their prior experience teaching sex education and, more importantly, their experience with that specific element of sex education.’ (Wight & Buston 2003, p.531)  ‘Teachers reported themselves to be most confident in largely information-based activities, using small group methods and remaining true to their own values (Table IV).’ (Wight & Buston 2003, p.534)   - facilitating discussion about the concordance (‘fit’) of the programme with:   - current practice  - pupils’ interests  - current school policies, resources and organisation  Inclusive SRE could engender a negative or hostile reaction from pupils, manifesting as a refusal to engage in lessons, embarrassment and discomfort. Teachers largely found it difficult to overcome these responses:  “.. you see in the all-boys class the people that are more volatile don’t want anything to do with them [gay mean or lesbians]. You know it was very much like ‘that’s it, don’t talk about it’” (female teacher, aged 41-50y)  “... they were a bit embarrassed, you know [about the discussion about homesexuality]. Let;s face it, a homosexual’s not a homosexual, he’s a poofter.” (male teacher, aged 51-60y)  “... pupils at that age, it [same-sex sex] is just a no-no, it’s disgusting, how could they possibly? Especially boys. But, and if you say ‘well everyone’s entitled to their own views and live the way they choose’, they are just not comfortable with that at all. And I just don’t know how you could get that over.” (female teacher, aged 31-40y) (Buston & Hart 2001, p.103)  Pupil survey results re: whether ‘people should be free to have sexual relationships with people of their own sex’:  Strongly agree or agree – 48.8%  Unsure – 25.4%  Disagree or strongly disagree – 25.8%  (of those answering ‘strongly agree’, 57.6% were female, 39% were male)  (Buston & Hart 2001, p.104)  ‘Time for reflecting on the delivery of sex education, and for thorough preparation, was often scarce or non-existent, further compounding teachers’ sense that they were operating with little guidance.’ – school ethos was identified as structuring which topics were prioritised ‘on the potentially very over-crowded PSE curriculum.’ (Buston et al. 2001, p.359)  The way in which a novel SRE programme could be introduced varied depending on the details of the school’s organisational history, e.g. it might be limited if a member of teaching staff has had lead responsibility for SRE for many years and is reluctant to change the approach, or it might be enabled by a growing professional relationship between a Head Teacher and a School Nurse (over a number of years) (Buston et al. 2001, p. 362)  Some teachers reported that the extent (20 sessions over 2 years) of the SHARE programme could be very difficult to fit in with other PSE and educational requirements, e.g.:  “I don’t think I could usually fit in more than 6 [sessions a year]. I mean this [SHARE] has really distorted my whole programme… I’ve not done any study skills, I’ve not done anything on drugs or alcohol, I’ve hardly done any work experience.” (teacher) (Buston et al. 2002; HER, p.66). |
| 1b | Identification of a potential health benefit for pupils at a local level  NR |
| 1c | Consultation with stakeholders (head-teacher, school staff, pupils, parents, governors)  The priority (or not) afforded to SRE could depend to a significant extent on competition for time in the school day – for example, schools whose reputation was perceived to depend on a high university entrance rate:  “There is a lot of pressure from the community, from parents. There are parents who are very supportive but also very demanding and they demand that their children do well in exams because it is very important in [school] and the community that youngsters get their Highers and get to university, etc.” (male PSE co-ordinator) (Buston et al. 2001, p. 360)  In other instances, there may be a reluctance on the part of, e.g. headteachers, to sanction ‘non-traditional’ SRE that it was believed might offend parents (Buston et al. 2001)  The majority of schools had a ‘closed-door’ approach to SRE, i.e. parents were not consulted about the content or extent of SRE. Only 6 schools had an ‘open’ policy about SRE that endeavoured to engage with parents, e.g. materials displayed at parents’ evening, invitations to discuss SRE, formal notification (by letter) of SRE programme prior to delivery. However, ‘no simple relationships between a school’s openness with parents, the social class profile of the catchment, and provision’. Parental engagement was also hard to achieve – even in the schools with an ‘open’/’engagement’ approach, meaning that responses to parents’ views were predominantly based on ‘a very small number of parents or on what the Head Teacher perceived to be their collective view.’ (Buston et al. 2001, p. 361) |
| 1d | Consideration of the concordance of the programme with current practice and interests  Reports a delicate path being trodden when introducing the programme. The programme components were theoretically-informed (e.g. social-cognitive models; analyses of power in heterosexual relations; phenomenological research of safer sex practices), but these could conflict with teachers’ views based on their professional experience – ‘Delicate negotiations were required to achieve a successful compromise of social-cognitive theory and orthodox health education principles... [and translation of] research-based findings into educational and teacher-training materials in a manner which made these ideas accessible to teachers.’ (p.30). The role of the lead author of the teacher education pack (Dixon), ‘a highly respected sex education consultant... was critical to the credibility of the project with both teachers and education consultants.’ (p.30). Health Education Board for Scotland funding for development of the pack gave the programme credibility within schools, especially over contentious elements (e,g. condom handling). (Wight & Abraham 2000) |
| 1e | Identifying clear aims and priorities, including intended outcomes  NR |
| 1f | Taking into account the current situation and competencies in a school and the implications of these for programme refinement  ‘In three schools timetabling and the low priority attached to sex education meant that most of the pupils did not receive this minimum package. In six schools timetabling constraints and teacher mobility led to non­trained teachers delivering the programme to a small minority of classes.’ (Wight et al. 2002, p.4) |
| **2 - Introducing a programme** | **The introduction of a health promotion programme to a school is more likely to be successful when it is incorporated into school activities through:** |
| 2a | Being integrated into school policy (e.g. a School Improvement Plan) and supported by governors and senior staff to whom monitoring and progress reports are made  The extent to which inclusive SRE was integrated into schools’ policy was unclear, but teachers *perceived* a lack of support (from within the school and in terms of national policy) for delivering inclusive SRE:  “We are restrained by instructions from our bosses to an extent as to where you can go with it [discussing homesexuality]... teachers don’t have a free hand.” (male teacher, aged 31-40y)  This was compounded by ‘lack of support and/or guidance from senior management within the school and/or local authority, ... national policy pronouncements.. and fear of media “exposure”’ (Buston & Hart 2001, p.101)  Teachers were unclear about their legal position in regard to teaching inclusive SRE, i.e. they were unclear how they would be supported in delivering it:  “I’m probably in breach of contract by doing that [challenging homophobic comments made by pupils]” (female teacher, aged 31-40y)  “I think the law is quite vague about that issue [homosexuality]... I feel a huge failure on that.” (female teacher, aged 41-50y)  (Buston & Hart 2001, p.102) |
| 2b | School staff using their leadership skills to co-ordinate activities or resources for programme delivery  NR |
| 2c | Providing adequate opportunity and/or training for the personal and professional development of those who will deliver the programme and engaging with their interests  SHARE teacher training (n=81 teachers in six different groups; each group comprise teachers from 3-5 different schools) took place over five days and involved practicing the delivery of SHARE curriculum exercises and ‘discussing and analysing these experiences in a supportive environment’ (p.525):  ‘When interviewed… teachers frequently said that [the training course] had made them ‘more comfortable’ or ‘more confident’ [to teach SRE]’, e.g.  “I was fairly comfortable with a lot of the approaches already. And that made it an awful lot easier for me than some other people. Mm, one of the questions we had to practice… was ‘How do girls masturbate?’ and I did think I’d never be asked that… and I was really glad I’d had that kind of opportunity, because I have been asked that since teaching the course.” (Teacher) (Wight & Buston 2003, p.529)  ‘Several teachers talked about how ‘making us aware of our own perceptions and prejudices’ contributed to building confidence and reducing embarrassment, as well as making it easier to be non-judgemental when delivering sex education’, e.g.:  “But the one lesson that stuck in my mind was where we had to put [different sexual activities as ‘high risk’ or ‘low risk’]… because without doing that you can be caught short in class, if somebody suddenly comes up with something, you have got to be guarding your expression, you don’t want to be in any way judgemental, you should just be thankful that they have been able to say something in class.” (Teacher) (Wight & Buston 2003, p.529)  “To be able to familiarise ourselves with the material and to be able to work through it” ‘was the most commonly reported benefit of the training in the questionnaire and was widely mentioned in the teacher interviews. Participating in the *SHARE* exercises [during training] was said to be a particularly useful preparation, largely because it gave teachers an insight into what the pupils were likely to feel during the lesson’ (Wight & Buston 2003, p.533)  Some teachers found delivering inclusive SRE (i.e. ‘one that recognises that not all pupils have a heterosexual identity, normalises gay and lesbian identities and sexual behaviour, and provides information of relevance to all pupils’ (p.107) very difficult because of their own values:  “I’m never that comfortable talking about, or when being asked to talk about, homosexuality for example. Some people say that we should be teaching about homosexuality in schools, it should be a compulsory part of the sex education programme and I’m not comfortable with that. So, if you like, that is one of my hang-ups.” (male teacher, aged 31-40y) (Buston & Hart 2001, p.101)  Teachers were ‘divide’ over whether or not they should ‘bring some of themselves into the sex education classroom’ (through telling pupils of their stance on issues and/or relating stories about their own experiences) or remain ‘completely neutral’. This conundrum could arise particularly where delivery of inclusive SRE required that homophobic comments by pupils be challenged, a situation that teachers could find particularly difficult:  “... the homosexuality and abortion thing, I don’t have a neutral stance and I try very hard to do it differently but I can’t help it because it so annoys me and the trite comments that I would normally just take-up and expand, I take-up and expand *and react*, you know. You know, the big lad saying ‘just so long as he doesn’t follow me with his bar of soap’. The other day I said ‘what makes you think he would fancy you Derek?” (female teacher, aged 31-40y) [emphasis added] (Buston & Hart 2001, p.102)  *Some* teachers were able to handle such situations without needing to refer to their own values:  “I try not to put my values, impose my values on [pupils] because they have the right to put their own values on, make their own decisions... it is more a case of trying to get them to look at it and look at both sides of the argument without them necessarily knowing what I think.” (male teacher, aged 41-50y) (Buston & Hart 2001, p.103)  Nevertheless, survey findings showed that most teachers (81.4%) reported feeling ‘some degree of confidence’ about discussing homesexuality in the classroom (Buston & Hart 2001, p.104)  Teachers more likely to report feeling ‘confident’ or ‘very confident’ teaching about more abstract issues relating to sex (STDs, contraception, attitudes to AIDS) than particular issues relating to homosexuality or explicit details relating to sexual activity (demonstrating condom application using a model phallus, discussing sexual activities *other* than sexual intercourse, discussing sexual pleasure and/or orgasms) (Buston & Hart 2001, p.104)  Teachers largely found the subject of sex itself problematic:  “[Sex education is] a difficult issue for people to handle… I’m not criticising people for finding these things difficult, they *are* difficult.” (male Associate Head Teacher) (Buston et al. 2001, p.357)  Although the teachers interviewed all had a pastoral remit (e.g. PSE co-ordinator), this was not specifically with regard to SRE. ‘Difficulty’ and ‘comfort’ ‘featured heavily’ in teachers’ interviews, even though ‘the importance of feeling comfortable and relaxed when delivering sex education was stressed by teachers, and yet discomfort was common’ (Buston et al. 2001, p.358)  Teachers expressed concern about ‘remaining neutral’ and found it quite difficulty to do so:  “You have to try very, very hard not to show your own values or your own views. I’ve been very careful, I’ve tried to be very conscious of it… I’ve made a conscious effort because I think there are sometimes when I’ve, I may have, you know, when I saw myself giving over what I might feel at the time, I just had to. Yes, quite difficult I find, you know, not to sway the children one way or the other.” (female teacher) (Buston et al. 2001, p.358) |
| 2d | Modes of delivery that appeal to and engage pupils and provide cognitive and/or emotional rewards  ‘ . . . sex education teachers were assessed [by the pupils] more on the basis of their general relationship with pupils, in and out of PSE, than on their delivery of sex education lessons or their personal values. Having a good sense of humour; respecting pupils “treating us like adults”; and being trustworthy “genuine”; were regarded as particularly important. Conversely the sex and personal values of teachers rarely featured in pupils’ comments, and while it was easier for young teachers to meet pupils’ approval youth was not essential. Thus an old, gruff male teacher, who had a didactic style and expressed his own personal belief that sex should ideally he reserved for marriage, was much liked by both sexes in his class’  Girl: “It makes you understand it better as well, because it’s not all serious. It’s a laugh, and that’s the sort of things that stick in your head. It’s what he’s made funny”  ‘In contrast a “sex-positive” teacher with active learning methods was disparaged by a boys’ group because he had reportedly betrayed a boy’s confidence and informed on him to the head teacher about a matter unrelated to sex education’ (Wight et al. 1998, p.11)  *School sex education – ‘*Particular aspects that were criticised were: that nothing new was learnt and/or it came too late (*n=*12) (discussed below [timing]); there was not enough education overall or on particular topics (*n=*9); and that the course or parts of it were unrealistic (*n=*6). On this latter point, most of those making this criticism had experienced sexual intercourse by the age of 14. (Buston & Wight 2002, p.240)  *Timing* – ‘As indicated above, equal numbers felt that their sex education had come at the right time or that it came too late and/or they had learnt nothing new from it.’  “Because we’re getting it in third year some of the girls have done it and been there, do you know what I mean? It’s like basic stuff that we’re getting now.” (female pupil, 14y, >1 sexual partner) (Buston & Wight 2002, p.240)  *Skills-based lessons* – ‘The young women often valued lessons to develop their skills, regardless of its immediate relevance for them in terms of their own lives. Most of the young women from School 2, for example, talked about how valuable the lesson was in which they were given the opportunity to practise putting a condom on a carrot.’ e.g.:  “… because you got the condom and the carrot and then inside was a leaflet so you read that through first and then we done it and that’s how you really sort of picked it up so I’d say that lesson was good (female pupil, 16y, one sexual partner) (Buston & Wight 2002, p.241)  ‘Nearly three-quarters of pupils interviewed individually, though fewer boys than girls, talked about being uncomfortable in the sex education classroom… discomfort manifested itself, according to pupils, in reluctance to ask questions in class and/or to join in with whole class or small group discussion, as well as in disruptive behaviour, such as frequent laughter, ‘messing around’ and asking ‘silly’ questions.’ (Buston et al. 2002, p.322)  A number of pupils were ‘afraid of being laughed at and/or ‘slagged off’ if they participated in classes, [e.g.]’  “I sat at the back and kept to myself… I think it was the sex education. I’m quite an outgoing person but I just thought, like, people will laugh at me.” (female pupil, 16y)  ‘The possibility that what they said would be ‘spread round the school’ and they would be made fun of not only in that specific lesson, but, for example, for several weeks to come was an element of this fear. Most commonly, anxieties for both boys and girls centred around making a comment or asking a question that revealed they did not know something that all their classmates did.’ (Buston et al. 2002, p.323)  ‘Gender dynamics among pupils was mentioned as problematic by nearly all those interviewed. The majority of pupils who identified gender issues talked about boys’ and girls’ different responses to sex education, which was invariably presented as problematic, while a smaller number talked specifically about being uncomfortable and feeling constrained in mixed-sex SRE classes… Just over half of the comments about discomfort referred to the general embarrassment of being in a mixed-sex context. Those that were more specific nearly all referred to the way in which the reactions of the opposite sex inhibited participation.’ – e.g. ‘one boy said he would not ask very explicit questions of the teacher because he was afraid the girls would laugh at him’; ‘three girls said they would not ask questions or join in discussions for fear of ‘getting slagged’ and being the butt of ‘snide remarks’ and/or jokes.’ (Buston et al. 2002, p.323)  Authors suggest that gender differences meant there was a risk that in mixed-sex classes SRE didn’t engage with (and therefore enable) boys sufficiently – this risked missing out on boys’ ways of learning about sex and relationships, e.g. ‘…the boys said they knew the girls thought they were immature when they laughed during the sex education classes and talked about ‘basic’ things, but they felt they were being open and that this was much more useful than the contributions of the girls’:  “…we bring up anything… about sex or the body or anything… as soon as you like speak about these things to girls they get all bashful and they don’t want to talk to you… I think they [the teachers] should make the girls feel a bit more comfortable” (male pupils, age 14-15y)  A girl (14y) from the same class described the boys as often “speaking about pervert things”, her class-mate adding “[the boys are] more explicit than the girls, you know, they don’t talk about feelings, it’s more like the physical.” (Buston et al. 2002, p.324)  ‘For pupils to feel comfortable and to participate in the lesson, they had to feel confident that their teacher would ensure that clas-mates would not ridicule them’ – this was related to:  a) class control, e.g. “You could hear them [boys] laughing in that bit and laughing this bit. You’re like that ‘Mr Betts, are you going to tell them to shut up and listen?’” (female pupil, 14y)  b) ‘heightened feelings of vulnerability for individual pupils’, e.g. cf.:  i) “Mrs Blackshaw, right, she can’t really talk to us properly about it because the boys start making snide remarks and everything like that… so you get to the stage where, if you want to ask anything you won’t ask it because the boys will start making remarks” (female pupil, 15y)  ii) “She’s [the teacher] relaxed, but then again if somebody stepped out of line she would be like telling them ‘don’t do that because it’s going to hurt someone’s feelings’” (female pupil, 15y)  (Buston et al. 2002, p.325)  Authors identify small group work and agreement of ground rules as ways in which trust between pupils could be developed – these were ‘mentioned by some pupils as useful in making them feel comfortable with classmates, but it appears to be their use by a particular teacher that is more important than the strategy *per se*’, i.e. it was how group work or ground rules were implemented by teachers that explained whether or not pupils found them to be useful strategies. (Buston et al. 2002, p.323)  ‘Being able to be light-hearted in sex education… was seen as facilitating engagement and learning by pupils of both sexes and all ages, e.g.:  “[jokey sex education] makes you understand it better as well because it’s not all serious… it’s a laugh and that’s the sort of thing that sticks in your head.” (female pupil, 14y)  “[fun] breaks the sort of nervousness” (female pupil, 16y)  “[humour] makes you comfortable as well. I mean you’re laughing and joining in when you look at it, you know, and you’re sharing the same emotion and that.” (male pupil, 15y)  (Buston et al. 2002, p.328)  Authors identify trust between pupils as the necessary, but not sufficient, factor that corresponded with engagement – fun, class control, and the teacher acting as a ‘friend’ (rather than ‘teacher’) were significant ‘boosters of engagement’, but were not of themselves sufficient to achieve engagement. (Buston et al. 2002, p.330)  Lesson observations of programme delivery highlighted variations in how classes with similar characteristics (e.g. age, maturity mix, interest in sexual health matters) could react quite differently to delivery styles (e.g. use of humour, level of discipline) (Buston & Wight 2004) |
| 2e | Providing support materials that are appealing and appropriate to pupils’ age, interests and culture  NR |
| **3 – Embedding a programme into routine practice** | **The introduction and routine delivery (‘embedding’) of a programme takes time and motivation. It is likely to involve changes in the school environment and the development of new relationships between stakeholders that require pro-active management so that:** |
| 3a | - different stakeholders’ goals are reconciled - organisational decisions in other areas of school life are made taking into account how they impact on programme delivery - school staffs’ existing relationships with children are built upon - stakeholders’ enthusiasm, knowledge and experience are harnessed - knowledge of ‘core’ and ‘peripheral’ elements and minimum resources, skills and informational content is retained - responsibility for programme delivery becomes rooted in the school   NR |
| 3b | The longer-term sustainability of programmes and the extent to which health promoting messages and activities permeate other aspects of school life, is dependent on continuing feedback, encouragement, and expectations about implementation. Over time, this may originate less from outside the school and more from inside.  NR |
| **4 - Programme adaptation** | **The preparation for, introduction, initial delivery, and ongoing sustainability of a health promotion programme in a school is more likely to be successful when there is:** |
| 4a | Specificity about ‘core’ (essential) and ‘peripheral’ (optional/adaptable) programme elements, including the minimum levels of resources and/or skills necessary to support these elements (to inform decision-making about how to deliver a programme in the context of a particular school)  ‘Core’ and ‘peripheral elements not identified – teachers made adaptations which in the researchers’ assessment were sometimes warranted (e.g. removal of cartoon pictures of naked people from teaching materials to avoid pupils making unflattering comparisons with teaching staff), but sometimes substantively distorted the programme (e.g. missing out sessions). There was great variability in the extent to which fidelity was attained, with no clear pattern except in relation to team cohesion. This variability was occurred in spite of the teachers who delivered the sex education being ‘fully involved in discussions about whether to participate in the study’ (Buston et al. 2002; HER, p.64).  Timetabling pressures drove revisions to the SHARE programme – almost all schools ‘raised this as a problem’. The final programme sessions (sexual health services; review of programme) were ‘most often abbreviated, often drastically’ (Buston et al. 2002; HER, p.66). |
| 4b | Scope for ‘mutual adaptation’ between the programme and the people delivering it, including the evolution and updating of programme content and mode of delivery over time  4b – [Commitment to research] ‘Although the intervention was generally regarded as very worthwhile, some teachers were more committed to the research project than others. In some schools teachers took their agreement to deliver an unmodified programme very seriously and a few sex education coordinators who appeared to be particularly committed to the study consulted D. W. on whether or not modifications would be acceptable. Other teachers felt their professional autonomy should allow them to adapt the programme without such consultation’ (Buston et al. 2002 p.68)  Authors’ claim a relationship across all the schools between team cohesion and fidelity of delivery (data reported in Buston et al. 2002 (HER), although it is not clear how this relationship has been established) – ‘Although not always the case, in schools where there was a sense that members pull together there tended to be greater within-school standardisation in provision.’ (Buston et al. 2001, p. 363). This is illustrated with a example:  ‘At Canalside High, the Guidance Team got along well. The member of senior management with responsibility for guidance was viewed as very much part of this team, teaching PSE to classes herself when timetabling difficulties meant that Guidance teachers could not teach their own case-load. The sex education programme here was tightly defined by this AHT, with the support of the team who agreed to deliver a particular package. The team then engaged in feedback about its delivery, discussing any problems that they encountered. In short, there was a culture of openness. Some of the team socialised with each other out of school but, more importantly, there was a supportive ethos within the team as a whole with members of staff regularly using the guidance room.’ (Buston et al. 2001, p.363) |

| **HP Programme** |  |
| --- | --- |
| Name of programme | Smoking and Me |
| Area of HP | Substance use (Tobacco) |
| Authors (year/ study type) | Newman et al. (1991)/ process evaluation |
| Quality appraisal | Significant weaknesses in reporting (scant or no information on study design, data collection and analysis) suggest that the overall study design and conduct was weak and that findings should be viewed with caution. |
| Programme description | Adapted from a US programme – teachers’ guide provides 5 lesson outlines (emphasising ‘recognising and practicing skills for managing social situations in which smoking occurs’) and ‘background information on young people and smoking, guidance for teachers on choosing group leaders… and managing groups’ (p.107). No visual aids or pupil project materials are provided. A large part of the ‘work’ of the programme is done by pupils themselves in small groups – ‘group leaders [peer leaders – same age] lead discussion, role-play and decision-making activities’, with support from teachers.  Prior to programme delivery, all staff from the schools who would be involved with its teaching were asked to attend a 1-day training course – which aimed to provide information about the issue (smoking and young people) and ‘familiarise teachers with the project guide and the group leader approach to teaching’ (p.108). Contributors to the training day were: researchers, health education officers, and teachers who had previously taught the programme. |
| Year(s) delivered | 1989-1990 |
| Description of UK location(s) where delivered | Mixed-sex comprehensives in England and Wales LEAs |
| **Research methods** |  |
| Theoretical approach | None |
| Sample | Schools randomly selected; unclear how teachers who taught the programme were selected |
| Participants | Teachers who taught the programme |
| Data collection | ‘Teachers’ record’ asking for feedback on programme delivery (n=65) – no further details provided |
| Analysis | NR |
| Time of follow-up | NR |
| **Evidence about programme theory#** |  |
| **1 - Preparing for implementation** | **Preparation for the introduction of a health promotion programme to a school is more likely to be successful when systematically planned in conjunction with other school responsibilities.** This involves: |
| 1a | Consultation with stakeholders, which involves:   - providing information on the programme (e.g. who and what is involved, evidence of effectiveness) - eliciting views on ‘readiness for change’ as a starting point for engagement - encouraging the sharing of previous experiences and knowledge of delivering health promotion programmes - facilitating discussion about the concordance (‘fit’) of the programme with:   - current practice  - pupils’ interests  - current school policies, resources and organisation  Mis-match between proposed programme lesson duration (45-50mins) and conventional single (35-40mins) or double periods (70-80mins).  The majority of pupils from non-smoking families were reported by teachers to have found the programme ‘meaningless’. |
| 1b | Identification of a potential health benefit for pupils at a local level  NR |
| 1c | Consultation with stakeholders (head-teacher, school staff, pupils, parents, governors)  NR |
| 1d | Consideration of the concordance of the programme with current practice and interests  NR |
| 1e | Identifying clear aims and priorities, including intended outcomes  NR |
| 1f | Taking into account the current situation and competencies in a school and the implications of these for programme refinement  NR |
| **2 - Introducing a programme** | **The introduction of a health promotion programme to a school is more likely to be successful when it is incorporated into school activities through:** |
| 2a | Being integrated into school policy (e.g. a School Improvement Plan) and supported by governors and senior staff to whom monitoring and progress reports are made  NR |
| 2b | School staff using their leadership skills to co-ordinate activities or resources for programme delivery  NR |
| 2c | Providing adequate opportunity and/or training for the personal and professional development of those who will deliver the programme and engaging with their interests  NR |
| 2d | Modes of delivery that appeal to and engage pupils and provide cognitive and/or emotional rewards  Teachers viewed pupils as engaging positively to the peer-led groups, e.g. because of the change from ‘teacher-led’ to peer-led groupwork. |
| 2e | Providing support materials that are appealing and appropriate to pupils’ age, interests and culture  ‘A number of teachers commented that the peer-leader notes were too complicated for some children to follow’ (p.108) |
| **3 – Embedding a programme into routine practice** | **The introduction and routine delivery (‘embedding’) of a programme takes time and motivation. It is likely to involve changes in the school environment and the development of new relationships between stakeholders that require pro-active management so that:** |
| 3a | - different stakeholders’ goals are reconciled - organisational decisions in other areas of school life are made taking into account how they impact on programme delivery - school staffs’ existing relationships with children are built upon - stakeholders’ enthusiasm, knowledge and experience are harnessed - knowledge of ‘core’ and ‘peripheral’ elements and minimum resources, skills and informational content is retained - responsibility for programme delivery becomes rooted in the school   NR |
| 3b | The longer-term sustainability of programmes and the extent to which health promoting messages and activities permeate other aspects of school life, is dependent on continuing feedback, encouragement, and expectations about implementation. Over time, this may originate less from outside the school and more from inside.  NR |
| **4 - Programme adaptation** | **The preparation for, introduction, initial delivery, and ongoing sustainability of a health promotion programme in a school is more likely to be successful when there is:** |
| 4a | Specificity about ‘core’ (essential) and ‘peripheral’ (optional/adaptable) programme elements, including the minimum levels of resources and/or skills necessary to support these elements (to inform decision-making about how to deliver a programme in the context of a particular school)  The use of peer-leaders was a core part of the programme, but only two-thirds of the teachers used peer-leaders in all programme lessons. Reasons identified by teachers for not using peer-leaders:  a) lack of momentum by groups and leaders  b) uncertainties about when to use group leaders  c) problems with low academic ability groups  d) peer-leaders’ need for more advice  e) some peer-leaders wanting to opt-out  ‘Most of the teachers reported following the lesson outlines’, with: alterations to 20% of lessons, omissions from 19%, and additions to 11%. These changes were made for reasons such as differing group work rates and some pupils/groups requiring additional direction from teachers. |
| 4b | Scope for ‘mutual adaptation’ between the programme and the people delivering it, including the evolution and updating of programme content and mode of delivery over time  Around 25% of the teachers did not follow the programme advice about selection of groups and peer-leaders, e.g. because of a perceived need mix genders and/or reading/writing abilities. It is unclear what the impact of this was. |

| **HP Programme** |  |
| --- | --- |
| Name of programme | SPICED |
| Area of HP | Substance use (legal & illegal drugs) |
| Authors (year/ study type) | Crosswaite et al. (2004/ process evaluation) |
| Quality appraisal | Data collection and analysis processes not reported. Sampling strategy of schools and respondents not reported. Presentation of qualitative data is weakly-contextualised and largely descriptive. Connection between data and analysis frequently unclear. |
| Programme description | ‘The SPICED programme consists of seven classroom-based lessons facilitated by the classroom teacher, a police officer and a school nurse. The three professionals worked in partnership for the duration of the programme using a SPICED pupils’ booklet and a range of supporting resources (e.g. substance use picture cards). Role play, participative learning and group discussion featured prominently in the programme. All professionals delivering SPICED participated in a two day training course and seven classroom-based sessions including an initial session with parents/carers. The other six sessions aimed to provide children with information about legal and illegal drugs, develop their understanding of risk, support them in discussing and exploring substance use issues, as well as promoting their personal confidence, self-esteem and decision making skills.’ (p.62) |
| Year(s) delivered | 1998-2001 |
| Description of UK location(s) where delivered | 91 primary schools in Kirklees – no further details provided |
| **Research methods** |  |
| Theoretical approach | NR |
| Sample | “A systematic sample” (p.62) – no further details provided |
| Participants | Pupils aged 9-11yrs |
| Data collection | Interviews (teachers, school nurses and police officers) (n=29) - explored the implementation of SPICED in the classroom setting, the training provided and issues around the involvement of each professional group  ‘Short’ focus groups (n=79 pupils; 6-8 pupils per group in 10 schools) - experiences of the SPICED programme |
| Analysis | NR |
| Time of follow-up | NR |
| **Evidence about programme theory#** |  |
| **1 - Preparing for implementation** | **Preparation for the introduction of a health promotion programme to a school is more likely to be successful when systematically planned in conjunction with other school responsibilities.** This involves: |
| 1a | Consultation with stakeholders, which involves:   - providing information on the programme (e.g. who and what is involved, evidence of effectiveness) - eliciting views on ‘readiness for change’ as a starting point for engagement - encouraging the sharing of previous experiences and knowledge of delivering health promotion programmes - facilitating discussion about the concordance (‘fit’) of the programme with:   - current practice  - pupils’ interests  - current school policies, resources and organisation  NR |
| 1b | Identification of a potential health benefit for pupils at a local level  NR |
| 1c | Consultation with stakeholders (head-teacher, school staff, pupils, parents, governors)  NR |
| 1d | Consideration of the concordance of the programme with current practice and interests  NR |
| 1e | Identifying clear aims and priorities, including intended outcomes  NR |
| 1f | Taking into account the current situation and competencies in a school and the implications of these for programme refinement  ‘The pressures of the primary school curriculum meant that some teachers found that there was insufficient time:  “We as a school tried to bring out the important things and we always felt [we] were cutting back on some things . . . we could have done with more time” (teacher) (p.64) |
| **2 - Introducing a programme** | **The introduction of a health promotion programme to a school is more likely to be successful when it is incorporated into school activities through:** |
| 2a | Being integrated into school policy (e.g. a School Improvement Plan) and supported by governors and senior staff to whom monitoring and progress reports are made  NR |
| 2b | School staff using their leadership skills to co-ordinate activities or resources for programme delivery  NR |
| 2c | Providing adequate opportunity and/or training for the personal and professional development of those who will deliver the programme and engaging with their interests  NR |
| 2d | Modes of delivery that appeal to and engage pupils and provide cognitive and/or emotional rewards  ‘Sources of support and advice for young people were explored’  “I liked the school nurse coming into school. It was interesting and I learnt a lot” (p.65)  ‘The SPICED lessons had been enjoyable and were valued for being different from ordinary school lessons’  “If a lesson is boring you don’t remember it, but if it’s fun you remember” (p.65-66)  ‘The children felt they would like more visual media to be used and would have preferred to have had more of an emphasis on role play and time for discussion. Similarly, some felt that there had been insufficient time available for them to speak to external professional and that on occasion questions had gone unanswered’  “We should do more about drugs in school”(p.66) |
| 2e | Providing support materials that are appealing and appropriate to pupils’ age, interests and culture  NR |
| **3 – Embedding a programme into routine practice** | **The introduction and routine delivery (‘embedding’) of a programme takes time and motivation. It is likely to involve changes in the school environment and the development of new relationships between stakeholders that require pro-active management so that:** |
| 3a | - different stakeholders’ goals are reconciled - organisational decisions in other areas of school life are made taking into account how they impact on programme delivery - school staffs’ existing relationships with children are built upon - stakeholders’ enthusiasm, knowledge and experience are harnessed - knowledge of ‘core’ and ‘peripheral’ elements and minimum resources, skills and informational content is retained - responsibility for programme delivery becomes rooted in the school   NR |
| 3b | The longer-term sustainability of programmes and the extent to which health promoting messages and activities permeate other aspects of school life, is dependent on continuing feedback, encouragement, and expectations about implementation. Over time, this may originate less from outside the school and more from inside.  ‘Awareness of the Health Schools initiative was high but few had had the opportunity to link SPICED in with other activities or to give consideration to continuation of SPICED work after the programme had been completed’ (p.64) |
| **4 - Programme adaptation** | **The preparation for, introduction, initial delivery, and ongoing sustainability of a health promotion programme in a school is more likely to be successful when there is:** |
| 4a | Specificity about ‘core’ (essential) and ‘peripheral’ (optional/adaptable) programme elements, including the minimum levels of resources and/or skills necessary to support these elements (to inform decision-making about how to deliver a programme in the context of a particular school)  NR |
| 4b | Scope for ‘mutual adaptation’ between the programme and the people delivering it, including the evolution and updating of programme content and mode of delivery over time  NR |

| **HP Programme** |  |
| --- | --- |
| Name of programme | UK Resilience Programme |
| Area of HP | Well-being |
| Authors (year/ study type) | ^1^Challen et al. (2009/ CBA)  ^2^Challen et al. (2011/ CBA) |
| Quality appraisal | ^1,2^Whilst sampling strategy and data collection procedures are well-described, data analysis of process evaluation data is reported in very little detail even though there was extensive qualitative data from interviews. Findings are closely linked to examples from the data, but lack the richness that would be expected in a process evaluation of this scale. |
| Programme description | The UK Resilience Programme is the first larger-scale use of the Penn Resilience Program (PRP) curriculum. Implemented as 18 hours of workshops for Year 7 children in 22 UK secondary schools.  ‘The Penn Resiliency Program is intended to build resilience and promote optimistic thinking, adaptive coping skills and social problem-solving in children, with the aim of improving psychological well-being, but potentially also behaviour, attendance and academic outcomes... It is a manualised intervention comprising 18 hours of workshops. The curriculum teaches cognitive-behavioural and social problem-solving skills. Participants are encouraged to identify and challenge negative beliefs, to employ evidence to make more accurate appraisals of situations and others’ behaviour, and to use effective coping mechanisms when faced with adversity. Participants also learn techniques for positive social behaviour, assertiveness, negotiation, decision-making, and relaxation.’ (p.4) The programme can be delivered by a range of professionals (teachers, learning mentors, teaching assistants, psychologists, health professionals). Preparation for delivery consisted of 8-10 days training *in the USA* (week 1: adult-level Cognitive Behavioural Therapy (CBT) skills; week 2: familiarisation with programme curriculum and ‘practising how to communicate it to pupils’) (Challen et al. 2009, p.4) |
| Year(s) delivered | 2007 – 2008 (1^st^ cohort) |
| Description of UK location(s) where delivered | Schools (n=22) in North Tyneside, Manchester & Hertfordshire |
| **Research methods** |  |
| Theoretical approach | NR |
| Sample | Purposive  Schools ‘selected to reflect the variation proportion of Yr.7 pupils receiving the programme, levels of pupil attainment, rates of eligibility for free school meals’ (Challen et al. 2011, p.64)  Teachers and managers invited to participate from each case study school  Pupils selected for interview by teachers, who were asked to suggest participants to reflect a ‘balance in boys and girls and including somewhat higher and lower attaining pupils’ (Challen et al. 2009, p.63) |
| Participants | Pupils who had participated in the programme  Teachers and managers who were involved with programme delivery |
| Data collection | For process evaluation, 10 case study schools (at least 3 from each local authority area)  Semi-structured interviews (managers n=9; teachers/facilitators n=20; pupils n=45 (24 boys, 21 girls) |
| Analysis | ‘Interviews were analysed thematically’ (Challen et al. 2009, p.65) (no further details reported) |
| Time of follow-up | Post workshop |
| **Evidence about programme theory#** |  |
| **1 - Preparing for implementation** | **Preparation for the introduction of a health promotion programme to a school is more likely to be successful when systematically planned in conjunction with other school responsibilities.** This involves: |
| 1a | Consultation with stakeholders, which involves:   - providing information on the programme (e.g. who and what is involved, evidence of effectiveness) - eliciting views on ‘readiness for change’ as a starting point for engagement   ‘Facilitators were also self-selected, although some may also have had some selection imposed by their school… the self selection might result in facilitators being particularly highly motivated (perhaps increasing the success of the workshops)… Facilitators were asked to give up the first two weeks of their summer holidays in order to train in Philadelphia, and had to prepare a large amount of new material in order to teach the workshops. One would therefore expect that these individuals and schools were highly motivated and enthusiastic, and had a strong belief in the importance of the subject being taught’ (Challen et al. 2009, p.6)  ‘At four of the schools senior managers had discussed how best to deliver the UKRP and which members of staff would be most suitable for the role. This most often included consideration of the suitability of members of staff for the facilitator role and secondarily consideration of where the UKRP would fit into the school’s present and future curriculum. As one manager put it:’  “I approached people I thought were suitable and they were pleased to be asked”  At other schools interviewees described an open call to staff at a briefing session and at three schools a combination of these approaches was used. Demand for places on the course varied markedly between schools. At one school there were ten applicants for each place available. Places were then allocated by a random ballot…At another school the senior manager chose from among the volunteers, taking into account their attributes and also the school timetable and curriculum needs of the school’ (Challen et al. 2009, p.89)  ‘The importance of recruiting the *right* people was echoed at all levels and by numerous interviewees. This included not only the recruitment of facilitators but also anyone taking on a role in training future facilitators’ (Challen et al. 2009, p.90)   - encouraging the sharing of previous experiences and knowledge of delivering health promotion programmes   ‘At another of the schools the headteacher had made it clear from the outset that, owing to a bad experience with a previous innovative programme, he was unwilling to commit to deliver the UKRP until after the training was completed because only then could his staff make a judgement as to the suitability of the programme. Consequently it was only when the senior management team were persuaded of its potential value that planning could begin in earnest’ (Challen et al. 2009, p.86)   - facilitating discussion about the concordance (‘fit’) of the programme with:   - current practice  - pupils’ interests  - current school policies, resources and organisation  ‘In addition, schools had to abide by certain restrictions when timetabling workshops. These included ensuring that only trained staff were timetabled to teach the subject, and that classes did not contain more than 15 pupils, resulting in a doubling of staffing and rooms for these classes’ (Challen et al. 2009, p.6)  ‘At three of the schools however it was only when the trained facilitators had returned to school in September 2007 that they began considering how to fit the UKRP into the timetable. In one of these schools the facilitators, two of whom were from the same subject department, persuaded their head of department that the programme addressed some of the Key Stage 3 curriculum aims for that subject. As a result they were able to identify two Year 7 form groups to whom the four facilitators could deliver the programme because the lessons coincided with facilitators’ free periods’ (Challen et al. 2009, p.86)  ‘At another school, a senior manager expressed the view that the UKRP had not had backing from the senior management team as a whole. When asked why the school had got involved with the UKRP this was the response:’  “It’s a tick box thing being involved in new initiatives and so on. It is seen to be good - and  sometimes the implications of what doing it well would actually mean haven’t been very  well thought through.  *Right and do you think that’s true in this case?*  Yes.  *And in what way has that shown itself?*  Nobody seems to be taking very strong a lead…I suppose really it’s just been passed  around people” (Challen et al. 2009, p.86)  ‘It was acknowledged by several interviewees that although there were undoubtedly timetabling problems, these could be resolved if the programme was given a sufficiently high priority within the school when the timetable was written’ (Challen et al. 2009, p.86)  ‘UKRP groups were required to be no larger than 15 pupils. Most often this was achieved by splitting a class in half. Clearly splitting classes in half also meant extra rooms needed to be available. This presented a challenge in schools with very little spare capacity and could result in UKRP sessions being run in areas that some facilitators thought were unsuitable (e.g. science  Laboratories)… At a school with a standard Year 7 class size of 23, groups of 15 were achieved by timetabling three classes for UKRP sessions in the same timetable slot so that they could be split into five UKRP groups. Given that a maximum of four members of staff had been trained per school, this necessitated bringing in external facilitators to deliver the programme (Challen et al. 2009, p.86-87)  ‘Of course, all such timetabling difficulties could be overcome but not all of the senior management teams chose to give the programme a sufficiently high priority. One senior manager responsible for timetabling explained that the programme would be sustainable if it involved a teacher and a teaching assistant delivering the programme to a proportion of the Year 7 cohort. However, it was felt that having two teachers facilitating the course for a single class would be too costly. It was explained that in the school, largely driven by national targets, all additional resources were targeted on Year 11 pupils and on stretching the most able pupils. (Having seen a substantial improvement in GCSE attainment in 2008 this interviewee may feel vindicated in this view.)’ (Challen et al. 2009, p.87)  ‘Despite these apparent complications, in one school the UKRP provided a timetabling solution rather than problem. In this school maths, modern languages and science were taught in the same sets across the three subjects. The rather untidy timetabling solution of replacing one of the maths lessons of the top set, one of the science lessons of the bottom set etc. then enabled the school to overcome staffing problems in that year (The following year UKRP lessons replaced a science lesson for all Year 7 pupils, facilitated by the departure of the head of science and the science department acquiring an extra lesson with all Year 9 pupils.)’ (Challen et al. 2009, p.87)  ‘In all schools, logistical challenges were lessened by the availability of facilitators who did not have a full teaching load’ (Challen et al. 2009, p.88)  ‘Respondents however uniformly emphasised the importance of the personal characteristics of the facilitator rather than their job title:’  “I think you’ve got to get the right staff judged on an individual basis…you’ve got to have people that will respond appropriately and sensitively to some of the issues. And not every teacher, or every individual, has the capacity to do that”  ‘Nevertheless, as a rule of thumb, a respondent explained:’  “Non-teachers have to know they’re comfortable leading a group of 15 and teachers have to know they’re comfortable with the “touchy feely””( Challen et al. 2009, p.88) |
| 1b | Identification of a potential health benefit for pupils at a local level  NR |
| 1c | Consultation with stakeholders (head-teacher, school staff, pupils, parents, governors)  NR |
| 1d | Consideration of the concordance of the programme with current practice and interests  NR |
| 1e | Identifying clear aims and priorities, including intended outcomes  NR |
| 1f | Taking into account the current situation and competencies in a school and the implications of these for programme refinement  NR |
| **2 - Introducing a programme** | **The introduction of a health promotion programme to a school is more likely to be successful when it is incorporated into school activities through:** |
| 2a | Being integrated into school policy (e.g. a School Improvement Plan) and supported by governors and senior staff to whom monitoring and progress reports are made  ‘Advance planning for the UKRP appeared to have varied considerably between schools. When asked how important management backing was to the successful implementation of the programme, one local authority co-ordinator remarked:’  “Absolutely crucial…Where it is working best, like anything else, it is totally coming from the top and there is a clear person responsible with time to monitor” (Challen et al. 2009, p.85) |
| 2b | School staff using their leadership skills to co-ordinate activities or resources for programme delivery  NR |
| 2c | Providing adequate opportunity and/or training for the personal and professional development of those who will deliver the programme and engaging with their interests  ‘Over 90% of facilitators agreed to some extent that they enjoyed facilitating the workshops (see Table 14). Almost as many felt that the experience had improved their professional skills to some extent (89%)… 30 facilitators responded to this question…The single most popular comment was that facilitators used the UKRP skills themselves, and some mentioned that they had become more optimistic or confident, with seven saying they had become more aware of their own emotions. In addition, five said they enjoyed workshops, five that they had become more aware of and sensitive to pupils and their problems, and three that they had had a chance to build closer relationships with pupils than their ordinary teaching allowed’ (Challen et al. 2009, p.27)  ‘Several facilitators found the peer teaching (teaching lessons to other trainee facilitators), which formed part of the training programme, daunting and this appeared to be particularly so for those who were not (and had never been) class teachers. Some of the non-teachers (for example, learning mentors, teaching assistants, cover supervisors) found the sessions in which they  practiced delivering the UKRP curriculum very valuable. In contrast for two respondents with greater confidence in their teaching skills, the theory element of the training was more important’ (Challen et al. 2009, p.76)  ‘The initial flush of enthusiasm reduced as they faced the reality of delivering a course based on cognitive-behavioural therapy to Year 7 pupils… Nevertheless, in general, facilitators were very positive about the programme. Indeed, they were exceptionally enthusiastic about the potential value of teaching resilience skills to pupils. Almost all the facilitators reported using the skills, which in itself indicates their belief in the value of the techniques’ (Challen et al. 2009, p.77)  ‘There was a fervour and excitement about the programme that was discernible among some of the first cohort of interviewees [who had training in the USA] but this was less obvious among interviewees from subsequent cohorts [who had training in the UK].’ (Challen et al. 2011, p.75) |
| 2d | Modes of delivery that appeal to and engage pupils and provide cognitive and/or emotional rewards  ‘Pupils most frequently reported liking UKRP sessions because they were concerned with real life, allowed pupils to talk about themselves, involved fun activities such as role play and did not involve much writing. Resilience lessons gave pupils the opportunity to discuss problems with the group and we were particularly keen to find out whether this had been a positive or negative experience…While some had discussed real life problems, several stated explicitly that they had chosen not to discuss some problems within the group because they did not want to share some aspects of their lives with other pupils or were not sure they would receive a supportive response from classmates. No pupil suggested that any member of their group had broken the confidentiality rule’ (Challen et al. 2009, p.75) |
| 2e | Providing support materials that are appealing and appropriate to pupils’ age, interests and culture  ‘Some of the facilitators also expressed the view that some of the examples used in the teaching materials were not appropriate for a Year 7 class. For example one facilitator suggested that pupils were too young to engage with role plays revolving around ‘dating’, while a few facilitators also suggested their groups had felt they were too old to have a story read to them. In addition,  the materials were reported to be recognisably ‘American’ in language and sometimes content also (without prompting as to the source of the programme, one group habitually enacted role plays with American accents) and this could sometimes create an additional and unnecessary distance between pupils and learning materials’ (Challen et al. 2009, p.79)  ‘Sessions in which pupils had played games (notably the ‘File game’ in which pupils examine evidence to examine the accuracy of an imaginary character’s thoughts and ‘jeopardy’ in which pupils review the course through a quiz) were reported to have been particularly popular. Similarly several facilitators reported that pupils had fun learning about ‘putting it into perspective’ (when worrying about something, considering the best, worst and most likely outcomes) and practicing relaxation techniques’ (Challen et al. 2009, p.79)  ‘Several facilitators expressed the view that the terminology of the programme was also very difficult for pupils, e.g. they might find it difficult to remember the meaning of optimism, pessimism, assertiveness or procrastination.’ (Challen et al. 2009, p.82) |
| **3 – Embedding a programme into routine practice** | **The introduction and routine delivery (‘embedding’) of a programme takes time and motivation. It is likely to involve changes in the school environment and the development of new relationships between stakeholders that require pro-active management so that:** |
| 3a | - different stakeholders’ goals are reconciled - organisational decisions in other areas of school life are made taking into account how they impact on programme delivery   In the one school in which the programme was discontinued, ‘the key facilitator had been taken off the UKRP team in order to devote more time to teaching English (which the school was under substantial pressure to improve levels of attainment in with the possible threat of closure if it should fail)’ (Challen et al. 2011, p.78)  In the third year of programme delivery, the programme ‘tended to be delivered by non-teachers’ – interviewees suggested that this was because non-teaching staff:  - offered greater flexibility (to overcome problems of timetabling two teachers to teach the same class in UKRP)  - liked the opportunity for additional earnings afforded to them by taking part in programme training during the school holiday (salaried teachers were not eligible for additional pay)  - valued the opportunity UKRP offered as a ‘stepping stone’ for going on to teacher training (Challen et al. 2011)   - school staffs’ existing relationships with children are built upon - stakeholders’ enthusiasm, knowledge and experience are harnessed - knowledge of ‘core’ and ‘peripheral’ elements and minimum resources, skills and informational content is retained - responsibility for programme delivery becomes rooted in the school   ‘Obviously the potential timetabling benefits of training non-teachers have to be traded off against any lack of experience teaching a group of 15 pupils over a sustained period. In addition, two interviewees spoke of the importance of continually marketing the UKRP within the school with one remarking that the continuation of the programme in that school may depend on the involvement of a senior and respected teacher as a facilitator’ (Challen et al. 2009, p.88) |
| 3b | The longer-term sustainability of programmes and the extent to which health promoting messages and activities permeate other aspects of school life, is dependent on continuing feedback, encouragement, and expectations about implementation. Over time, this may originate less from outside the school and more from inside.  NR |
| **4 - Programme adaptation** | **The preparation for, introduction, initial delivery, and ongoing sustainability of a health promotion programme in a school is more likely to be successful when there is:** |
| 4a | Specificity about ‘core’ (essential) and ‘peripheral’ (optional/adaptable) programme elements, including the minimum levels of resources and/or skills necessary to support these elements (to inform decision-making about how to deliver a programme in the context of a particular school)  NR |
| 4b | Scope for ‘mutual adaptation’ between the programme and the people delivering it, including the evolution and updating of programme content and mode of delivery over time  Variation in ‘adaptation’, ‘sometimes’:  - ‘adjustments to the teaching resources, including replacing some of the teaching materials with resources from alternative sources’  - adjustments to fit with the (less-prescriptive) lesson plans expected by Ofsted  - acknowledgement by teachers of a less ‘active’ role on the basis of areas of expertise, e.g.  “It’s not something we want to mess around with… because my concern is that none of us are trained psychologists or anything like that… we’re not qualified or in a position to start altering the content of something that is clearly long-established and obviously works.” (facilitator) (Challen et al. 2011, p.75) |

| **HP Programme** |  |
| --- | --- |
| Name of programme | Y-Active Programme |
| Area of HP | Physical activity / wellbeing |
| Authors (year/ study type) | Stathi & Sebire (2011/ case study) |
| Quality appraisal | While this study was well reported in terms of context, sampling, data collection, analysis, reflexivity and generalisability, it does reflect a single school: “The case study school used in the present research represents the most developed partnership, the outcome of approximately 5 years of collaboration between the YMCA and the school” (S240). On this basis its generalisability relating to the other Y-Active programmes is limited. |
| Programme description | ‘Y-Active is a multicomponent children’s program delivered by the Central YMCA in London, UK. A component of Y-Active is the outreach PA and well-being program which is delivered in 7 schools in the London area. The case-study school used in the present research represents the most developed partnership, the outcome of approximately 5 years of collaboration between the YMCA and the school.  *Intervention Content.* The Y-Active program consists of (a) breakfast club, (b) physical education, (c) Fit Kids lunchtime fitness classes, (d) after-school play club, and (e) after-school sports. Physical education and Fit Kids (both once per week per year group) are integrated in the school’s provision for all pupils. Breakfast club, after-school play club and after-school sports (once per week per year group) are opt-in activities for which parents pay a small fee.  *Location/Environment.* Breakfast club, Fit Kids classes, and after-school sports are delivered at school in a variety of places, including a sports hall, a dining hall, and the playground. Due to restricted space within the school, physical education is delivered at the nearby YMCA sports center. Pupils walk approximately 10 minutes under supervision to and from this facility.  *Personnel.* All intervention components are organized, planned, and delivered by a team of trained YMCA sports coaches and play workers. A school administrator liaises between the school and the YMCA on logistic issues such as timetabling and collecting fees. The Y-Active manager at the YMCA overseas the broader Y-Active program.’ (p.S240) |
| Year(s) delivered | NR |
| Description of UK location(s) where delivered | - A mixed gender, primary school in central London, UK (situated 0.70 miles from the Central YMCA service provider).  - 144 pupils from 4 -11 years old, diverse ethnic backgrounds.  - 1 class per year group, approximately 20 pupils per class.  - School employs 21 staff (1 head teacher, 10 teaching staff, 8 learning mentors and 2 administrative staff).  - Space for PA is limited to a small school dining hall, a purpose built small sports hall and the playground. The playground is a small outdoor space at street level and is surrounded on 3 sides by high-rise buildings. |
| **Research methods** |  |
| Theoretical approach | NR |
| Sample | Purposive |
| Participants | Pupils ages 9-11 years/ Y-Active sports leaders/ head teacher & class teachers/ Y-Active administrator |
| Data collection | Focus group (pupils n=9)  Interviews (Pupils n=8; YMCA leaders n=4; Teachers n=2; Administrator n=1) |
| Analysis | Thematic analysis (Atlas.ti - version 5.6.3) researchers conducted initial coding, refining of codes, created higher order themes |
| Time of follow-up | NR |
| **Evidence about programme theory#** |  |
| **1 - Preparing for implementation** | **Preparation for the introduction of a health promotion programme to a school is more likely to be successful when systematically planned in conjunction with other school responsibilities -** this involves**:** |
| 1a | Consultation with stakeholders, which involves:   - providing information on the programme (e.g. who and what is involved, evidence of effectiveness) - eliciting views on ‘readiness for change’ as a starting point for engagement - encouraging the sharing of previous experiences and knowledge of delivering health promotion programmes - facilitating discussion about the concordance (‘fit’) of the programme with:   - current practice  - pupils’ interests  - current school policies, resources and organisation  NR |
| 1b | Identification of a potential health benefit for pupils at a local level  NR |
| 1c | Consultation with stakeholders (head-teacher, school staff, pupils, parents, governors)  NR |
| 1d | Consideration of the concordance of the programme with current practice and interests  NR |
| 1e | Identifying clear aims and priorities, including intended outcomes  NR |
| 1f | Taking into account the current situation and competencies in a school and the implications of these for programme refinement  NR |
| **2 - Establishing a programme** | **The introduction of a health promotion programme to a school is more likely to be successful when it is incorporated into school activities through:** |
| 2a | Being integrated into school policy (e.g. a School Improvement Plan) and supported by governors and senior staff to whom monitoring and progress reports are made  NR |
| 2b | School staff using their leadership skills to co-ordinate activities or resources for programme delivery  The Service Provider-School Partnership. ‘The relationship that the YMCA leaders developed with the pupils and teachers was strong and built on open channels of communication between school management, staff and YMCA staff. Central to the positive relationship was the supportive head teacher who endorsed and encouraged the program and fostered a positive attitude toward the Y-Active program and leaders:’  “We work very closely with the Head Teacher. Her door is always open. . . . If a problem does arise we’re straight in there; it get sorted out, ironed out straight away. There’s a lot of communication in that school and the teachers are very on board with what we do and they support us in any way that they can which is always good.” (*Female Y-Active leader*)  “I think that if you don’t have the backing of your head teacher, or the backing of the staff I don’t think your extended school program would work at all. I think its one of these things that would either be very limited or completely fizzle out.” (*School administrator*) (p.S244)  ‘The logistics of having dedicated personnel to communicate between the school and the YMCA were also highlighted:’  “It is just ideal having somebody within a school who is specifically designated to do something like that, or if it is within their role because a lot of schools you’re going to go to, you’ll find that it is somebody they’ll tag on to their duties and then they don’t either care enough or they haven’t got the time to deal with it and I think that’s when messages are lost or chaos ensues.” (*School administrator*) (p.S245)  ‘Trust was also identified as a key element of the successful partnership:’  “I have to recommend [YMCA staff] they’re fantastic, I know that I could safely leave everything in their hands and they would deal with it.” (*School administrator*) (p.S245) |
| 2c | Providing adequate opportunity and/or training for the personal and professional development of those who will deliver the programme and engaging with their interests  NR |
| 2d | Modes of delivery that appeal to and engage pupils and provide cognitive and/or emotional rewards  Delivery Style. ***‘***Central to Y-Active implementation was the delivery style of the YMCA leaders who showed empathy and allowed pupils to choose their preferred activities.’  “We talk to the kids, we try and go down to their level and give them a one-to-one kind of personal “what would you like to do,” you know, and “do you think you’d be doing this, that” and try and get their opinion out there as well. (*Male Y-Active leader*)”  ‘Discipline was an integral part of the Y-Active delivery but this was balanced with positive encouragement and self-referenced feedback to the pupils:’  “I think sport needs to be disciplined, so if I’m focused and disciplined, I can’t have nobody chatting around because I’m gonna tell them straight up and say, “You know what? We got to do this.” A little smile goes a long way. . . . I really do believe that, I mean I know it sounds such a cliché but a little smile goes a long way and also you need to be doing it with them. It’s forever using the positive language I mean, “Come on you’re doing great that’s looking good that’s looking really good, you’re much quicker this week.” (*Male Y-Active leader*) (p.S244)  ‘The YMCA staff found self-referenced and task focused feedback to be particularly useful in motivating the children that were not as highly motivated toward football’:  “The kids don’t love football that much but I found that when they’re doing the steps [a timed run up and down steps in a local park] they remember the time for week 1, I write it down and I say, “this week you did this,” but those kids particularly those kids that don’t love the football that much they cant wait to get to the steps.” (*Male Y-Active leader*) (p.S244)  ‘The challenge of creating a balance between structure and discipline was also highlighted by the school teachers who recognized that this was achieved success fully by the YMCA staff:’  “I think its brilliant the way they balance the kind of play fun side of things with maintaining the discipline because in some senses it is easier in the class room . . . because you just maintain the discipline, you don’t do the kind of fun play side of things in the same way and I think that’s such a hard balance to create. (*Female teacher*)” (p.S244)  Challenges. ‘Developing rapport with the pupils was perceived as challenging as Y-Active leaders felt that they had to be different to the school teachers:’  “If you go there with a strict face and the tone of your voice, you aren’t gonna be getting too much anywhere. ‘Cause they might get that with their teachers, and whatever behavioral problems, when you step in you need to be someone different compared to the teacher.” (*Male Y-Active leader*) (p. S245)  ‘Another challenge was to ensure that the interpersonal style of the Y-Active leaders that focused on child autonomy was supported by the academic teachers. An unexpected finding was that the school teachers reported using the threat of withdrawal of pupils’ access to the Y-Active sessions as a contingent reward or disciplinary strategy:’  “It’s a nice carrot to dangle at the end of the week you know “you’ll miss football time” and they’re most distraught about. Other stuff they don’t care.” (*Male teacher*)  “There’s a special year 5–6 football club which happens during our golden time on Friday afternoon. So a lot of my boys miss that if they’ve not been behaving.” (*Female teacher*) (p. S245)  ‘This is a potentially concerning and unintended use of Y-Active. The purpose of Y-Active is to increase children’s PA and consequently their physical and mental health, therefore withdrawing access or threatening to do so may not only negatively affect time spent in PA but also create maladaptive motivation for good behavior in class (ie, not because it is the right thing to do, but because if they don’t, Y-Active is withdrawn).’ (p. S245) |
| 2e | Providing support materials that are appealing and appropriate to pupils’ age, interests and culture  NR |
| **3 – Embedding a programme into routine practice** | **The introduction and routine delivery (‘embedding’) of a programme takes time and motivation. It is likely to involve changes in the school environment and the development of new relationships between stakeholders that require pro-active management so that:** |
| 3a | - different stakeholders’ goals are reconciled - organisational decisions in other areas of school life are made taking into account how they impact on programme delivery - school staff’s existing relationships with children are built upon - stakeholders’ enthusiasm, knowledge and experience are harnessed - knowledge of ‘core’ and ‘peripheral’ elements and minimum resources, skills and informational content is retained - responsibility for programme delivery becomes rooted in the school   NR |
| 3b | The longer-term sustainability of programmes and the extent to which health promoting messages and activities permeate other aspects of school life, is dependent on continuing feedback, encouragement, and expectations about implementation. Over time, this may originate less from outside the school and more from inside.  NR |
| **4 - Programme adaptation** | **The preparation for, introduction, initial delivery, and ongoing sustainability of a health promotion programme in a school is more likely to be successful when there is:** |
| 4a | Specificity about ‘core’ (essential) and ‘peripheral’ (optional/adaptable) programme elements, including the minimum levels of resources and/or skills necessary to support these elements (to inform decision-making about how to deliver a programme in the context of a particular school)  NR |
| 4b | Scope for ‘mutual adaptation’ between the programme and the people delivering it, including the evolution and updating of programme content and mode of delivery over time  ‘The YMCA leaders also faced logistical challenges to successful implementation. Space, scheduling and equipment were cited as creating particular difficulties and YMCA leaders reported having to improvise session content based on the immediate school and staffing conditions. Further challenges faced by the YMCA leaders included role clarity, remuneration and irregular working hours. Such challenges have implications for the program more broadly in terms of staff retention and thus consistent program implementation. From their perspective, future provision should carefully allow for sufficient time for setting up their lessons:’  “It has been really hard to keep up this job actually ’cause the hours are quite strange. . . . It is really hard like I mean like I don’t work mornings, I don’t work nights; I work mornings and I work nights, sometimes, some days of the week. It is just really random so it is really hard to fit in another job in there and it can be a full time job because the classes, if you’re working with schools they are only at certain times.” (*Female Y-Active leader*) (p. S245) |
