# Supplementary material for: Implementing health promotion programmes in schools: a realist systematic review of research and experience in the United Kingdom
Source: Implement Sci. 2015 Oct 28;10:149. doi: 10.1186/s13012-015-0338-6 (PMC4625879; doi:10.1186/s13012-015-0338-6)
Supplement: Additional file 9: — Markers of implementation. Our decision-making was guided by looking for ‘markers of implementation’, although we did not limit extracted data to only these ‘markers’ if we judged other evidence to be relevant. (DOCX 19 kb) [file 13012_2015_338_MOESM9_ESM.docx]

| **PT#** | **Programme theory** | **Likely ‘markers’ of implementation** |
| --- | --- | --- |
| 1 | **Preparing for implementation**  Preparation for the introduction of a health promotion programme to a school is more likely to be successful when systematically planned in conjunction with other school responsibilities. This involves:   - identification of a potential health benefit for pupils at a local level - consultation with stakeholders (head-teacher, school staff, pupils, parents, governors) - consideration of the concordance of the programme with current practice and interests - identifying clear aims and priorities, including intended outcomes - taking into account the current situation and competencies in a school and the implications of these for programme refinement   *Consultation with stakeholders involves*:   - providing information on the programme (e.g. who and what is involved, evidence of effectiveness) - eliciting views on ‘readiness for change’ as a starting point for engagement - encouraging the sharing of previous experiences and knowledge of delivering health promotion programmes - facilitating discussion about the concordance (‘fit’) of the programme with:   - current practice  - pupils’ interests  - current school policies, resources and organisation | Uptake of programme  Documentation of consultation  Perceptions of consultative processes  Perceptions/ understanding of programme  Programme information (format, style, availability) |
| 2 | **Introducing a programme within a school**  The introduction of a health promotion programme to a school is more likely to be successful when it is incorporated into school activities through:   - being integrated into school policy (e.g. a School Improvement Plan) and supported by governors and senior staff to whom monitoring and progress reports are made - school staff using their leadership skills to co-ordinate activities or resources for programme delivery - providing adequate opportunity and/or training for the personal and professional development of those who will deliver the programme and engaging with their interests - modes of delivery that appeal to and engage pupils and provide cognitive and/or emotional rewards - providing support materials that are appealing and appropriate to pupils’ age, interests and culture | School policy documents  Programme documentation (e.g. if training is part of the programme ‘package’)  Knowledge about the programme and how it is proposed to be delivered  Uptake of training  Perceptions of programme delivery skills  Ability of those delivering the programme to deliver all programme components as designed |
| 3 | **Embedding a programme into routine practice**  The routine delivery (‘embedding’) of a programme takes time and motivation. It is likely to involve changes in the school environment and the development of new relationships between stakeholders that require pro-active management so that:   - different stakeholders’ goals are reconciled - organisational decisions in other areas of school life are made taking into account how they impact on programme delivery - school staff’s existing relationships with pupils are built upon - stakeholders’ enthusiasm, knowledge and experience are harnessed - knowledge of ‘core’ and ‘peripheral’ elements and minimum resources, skills and informational content is retained - responsibility for programme delivery becomes rooted in the school   The longer-term sustainability of programmes and the extent to which health promoting messages and activities permeate other aspects of school life, is dependent on continuing feedback, encouragement, and expectations about implementation. Over time, this may originate less from outside the school and more from inside. | Perceptions of programme implementation  Participation/ engagement in programme  Observation of programme management  Uptake of programme in schools other than those being evaluated |
| 4 | **Programme adaptation**  The preparation for, introduction, initial delivery, and ongoing sustainability of a health promotion programme in a school is more likely to be successful when there is:   - specificity about ‘core’ (essential) and ‘peripheral’ (optional/adaptable) programme elements, including the minimum levels of resources and/or skills necessary to support these elements (to inform decision-making about how to deliver a programme in the context of a particular school) - scope for ‘mutual adaptation’ between the programme and the people delivering it, including the evolution and updating of programme content and mode of delivery over time | Description of the components and function of each programme element |
