# Supplementary material for: Implementing health promotion programmes in schools: a realist systematic review of research and experience in the United Kingdom
Source: Implement Sci. 2015 Oct 28;10:149. doi: 10.1186/s13012-015-0338-6 (PMC4625879; doi:10.1186/s13012-015-0338-6)
Supplement: Additional file 10: — Practical stages of the synthesis. The file lists the details of the practical stages of the synthesis. (DOCX 17 kb) [file 13012_2015_338_MOESM10_ESM.docx]

In practical terms, the process of synthesis was:

1) Both reviewers read ‘across’ the element of programme theory (e.g. 1a, 1b, etc.) from the data extraction tables to (re-)familiarise themselves with the sources of evidence and develop broad themes that could help express the synthesis.

2) Notes were produced in a way that we considered best for facilitating the process of analysis and synthesis.

3) An initial synthesis (explanation) of context-mechanism-outcome configurations was produced. This involved *juxtaposing*, *reconciling*, *consolidating*, *situating*, and *adjudicating* between sources of evidence (see Table below). The synthesis could be written informally, but had to include explicit reference to the sources of evidence. The point of the exercise was to facilitate the reviewer’s analytical abilities and to produce material that could be shared, discussed and critiqued with the other reviewer.

4) The initial synthesis was iteratively developed through further discussion between the two reviewers, to ensure comprehensive treatment of all extracted data and coherency in the way that it was synthesised. We sought to move forward from a more rudimentary understanding of implementation issues (e.g. as expressed in themes) to one that was ‘deeper’ and more complex (i.e. which expressed the ‘actual’ mechanisms at play within particular context and outcome configurations). We decided on the number of iterations based on the extent to which extracted data could continue to contribute to programme theory testing and refinement, analogous to the notion of ‘saturation’ used in qualitative research. Testing and refinement could also cast new light on ‘previously used’ data that would contribute anew to further theory testing and refinement.

Synthesis process - definition of terms

| Consolidate | To bring together - in a realist synthesis, ‘to bring together *into a more coherent whole’* |
| --- | --- |
| Juxtapose | To place two or more things (evidence fragments) together, especially in order to suggest a link between them or emphasise the contrast between them |
| Reconcile | To make two or more apparently conflicting things (evidence fragments) consistent or compatible |
| Situate | To place something (a piece or pieces of evidence) in a context or set of circumstances and show the connections (between it/them and other evidence fragments) |
