# Supplementary material for: Implementing health promotion programmes in schools: a realist systematic review of research and experience in the United Kingdom
Source: Implement Sci. 2015 Oct 28;10:149. doi: 10.1186/s13012-015-0338-6 (PMC4625879; doi:10.1186/s13012-015-0338-6)
Supplement: Additional file 11: — Long version of synthesis. The file contains a long version of the findings, with examples and greater detail about the rigour of individual pieces of evidence used in the synthesis. (DOCX 84 kb) [file 13012_2015_338_MOESM11_ESM.docx]

## Findings: Programme theory 1

**Preparation for the introduction of a health promotion programme to a school is more likely to be successful when systematically planned in conjunction with other school responsibilities. This involves:**

- Consultation with stakeholders, which involves:
  - providing information on the programme (e.g. who and what is involved, evidence of effectiveness)

Evaluations of three programmes (Audrey et al., 2004, Audrey et al., 2008, Lowden and Powney, 1996, Blueprint Evaluation Team, 2007) provided information about this initial stage. An evaluation of the Greater Glasgow Health Board (GGHB) sex and relationship education (SRE) programme reported few details about the information provided during consultation with stakeholders. However, it was reported that the teachers and health worker involved with programme delivery had four one-hour sessions where programme materials were ‘scrutinised and discussed’ and, it is suggested, refined (Lowden and Powney, 1996,p.28). Informal assessment of teachers’ views suggested that this process engaged them and facilitated their delivery of the programme (Lowden and Powney, 1996), but social desirability bias was not considered. An evaluation of the Blueprint substance use education programme documented how, prior to programme delivery, consultation took place with schools at managerial and staff levels and how agreement was obtained for a named staff member to take on a co-ordination and liaison role for programme delivery (Blueprint Evaluation Team, 2007). However, the impact of this consultative process remains unclear in the evaluation.

The process of initial engagement with teachers was quite different in the ASSIST (smoking prevention) programme, which trained pupils in each school to provide information, advice and support to their peers (Audrey et al., 2004) rather than the more conventional teaching and small group work model used in the GGHB SRE programme (Lowden and Powney, 1996). The ASSIST programme explicitly negotiated and agreed what the school would do to enable programme delivery - this included researcher access to pupils, time and support for the peer-supporters to attend off-site training, and on-site follow-up visits from researchers (Audrey et al., 2004). A senior teacher was also enrolled at each school to assume responsibility for the co-ordination of programme delivery, which included negotiating with teachers when ‘peer-supporters’ would be absent from lessons. Drawing on teacher interview data from a well-conducted process evaluation, there is evidence to suggest that this consultative approach enabled implementation issues to be addressed in a timely fashion. For example, some teachers were initially resistant to allowing pupils who had a history of truancy or disruptive behaviour to become peer-supporters in the programme. However, discussion about these issues took place during the consultation process and, it is suggested, enabled the programme to recruit peer-supporters who were ‘balanced in terms of gender and broadly representative of their peer group’ (Audrey et al., 2008, p.83). More broadly, although only approximately 50% of the schools approached to take part in the trial wanted to participate, none of those who chose to participate withdrew from the implementation of the programme (Audrey et al., 2008). All of these findings should perhaps be considered in relation to the potential positive impact that ‘being studied’ has on levels of teacher and pupil engagement. In the RIPPLE evaluation, reflection on the fieldnotes in which pre-trial arm allocation discussion with teachers was documented led the authors to conclude that ‘the greater engagement of both students and teachers in the experimental as compared with the control schools… raises the possibility that the impact of ‘being studied’ may have been greater for participants in the experimental arm’ (Oakley et al., 2004, p.456).

- - eliciting views on ‘readiness for change’ as a starting point for engagement

In both programmes that reported relevant data, there was a perception that the programmes addressed important and relevant issues - obesity (Sahota et al., 2001) and well-being (Challen et al., 2009). A survey of school staff and parents informed the design and development of the APPLES programme (obesity prevention), achieving an overall response rate of 64% (school staff n=124; parents n=410) (Sahota et al., 2001). On most survey items relating to the promotion of healthy eating in schools, the responses of over 85% of staff and teachers indicated their acknowledgement of the importance of children’s diets (Sahota et al., 2001). The APPLES programme also used the survey to elicit parents’ views on changes that they would like to see within schools to promote healthy eating - these findings were integrated into school action plans that contained monitoring and feedback mechanisms by which progress could be monitored (Sahota et al., 2001). In the UK Resilience Programme (well-being) participating teachers (some of whom had volunteered, others who had been suggested as suitable by senior managers) attended a two-week programme delivery workshop in the USA (Challen et al., 2009). The evaluation suggests that, despite the commitment required from teachers to prepare for the workshop during the summer holidays, the novelty of the overseas training programme may have impacted positively on motivation (Challen et al., 2009). However, the extent to which this connection can be made is unclear from the reported data.

- - encouraging the sharing of previous experiences and knowledge of delivering health promotion programmes

The role of prior experience in delivering health promotion programmes was identified as important for teachers to ‘take on’ the delivery of novel health promotion programmes in schools. In an area such as SRE, where the sensitivity of the topic is acknowledged to make delivery more difficult, the confidence of teachers in delivering SRE in the GGHB and SHARE programmes was strongly related to the extent of their prior experience with SRE as a whole (Lowden and Powney, 1996, Wight and Buston, 2003), the specific elements that they were required to teach (Wight and Buston, 2003), and the way in which the programme was to be delivered (for example, teachers were comfortable with a teacher-led approach that they perceived would minimise disruption and improve discipline) (Lowden and Powney, 1996). In SRE, teachers were most confident providing information to pupils rather than more interactive activities, although small groups were preferred (Wight and Buston, 2003). Delivering SRE that was consistent with their own values was also identified as important for their confidence in programme delivery (Wight and Buston, 2003). Note that these associations were based on group interview data for which the sampling and data analysis strategies were not reported (Lowden and Powney, 1996), and a survey for which the number of respondents, response rate, and sampling strategy were not reported (Wight and Buston, 2003).

For a programme promoting physical activity, where there is probably less sensitivity around the topic, the extent to which the sharing of previous experiences impacts on programme delivery can be much smaller. For example, an established interest in physical activity promotion at local authority and school management level was claimed to facilitate the uptake of the Active Primary School pilot (Lowden et al., 2004). On the basis of this interview data, for which very limited information about collection and analysis was reported, it was also claimed that where local interest was already established, programme delivery was facilitated by the associated networks and partnerships that were in existence and which could provide a framework for the delivery of a programme (Lowden et al., 2004).

A ‘sensitive’ health promotion area such as SRE could also be well-established within a school, but this may not facilitate implementation in a straightforward fashion. It is suggested, based on in-depth interviews with teachers for which data collection and analysis was thoroughly described and findings strongly-contextualised, that the way in which a SRE programme such as SHARE is introduced to a school should be decided based on knowledge of the school’s recent history in relation to SRE (Buston et al., 2001). For example, the approach would differ in a school where one member of staff had led SRE for a number of years (and was reluctant to make any changes to pupils’ SRE) and a school where a growing working relationship between a head teacher and school nurse provided quite different opportunities for programme delivery (Buston et al., 2001). A related finding, although based on a weakly described analysis of qualitative data, was reported in the evaluation of the UK Resilience Programme (Challen et al., 2009). Across 29 interviews with school staff it was ‘uniformly reported’ that the personal characteristics of staff, such as responsiveness and sensitivity, that were the most important factor in successfully delivering a programme that involved well-being topics that could be emotionally and psychologically sensitive for pupils (Challen et al., 2009).

In an instance where senior staff had prior negative experiences with a similar programme, Challen et al. (2009) observed that time, and the completion of staff training was required, before senior staff could be ‘convinced’ of the programme’s value and to provide their support.

During the introduction of all four of the above programmes, it is apparent that some form of ‘experience sharing’ has taken place at some level of school organisation, but the precise form that this consultation has taken (and the associated strengths of weaknesses of different approaches) remains unclear.

- - facilitating discussion about the concordance (‘fit’) of the programme with:
    - current practice
    - pupils’ interests
    - current school policies, resources and organisation

Evaluations identified areas where issues arose that were related to the extent to which consultation had taken place. At a fundamental level, across a range of health promotion areas, the importance of programmes appealing to pupil’s interests was identified. For example, an obesity prevention programme’s approach was confirmed by teachers to be appropriate to the targeted pupil’s developmental stage (Wyatt et al., 2011), whilst a smoking prevention programme’s delivery was perceived by some teachers to be hampered through being ‘meaningless’ to pupils from non-smoking families (Newman and Nutbeam, 1989, Newman et al., 1991). In an area such as sexual health, designing a programme to engage children of both sexes who are maturing at different rates can be problematic (Buston et al., 2002a). Similarly, Lowden and Powney (1996) report that differences in maturity between boys and girls of the same age can make mixed-sex group work problematic, but no supporting data is presented. Whilst the developmentally appropriate areas of pupils’ interest in an area of health can be established in a school through, for example, a survey, the extent to which this enables tailoring of programme delivery in classes is unclear (Lowden and Powney, 1996).

In SRE, appealing to pupils’ interests can require consideration of more than programme appropriateness to pupils’ developmental age and making content specific to pupils’ information needs. For example, in an extensive process evaluation across 25 schools, where pupils’ views did not align wholly with the non-discriminatory nature of the SHARE programme, pupils were perceived to express their discomfort in programme sessions through negative or hostile behaviour (Buston and Hart, 2001):

“...pupils at that age, it [same-sex sex] is just a no-no, it’s disgusting, how could they possibly? Especially boys. But, and if you say ‘well everyone’s entitled to their own views and live the way they choose’, they’re just not comfortable with that at all. And I just don’t know how you could get that over.” (female teacher, aged 31-40y) (Buston and Hart, 2001, p.103)

This teacher’s perceptions were borne out by the pupil survey conducted in the same programme, which found that while nearly half (48.8%) of pupils ‘agreed’ or ‘strongly agreed’ that ‘people should be free to have sexual relationships with people of the same sex’, just over a quarter (25.8%) of pupils ‘disagreed’ or ‘strongly disagreed’(Buston and Hart, 2001). A ‘peer-supporter’ smoking prevention programme (ASSIST) suggests, based on teachers’ perceptions, that appealing to pupils’ interests can be achieved simply by providing a change from teacher and school-based learning. For example, teachers viewed the training of peer-supporters (who were an integral part of programme delivery) by an external team to be important both for creating additional interest amongst the peer-supporters and providing an environment in which they could openly discuss smoking behaviour (Audrey et al., 2008). Peer-supporters could also be motivated by the way in which the programme could enable them to develop transferable skills that would help them in their school and working life, a potential benefit of taking part in the programme that was stressed at the peer-supporter recruitment meeting (Audrey et al., 2004).

The extent to which health promotion programmes could ‘work with’ the existing commitments of both teachers and pupils was a recurring theme. At its most basic, if a programme’s structure clashed with the structure of a school timetable then problems with implementation arose at the outset (Newman et al., 1991). However, the educational, pastoral, and professional demands placed on teachers and the educational and developmental demands placed on pupils meant that difficulties were often found in trying to fit a health promotion programme into an already very busy timetable. This could occur particularly around the time that pupils were sitting exams such as SATs (Stathi et al., 2006) but also more generally within the timetable at both primary (e.g. Lowden et al., 2004) and secondary schools (Buston et al., 2002b):

“I don’t think I could usually fit in more than 6 [sessions a year]. I mean this [SHARE health promotion programme] has really distorted my whole programme... I’ve not done any study skills, I’ve not done anything on drugs or alcohol, I’ve hardly done any work experience.” (teacher) (Buston et al., 2002b, p.66)

There was a feeling amongst some teachers that a mis-match between programme demands and space in their workload and the timetable impinged significantly on programme delivery. For example, in the SHARE programme some teachers reported that they had insufficient time for SRE session preparation or reflection on their own delivery of the programme (Buston et al., 2001), but 10 of 13 intervention schools found the time within the school day to deliver the programme successfully (Wight and Buston, 2003). In the ASSIST programme some teachers reported that, whilst they were supportive of the programme, it imposed additional administrative demands on teachers who had to re-organise lessons or tests so that peer-supporters could attend training (Audrey et al., 2008). In the SHARE programme, a downward spiral in programme delivery could be initiated when for some teachers the initial lack of defined preparation time impacted on the extent and quality of programme delivery and a sense amongst some teaching staff that they were delivering the SRE programme without the level of guidance that they felt was needed (Buston et al., 2001). Some schools delivering the ASSIST programme overcame timetabling conflicts for the delivery of peer-supporter training by scheduling this to take place in a way that impacted equitably on other subjects within the timetable (Audrey et al., 2008).

Teachers identified the important role of senior support in timetabling that enabled teachers to meet all of their work requirements in a well-being health promotion programme (Challen et al., 2009). Similarly, the problems caused by a lack of co-ordination of programme activities with other school lessons, health promotion initiatives, or PSHE activities were identified by teachers in a healthy eating programme (Teeman et al., 2008). An alternative approach was used in the delivery of the ASSIST programme; in response to teachers’ feedback, this programme targeted pupils aged 12-13 years because at this age between SATs and GCSEs there was a ‘window of opportunity’ in the time pupils and teachers had available for health promotion activities (Audrey et al., 2008). This was of particular importance for the ASSIST programme, as peer-supporters (who were delivering the programme in schools) were the same age as the pupils who were the intended programme participants. The high completion rate (90%) of peer-supporter training (2 days), 10-week peer-supporter role (82% - judged through assessment of peer-supporters’ reflective diaries) (Audrey et al., 2004) and participation in >=75% of the four follow-up sessions (89%) (Holliday et al., 2009) suggests that the ‘window of opportunity’ was important for the delivery of this programme.

Programmes that endeavoured to address teachers’ time issues through programme delivery being primarily achieved using external providers were largely appreciated by teachers (Audrey et al., 2008, Lowden et al., 2004).

Finally, concordance between local public health policy and the focus of a school health promotion programme could support delivery (Lowden and Powney, 1996), as could the degree of ‘fit’ between school policy (for example, in the form of a ‘whole school approach’) and the health promotion topic (Stathi et al., 2006).

- Identification of a potential health benefit for pupils at a local level

One evaluation of a smoking prevention programme (ASSIST) reported, on the basis of interviews with teachers, how teachers’ perceptions of a local health need could facilitate the uptake of a relevant health promotion programme (Audrey et al., 2008). In this instance, staff welcomed a smoking prevention programme as they believed their own efforts to discourage pupils from smoking were ineffective:

“We discuss and we sort of run out of ideas really, when it comes to smokers on the field and to what degree punishing them, writing letters home, informing parents. All that stuff is a bit, it’s negative and not very effective. So to have an approach which is more thought out, and more involving kids, involving pupils being more active and spreading the message, yeah it’s good.” (Head of Year 8) (Audrey et al., 2008, p.85)

- Consultation with stakeholders (head-teacher, school staff, pupils, parents, governors)

At a basic level, discussion between programme designers and teachers was identified as important by teachers. Lowden and Powney (1996) report that by collaborating with teachers at the stage of programme design and incorporating their feedback into the design and content of a SRE programme, teachers’ confidence in and enthusiasm for the programme was significantly boosted. This claim is based on interview data for which neither the sampling, data analysis strategies or underlying data were reported. A more in-depth and extensive process evaluation (Buston et al., 2001) for which sampling, data collection and data analysis processes were thoroughly reported, suggests that consultation with schools about a SRE programme is more complex because of the need to meet the real or perceived demands of parents. This could involve striking the right balance between academic education and health promotion in the school timetable so that parents’ stated expectations about academic achievement were met (Buston et al., 2001). However, specific consultation about the content or extent of SRE programmes occurred in only a minority of the 13 intervention schools in the SHARE trial, and even where it did occur, this tended to be with a small number of parents (Buston et al., 2001) . Engaging parents on a topic which, it is implied, many of them felt uncomfortable discussing and about which there could be sensitivities that teachers are highly reluctant to broach, meant that consulting parents could prove very difficult (Buston et al., 2001).

- Consideration of the concordance of the programme with current practice and interests

Four evaluations reported how concordance between a programme and other ongoing school initiatives could facilitate uptake at both primary and secondary school levels (Audrey et al., 2008, Lowden et al., 2004, Teeman et al., 2008, Wyatt et al., 2011). At the primary school level, it was observed that teachers valued the HeLP obesity prevention programme for the way that it met National Curriculum guidelines for PSHE and facilitated engagement with parents (Wyatt et al., 2011). Similarly, school staff valued the PhunkyFoods programme for the way in which it could contribute to the National Healthy Schools Standard accreditation (Teeman et al., 2008). In the Active Primary School programme, teachers generally responded positively to programme staff who organised physical activities that the school did not usually provide (Lowden et al., 2004). It is reported on the basis of interviews with headteachers that teachers mostly responded well to the coaching provided by programme staff, although there is no data reported to support this (Lowden et al., 2004). In both of these examples, ‘concordance’ is very much about addressing an unmet need in schools in a way that fits well with other school activities.

At the secondary school level, the ASSIST smoking prevention programme’s focus on developing the skills of peer-supporters (who would in turn use their skills to help their peers) fitted well with a wider school ethos promoting pupil autonomy and responsibility (Audrey et al., 2008). Although the number of schools in which this occurred is not reported, teachers identified its role in interviews, for example:

“Health education might be the particular message but I think it fits into a much broader spectrum about education in general, about giving kids confidence with information to actually go and talk to other people and put forward a point of view.” (Guidance co-ordinator) (Audrey et al., 2008, p.85)

Concordance could also be an aim of programme delivery in that it was something necessary to achieve in order that time and resources were co-ordinated across a school (Teeman et al., 2008). This could manifest in terms of ensuring teachers had sufficient time to incorporate a programme into their curriculum plans or co-ordinating delivery so that programme materials were not stretched across too many classes at one time (Teeman et al., 2008).

In certain instances, a level of discordance between a programme and school activities could act in a positive way to stimulate change and, ultimately, work towards achieving concordance between the programme and wider school activities and environment. In the PhunkyFoods obesity prevention programme, staff in one primary school reported that seeing the positive response of the pupils to the range of activities in the programme had prompted them to re-examine other aspects of the curriculum, although there are weaknesses in the reporting of how this interview data was analysed (Teeman et al., 2008). At the secondary school level, the development of the SHARE SRE programme involved striking a balance between the theoretically-informed programme components and teachers’ views based on their professional experience, a process described by the researchers as requiring ‘delicate negotiations ... to achieve a successful compromise of social-cognitive theory and orthodox health education principles’ (Wight and Abraham, 2000, p.30). Based on critical reflection on the process, it is argued by the researchers that the involvement of a respected sex education consultant and statutory funding for programme development provided the credibility that the programme needed to have in teachers’ eyes for the ‘delicate negotiations’ to be successful (Wight and Abraham, 2000).

- Identifying clear aims and priorities, including intended outcomes

No evidence identified for this sub-theory.

- Taking into account the current situation and competencies in a school and the implications of these for programme refinement

A number of evaluations reporetd issues that helped or hindered programme delivery in terms of the extent to which the current situation and competencies were accounted for by the programme. This evidence was descriptive and therefore synthesis in a higher-level summary was not possible. However, this descriptive data may still prove informative, for example:

- Where teaching assistants were charged with programme delivery, the prescriptive detail of lesson plans in PhunkyFoods was highly valued (Teeman et al., 2008)
- Simple time shortages in the timetable could impact significantly on the extent to which a programme was actually delivered (Crosswaite et al., 2004, Oakley et al., 2004, Wight et al., 2002); sometimes these pressures were perceived to be as a result of a low strategic priority being given to health promotion within a school (Wight et al., 2002)
- Resources to pay for supply teachers to cover teachers’ other teaching commitments (i.e. those they were unable to fulfil when committed to health promotion programme delivery) was reported by Stead et al. (2001) to be appreciated by teachers and to facilitate programme delivery. Teachers also perceived the provision of such resources to be a sign of genuine strategic commitment to a programme (Stead et al., 2001)
- Pupils identified factors that made it more difficult for peer-educators to deliver SRE, although the extent of these factors is not clear from the reported data:
  - inappropriate classroom space (e.g. science laboratories)
  - a perceived lack of support for the programme by some teachers
  - the length of time between the training of peer-educators and their delivery of the programme
- Risk-averseness, e.g. cautious about promoting physical activity where play surfaces were not ideal (but were not dangerous) (Lowden et al., 2004)
- Physical resources within a school - for example, where sports hall facilities, playing fields and levels of sporting equipment were perceived as insufficient for implementing a physical activity promotion programme; (Lowden et al., 2004); where schools did not always have kitchen facilities in which pupils could undertake the programme components which involved cooking in an obesity prevention programme (Teeman et al., 2008)

## Findings: Programme theory 2

**The introduction of a health promotion programme to a school is more likely to be successful when it is incorporated into school activities through:**

- Being integrated into school policy (e.g. School Improvement Plan) and supported by governors and senior staff to whom monitoring and progress reports are made

Evaluations covering a wide range of health promotion topics (physical activity, obesity, smoking, SRE, well-being) in both primary school (Lowden et al., 2004, Sahota et al., 2001) and secondary school settings (Buston and Hart, 2001, Challen et al., 2009, Holliday et al., 2009, Oakley et al., 2004) highlighted the role of senior support in enabling programme delivery. However, this evidence is largely descriptive rather than analytic, providing simple measures of uptake rather than analyses of explanatory processes. In primary school settings, reference is made in evaluations to increased implementation of aspects of school action plans related to the programmes (Lowden et al., 2004, Sahota et al., 2001), but these causal links are made on the basis of weakly-conducted process evaluations for which the underlying data is not reported.

In secondary school settings, the process evaluations conducted were of a higher quality and reported similar findings about the important role of senior support for a programme (Challen et al., 2009, Oakley et al., 2004), but the extent and depth of evidence for such causal assertions remains unreported. Reports can be found in process evaluations about negative experiences that suggest that senior support for programme delivery was either not forthcoming or partial. This could be in the form of ‘being landed with’ unsuitable classroom facilities, such as a science laboratory where fixtures and fittings inhibited the group work that was a core part of a smoking prevention programme (Holliday et al., 2009). In a SRE programme in which data collection and analysis was thoroughly reported, perceived non-integration with school policy and lack of senior support left some teachers feeling ‘on their own’ in their delivery of non-discriminatory SRE. For these teachers, there was a tension between programme content and school policy:

“We are restrained by instructions from our bosses to an extent as to where you can go with it [discussing homosexuality]... teachers don’t have a free hand.” (male teacher, aged 31-40y) (Buston and Hart, 2001, p.101)

Teachers who felt that they were at the mercy of national policy, media ‘exposure’ about teaching practices and topics, and their legal position with regard to teaching SRE which explicitly incorporated homosexuality, could therefore substantially limit aspects of SRE programme delivery when they felt unsupported by senior staff (Buston and Hart, 2001).

- School staff using their leadership skils to co-ordinate activities or resources for programme delivery

Evaluations covering a wide range of health promotion topics (physical activity, obesity, smoking, SRE) in both primary school (Lowden et al., 2004, Stathi and Sebire, 2011, Teeman et al., 2008) and secondary school settings (Holliday et al., 2009) reported how leadership skills contributed to the co-ordination of activities and resources for programme delivery. However, the evidence reported relates more to the pivotal role of a named co-ordinator than staff members as a whole using their leadership skills.

The evidence from primary schools is drawn from quite different stages of programme delivery, although all relating to obesity prevention programmes. These range from a physical activity programme established in a single school over five years (Stathi and Sebire, 2011) to evaluations across between 11 and 20 schools of recently introduced programmes (Lowden et al., 2004, Teeman et al., 2008). There were considerable study limitations in both of the process evaluations of recently introduced programmes, with the links between analysis and underlying data remaining unclear. However, in both of these programmes, the pivotal role of a co-ordinator was identified - in the Active Primary Schools programme, headteachers ‘often emphasised’ that the full-time co-ordinator employed by the programme played a vital role in promoting and sustaining delivery in schools, although this was also dependent on having support for the programme at a senior level within the school (Lowden et al., 2004, p.31). In the PhunkyFoods programme, greater success in programme delivery was reported in schools where co-ordinators were teaching staff or had an existing co-ordination role (five of the 18 schools in which staff perceived insufficient time for programme delivery were able to deliver at least ‘some’ elements of the programme - all of these schools had a co-ordinator who was a member of teaching staff) (Teeman et al., 2008). It is reported that Teaching Assistants who had the role of co-ordinator felt that they did not have the power or influence to address issues such as timetabling which impacted on programme delivery, although the data underlying this claim is not reported (Teeman et al., 2008).

In the more established primary school obesity prevention programme (Y-Active), in which all aspects of the process evaluation were thoroughly conducted, the importance of a school co-ordinator who had the time and resources to liaise between school management/staff and YMCA staff who were delivering the programme, was identified (Stathi and Sebire, 2011). However, neither the number of schools in which this was identified nor an analysis of data in addition to staff perceptions is reported. The importance of senior support for the programme, both in strategic and practical terms, is again identified through interviews with those delivering the programme, for example:

“We work very closely with the head teacher. Her door is always open… There’s a lot of communication in that school and the teachers are very on board with what we do and they support us in any way…” (Female YMCA programme leader) (Stathi and Sebire, 2011, p.S244)

Similar experiences were reported in secondary school settings in a smoking prevention programme (ASSIST). Although there were limitations in the process evaluation regarding the sampling strategy of schools, data analysis is fully reported and the potential impact of researchers and peer-trainers sharing office space is candidly reflected upon. In the ASSIST programme, the motivation and ability of school co-ordinators to fulfil their role reflected both their personal characteristics and those of the school (in terms of levels of support for co-ordinators). For example:

“… at [school, the school co-ordinator has] got so many other responsibilities in the school I know it has been hard for her to get messages round and although she has done it, often it has been quite late in the day and often we have had to ring up and remind her… and then you get the other extreme, you know, [teacher] at [school] everything is just so organised you know.” (Trainer) (Holliday et al., 2009, p.56)

The role of a co-ordinator was not identified in the process evaluation of a secondary school SRE programme (RIPPLE) where programme delivery was peer-to-peer. In this case, teachers’ leadership ideally took the form of enabling peer-educators to deliver the programme through facilitating access to resources, advising on classroom management, or simply recognising the value of peer-educators’ role in health promotion (Strange et al., 2002b). A survey of peer-educators who responded found that 10% of peer-educators did not feel supported by teachers, but that 34% did feel they were well-supported (Strange et al., 2002b).

- Providing adequate opportunity and/or training for the personal and professional development of those who will deliver the programme and engaging with their interests

The personal and professional development of people delivering a health promotion programme related to needs in a number of areas, namely knowledge, motivation, confidence, skills and reflection on personal values. The diversity of approaches in health promotion programmes in schools means that whilst the ‘programme deliverers’ are often teaching staff, they may be working in close collaboration with an external agency or, in the case of peer-education programmes, fulfilling a support role for the pupils who are providing the ‘final delivery’ of a programme.

Broadly, teachers welcomed opportunities to enhance their knowledge when this would improve their delivery of a health promotion programme. Knowledge needs could take quite different forms. For example, in an obesity prevention programme in a primary school, aspects of ‘training’ could take the form of providing fresh ideas for how to deliver novel physical activities that would engage pupils (Lowden et al., 2004). In contrast, in an area such as substance use, where pupils’ knowledge of some aspects could significantly out-strip teachers’, the opportunity for teachers to learn the names and effects of new substances was welcomed (Stead et al., 2001). Even where school staff might not have been aware of what they did not know, for example in a programme promoting well-being in pupils, their response to training could be positive (Challen et al., 2009).

Process evaluations identified issues in two distinct areas relating to teaching staff’s confidence in programme delivery. First, in both primary and secondary school settings, teaching staff identified a barrier to programme delivery as the lack of coverage of issues around class control when delivering a programme that contained novel physical or interactive activities (Stead et al., 2007b, Teeman et al., 2008). Second, in secondary school settings where SRE and substance use programmes address more sensitive health issues, training significantly increased teaching staff’s confidence in programme delivery. In the Blueprint substance use prevention programme, 97% of teachers rated the ‘delivery practice’ component of the training programme as ‘useful’ (Stead et al., 2007a), whilst in the SHARE SRE programme the combination of delivery practice and group reflection training components was identified as important in increasing teachers’ confidence in delivery (Wight and Buston, 2003). This was assessed through interviews with teachers in which teachers ‘frequently’ mentioned that the training had made them more comfortable or more confident in delivering SRE (Wight and Buston, 2003, p.529). For example, the opportunity to experience handling ‘awkward questions’ outside of the classroom was valued by this teacher:

“I was fairly comfortable with a lot of the approaches already. And that made it an awful lot easier for me than some other people. Mm, one of the questions we had to practice… was ‘How do girls masturbate?’ and I did think I’d never be asked that… and I was really glad of that opportunity, because I have been asked that since teaching the course.” (Teacher) (Wight and Buston, 2003, p.529)

However, training cannot improve the confidence of programme deliverers for all areas of a programme’s topics. An extensive and thoroughly conducted survey across schools delivering the SHARE programme established that teachers were more likely to report feeling ‘confident’ or ‘very confident’ teaching about more indirect issues relating to sex (such as contraception or sexually-transmitted diseases) than issues relating to homosexuality or explicit details about sexual activities other than sexual intercourse, despite both topics being addressed in training (Buston and Hart, 2001).

The impact of teachers’ values on the delivery of health promotion programmes is only reported in relation to SRE, and its potential impact in other areas of health promotion remains unclear. In the SHARE SRE programme, some teachers experienced siginificant discomfort in delivering a programme that was in some respects in tension with their own values. For example:

“I’m never that comfortable talking about… homosexuality… Some people say… it should be a compulsory part of the sex education programme and I’m not comfortable with that.” (Male teacher, aged 31-40y) (Buston and Hart, 2001, p.101)

In other situations, teachers found that their values could be a teaching resource, albeit one that they were sometimes afraid to use for fear of not remaining ‘neutral’:

“… trite comments [from pupils]… I take up and expand and react. You know, [in a discussion about homosexuality] the big lad saying ‘just so long as he doesn’t follow me with his bar of soap’. The other day I said ‘what makes you think he would fancy you Derek?’ (Female teacher, aged 31-40y) (Buston and Hart, 2001, p.102)

The extent to which training enabled teachers to use their values in a way that was consistent with programme delivery remains unclear. Specifically in relation to SRE, interviews with teachers suggested that there may be inherent difficulties with addressing a topic that by its very nature is personal and has the potential to provoke discomfort in teachers (Buston et al., 2001). This suggests there may be limits to the extent to which training can address difficult and personal issues around SRE programme delivery, as expressed by this interviewee:

“[SRE is] a difficult issue for people to handle… I’m not criticising people for finding these things difficult, they are difficult.” (emphasis in original text) (Male Associate Head Teacher) (Buston et al., 2001, p.357)

Evidence about the impact of engaging the interests of those delivering the programme is limited to the engagement of peer-educators in the ASSIST and RIPPLE programmes. Both of these programmes reported success in recruiting the proposed numbers and characteristics of pupils, but from the reported data it is difficult to explain why the motivations of pupils for becoming peer-educators differed so markedly in the two programmes. In the ASSIST smoking prevention programme, interviews and focus groups with peer-supporters suggested a number of reasons for pupils volunteering as peer-educators. These reasons were a mixture of the personal (e.g. pride at being nominated by peers; a commitment to reducing smoking) and more emotional reasons relating to the novelty of the programme (which provided the opportunity to participate in training outside of school and being able to take ‘time off’ school to attend follow-up lessons) (Audrey et al., 2006a). In contrast, in the RIPPLE SRE programme, focus groups with peer-educators suggested that the way in which the programme had helped pupils to develop transferable skills (e.g. confidence in speaking to large groups, presenting information, and talking about sensitive issues) was a significant element in engaging them in project delivery (Strange et al., 2002a). Post-programme surveys with peer-educators also identified the role of the programme in developing peer-educators’ transferable skills, with 66% ‘agreeing or strongly agreeing’ that their involvement as a peer-educator in the programme would be useful to their life outside of school (Strange et al., 2002a). It is not clear whether it is the way that the programmes were presented to the potential peer-educators, the focus of the interviews and focus groups, or differences related to the health promotion topics or the way that peer-education was delivered that accounted for the differences in these findings.

- Modes of delivery that appeal to and engage pupils and provide cognitive and/or emotional rewards

All included studies alluded to the perceptions of pupils and teachers about the importance for engagement in health promotion programmes of them being appealing to pupils. However, the included process evaluations reported little assessment about the cognitive components of engagement such as reasoning and decision-making. This was mostly limited to the extent to which programme content was perceived by pupils to be relevant to their own lives. For example, in the RIPPLE SRE programme, peer-led lessons were mentioned in >85% of 41 focus groups as providing novel and relevant information about sex and relationships, whereas teacher-led lessons were only mentioned in 40% of 10 focus groups in this respect (Forrest et al., 2002). The characteristics of pupils’ behaviour in a local area may require a health promotion programme to be adapted so that it addresses issues high on their agenda. For example, older female pupils (aged 15-16y) in an area where sexual relationships and pregnancy were ‘normalised’ within their social networks, felt that the GGHB SRE programme did not address their needs and that teachers were unwilling to discuss issues around HIV, contraception and pregnancy (Lowden and Powney, 1996).

In a physical activity promotion programme (Schools on the Move) delivered in both primary and secondary schools, pupil survey results about motivations for participating in the programme are reported. The study authors summarise these motivations as: feeling competent about the tasks in the programme; choosing to take part; feeling that the programme was valuable and useful; and, feeling meaningfully related to other participants in the project (Stathi et al., 2006). However, only partial and summarised data that cannot be explicitly linked to these claims is provided.

Evidence about the impact of emotional reward in health promotion programmes delivered in primary schools is largely descriptive rather than analytic, and sourced from observations of practice, focus groups, or interviews in which only summary data is reported. These consistently suggest across different modes of delivery (e.g. tasks, quizzes, practicals, drama) that pupils need to find the activities fun to take part in (Crosswaite et al., 2004, Rothwell and Segrott, 2011, Stathi et al., 2006, Teeman et al., 2008, Warren et al., 2003, Wyatt et al., 2011). Well-conducted process evaluations of SRE programmes in secondary schools (RIPPLE and SHARE) report a similar finding, although in this context ‘having fun’ is identified not only as a way of engaging pupils but of helping them to discuss and ‘deal with’ a difficult and personal topic. In the RIPPLE programme, there was a statistically significant difference, favouring peer-led provision, in ‘pupil enjoyment’ between peer-led and teacher-led SRE (OR 2.15 (95%CI 1.58-2.90) - adjusted for gender). Interviews with pupils in the RIPPLE and SHARE studies identified the way in which SRE that was fun helped address the embarrassment and anxiety that could arise when talking about sex and relationships:

“I mean, the best kind of teachers are those who can mess about with you and be really open and still try to get the point across.” (RIPPLE; male pupil, 13y) (Forrest et al., 2002, p.203)

“… [jokey sex education] makes you understand it better as well because it’s not all serious… it’s a laugh and that’s the sort of thing that sticks in your head.” (SHARE; female pupil, 14y) (Buston et al., 2002a, p.328)

In other words, key social/professional skills such as creating fun and engaging group interactions are essential to the competent delivery of many behaviour change interventions.

Where programmes were delivered to groups of pupils, there remained an inherent challenge in delivering programmes that provided cognitive and emotional rewards to different types of pupils. In certain contexts, programme design could easily help these challenges to be overcome. For example, in the PhunkyFoods primary school programme, physical activities could be stratified in a way that enabled pupils of different physical abilities to participate (Teeman et al., 2008). However, in classroom settings with older pupils, where a degree of parity in understanding was necessary for groupwork and discussion to take place, teachers could struggle to deliver and coherently adapt content so that pupils of varying literacy and/or attention levels were kept engaged (Stead et al., 2007a).

A further type of difference in pupils was their experience or knowledge of areas of health. Teachers experienced difficulties in delivering programmes that assumed too little, or too much, pupil knowledge. In the Blueprint substance use education programme, some teachers’ experience of delivering the programme highlighted the way in which content on illegal substances ‘bewildered’ pupils aged 11-12 years (Stead et al., 2007a):

“… Yes they do need to know about it [crack cocaine] but when I was delivering it, there was a bemused sort of silence… We had all the details and things like poppers being used as a sex drug in the research, and I felt uncomfortable with that I must say. “ (Teacher) (Stead et al., 2007a, p.115)

The timing of SRE in the SHARE programme encountered similar issues, although was also perceived as ‘too little too late’ by the minority of more sexually-experienced pupils (Buston and Wight, 2002). Focus group data suggested that pupils were approximately equally divided in their views about whether the timing of the SHARE SRE programme was correct or not (Buston and Wight, 2002). The opportunity to practice skills in the classroom was valued by female pupils in the SHARE programme:

“… because you got the condom and the carrot and then inside was a leaflet so you read that through first and then we done it and that’s how you really sort of picked it up so I’d say that lesson was good.” (Female pupil, 16y, one sexual partner) (Buston and Wight, 2002, p.241)

Comparative analysis of survey data from the RIPPLE SRE programme found that over half of pupils in both trial arms would have liked to receive SRE earlier than the age at which RIPPLE was delivered (age 13 years) (Oakley et al., 2004).

Whatever the mode of delivery, the quality of the relationship between the people delivering the programme and the pupils participating in it was identified as important in both primary and secondary school settings. At the primary school level, interview data with staff suggested that engaging with pupils and striking a balance between discipline and enjoyment pivoted around developing a constructive relationship with pupils (Stathi and Sebire, 2011). At secondary school level, the way in which peer-educators acted when interacting with pupils about health issues was often perceived by pupils to be better than the likely behaviour of teachers (Audrey et al., 2006b, Strange et al., 2002b). Key attributes of peer-educators in programmes valued by pupils were similarities in values and language (Strange et al., 2002b), being less moralistic (Strange et al., 2002b), and being subtle in how health issues were approached, for example:

“They didn’t ask you directly, you know, ‘do you smoke?’… they just kind of brought it into the conversation, which made people feel sort of more comfortable about talking about it. So I think that was good how they did it.” (Female pupil) (Audrey et al., 2006a, p.325)

In the RIPPLE programme, peer-educators (who were two to three years older than the targeted pupils) were also described by pupils as being more respectful towards them than teachers (Forrest et al., 2002). Pupils also reported that peer-educators talked to them ‘as equals’:

“[Peer-educators] treated us as one of them really. Like, teachers say they’re always looking down on you – saying, you done that wrong, you ask this stupid question, but they seemed to treat us better…” (Female pupil, 13y) (Forrest et al., 2002, p.204)

Gender differences in within-class relationships could impact on modes of delivery of SRE programmes, although the picture is complex. Focus group data from the RIPPLE study showed that some female pupils felt inhibited from asking questions in mixed-sex SRE classes (Strange et al., 2002b), whilst data from interviews and focus groups in the SHARE study showed how both male and female pupils could be reluctant to ask questions that revealed either their naivety or experience to members of the opposite sex (Buston et al., 2002a). Whatever the source of differences in behaviour between male and female pupils, the enduring difficulties that they created in mixed-sex SRE classes is demonstrated in focus groups with male and female pupils in the SHARE programme:

“… we bring up anything… about sex or the body or anything… as soon as you like speak about these things to girls they get all bashful and don’t want to talk to you…” (Male pupils, 14-15y) (Buston et al., 2002a, p.324)

“[boys often] speak about pervert things… [the boys are] more explicit than the girls, you know, they don’t talk about feelings, it’s more like the physical.” (Female pupils, 14y) (Buston et al., 2002a, p.324)

In secondary school settings, where health promotion topics around substance use and sex and relationships by their very nature touch upon personal and sensitive topics, confidentiality was identified as an important aspect of programme delivery that goes some way beyond cognitive or emotional engagement. In short, pupils expressed a desire to be able to express themselves and ask questions without fear of retribution from teaching staff, fellow pupils, or external authorities. A variety of ways in which this trust could be gained were identified. For example, in the NE Choices substance use prevention programme, actors running a drama group as part of the programme were perceived as ‘more trustworthy’ than teaching staff by pupils (Stead et al., 2001). In the Blueprint substance use prevention programme, group work by pupils was perceived as being inhibited when groups comprised pupils who did not know each other well (Stead et al., 2007a). In the SHARE SRE programme, on the basis of class observations, interviews and focus groups, teachers’ role in assuring confidentiality for pupils was argued to hinge upon the way in which they implemented ground rules for group work and class control (Buston et al., 2002a).

## Findings: Programme theory 3

**The routine delivery (‘embedding’) of a programme takes time and motivation. It is likely to involve changes in the school environment and the development of new relationships between stakeholders that require pro-active management so that:**

- Different stakeholders’ goals are reconciled
- School staffs’ existing relationships with pupils are built upon
- Stakeholders’ enthusiasm, knowledge and experience are harnessed
- Knowledge of ‘core’ and ‘peripheral’ elements and minimum resources, skills and informational content is retained

No evidence identified for these sub-theories.

- Organisational decisions in other areas of school life are made taking into account how they impact on programme delivery

Evaluations of two programmes (ASSIST and UK Resilience) provide examples of negative impacts when organisational decisions do not take programme delivery into account. Whilst schools mostly facilitated the initial training of peer-supporters in the ASSIST programme, co-ordination of follow-up sessions to provide feedback for peer-supporters and address issues that had arisen, varied. For example, some schools scheduled follow-up sessions only during lessons such as PE (physical education) or CDT (craft, design and technology) that particularly appealed to pupils, and who did not want to miss these lessons (Holliday et al., 2009). The evaluation of the UK Resilience programme noted one school where pressure to increase pupils’ attainment in English led to the lead co-ordinator for the programme being required to relinquish their health promotion role in order to focus on their educational role (Challen et al., 2011).

- Responsibility for programme delivery becomes rooted in the school

No evaluations reported evidence to enable this sub-theory to be tested, but the perception of stakeholders are reported in evaluations of the Active Primary School and UK Resilience programmes. The view of some teachers who had delivered the UK Resilience programme was that ‘continual marketing’ of the programme within the school would be necessary for sustaining delivery, and that senior support may be an important component of this (Challen et al., 2009). In the Active Primary School programme, local authority managers involved with the programme perceived a need for a wider supportive infrastructure to ensure longer-term programme delivery. For example, the evaluation summarises these managers view that schools’ networking with other bodies such as local authority departments, NHS health promotion staff, and local clubs would be important (Lowden et al., 2004), but does not present supporting data.

- The longer-term sustainability of programmes and the extent to which health promoting messages and activities permeate other aspects of school life, is dependent on continuing feedback, encouragement and expectations about implementation. Over time, this may originate less from outside the school and more from inside.

Evaluations either did not investigate the longer-term sustainability of programmes, or only reported the views of stakeholders about what may help make programme delivery sustainable. A common theme was that consideration of sustainability was simply not part of either the programmes themselves or schools’ planning. This meant that teachers gave little consideration to how a programme could continue be linked into other school activities in the future (Crosswaite et al., 2004), perceived there to be no space in the school day (Teeman et al., 2008), or could only speculate on how a programme *might* usefully be incorporated into the curriculum (Warren et al., 2003). Where it was found that schools were positive about continuing programme delivery, the reasons for this were not explored or reported (Teeman et al., 2008). The ASSIST smoking prevention programme highlighted distinct issues for sustainability with regard to the use of peer-supporters, who reported finding it progressively harder to initiate conversations about smoking (Audrey et al., 2006a). The proportion of peer-supporters finding it ‘quite hard’ to engage peers in these conversation increased from 9.3% in week 1 to 19.2% in week 10 (Audrey et al., 2006a). Interview data suggests that the novelty of peer-supporters having taken part in training outside of school provided a useful point of interest for initiating conversations:

“Like we had two days off school [for the peer-supporter training] and then in the first week there was loads of people saying ‘oh, what did they teach you?’ and you had loads more conversations then.” (Male peer-supporter) (Audrey et al., 2006a, p.328)

However, this ‘hook’ faded fairly quickly, meaning that peer-supporters were sometimes at a loss for how to re-generate interest in the topic amongst their peers:

“I ended up talking to the same people again. Not many of my friends smoked or anything so it was just like telling them the facts and they got bored in the end.” (Female peer-supporter) (Audrey et al., 2006a, p.328)

## Findings: Programme theory 4

**The preparation for, introduction, initial delivery, and ongoing sustainability of a health promotion programme in a school is more likely to be successful when there is:**

- Specificity about ‘core’ (essential) and ‘peripheral’ (optional/adaptable) elements, including the minimum level of resources and/or skills necessary to support these elements (to inform decision-making about how to deliver a programme in the context of a particular school)
- Scope for ‘mutual adaptation’ between the programme and the people delivering it, including the evolution and updating of programme content and mode of delivery over time

Three programmes identified core and peripheral elements that were intended to help schools tailor programme delivery without losing the programme’s ‘active ingredients’. In the ASSIST programme, a ‘traffic light’ system was used to identify programme components that were essential (red), omittable if related activities had already been conducted (amber), and optional (green) (Audrey et al., 2004). A simpler classification of core and customisable elements was used in NE Choices and Project Tomato, although these were very broadly defined as core/customisable (NE Choices - drama workshop/ lesson plans; Project Tomato - lesson plans/ activities) (Christian, 2012, Stead et al., 2001). In most other programmes, all programme components were considered to be core, but there could be wide levels of variation in the fidelity of programme delivery. The PhunkyFoods programme was an exception for the way that there appeared to be little expectation that the lesson plans would be adhered to; unsurprisingly, there were considerable variations in implementation in all 18 schools in which the programme was delivered (Teeman et al., 2008).

The extent to which the identification of core and peripheral elements facilitated tailored programme delivery remains unclear, in particular as comparison between programmes is hindered by differences in how fully-developed programmes were. For example, teachers might struggle to deliver a less-developed programme as intended simply because allocated timings were unrealistic (e.g. Stead et al., 2007b, p.661) rather than because they were limited by the definition of core programme elements. Programme deliverers were also ambivalent about whether it was more useful to have tightly prescribed programmes (or lessons) or ones that identified components that could be tailored. The evaluation of the Blueprint substance use prevention programme identified initial resistance amongst teachers at the perceived prescriptive nature of the programme, but noted that this quickly changed once delivery started and the benefits of the detailed information and structure of the programme began to be appreciated (Stead et al., 2007a). Detailed lesson plans were also appreciated by teachers in the GGHB sexual health programme (Lowden and Powney, 1996) and PhunkyFoods programme (Teeman et al., 2008). However, trainers in the ASSIST programme expressed divergent views, with no pattern identifiable to explain the reasons for these differences. For example:

“… there were certain things that you couldn’t let drop. That inflexibility I found sort of hard, because then you were just delivering the programme regardless of the needs of the group.” (Trainer) (Holliday et al., 2009, p.57)

In contrast:

“You get stale if you have to deliver everything literally to the letter and it was nice to have a certain amount of flexibility to be able to do things differently… It just comes down to personality and the style of the trainers ultimately as to how it’s delivered.” (Trainer) (Holliday et al., 2009, p.57)

Evaluations of all programmes reported wide levels of variation in how programmes were delivered in certain schools, but all struggled to distinguish (or did not try to distinguish) between variation resulting from professional judgement about the particularities of situations and variation as a result of other constraints. Programme deliverers made changes to programmes in many ways, for example to:

- make them more suitable to pupils of lower academic abilities or where group work rates differed (Newman et al., 1991)
- use teaching resources that were considered more suitable and/or to fit better with regulatory authorities expectations (Challen et al., 2011)
- help pupils cope better with difficult or contentious health topics (Stead et al., 2007a)
- facilitate group work in friendship groups (Stead et al., 2007a)
- fit into available timetable slots (Teeman et al., 2008)
- improvise session content to accommodate the schools’ environmental or staffing conditions (Stathi and Sebire, 2011)

In some instances where programmes had been developed by specialists, some teachers paid close attention to delivering a programme as planned (Challen et al., 2011), and/or consulting the programme designers about proposed modifications (Buston et al., 2002b). However, this relationship is not consistent, as other teachers believed that their professional autonomy should allow them to modify a programme without consultation (Buston et al., 2002b). Data from the eight schools in which programme implementation was evaluated in the SHARE SRE programme suggests that fidelity of delivery is linked to broader issues around team cohesion (Buston et al., 2002b). Where senior and junior staff shared a common goal of programme delivery, were supported to achieve this goal, and felt able to openly discuss issues around programme delivery in a supportive atmosphere, programme components were delivered with greater fidelity (Buston et al., 2002b).

References

AUDREY, S., CORDALL, K., MOORE, L., COHEN, D. & CAMPBELL, R. 2004. The Development and Implementation of a Peer-Led Intervention to Prevent Smoking among Secondary School Students Using Their Established Social Networks. *Health Education Journal,* 63**,** 266-284.

AUDREY, S., HOLLIDAY, J. & CAMPBELL, R. 2006a. It's good to talk: Adolescent perspectives of an informal, peer-led intervention to reduce smoking. *Social Science & Medicine,* 63**,** 320-334.

AUDREY, S., HOLLIDAY, J. & CAMPBELL, R. 2008. Commitment and compatibility: teachers' perspectives on the implementation of an effective school-based, peer-led smoking intervention. *Health Education Journal,* 67**,** 74-90.

AUDREY, S., HOLLIDAY, J., PARRY LANGDON, N. & CAMPBELL, R. 2006b. Meeting the challenges of implementing process evaluation within randomized controlled trials: the example of ASSIST (A Stop Smoking in Schools Trial). *Health Educ Res,* 21**,** 366-377.

BLUEPRINT EVALUATION TEAM 2007. Blueprint Drugs Education: The Response of Pupils and Parents to the Programme. London: Home Office.

BUSTON, K. & HART, G. 2001. Heterosexism and Homophobia in Scottish School Sex Education: exploring the nature of the problem. *Journal of Adolescence,* 24**,** 95-109.

BUSTON, K. & WIGHT, D. 2002. The salience and utility of school sex education to young women. *Sex Education,* 2**,** 233-250.

BUSTON, K., WIGHT, D. & HART, G. 2002a. Inside the sex education classroom: the importance of class context in engaging pupils. *Culture, Health and Sexuality,* 4**,** 317-335.

BUSTON, K., WIGHT, D., HART, G., SCOTT, S., BUSTON, K., WIGHT, D., HART, G. & SCOTT, S. 2002b. Implementation of a teacher-delivered sex education programme: obstacles and facilitating factors. *Health Education Research,* 17**,** 59-72.

BUSTON, K., WIGHT, D. & SCOTT, S. 2001. Difficulty and Diversity: the context and practice of sex education. *British Journal of Sociology of Education,* 22**,** 353-268.

CHALLEN, A., NODEN, P., WEST, A. & MACHIN, S. 2009. UK Resilience Programme evaluation: interim report (Research report DCSF-RR094). London: Department for Children, Schools and Families.

CHALLEN, A., NODEN, P., WEST, A. & MACHIN, S. 2011. UK Resilience Programme: final report. London: Department for Education.

CHRISTIAN, M. S., EVANS, C. E., RANSLEY, J. K., GREENWOOD, D. C., THOMAS, J. D., CADE, J. E., 2012. Process evaluation of a cluster randomised controlled trial of a school-based fruit and vegetable intervention: Project Tomato. *Public Health Nutrition,* 15**,** 459-465.

CROSSWAITE, C., TOOBY, J. & CYSTER, R. 2004. SPICED: evaluation of a drug education project in Kirklees primary schools... Schools Partnership in Children's Education on Drugs. *Health Education Journal,* 63**,** 61-69.

FORREST, S., STRANGE, V. & ANN, O. 2002. A comparison of students' evaluations of a peer-delivered sex education programme and teacher-led provision. *Sex Education,* 2**,** 195-214.

HOLLIDAY, J., AUDREY, S., MOORE, L., PARRY-LANGDON, N. & CAMPBELL, R. 2009. High Fidelity? How Should We Consider Variations in the Delivery of School-Based Health Promotion Interventions? *Health Education Journal,* 68**,** 44-62.

LOWDEN, K. & POWNEY, J. 1996. An Evolving Sexual Health Education Programme: From Health Workers to Teachers. Glasgow: The Scottish Council for Research in Education.

LOWDEN, K., QUINN, J. & KIRK, S. 2004. Evaluation of the Active Primary School Pilot Programme: Research Report no.90. Edinburgh: SportScotland.

NEWMAN, R. & NUTBEAM, D. 1989. Teachers' views of the Family Smoking Education Project. *Health Education Journal,* 48**,** 9-13.

NEWMAN, R., SMITH, C. & NUTBEAM, D. 1991. Teachers' views of the 'Smoking And Me' project. *Health Education Journal,* 50**,** 107-110.

OAKLEY, A., STRANGE, V., STEPHENSON, J., FORREST, S. & MONEIRO, H. 2004. Evaluating processes: a case study of a randomized controlled trial of sex education. *Evaluation,* 10**,** 440-462.

ROTHWELL, H. & SEGROTT, J. 2011. Preventing alcohol misuse in young people aged 9-11 years through promoting family communication: an exploratory evaluation of the Kids, Adults Together (KAT) Programme. *BMC Public Health,* 11**,** 810.

SAHOTA, P., RUDOLF, M. C., DIXEY, R., HILL, A. J., BARTH, J. H., CADE, J., SAHOTA, P., RUDOLF, M. C., DIXEY, R., HILL, A. J., BARTH, J. H. & CADE, J. 2001. Evaluation of implementation and effect of primary school based intervention to reduce risk factors for obesity. *BMJ,* 323**,** 1027-1029.

STATHI, A., NORDIN, S. & RIDDOCH, C. 2006. Evaluation of the 'Schools on the Move' project. London: Middlesex University.

STATHI, A. & SEBIRE, S. J. 2011. A process evaluation of an outreach physical activity program in an inner-city primary school. *Journal of Physical Activity & Health,* 8**,** S239-S248.

STEAD, M., MACKINTOSH, A. M., EADIE, D. & HASTINGS, G. 2001. NE Choices: the results of a multi-component drug prevention programme for adolescents (DPAS paper no 14). London: Home Office.

STEAD, M., STRADLING, B., MACKINTOSH ANNE, M., MACNEIL, M., MINTY, S. & EADIE, D. 2007a. Delivery of the Blueprint programme: report. Stirling: University of Stirling.

STEAD, M., STRADLING, R., MACNEIL, M., MACKINTOSH, A. M., MINTY, S., STEAD, M., STRADLING, R., MACNEIL, M., MACKINTOSH ANNE, M. & MINTY, S. 2007b. Implementation evaluation of the Blueprint multi-component drug prevention programme: fidelity of school component delivery. *Drug & Alcohol Review,* 26**,** 653-664.

STRANGE, V., FORREST, S. & OAKLEY, A. 2002a. Peer-led sex education - characteristics of peer educators and their perceptions of the impact on them of participation in a peer education programme. *Health Education Research,* 17**,** 327-337.

STRANGE, V., FORREST, S. & OAKLEY, A. 2002b. What influences peer-led sex education in the classroom? A view from the peer educators. *Health Education Research,* 17**,** 339-349.

TEEMAN, D., REED, F., BIELBY, G., SCOTT, E. & SIMS, D. 2008. Evaluation of the PhunkyFoods Programme. Final Report. Slough: National Foundation for Educational Research.

WARREN, J. M., HENRY, C. J., LIGHTOWLER, H. J., BRADSHAW, S. M., PERWAIZ, S., WARREN, J. M., HENRY, C. J. K., LIGHTOWLER, H. J., BRADSHAW, S. M. & PERWAIZ, S. 2003. Evaluation of a pilot school programme aimed at the prevention of obesity in children. *Health Promotion International,* 18**,** 287-296.

WIGHT, D. & ABRAHAM, C. 2000. From Psycho-Social Theory to Sustainable Classroom Practice: Developing a research-based teacher-delivered sex education programme. *Health Education Research: Theory and Practice,* 15**,** 25-38.

WIGHT, D. & BUSTON, K. 2003. Meeting needs but not changing goals: evaluation of inservice teacher training for sex education. *Oxford Review of Education,* 29**,** 521-543.

WIGHT, D., RAAB, G., HENDERSON, M., ABRAHAM, C., BUSTON, K. & HART G SCOTT, S. 2002. The limits of teacher-delivered sex education: interim behavioural outcomes from a randomised trial. *British Medical Journal,* 324**,** 1430-1433.

WYATT, K. M., LLOYD, J. J., CREANOR, S. & LOGAN, S. 2011. The development, feasibility and acceptability of a school-based obesity prevention programme: results from three phases of piloting. *BMJ Open,* 1**,** e000026.
